# Integrated Genomic Profiling and Drug Screening of Patient-Derived Cultures Identifies Individualized Copy Number-Dependent Susceptibilities Involving PI3K Pathway and 17q Genes in Neuroblastoma

**DOI:** 10.3389/fonc.2021.709525

**Published:** 2021-10-14

**Authors:** Rachel L. Y. Wong, Megan R. E. Wong, Chik Hong Kuick, Seyed Ehsan Saffari, Meng Kang Wong, Sheng Hui Tan, Khurshid Merchant, Kenneth T. E. Chang, Matan Thangavelu, Giridharan Periyasamy, Zhi Xiong Chen, Prasad Iyer, Enrica E. K. Tan, Shui Yen Soh, N. Gopalakrishna Iyer, Qiao Fan, Amos H. P. Loh

**Affiliations:** ^1^ Duke NUS Medical School, Singapore, Singapore; ^2^ VIVA-KKH Paediatric Brain and Solid Tumour Programme, Children’s Blood and Cancer Centre, KK Women’s and Children’s Hospital, Singapore, Singapore; ^3^ Department of Pathology and Laboratory Medicine, KK Women’s and Children’s Hospital, Singapore, Singapore; ^4^ Centre for Quantitative Medicine, Duke NUS Medical School, Singapore, Singapore; ^5^ Centre for High Throughput Phenomics (CHiP-GIS), Genome Institute of Singapore, Singapore, Singapore; ^6^ Department of Physiology, National University of Singapore, Singapore, Singapore; ^7^ Department of Paediatric Subspecialties Haematology Oncology Service, KK Women’s and Children’s Hospital, Singapore, Singapore; ^8^ Division of Medical Sciences, National Cancer Centre, Singapore, Singapore; ^9^ Department of Paediatric Surgery, KK Women’s and Children’s Hospital, Singapore, Singapore

**Keywords:** neuroblastoma, patient-derived culture, copy number variations (CNV), Comprehensive genomic profiling (CGP), PI3K - AKT pathway, CDK (cyclin-dependent kinase), JAK-STAT cascade

## Abstract

Neuroblastoma is the commonest extracranial pediatric malignancy. With few recurrent single nucleotide variations (SNVs), mutation-based precision oncology approaches have limited utility, but its frequent and heterogenous copy number variations (CNVs) could represent genomic dependencies that may be exploited for personalized therapy. Patient-derived cell culture (PDC) models can facilitate rapid testing of multiple agents to determine such individualized drug-responses. Thus, to study the relationship between individual genomic aberrations and therapeutic susceptibilities, we integrated comprehensive genomic profiling of neuroblastoma tumors with drug screening of corresponding PDCs against 418 targeted inhibitors. We quantified the strength of association between copy number and cytotoxicity, and validated significantly correlated gene-drug pairs in public data and using machine learning models. Somatic mutations were infrequent (3.1 per case), but copy number losses in 1p (31%) and 11q (38%), and gains in 17q (69%) were prevalent. Critically, *in-vitro* cytotoxicity significantly correlated only with CNVs, but not SNVs. Among 1278 significantly correlated gene-drug pairs, copy number of GNA13 and DNA damage response genes CBL, DNMT3A, and PPM1D were most significantly correlated with cytotoxicity; the drugs most commonly associated with these genes were PI3K/mTOR inhibitor PIK-75, and CDK inhibitors P276-00, SNS-032, AT7519, flavopiridol and dinaciclib. Predictive Markov random field models constructed from CNVs alone recapitulated the true z-score-weighted associations, with the strongest gene-drug functional interactions in subnetworks involving PI3K and JAK-STAT pathways. Together, our data defined individualized dose-dependent relationships between copy number gains of PI3K and STAT family genes particularly on 17q and susceptibility to PI3K and cell cycle agents in neuroblastoma. Integration of genomic profiling and drug screening of patient-derived models of neuroblastoma can quantitatively define copy number-dependent sensitivities to targeted inhibitors, which can guide personalized therapy for such mutationally quiet cancers.

## Introduction

Neuroblastoma is the most common pediatric extracranial malignant tumor and is responsible for a disproportionate 15% of all childhood cancer deaths. Despite current intensive multimodal therapy for patients with high-risk disease, 5-year survival remains at 30-50% even after decades of international multicenter trials. Due to its histologic and biologic heterogeneity, the classic stage- and risk-based clinical trial approach cannot adequately match treatments to the diverse individual susceptibilities of each patient’s tumor, and is further limited by the small numbers of patients in each subgroup (1). Thus, therapeutic advancements for neuroblastoma may be better realized with a personalized approach.

Precision oncology approaches based on identification of targetable single nucleotide variants (SNVs) have limited usefulness in embryonal tumors because of their low mutational burden (2). Instead, copy number variations (CNVs) are more prevalent and are stronger prognostic factors in neuroblastoma (2–4). In particular, segmental chromosomal aberrations (SCAs) are associated with advanced disease stage and poorer prognosis. This corroborates with recent evidence that the pathogenicity of CNVs correlate with dosage sensitivity of involved genes, and are enriched for embryonal neurodevelopmental functions (5). This suggests that gene copy number could be used to predict and select targeted therapies, especially in embryonal tumors of childhood (6). While current sequencing-based panels may be an efficient and cost-effective manner to perform clinical genomic profiling, most are designed for adult cancers or hematological malignancies. Recently, the Oncomine Childhood Cancer Research Assay (OCCRA) was developed as a diagnostic-grade genomic profiling tool curated specifically for pediatric cancers (7, 8). This is a promising new resource to detect significant and potentially actionable SNVs and CNVs in neuroblastoma.

Individualized preclinical tumor models can be a complementary means to rapidly test drug therapies *ex vivo*, and have been able to successfully uncover potential therapeutic leads (9, 10). While this has been employed extensively in epithelial carcinomas, embryonal tumors like neuroblastoma have not been consistently engrafted as personalized *in vitro* models. Commercial cell lines are often significantly changed with multiple serial passages or have been contaminated over time, and do not capture individual patient genetic or phenotypic differences in treatment response (11–14). We previously developed multi-lineage patient-derived cell cultures (PDCs) of neuroblastoma from pre- and post-treatment tumors and demonstrated their recapitulation of original tumors’ chromosomal alterations, immunohistochemical and gene expression profiles, and ability to predict individualized responses to standard-of-care chemotherapy (15). However, the utility of patient-derived models for prediction of targeted therapies for neuroblastoma have not been well studied.

We hypothesize that in neuroblastoma, gene copy number and *in vitro* cytotoxicity to corresponding targeted inhibitors display distinct relationships in a dose-dependent manner. To study key genotype-phenotype correlations in neuroblastoma, we characterized SNVs and CNVs of neuroblastoma tumors using clinical genomic profiling and interrogated corresponding PDCs with a medium-throughput inhibitor screen, then correlated resulting genomic and phenotypic readouts. We then defined the quantitative associations between these genomic aberrations and the corresponding responses to targeted agents as a framework to potentially guide personalized targeted therapeutic options, particularly for patients with few or no targetable mutations.

## Materials and Methods

### Patients and Tumor Samples

Patients with neuroblastoma were prospectively recruited at KK Women’s and Children’s Hospital with Institutional Review Board approval (CIRB 2014/2079). Written consent from parents, and assent from children were obtained. Criteria for study inclusion were: patients under undergoing surgical biopsies or resections, with available excess tumor tissue; male and female patients with neuroblastoma aged 1–8 were included. No subject attrition was encountered, no randomization or power calculation was required, and investigators were blinded to patient identities. Excess tumor tissue from routine surgical procedures were transported on ice on the same day to the VIVA-KKH Paediatric Solid Tumour Laboratory for generation of PDCs. Corresponding tumor aliquots were snap frozen for molecular analysis.

### OCCRA Comprehensive Genomic Profiling

Access to OCCRA (RRID : SCR_007834) (Thermo Fisher Scientific, Waltham, MA) was granted *via* an early access program. The DNA assay was utilized, which calls SNVs from hotspots of 86 genes and full exons of 44 genes, and CNVs from 28 genes. DNA was extracted from macro-dissected tissue using ReliaPrep FFPE DNA extraction kit (Promega, Madison, WI, USA). OCCRA primers and AmpliSeq Library Kit Plus (Illumina, San Diego, California, USA) were used for library preparation. The prepared libraries were sequenced using a MiniSeq sequencer (Illumina). Base calling and mapping to a reference genome (hg19) was performed using the BaseSpace Informatics suite (Illumina). SNV variant calling was performed with the DNA amplicon application (Illumina, version 2.1.1) and CNV calling was performed with the OncoCNV caller application (Illumina, version 1.2.0). All VCF files were loaded into variant interpreter (version 2.7.0.412) for interpretation. Criteria for selecting somatic SNV candidates were: >100 minimum coverage reads, 10% minimum allele frequency, cosmic reported variant, and <1% prevalence in the 1000 Genome Population Database.

### Cell Culture

PDCs were generated as previously described by explant culture under growth conditions of 37°C with 5% CO_2_ (15), and passaged at 1:2 split ratio upon reaching 80-100% confluence (15). For each PDC line, optimal seeding density to achieve 72-hour log-phase growth was determined. Cells of each PDC line were seeded on clear bottom white 96-well plates in triplicate (Corning, Cat 3903) in a 1:2 split ratio and assessed for appropriate responses to positive and negative control agents 0.5% DMSO and 1μM Staurosporine over 72-hours using IncuCyte^®^ S3 Live-Cell Analysis System (RRID : SCR_019874) (Essen BioScience, Sartorius, Japan) and IncuCyte Base Software (v.2018B), using default analysis settings.

### Short Tandem Repeat Fingerprinting

STR genotyping of all tumor-PDC pairs was performed using PowerPlex^®^ 21(Promega, Cat DC8902) using 1ng DNA. PCR products were resolved in an Applied Biosystems^®^ SeqStudio genetic analyzer and compared.

### Medium-Throughput Screening Assay

Selleckchem Kinase Inhibitor Library (SelleckChem, Cat. L1200), consisting of 418 tyrosine kinase inhibitors, was reformatted into 96-well format and diluted with DMSO to achieve final concentrations of 1uM. Daughter plates were stored at -20°C and underwent no more than 15 freeze-thaw cycles.

Cells were seeded in a 1:2 dilution factor on 96-well white bottom plates (Corning, Cat 3917) using MultiFlo™ FX (RRID : SCR_019746) (Biotek, Winooski VT). Plates were incubated for 24 hours at 37°C with 5% CO_2_, and 0.5μL of each drug was added to the corresponding wells using Bravo BenchCel Workstation (RRID : SCR_019468) (Agilent, Sata Clara CA) equipped with automated liquid and microplate handling. Treated plates were centrifuged for 1 minute at 1000rpm before and incubated for the next 72 hours. Cytotoxicity was determined using CellTiter-Glo^®^ Luminescent Cell Viability Assay (Promega, Cat G7572). Media was aspirated before adding 50uL of CellTiter-Glo reagent prepared as per manufacturer’s protocol. Plates were shielded from light and agitated at 300rpm for 15 minutes; bioluminescence was measured using Infinite M1000 series microplate reader (Tecan, Mannerdorf, Switzerland) using a luminescence integration time of 250ms. Relative light units (RLUs) were normalized against DMSO negative controls of each corresponding plate to obtain normalized cytotoxicity values. Screening runs were validated only if high cytotoxicity to positive control staurosporine (1μM) and minimal DMSO effect were observed

### Data Analysis

Clustering of copy number and z-score data was performed on ComplexHeatmap (v.2.0.0, RRID : SCR_017270) using R 3.6.1 with complete Euclidean clustering applied.

Pearson’s correlation coefficients were calculated for each of 56,848 gene-drug pairs, comparing cytotoxicity and SNV, and cytotoxicity and copy number, using Fisher’s z-transformation approach. Positive or negative drug-gene correlations were obtained for all 56,848 gene-drug combinations for both mutational variants and gene copy number. False discovery rates (FDR) for the Benjamini-Hochberg procedure were set at 0.1 as a threshold of statistical significance of gene-drug pairs (16). Data analysis was performed using R 4.0.3.

### Probabilistic *In Silico* Model

Probabilistic Graphical Models (PGMs) were constructed with ReactomeFIViz (v. 7.2.3) using Reactome Functional Interaction (FI) network version 2018, which utilizes an adaptation of the PARADIGM approach (17–19) (RRID : SCR_009634). Briefly, this infers case-specific genetic variations, incorporating information from curated Reactome pathways, converting these reactions into factors then modeling the variations in each gene as constraint graphs which represent them as probability distribution functions. CNVs were input as continuous observation variables without discretizing and pairwise Markov random field models using empirical distributions were constructed over 100 permutations. Genes with mean differences in protein impact scores of <-0.01 and >0.01 between real and random samples were selected; genes with no CNV were removed. The PGM network was overlaid with associated Cancer Targetome drugs (20). A corresponding network was constructed from gene-drug pairs weighted according to z-scores, using Cytoscape (ver 3.8.0, RRID : SCR_003032). Both networks were clustered according to Reactome FI scores. Sub-networks (Reactome FI Modules) were annotated for significantly enriched pathways from CellMap (RRID : SCR_010642), Reactome, KEGG (RRID : SCR_012773), NCI Pathway Interaction Database, Protein ANalysis THrough Evolutionary Relationships (PANTHER), and BioCarta databases (RRID : SCR_006917).

## Results

### Molecular Profiling of Neuroblastoma Identifies Few Actionable Pathogenic Mutations but Multiple Copy Number-Altered Genes Involved in Transcriptional Regulation

**Figure 1:**
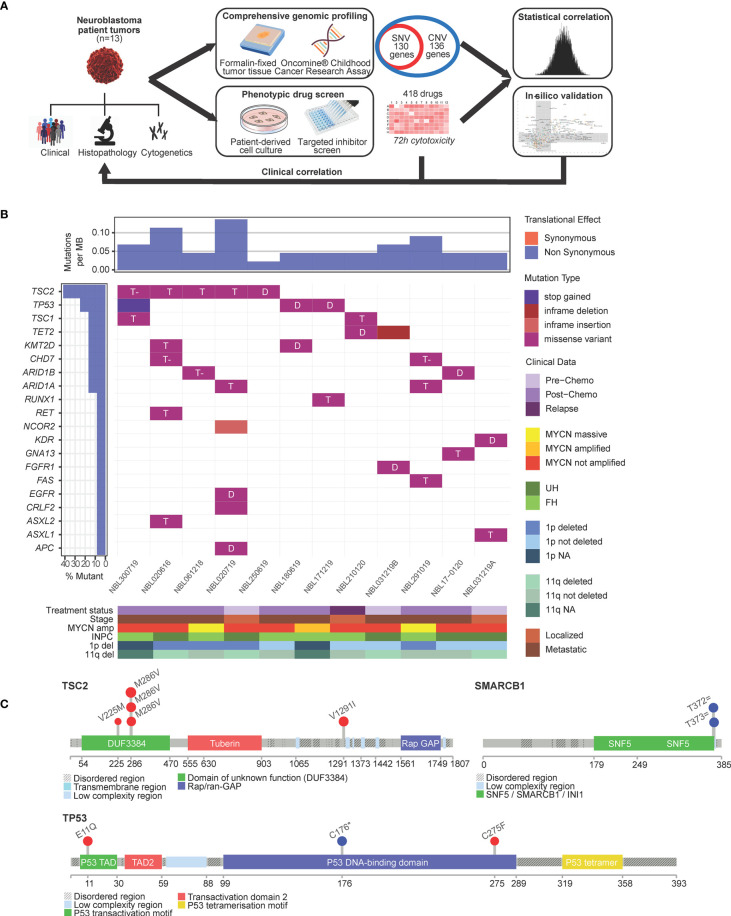
Single nucleotide variants in neuroblastoma are largely not actionable. **(A)** Schema of experimental protocol. **(B)** Waterfall plot of the distribution of mutations found in 13 neuroblastoma tumors, filtered for population SNPs, reflecting mutation type predicted according to Sift in each identified variant (main plot), frequency of mutations per sample (upper panel), frequency of samples mutated (left panel) and clinical-pathological covariates (lower panel heatmap). T, tolerated; T- tolerated (low); D, deleterious; INPC, International Neuroblastoma Pathology Classification; UH, unfavorable histology; FH, favorable histology. **(C)** Lollipop plots showing overall low frequency of mutations but occuring in expected hospots: TSC2 mutations in the DUF3384/TSC1-binding domain and TP53 variants in the P53 DNA-binding hotspot region; germline splice variants in SMARCB1 occurred in the coiled-coil C-terminal domain of the BAF47 subunit associated with mutations of SWI/SNF genes.

Fresh tumor specimens were obtained from surgical biopsies and resections of 13 patients with neuroblastoma following informed consent. Median age was 2.65 (range: 1.45–7.80). The study population appropriately demonstrated the expected spectrum of pathological and cytogenetic risk features in neuroblastoma: 9/13 (70%) had stage 4 disease and 3/13 (23%) had MYCN amplification (**Supplementary Table S1**). Tumor specimens were subjected to genomic profiling while corresponding PDCs were subjected to phenotypic drug-response testing (**Figure 1A**).

Genomic profiling was performed using the OCCRA DNA panel. Among 130 genes interrogated for SNVs, 71 separate SNVs in 12 patients fulfilled criteria as sequence variants of IARC Class 3 and above according to American College of Medical Genetics standards (21); 27 of 71 SNVs were recurrent single nucleotide polymorphisms (SNPs) frequently encountered in our local population and excluded. Of the remaining 44 SNVs, 34 were Indels, amounting to a mean incidence of 3.1 mutations per case (**Figure 1B**). The most frequent mutations were encountered in TSC2, TP53, ARID1A and ARID1B (**Supplementary Table S2**). Among these, 11 were predicted by Sift scores to be deleterious (**Figure 1B**), and by PolyPhen scores, 5 as probably damaging and 4 as possibly damaging. TSC2 and TP53 mutations were observed in recognized hotspots (21) (**Figure 1C**). Two patients had germline splice variants of SMARCB1, though neither exhibited clinical phenotype of the associated Coffin-Siris syndrome (22) (**Figure 1C**).

**Figure 2:**
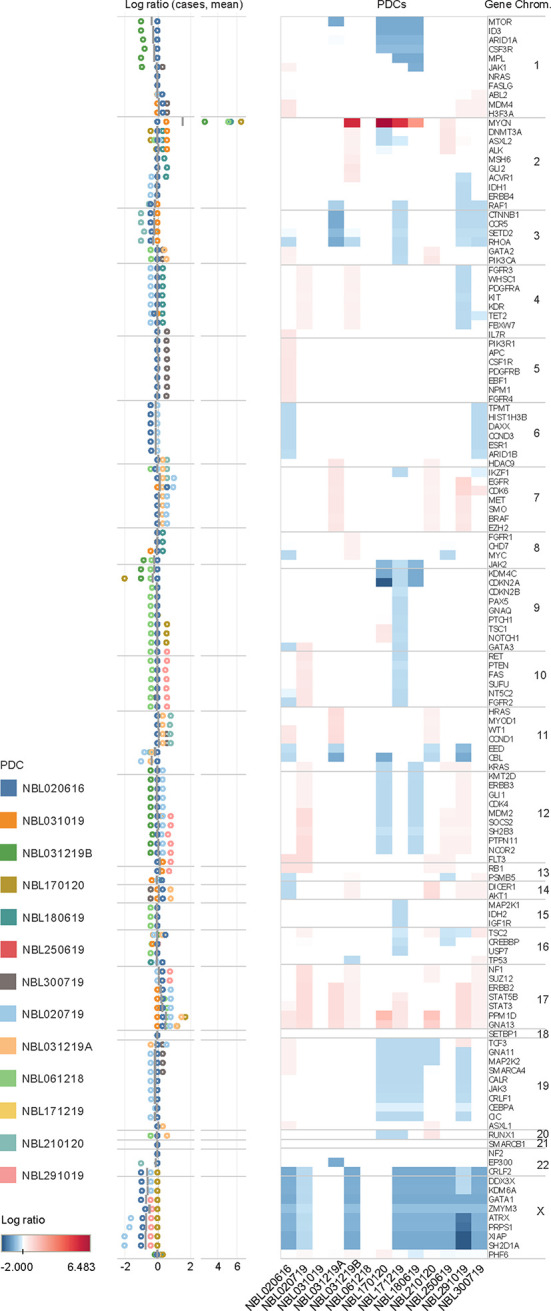
Copy number variations in neuroblastoma. Copy number log ratio alterations of 136 genes in 13 neuroblastoma tumor samples in upper panel, and distribution of log ratios of individual cases (circles) and mean log ratios (horizontal bars) in lower panel. Mean copy number losses are observed in chromosomes 1p (MTOR, ID3, ARID1A, CSF3R, MPL, JAK1), 3p (CCNB1, CCR5,SETD2, RHOA), 8q (JAK2), 9p (KDM4C, CDKN2A), and 11q (EED, CBL); with mean gains in chromosomes 7 (EGFR, CDK6), and 17 (STAT5B, STAT3, PPM1D, GNA13). Gains are in red and losses in blue.

To profile CNVs, normalized copy number of all 2998 probes were segmented to derive the mean copy number log_2_ ratios of 136 genes (**Supplementary Table S3**). Characteristic copy number losses in 1p [n=4 (31%)] and 11q [n=5 (38%)], and gains in 17q [n=9 (69%)] were observed (**Figure 2**). No CNVs were detected in 5 genes: FASLG, NF2, NRAS, SETBP1, SMARCB1. On unsupervised clustering, only MYCN clustered significantly from the copy number profiles of the other genes, with no significant clustering of other clinical covariates by mean copy number (**Supplementary Figure S1**). Of 4 patients with MYCN gain, 3 had massive amplifications (>10 copies) and 3 had 1p deletion. Both findings were separately verified on FISH. Among the genes with the highest mean copy numbers were transcription factors PPM1D, GNA13, STAT3 and STAT5B (log_2_ ratios 0.568, 0.442, 0.228, 0.200, respectively) – all sited on chromosome 17q. Lowest copy number log_2_ ratios were seen in X chromosome genes ATRX (-0.666) and XIAP, and transcriptional regulators GATA1 (-0.678) and SH2D1A (-0.720 each).

### High Throughput Screening of Neuroblastoma PDCs Reveals Drug Hits Targeting key Molecular Pathways Implicated in Neuroblastoma

**Figure 3:**
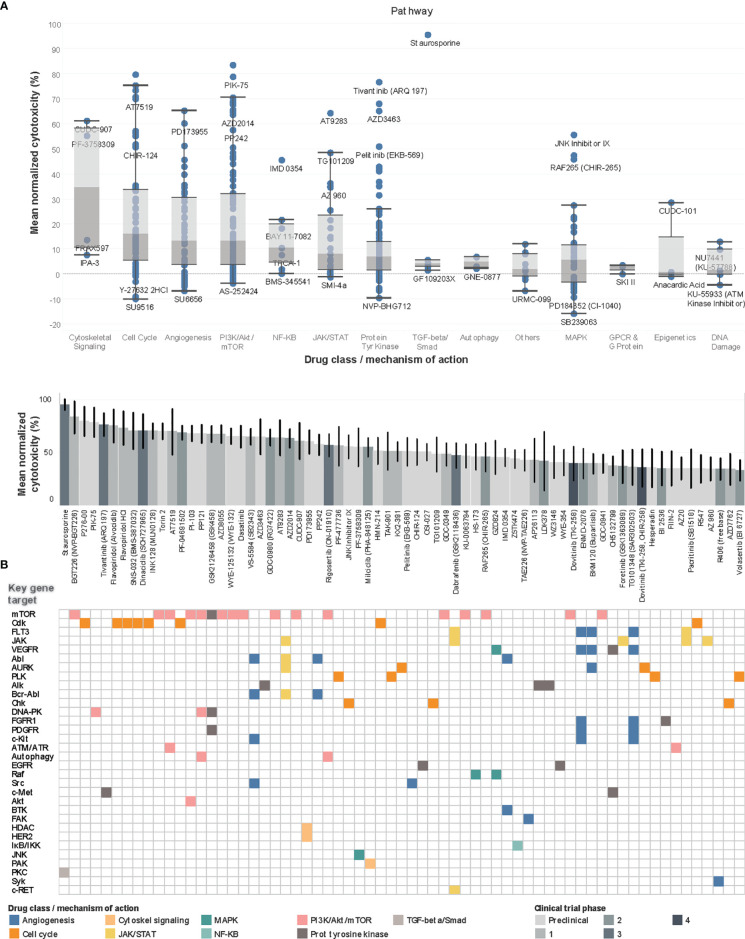
Drug screen of neuroblastoma PDCs identify agents targeting PI3K pathway and cell cycle. **(A)** Boxplots of drug classes of all 418 compounds, ranked by mean normalized cytotoxicity, with drug names of outliers labelled. **(B)** Waterfall plot of top 70 drug hits with >33% cytotoxicity in top panel, with current clinical trial phase indicated in shades of grey; bottom panel reflects corresponding key gene targets and main mechanism of action. Among the 70 top drug hits, 18 (26%) had progressed beyond Phase 1 trials. The 5 top drug hits included BGT226, a class I PI3K/mTOR inhibitor for PI3Kα/β/γ; P276-00, a Cdk inhibitor; PIK-75, a PI3K inhibitor; and tivantinib, a c-Met receptor tyrosine kinase non-ATP-competitive inhibitor (83.6%, 79.7%, 78.9%, 76.7% mean normalized cytotoxicity, respectively).

Concurrently, PDCs were generated from the same 13 tumors and their recapitulation of the corresponding original tumors were validated as previously described (15) (**Supplementary Data 3**). To evaluate the *ex vivo* drug response phenotype, PDCs in log-phase growth were screened against an established 418-compound targeted inhibitor library and cytotoxicity normalized against DMSO negative controls (see **Supplementary Data 4**). The compound library was restricted to kinase inhibitors, to constrain target selectivity and limit off-target effects. Drug classes associated with the highest mean normalized cytotoxicity targeted cytoskeletal signaling, cell cycle, angiogenesis and PI3K/Akt/mTOR pathway (**Figure 3A**).

Overall, 351 (84.0%) compounds showed positive mean normalized cytotoxicity, among which 70 (16.7%) compounds had >33% cytotoxicity and were profiled as hits. To characterize the expected molecular targets, drugs were mapped to corresponding genes and signaling pathways according to drug library specifications. The commonest genes targeted by the 70 hits included mTOR [n=17 (24%)], Cdk [n=8, (11%)], FLT3 and JAK, [n=5 (7%), each] (**Figure 3B**). Correspondingly, among the identified drug hits were pan-CDK inhibitors flavopiridol and dinaciclib which are in early phase trials for neuroblastoma and advanced solid tumors (23–25), and mTOR ATP-competitive inhibitor INK128 (70.5% mean cytotoxicity) and PI3K inhibitor PIK-75, both with known preclinical activity against neuroblastoma (26, 27).

To determine the clinical significance of drug screen results, we correlated cytotoxicity and clinical variables using non-hierarchical unsupervised clustering, but no significant associations between PDC drug-response profiles and clinical variables were observed (**Supplementary Figure S2**) (28).

### Correlation of Cytotoxicity and Copy Number Identify Mostly Novel Gene-Drug Associations

**Figure 4:**
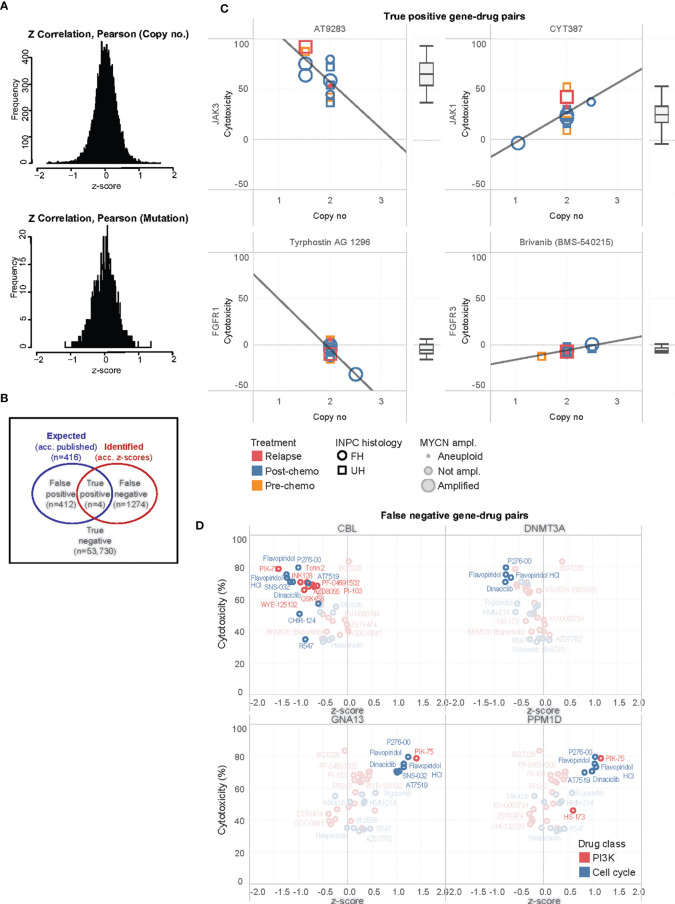
Correlation of significantly associated gene-drug pairs. **(A)** Distributions of z-scores of Pearson correlation coefficients for correlations of cytotoxicity versus mutational variants and gene copy number among 54,758 gene-drug combinations. **(B)** Venn diagram illustrating distribution of true positive, false positive, false negative, and true negative gene-drug pairs. **(C)** Scatterplots of cytotoxicity against copy number for 4 true positive gene-drug pairs across 13 PDC lines, ranked by median cytotoxicity which is represented in the adjacent boxplots. Clinical-pathological characteristics of individual PDC lines are reflected in legend. INPC, International Neuroblastoma Pathology Classification; UH, unfavorable histology; FH, favorable histology. **(D)** Scatterplots of mean cytotoxicity and z-scores of top-ranked false negative gene-drug pairs of PI3K and cell cycle drugs and CBL, DNMT3A, GNA13 and PPM1D. Gene-drug pairs significantly correlated at 0.1 FDR are in bold while non-significant pairs are ghosted.

Having separately characterized the molecular aberrations of the patient tumors and the drug response profiles of their PDCs, we then sought to correlate both genomic and phenotypic readouts. Copy number and mutation calls of 131 genes and normalized cytotoxicity to 418 compounds were compared to determine significantly correlated gene-drug pairs. The z-score of Pearson correlation coefficients of 54,758 possible gene-drug combinations were normally distributed for both copy number and SNVs (**Figure 4A**). Selecting for significantly-correlated gene-drug pairs at 0.1 false discovery rate (FDR) cutoff, copy number and cytotoxicity were significantly correlated in 1278 gene-drug combinations (**Supplementary Figure S3**). Among these were 49 hit compounds with >33% mean cytotoxicity, which involved 159 significantly correlated gene-drug pairs (**Table 1**).

**Table 1:** Pairs of drug hits and corresponding genes with significant association between cytotoxicity and copy number.

Drug	Mechanism of action	Mean cytotoxicity (S.D.)	Gene (mean copy number, z-score)
Staurosporine	TGF-beta/Smad	95.7 ( ± 5.5)	GNA13 (2.9, 0.73); ABL2 (2, -0.81); GNA11 (1.8, 0.86)
BGT226 (NVP-BGT226)	PI3K/Akt/mTOR	83.6 ( ± 16)	GNA11 (1.8, 0.86); MAP2K2 (1.8, 0.86); MPL (1.8, 0.82); JAK1 (2, 0.72)
P276-00	Cell Cycle	79.7 ( ± 14.5)	GNA13 (2.9, 1.23); PPM1D (3.2, 1.05); ASXL2 (2.1, -0.73); DNMT3A (2.1, -0.77); CBL (1.7, -1.01)
PIK-75	PI3K/Akt/mTOR	78.9 ( ± 14.5)	GNA13 (2.9, 1.4); PPM1D (3.2, 1.16); BRAF (2.2, 0.74); EZH2 (2.2, 0.74); MET (2.2, 0.74); SMO (2.2, 0.74); EED (1.8, -0.89); CBL (1.7, -1.41)
Tivantinib (ARQ 197)	Protein T.K.	76.7 ( ± 11.2)	GNA13 (2.9, 0.88); PPM1D (3.2, 0.75); DNMT3A (2.1, -0.72); CBL (1.7, -0.99)
Flavopiridol (Alvocidib)	Cell Cycle	75.4 ( ± 16.4)	GNA13 (2.9, 1.14); PPM1D (3.2, 1.04); ASXL2 (2.1, -0.71); DNMT3A (2.1, -0.77); EED (1.8, -0.79); CBL (1.7, -1.25)
Flavopiridol HCl	Cell Cycle	73.4 ( ± 16.7)	GNA13 (2.9, 1.14); PPM1D (3.2, 1.07); EED (1.8, -0.77); CBL (1.7, -1.24)
SNS-032 (BMS-387032)	Cell Cycle	70.8 ( ± 18)	GNA13 (2.9, 1.05); PPM1D (3.2, 0.98); EED (1.8, -0.75); CBL (1.7, -1.17)
Dinaciclib (SCH727965)	Cell Cycle	70.7 ( ± 16.9)	GNA13 (2.9, 1); PPM1D (3.2, 0.98); ASXL2 (2.1, -0.7); DNMT3A (2.1, -0.79); CBL (1.7, -1.12)
INK 128 (MLN0128)	PI3K/Akt/mTOR	70.5 ( ± 7.6)	CBL (1.7, -0.96)
Torin 2	PI3K/Akt/mTOR	70.4 ( ± 7.8)	JAK1 (2, 0.78); CBL (1.7, -0.83)
AT7519	Cell Cycle	70 ( ± 22.5)	GNA13 (2.9, 1.01); JAK1 (2, 0.89); PPM1D (3.2, 0.83); CBL (1.7, -0.81)
PF-04691502	PI3K/Akt/mTOR	69 ( ± 7.2)	CBL (1.7, -0.72)
GSK2126458 (GSK458)	PI3K/Akt/mTOR	67.8 ( ± 8)	NF1 (2.2, -0.7); SUZ12 (2.2, -0.7); CBL (1.7, -0.8)
AZD8055	PI3K/Akt/mTOR	67.7 ( ± 8.9)	JAK1 (2, 0.77)
WYE-125132 (WYE-132)	PI3K/Akt/mTOR	65.6 ( ± 7.7)	H3F3A (2.2, 0.75); MDM4 (2.2, 0.75); CBL (1.7, -0.89)
VS-5584 (SB2343)	PI3K/Akt/mTOR	65.2 ( ± 7.9)	JAK1 (2, 0.77); MPL (1.8, 0.72)
AZD3463	Protein T.K.	65.2 ( ± 17.4)	TCF3 (2, 0.88); JAK1 (2, 0.7)
GDC-0980 (RG7422)	PI3K/Akt/mTOR	64.5 ( ± 8.1)	JAK1 (2, 0.8); MPL (1.8, 0.71); TCF3 (2, 0.69)
AT9283	JAK/STAT	64.3 ( ± 17.4)	DNMT3A (2.1, -0.7); CBL (1.7, -0.72); CALR (1.8, -0.72); CEBPA (1.9, -0.72); CIC (1.8, -0.72); CRLF1 (1.9, -0.72); JAK3 (1.9, -0.72)
AZD2014	PI3K/Akt/mTOR	63.7 ( ± 9.1)	JAK1 (2, 0.87); MPL (1.8, 0.77)
PP242	PI3K/Akt/mTOR	57.6 ( ± 10.3)	TCF3 (2, 0.76); MPL (1.8, 0.75); JAK1 (2, 0.73)
Rigosertib (ON-01910)	Cell Cycle	57.2 ( ± 10.9)	JAK1 (2, 0.89); PHF6 (2.1, 0.71)
PF-477736	Cell Cycle	56.2 ( ± 19.2)	JAK1 (2, 1)
JNK Inhibitor IX	MAPK	55.6 ( ± 11.4)	JAK1 (2, 0.85)
PF-3758309	Cytoskeletal Signaling	55.2 ( ± 18.7)	JAK1 (2, 0.85); CBL (1.7, -0.94)
Milciclib (PHA-848125)	Cell Cycle	55.2 ( ± 9.1)	ASXL1 (2.1, -0.7)
HMN-214	Cell Cycle	51.9 ( ± 13)	JAK1 (2, 0.72)
TAK-901	Cell Cycle	51.6 ( ± 18.3)	JAK1 (2, 0.85)
KX2-391	Angiogenesis	51.2 ( ± 9.4)	JAK1 (2, 0.76); GNA13 (2.9, 0.71); ASXL2 (2.1, -0.74); DNMT3A (2.1, -0.75)
Pelitinib (EKB-569)	Protein T.K.	51 ( ± 13.7)	RUNX1 (2, -0.73)
CHIR-124	Cell Cycle	50.7 ( ± 12.7)	FBXW7 (2, -0.73); FGFR3 (2, -0.73); KDR (2, -0.73); KIT (2, -0.73); PDGFRA (2, -0.73); CBL (1.7, -0.98)
OSI-027	PI3K/Akt/mTOR	50.5 ( ± 8.1)	CRLF2 (1.4, 0.89); DDX3X (1.4, 0.89); KDM6A (1.4, 0.89); MPL (1.8, 0.77); ABL1 (2, 0.71); NOTCH1 (2, 0.71)
TG101209	JAK/STAT	48.5 ( ± 16.8)	TCF3 (2, 0.89)
GDC-0349	PI3K/Akt/mTOR	48.4 ( ± 11.1)	JAK1 (2, 0.72); TCF3 (2, 0.7)
KU-0063794	PI3K/Akt/mTOR	47 ( ± 11.5)	GNA11 (1.8, 0.85); MAP2K2 (1.8, 0.85)
HS-173	PI3K/Akt/mTOR	46.2 ( ± 12.6)	PHF6 (2.1, 0.76); MYCN (27.9, 0.7)
IMD 0354	NF-KB	45.6 ( ± 8.5)	GNA11 (1.8, 0.85); MAP2K2 (1.8, 0.85); PHF6 (2.1, 0.76); MYCN (27.9, 0.7); PPM1D (3.2, 0.72); SMARCA4 (1.9, -0.73); GNA11 (1.8, -0.84); MAP2K2 (1.8, -0.84); APC (2.1, -0.89); CSF1R (2.1, -0.89); EBF1 (2.1, -0.89); FGFR4 (2.1, -0.89); IL7R (2.1, -0.89)
AP26113	Protein T.K.	42.9 ( ± 21.3)	H3F3A (2.2, 0.89); MDM4 (2.2, 0.89); TCF3 (2, 0.78); JAK1 (2, 0.74); ARID1B (1.9, -0.7); CCND3 (1.9, -0.7); DAXX (1.9, -0.7); ESR1 (1.9, -0.7); HIST1H3B (1.9, -0.7)
LDK378	Protein T.K.	42.1 ( ± 28.8)	H3F3A (2.2, 0.95); MDM4 (2.2, 0.95); TCF3 (2, 0.91); GNA11 (1.8, 0.79); MAP2K2 (1.8, 0.79); ARID1B (1.9, -0.82); CCND3 (1.9, -0.82); DAXX (1.9, -0.82); ESR1 (1.9, -0.82); HIST1H3B (1.9, -0.82)
TG101348 (SAR302503)	JAK/STAT	36.3 ( ± 17.3)	TCF3 (2, 0.9); H3F3A (2.2, 0.71); MDM4 (2.2, 0.71); GNA11 (1.8, 0.7); MAP2K2 (1.8, 0.7)
BI 2536	Cell Cycle	35.2 ( ± 16.6)	H3F3A (2.2, 0.82); MDM4 (2.2, 0.82)
FIIN-2	Protein T.K.	35.2 ( ± 17)	JAK1 (2, 0.75)
AZ20	PI3K/Akt/mTOR	35.2 ( ± 11)	JAK1 (2, 0.88)
Pacritinib (SB1518)	JAK/STAT	35.1 ( ± 19.1)	H3F3A (2.2, 0.88); MDM4 (2.2, 0.88); TCF3 (2, 0.78); CBL (1.7, -0.72)
R547	Cell Cycle	34.8 ( ± 13.7)	CBL (1.7, -0.87)
R406 (free base)	Angiogenesis	34.6 ( ± 10.8)	AKT1 (2.2, -0.8); DICER1 (2.2, -0.8); ASXL1 (2.1, -0.83); PIK3CA (2.1, -0.9); RUNX1 (2, -1.05)
AZD7762	Cell Cycle	34.3 ( ± 15.3)	JAK1 (2, 0.89)
Volasertib (BI 6727)	Cell Cycle	33.2 ( ± 10.1)	H3F3A (2.2, 0.79); MDM4 (2.2, 0.79); MPL (1.8, 0.7)

T.K., tyrosine kinase.

Due to the small number of mutations, no significant gene-drug combinations could be identified from a similar correlation of SNVs and cytotoxicity (**Figure 4A**). Thus, we subsequently focused on evaluating the gene-drug pairs with significantly correlations of copy number and cytotoxicity.

To further study these observations, we compared the expected gene-drug associations (according to drug library specifications) with the significantly correlated gene-drug pairs (according to z-scores of copy number-cytotoxicity correlations) identified (**Figure 4B**). A total of 416 gene-drug pairs were expected (**Supplementary Data 1**). Among these, copy number and cytotoxicity of 4 gene-drug pairs showed significant correlations at 0.1 FDR cutoff (true positive pairs): JAK3 with AT9283, JAK1 with CYT387, FGFR1 with tyrphostin (AG1296), and FGFR3 with brivanib (BMS540215) (**Figure 4C**). Only the former 2 drugs demonstrated >33% cytotoxicity and qualified as hits (**Figure 4C**).

In 1274 gene-drug pairs, correlations were not expected but were observed. To identify pairs with the strongest treatment effect and correlation, we ranked them according to the product of their cytotoxicity and z-score (see **Supplementary Data 1**). Among the top- and bottom-ranked pairs, were PI3K and cell cycle agents that correlated with copy number losses of transcriptional regulators CBL and DNMT3A (log_2_ ratios -0.294, and 0.039, respectively), and copy number gains of GNA13 and PPM1D (**Figure 4D**). The drugs most commonly associated with these pairs were PI3K/mTOR inhibitor PIK-75, and CDK inhibitors P276-00, SNS-032, AT7519, flavopiridol and dinaciclib (**Supplementary Figure S4**). Intertumor heterogeneity of copy-number dependent drug sensitivities were observed. For example, MYCN non-amplified tumors showed increased drug sensitivity to these agents with increased PPM1D and GNA13 copy number, while MYCN amplified tumors did not show similar trends (**Supplementary Figure S4**). As this was a substantial number of novel gene-drug associations that were not expected, we next verified them against available data sources and *via in-silico* modelling.

### Significantly Correlated Gene-Drug Pairs Are Verified in Public Datasets

To verify if the significantly correlated gene-drug pairs were consistent with published literature, we compared them against known gene-drug associations in 6 public datasets. In each dataset, median 224 (range:46–965) pairs were identified as having an established association of drug and gene target (**Supplementary Figure S5** and **Supplementary Data 1**). In all, 1305 of 54,758 (2.4%) pairs were identified in at least 1 dataset. Verified and unverified gene-drug pairs did not significantly differ in the distribution of cytotoxicity of the drug involved, copy number of the gene involved, and z-score of the pair across the 6 datasets (**Supplementary Figure S5**).

**Figure 5:**
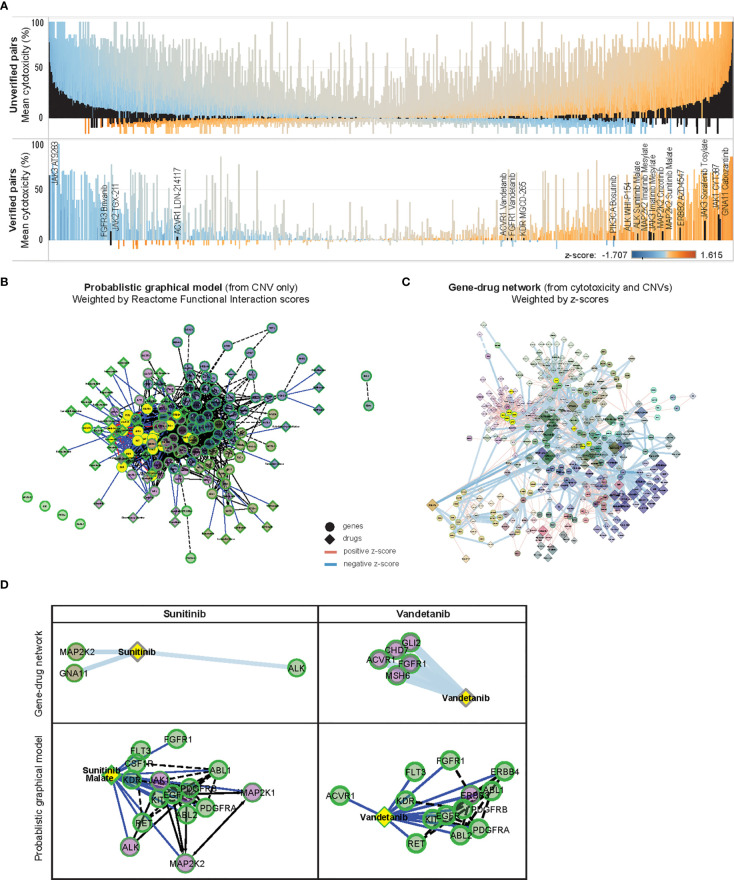
*In-silico* validation of gene-drug pairs. **(A)** Parallel waterfall plots of the mean cytotoxicity of drugs unverified or verified in public datasets, ranked by the product of their z-scores and cytotoxicity; with similar distribution of z-scores and cytotoxicity are seen among both groups. Significantly correlated gene-drug pairs in each group are indicated in black [upper panel: 1259 of 55,543 unverified gene-drug pairs, lower panel: 19 of 1305 verified gene-drug pairs (total 54,758 gene-drug pairs)], color scale indicates z-scores. **(B)** ReactomeFIViz Probabilistic Graphical Model (PGM) constructed from weighted factors derived from CNV of 131 OCCRA genes in 13 neuroblastoma cases, annotated with 46 associated Cancer Targetome drugs and clustered according to Reactome Functional Interaction (FI) scores; solid links indicate known associations and dotted links indicate predicted association, nodes are colored according to Reactome FI clusters **(C)** z-score weighted network of 131 OCCRA genes and 418 drugs, clustered similarly according to Reactome FI scores; width of links indicates z-score and size of diamonds indicates cytotoxicity. **(D)** Association of sunitinib and vandetanib with first-degree neighbor genes, determined by z-scores, and as predicted by PGM. Both sets of gene-drug pairs are correspondingly indicated in yellow in **(B, C)**.

Among the 1278 significantly correlated gene-drug pairs, 19 pairs (1.5%) were verified in at least 1 data set (**Supplementary Table 4**) (**Figure 5A**), though only 1 pair (5.3%) qualified as a hit with >33% mean cytotoxicity [JAK3 with AT9283 (mean cytotoxicity 64.3 ± 16.8%)]. Among the remaining unverified 1259 gene-drug pairs, a higher percentage of pairs [12.9% (n=162)] were predicted to be hits with >33% mean cytotoxicity. Among these pairs, cytotoxicity was positively associated with copy number of GNA13, PPM1D, JAK1, and MAP2K2, and inversely associated with copy number of CBL, EED, DNMT3A and ASXL2. The drugs most commonly associated with these pairs were PI3K inhibitors PIK-75 and P276-00, and CDK inhibitors SNS-032, flavopiridol and dinaciclib (**Supplementary Tables 5**, **6**) – similar to the commonest genes and drugs identified from the above comparison with specified targets of the drug library. The high incidence of hits among unverified top gene-drug pairs further suggested that important gene-drug relationships may exist among this group, and warranted further exploration.

### Identified Gene-Drug Associations Are Replicated in Probabilistic *In-Silico* Model and Reveal Gene-Drug Subnetworks Centered Around PI3K and JAK-STAT Signaling

Since published data on this majority of the significantly correlated gene-drug relationships was lacking, we performed an *in-silico* prediction of how the CNV profile of our neuroblastoma tumors would be expected to influence drug response, and compared that with the corresponding z-score-based relationships. We constructed a Probabilistic Graphical Model (PGM) network by weighting our CNV data with Reactome pathway-based interactions to infer variations in inter-gene relationships (17, 19), then overlaying networks with associated Cancer Targetome drugs (20) (**Figure 5B**). A corresponding network was constructed from the 1278 significantly correlated gene-drug pairs and weighted according to z-scores (**Figure 5C**). Both networks were clustered according to Reactome Functional Interaction (FI) scores, and sub-networks of commonly represented drugs were examined.

Since the curated source included only FDA-approved cancer drugs, as expected only verified gene-drug pairs were found in the PGM network (**Supplementary Data 1**). Among them, gene-drug pairs involving sunitinib and vandetanib were modeled by the PGM network – both receptor tyrosine kinase inhibitors with known effects against neuroblastoma (29–31). Examples in **Figure 5D** show how the PGM, derived only from CNV data, predicted associations between sunitinib malate and ALK and MAP2K2, and between vandetanib and ACVR1 and FGFR1 – which were similarly demonstrated in the z-score-weighted network. The similarities observed between the CNV-based PGM predictions and the z-score-weighted network supported the integrity of the gene-drug associations identified by our experimental system.

Since FI-based clustering might reveal associations between drugs and signaling pathways with potential functional significance, we isolated FI-clustered sub-networks of gene-drug pairs and annotated them for enriched Reactome pathways and Gene Ontology features (**Supplementary Data 2**). Interestingly, 2 sub-networks coincided with the unverified significantly associated gene-drug pairs that were earlier identified (**Supplementary Figures S6A, B**). In the former, copy number of ALK, DNMT3A and ASXL2 was inversely associated with efficacy of multiple kinase inhibitors like saracatanib (Module 1). In the latter, copy number of 17q genes CBL, PPM1D and GNA13 were positively associated with cytoxicity to dinacilib, flavopiridol and ponatinib (Module 8). Other sub-networks shared common involvement of the PI3K-mTOR (Modules 3, 6, 11), JAK-STAT (Modules 0, 3, 5, 6) pathways. Notably the z-score network also illustrated the lack of inhibitors associating with MYCN despite its substantial degree of copy number amplification.

## Discussion

CNVs are more prevalent in mutationally quiet embryonal cancers like neuroblastoma and may reflect fundamental oncogenic dependencies that can be potentially exploited for cancer therapeutics – particularly since this phenomenon has already been utilized to target oncogene amplifications in adult cancers. In this study we hypothesized that CNVs may modulate gene dosage and correspondingly, drug response to respective targeted inhibitors. Thus, we integrated diagnostic-grade genomic profiling with phenotypic readouts of personalized preclinical tumor models to identify copy number-dependent therapeutic susceptibilities in patients with neuroblastoma. We used correlational coefficients to quantify the strength of association between tumor copy number and cytotoxicity of PDCs in corresponding gene-drug pairs, and validated this against public data and *in silico*. The findings defined a prioritized set of targetable copy number-altered genes and their ideal corresponding inhibitors for copy-number dependent drug targeting. To provide a statistically robust context to apply these in clinical practice, we generated prediction models of the expected therapeutic response for given degrees of copy number change for each gene-drug pair. These potentially form the basic elements of a decision-making framework that could be used to inform personalized therapeutic decisions by predicting potential drug hits based on CNVs.

While the identified SNVs were known genomic aberrations in neuroblastoma (32–34), they were mostly unactionable. This underscored the limitations of targeted mutational profiling for identifying personalized treatment options for neuroblastoma and other mutationally quiet embryonal cancers (2, 35). Among the commonest variants observed in our study cohort, TP53 mutations are still largely considered untargetable (36, 37). Similarly, options for TSC2-deficient tumors are limited and largely aimed at downstream mTOR pathway targets (38). Frequency of these SNVs differed from Western series where ALK, TERT, PTPN11 and NRAS mutations are more frequent, likely reflecting known inter-ethnic differences in mutational incidence. For example, *de novo* somatic TP53 mutations are uncommon in Western series of neuroblastoma (33), but polymorphisms such as R72P R337H are more frequent in non-Caucasian populations and are associated with varying incidence of neuroblastoma (39, 40). Similarly, recurrent mutations in TSC, ARID1A and ARID1B have been reported at a lower incidence (1.7%, 11% and 6.9%, respectively) in Western populations (41–43).

Conversely, CNVs were more prevalent and importantly, demonstrated significant and biologically consistent correlations with cytotoxicity to corresponding targeted inhibitors. PPM1D, GNA13, and STAT family genes – all on 17q and implicated in up to 70% of neuroblastomas – showed the strongest correlations of copy number and cytotoxicity to targeted inhibitors (44–50). This could be due to SCAs, which are frequent and associated with worse outcome in neuroblastoma (4, 51), and regulate sensitivity to drug response in various cancers (52–54). Hence, with the known prevalence of SCAs in neuroblastoma, these associations between CNVs of key transcription factors and sensitivity to corresponding inhibitors suggests that differential copy number may be a potential quantitative predictor of drug response in neuroblastoma.

Significant associations between PI3K/Akt/mTOR pathway genes and their corresponding inhibitors were most frequently observed, accounting for half of the top 20 drug hits. PI3K/Akt/mTOR pathway upregulation, with cytoplasmic phosphorylated Akt, occurs in 62% of neuroblastomas and is a promising therapeutic target (55, 56), due to the central role played by PI3K activation in stabilizing MYCN, rendering neuroblastoma tumors sensitive to PI3K inhibition *via* downregulation of MYCN and corresponding tumor proliferation (27, 57). BGT226 was the most potent PI3K inhibitor identified (83.6% cytotoxicity), but its effects on neuroblastoma remain under-studied, and development has been discontinued following phase I/II trials in advanced adult solid tumors for lack of efficacy, inconsistent target inhibition and dose-limiting toxicities (58, 59). PI3Kα inhibitor PIK-75 (78.9% cytotoxicity) stimulates anti-neuroblastoma activity by destabilizing MYCN and sensitizing tumor cells to anthracyclines (27, 60), and indeed increased cytotoxicity with PIK-75 was associated with PPM1D and GNA13 gain, and DNMT3A, CBL, EED and ASXL2 loss. Next most potent was PI-103 (68.3% cytotoxicity), which sensitizes neuroblastoma cells to TRAIL-induced apoptosis (61), and primes them to cytotoxicity from anthracyclines (62).

Cyclin dependent kinases (CDKs) critically affect cell growth, proliferation and transcriptional regulation in neuroblastoma (63). Pan-CDK inhibitors flavopiridol and dinaciclib – both among our top hits – are in now early phase trials for neuroblastoma and advanced solid tumors (23–25). AT7519, a CDK2 inhibitor (70.0% cytotoxicity), has shown excellent preclinical anti-tumor responses against MYCN-amplified neuroblastoma (64), and is currently in phase II clinical trials for adults (NCT00390117) and children (NCT01627054). CDK4/6 expression is upregulated in 30% of neuroblastomas resulting in E2F overexpression and S and G2/M phase progression (32), and is another attractive therapeutic target. Palbociclib, a CDK4/6 inhibitor, is currently approved for advanced breast cancer and in phase III clinical trials (65); P276-00, a selective CDK4 inhibitor (79.7% cytotoxicity), has shown anti-proliferative effects against other cancers (66), but too has not been assessed in neuroblastoma.

Mixed patterns of gene-drug associations were observed in JAK/STAT pathway genes, MAPK and ALK. JAK3 showed significant correlation with its corresponding inhibitor AT9283 and qualified as a drug hit, but not with other JAK inhibitors like ruxolitinib. While RAS-MAPK mutations are frequent in relapsed neuroblastoma, response to MAPK inhibitors (67), and BET and MEK inhibitors (68), have been inconsistent *in vivo*, possibly due to more complex relationships between receptor tyrosine kinase inhibitors and MAPK family genes. Similarly, we also did not find significant correlation between ALK copy number and cytotoxicity with crizotinib, but instead observed associations between ALK copy number and response to other kinase inhibitors. Despite the well-established prognostic significance of ALK mutations in neuroblastoma (69), a phase I/II clinical trial for pediatric patients with relapsed and refractory solid tumors yielded complete responses in only 1 out of 11 patients with known ALK mutations (NCT00939770) (70). Results are awaited from more recent trials for patients with ALK mutations investigating loratinib, a third generation ALK inhibitor (NCT03107988), ceritinib and ribociclib (NCT02780128), and ensartinib (NCT03155620). These findings may indicate variations in gene dosage-related sensitivity to response within drug classes.

This study was limited by the small number of cases, which may limit the robustness of the gene-drug correlations identified, and the inherent inability to test every drug hit in individual patients. Few of our shortlisted gene-drug associations could ultimately be verified against available datasets, limiting the confidence of our observations. However, our low incidence of verified gene-drug pairs is mirrored in the other drug repurposing studies mapping gene-drug associations in human disease (71). In future, corroboration of our findings in larger cohorts, and further functional validation will be required to verify the true clinical efficacy of our identified gene-drug pairs.

Associations between gene copy number and drug response could have been influenced by prior systemic treatment, or activation of alternate signaling pathways, though these are frequently encountered in relapse clinical cases where personalized therapeutic solutions are most often pursued. For example, the higher frequency of TSC2 mutants in our series could have contributed to the high rates of sensitivity of the PDCs to PI3K/mTOR-targeted agents (72). The frequently observed massive amplifications of MYCN may have also affected responses to cell cycle agents. While MYCN is similarly recognized as an undruggable target due to the lack of appropriate drug binding surfaces on its DNA binding domain, BH3 mimetics and pan-BCl-2 inhibitors can exploit the Bcl-2 over-expression frequently accompanying MYCN amplification (73–75). Alternative treatment strategies have also been suggested that capitalize on its interactions with ALK and AURKA (76, 77).

Treatment susceptibility prediction *via* genomic profiling and phenotypic assays also have their respective limitations: genomic profiling makes fundamental assumptions on correlations between genotype and phenotypic response to targeted inhibitors, while *in vitro* models may not fully recapitulate the original tumor drug-response phenotype. Also, while OCCRA offers a more efficient and focused means to survey for meaningful genomic aberrations, other novel gene targets may be missed. Future studies could complement mutational profiling with gene expression results. Also, in future, drug sensitivity could be evaluated more accurately with three-dimensional organoid models, which may better represent the true tumor microenvironment and expression profile that may influence response to test agents (41, 78).

In conclusion, integration of individualized genomic profiling and drug screening of patient-derived models of neuroblastoma showed that susceptibility to PI3K and cell cycle agents was significantly associated with copy number gains of PI3K and STAT family genes, particularly on 17q. This approach can facilitate CNV-based quantitative treatment response prediction in other mutationally quiet embryonal cancers like neuroblastoma.

## Data Availability Statement

The original contributions presented in the study are included in the article/**Supplementary Material**. Further inquiries can be directed to the corresponding author.

## Ethics Statement

The studies involving human participants were reviewed and approved by SingHealth Duke NUS Central Institutional Review Board (number 2014/2079). Written informed consent to participate in this study was provided by the participants’ legal guardian/next of kin.

## Author Contributions

RW: Conceptualization, Data curation, Funding acquisition, Investigation, Visualization, and Writing - original draft. MRW: Conceptualization, Data curation, Investigation, and Methodology. CK: Data curation, Investigation, and Software. SES: Formal analysis and Software. MKW: Data curation and Investigation. ST: Data curation and Project administration. KM: Data curation and Resources. KC: Data curation and Resources. MT: Investigation and Methodology. GP: Methodology and Resources. ZC: Writing - review & editing. PI: Writing - review & editing. ET: Writing - review & editing. SYS: Writing - review & editing. NGI: Supervision. QF: Conceptualization, Formal analysis, Validation. AL: Conceptualization, Funding acquisition, Formal analysis, Validation, Visualization, Supervision. All authors contributed to the article and approved the submitted version.

## Funding

This work was supported by the VIVA Foundation for Children with Cancer (VIVA-KKH Paediatric Brain and Solid Tumour Programme), Duke NUS Medical School (grant AM-ETHOS01-19-A19); SingHealth Duke NUS Surgery Academic Clinical Programme (grant GRSG14AL). The funders had no role in study design, data collection and analysis, decision to publish, or preparation of the manuscript.

## Conflict of Interest

The authors declare that the research was conducted in the absence of any commercial or financial relationships that could be construed as a potential conflict of interest.

## Publisher’s Note

All claims expressed in this article are solely those of the authors and do not necessarily represent those of their affiliated organizations, or those of the publisher, the editors and the reviewers. Any product that may be evaluated in this article, or claim that may be made by its manufacturer, is not guaranteed or endorsed by the publisher.

## Supplementary Material

Supplementary Figure S1 Unsupervised hierarchical clustering of normalized cytotoxicity of 418 drugs against 13 PDCs. Rows represent cytotoxicity (blue-red scale) and columns represent cases. INPC, International Neuroblastoma Pathology Classification; FH, favorable histology; UH, unfavorable histology; INSS, International Neuroblastoma Staging System; MOA, mechanism of action.

Supplementary Figure S2 Unsupervised hierarchical clustering of copy number profiles of 13 neuroblastoma tumor samples. Rows represent gains (red) and losses (blue) of each gene and columns represent cases. Four tumors with MYCN amplification clustered together, of which 3 cases had concurrent copy number losses in 1p genes MPL, CSF3R, ARID1A, and ID3, and 19p genes MAP2K2, GNA11, CIC, CALR, JAK3, CRLF1, and SMARCA4 (NBL170120, NBL061218, NBL031219B) (solid box) and 1 case had multiple losses involving chromosome 9, 10, 15 and 16p genes (NBL061218) (dotted box).

Supplementary Figure S3 Heatmap of z-scores of 418 drugs (columns) and 136 genes (rows) using unsupervised Euclidean clustering. Blue-red color scale highlights gene-drug pairs with 2.5% top- and bottom-most z-scores. Significant clusters of genes and drugs are annotated, and corresponding OCCRA panels for respective genes are indicated in grey and black on the right panel.

Supplementary Figure S4 Scatterplots of cytotoxicity against copy number for top 6 false negative gene-drug pairs, ranked left to right by product of cytotoxicity (%) and z-score. Clinical-pathological characteristics of individual PDC lines are reflected in legend. INPC, International Neuroblastoma Pathology Classification; UH, unfavorable histology; FH, favorable histology.

Supplementary Figure S5 Boxplots showing distribution (median, quartiles, range) of: (top panel) copy number of matched genes, (middle panel) cytotoxicity of matched drugs, and (lower panel) z-scores of matched gene-drug pairs, verified in 6 datasets of gene-drug associations.

Supplementary Figure S6
**(A–M)** z-score-weighted sub-networks determined by FI-based clustering; corresponding top 5 signaling pathways most enriched in the sub-networks are listed. Source of pathways are annotated as follows: (C) CellMap, (R) Reactome, (K) KEGG, (N) NCI PID, (P) Panther, (B) BioCarta.

Supplementary Data Sheet 1 Genomic profiling, drug screening and Pearson correlation data.

Supplementary Data Sheet 2 Reactome and GO annotations

Supplementary Data Sheet 3 Neuroblastoma PDC and tumor STR profiles

Supplementary Data Sheet 4 Neuroblastoma PDC growth curves

## Abbreviations

SNV, single nucleotide variant; CNV, copy number variation; SCA, segmental chromosomal aberration; OCCRA, Oncomine Childhood Cancer Research Assay; PDC, patient-derived cell culture; FDR, false discovery rate; PGM, Probabilistic Graphical Model; CDK, cyclin dependent kinase.
