# Supplementary figures and images for: Integrated Genomic Profiling and Drug Screening of Patient-Derived Cultures Identifies Individualized Copy Number-Dependent Susceptibilities Involving PI3K Pathway and 17q Genes in Neuroblastoma
Source: Front Oncol. 2021 Oct 14;11:709525. doi: 10.3389/fonc.2021.709525 (PMC8551924; doi:10.3389/fonc.2021.709525)

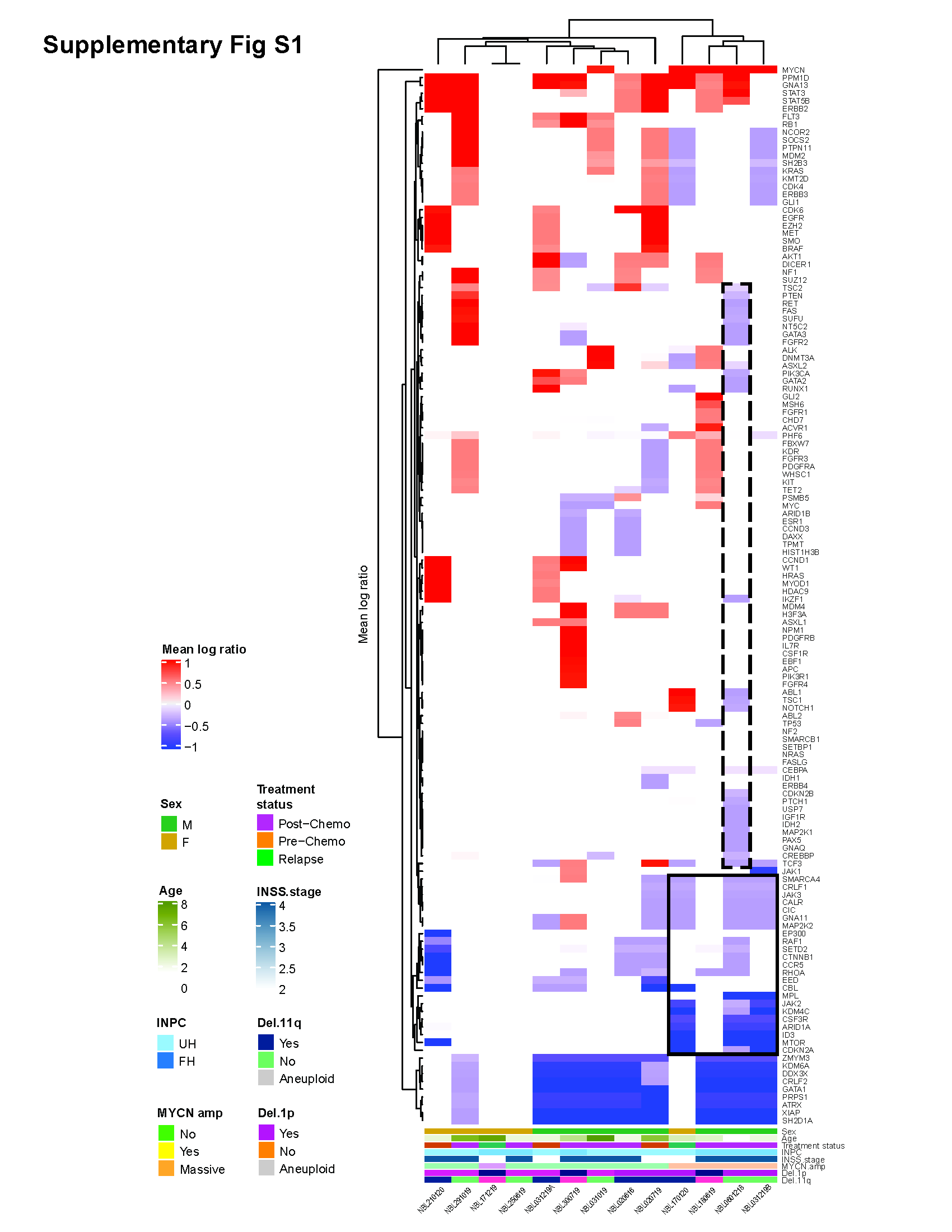

Supplement: Supplementary Figure S1 — Unsupervised hierarchical clustering of normalized cytotoxicity of 418 drugs against 13 PDCs. Rows represent cytotoxicity (blue-red scale) and columns represent cases. INPC, International Neuroblastoma Pathology Classification; FH, favorable histology; UH, unfavorable histology; INSS, International Neuroblastoma Staging System; MOA, mechanism of action. [file Image_1.tif]

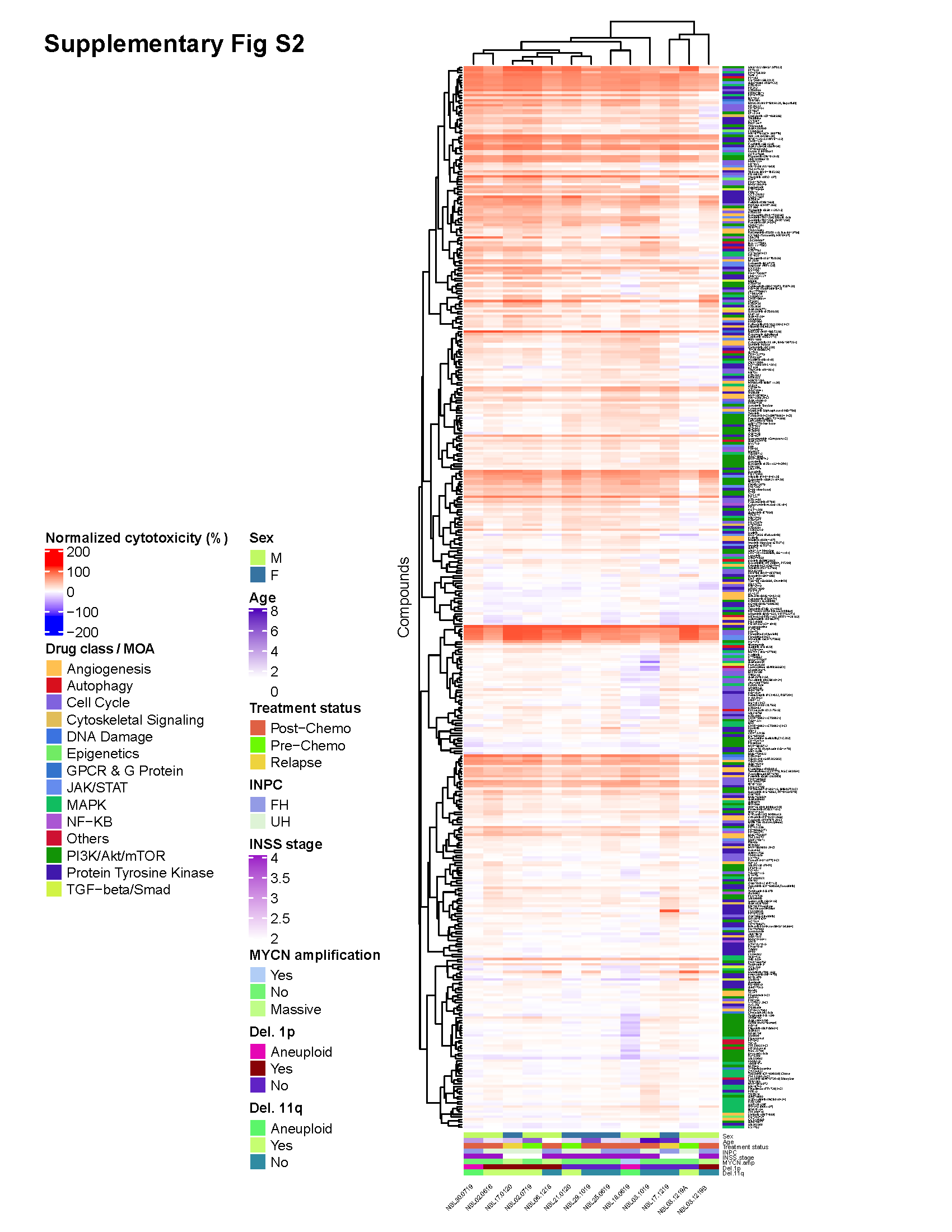

Supplement: Supplementary Figure S2 — Unsupervised hierarchical clustering of copy number profiles of 13 neuroblastoma tumor samples. Rows represent gains (red) and losses (blue) of each gene and columns represent cases. Four tumors with MYCN amplification clustered together, of which 3 cases had concurrent copy number losses in 1p genes MPL, CSF3R, ARID1A, and ID3, and 19p genes MAP2K2, GNA11, CIC, CALR, JAK3, CRLF1, and SMARCA4 (NBL170120, NBL061218, NBL031219B) (solid box) and 1 case had multiple losses involving chromosome 9, 10, 15 and 16p genes (NBL061218) (dotted box). [file Image_2.tif]

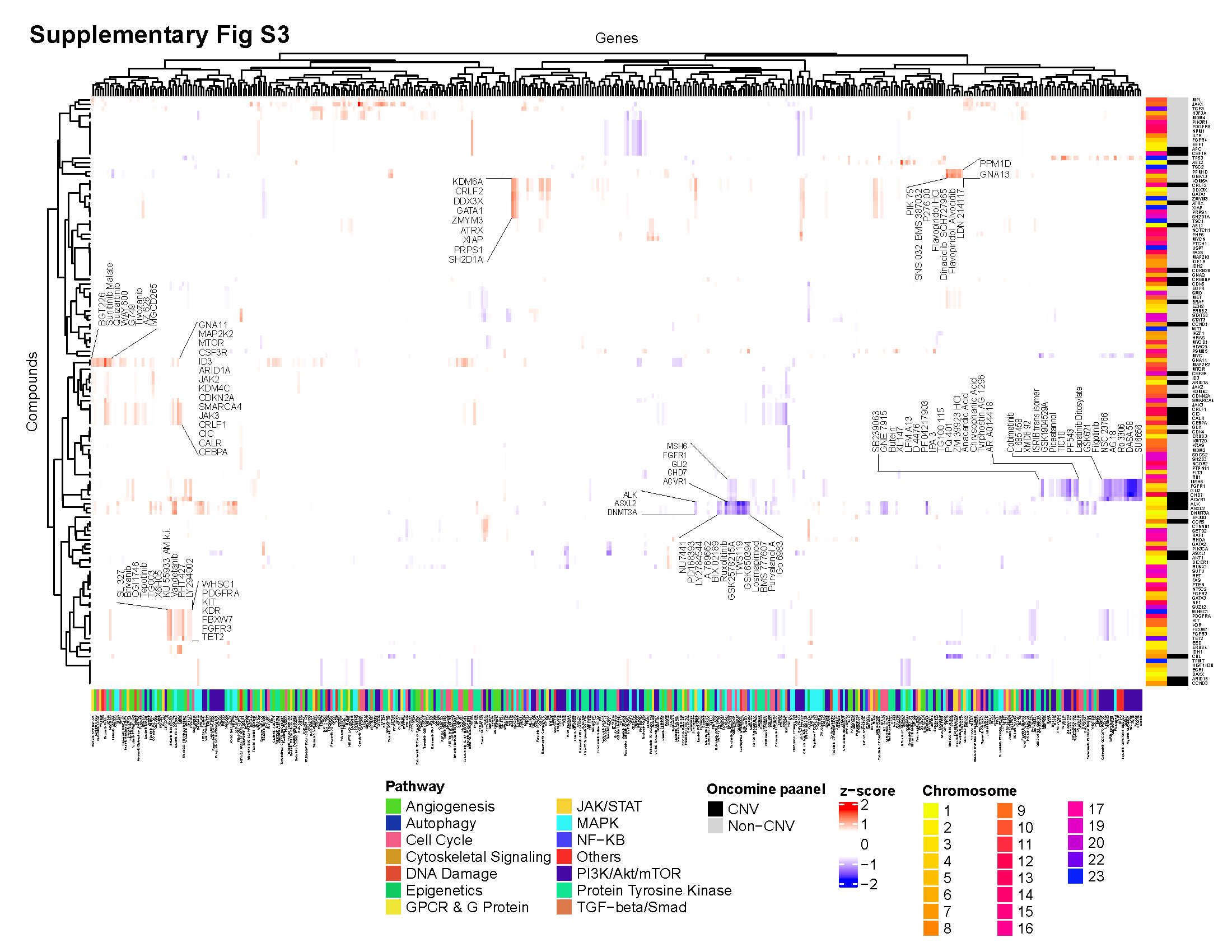

Supplement: Supplementary Figure S3 — Heatmap of z-scores of 418 drugs (columns) and 136 genes (rows) using unsupervised Euclidean clustering. Blue-red color scale highlights gene-drug pairs with 2.5% top- and bottom-most z-scores. Significant clusters of genes and drugs are annotated, and corresponding OCCRA panels for respective genes are indicated in grey and black on the right panel. [file Image_3.tif]

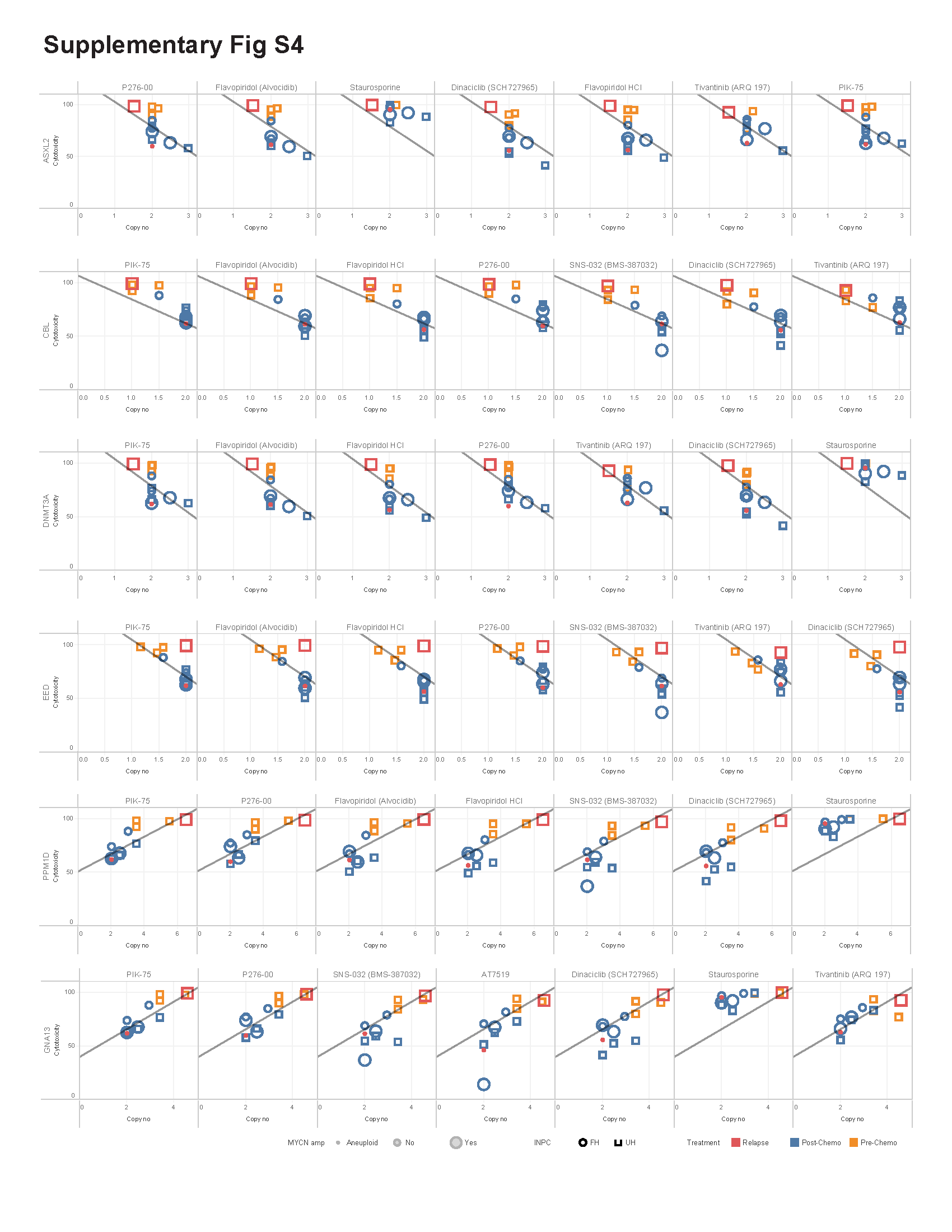

Supplement: Supplementary Figure S4 — Scatterplots of cytotoxicity against copy number for top 6 false negative gene-drug pairs, ranked left to right by product of cytotoxicity (%) and z-score. Clinical-pathological characteristics of individual PDC lines are reflected in legend. INPC, International Neuroblastoma Pathology Classification; UH, unfavorable histology; FH, favorable histology. [file Image_4.tif]

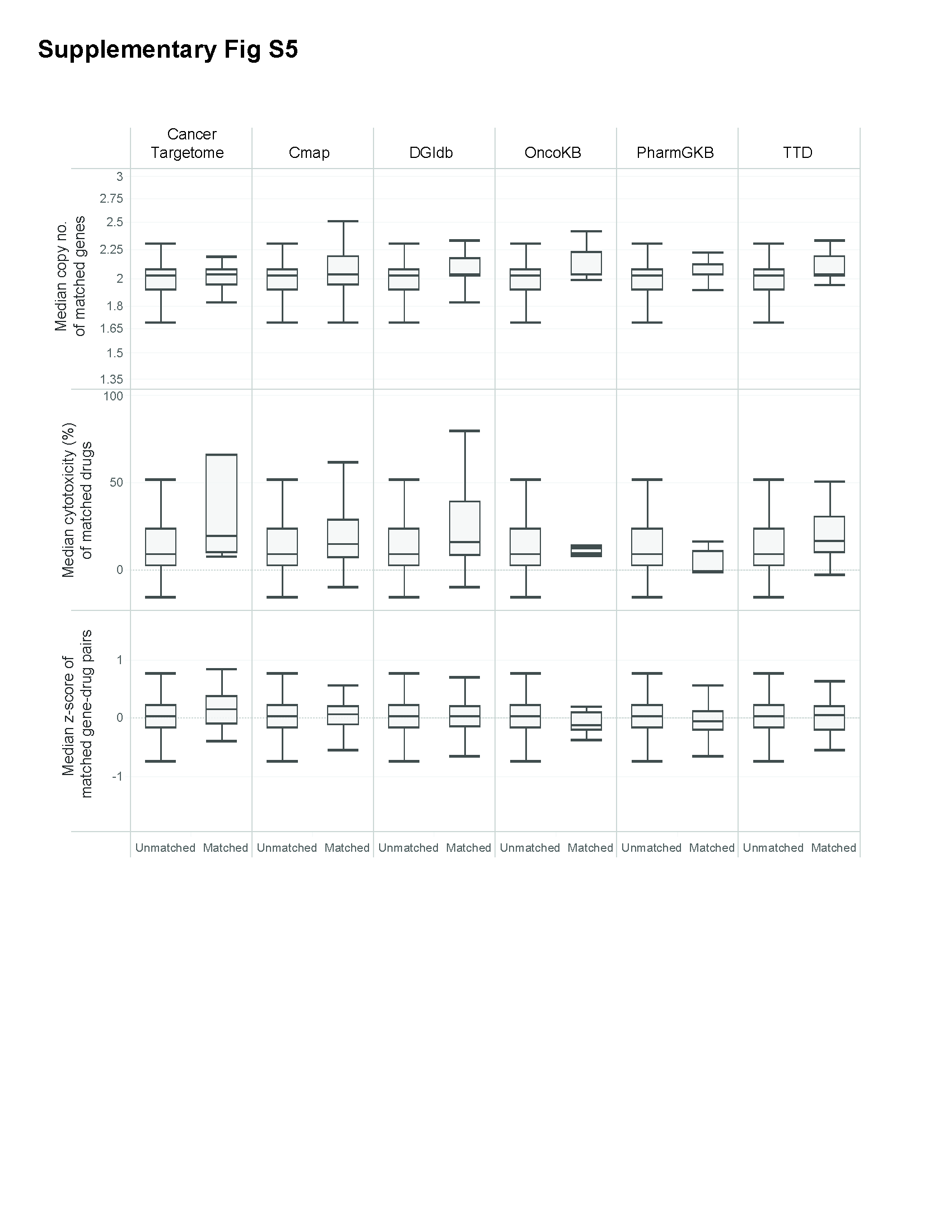

Supplement: Supplementary Figure S5 — Boxplots showing distribution (median, quartiles, range) of: (top panel) copy number of matched genes, (middle panel) cytotoxicity of matched drugs, and (lower panel) z-scores of matched gene-drug pairs, verified in 6 datasets of gene-drug associations. [file Image_5.tif]

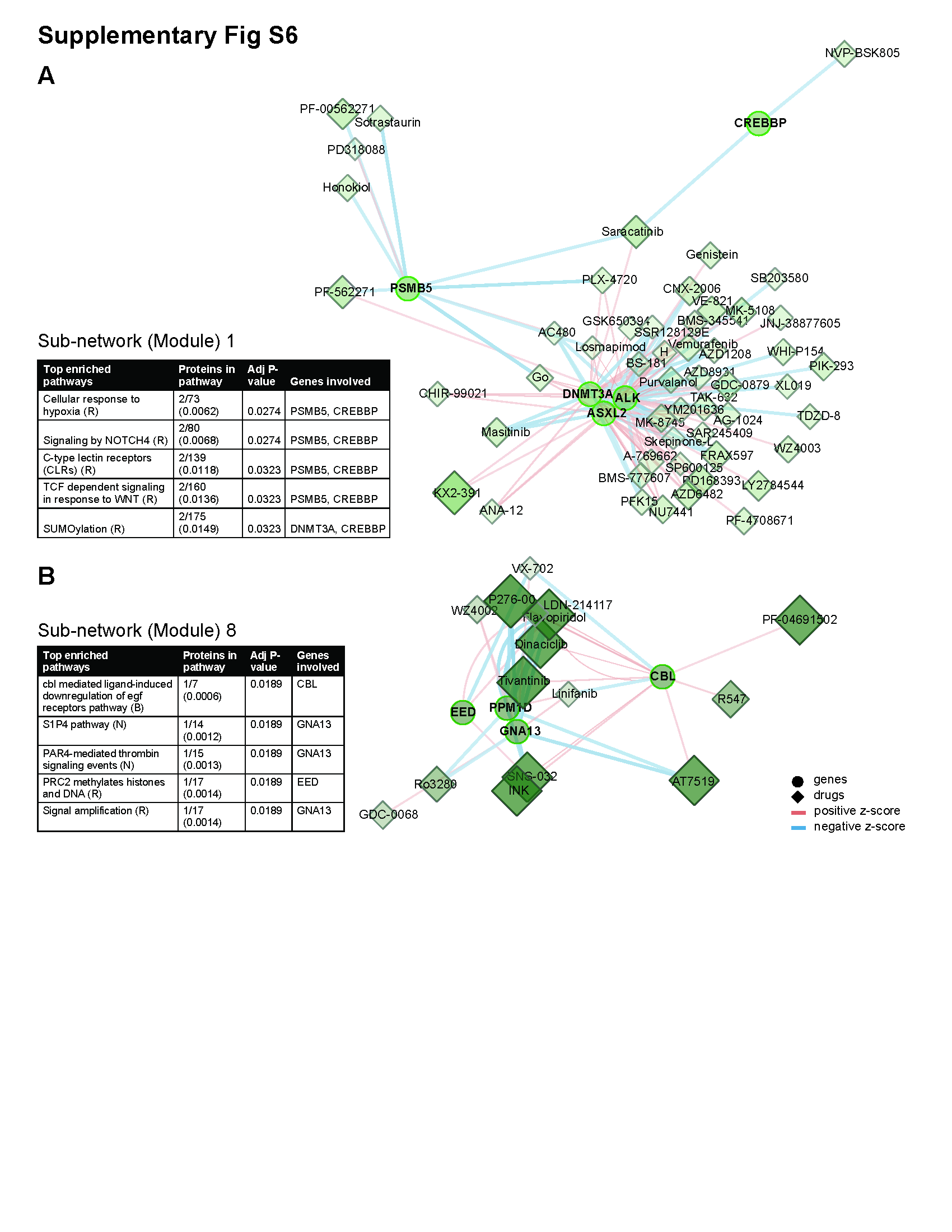

Supplement: Supplementary Figure S6 — (A–M) z-score-weighted sub-networks determined by FI-based clustering; corresponding top 5 signaling pathways most enriched in the sub-networks are listed. Source of pathways are annotated as follows: (C) CellMap, (R) Reactome, (K) KEGG, (N) NCI PID, (P) Panther, (B) BioCarta. [file Image_6.tiff]

# NBL02-0616

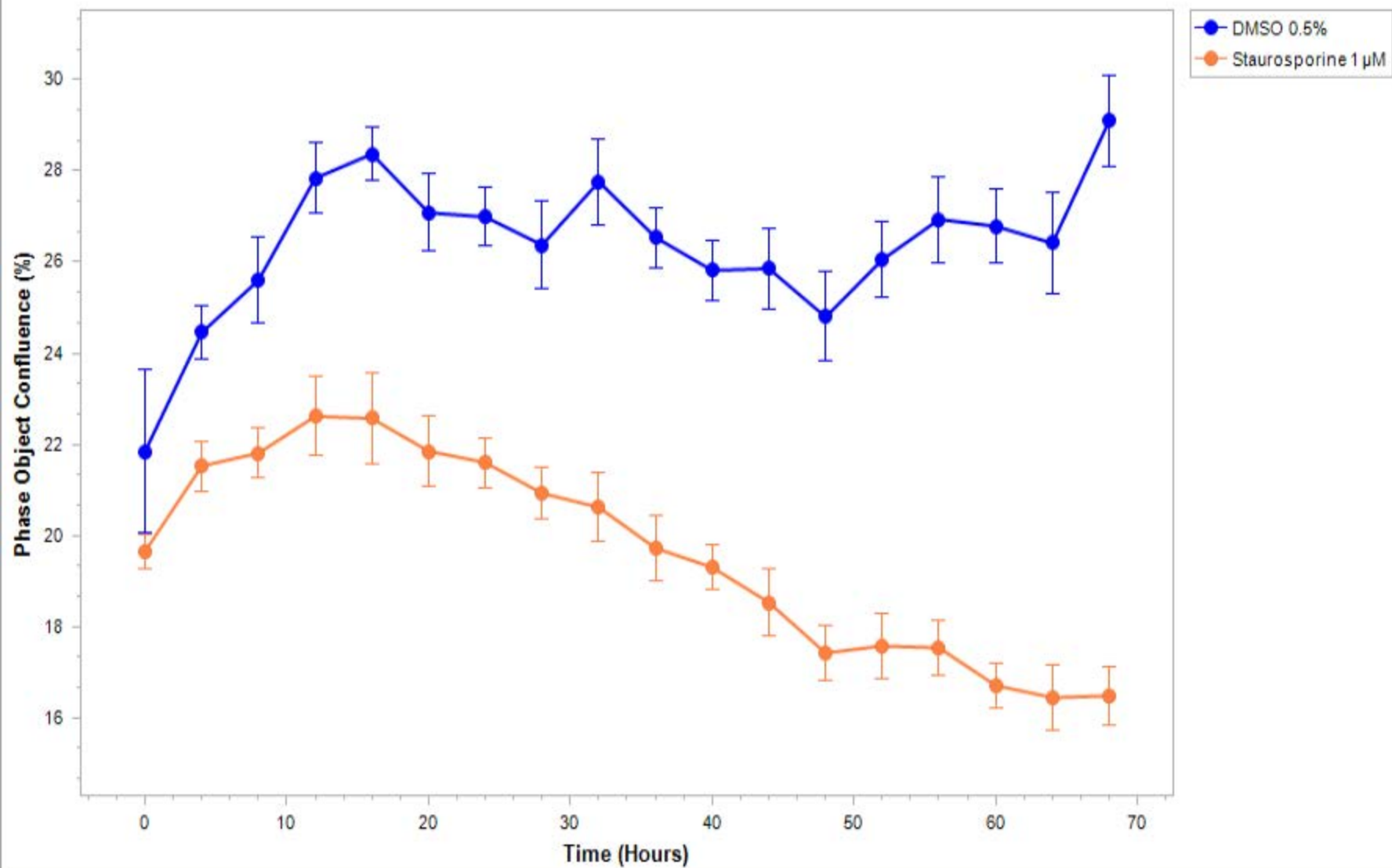

# NBL06-1218

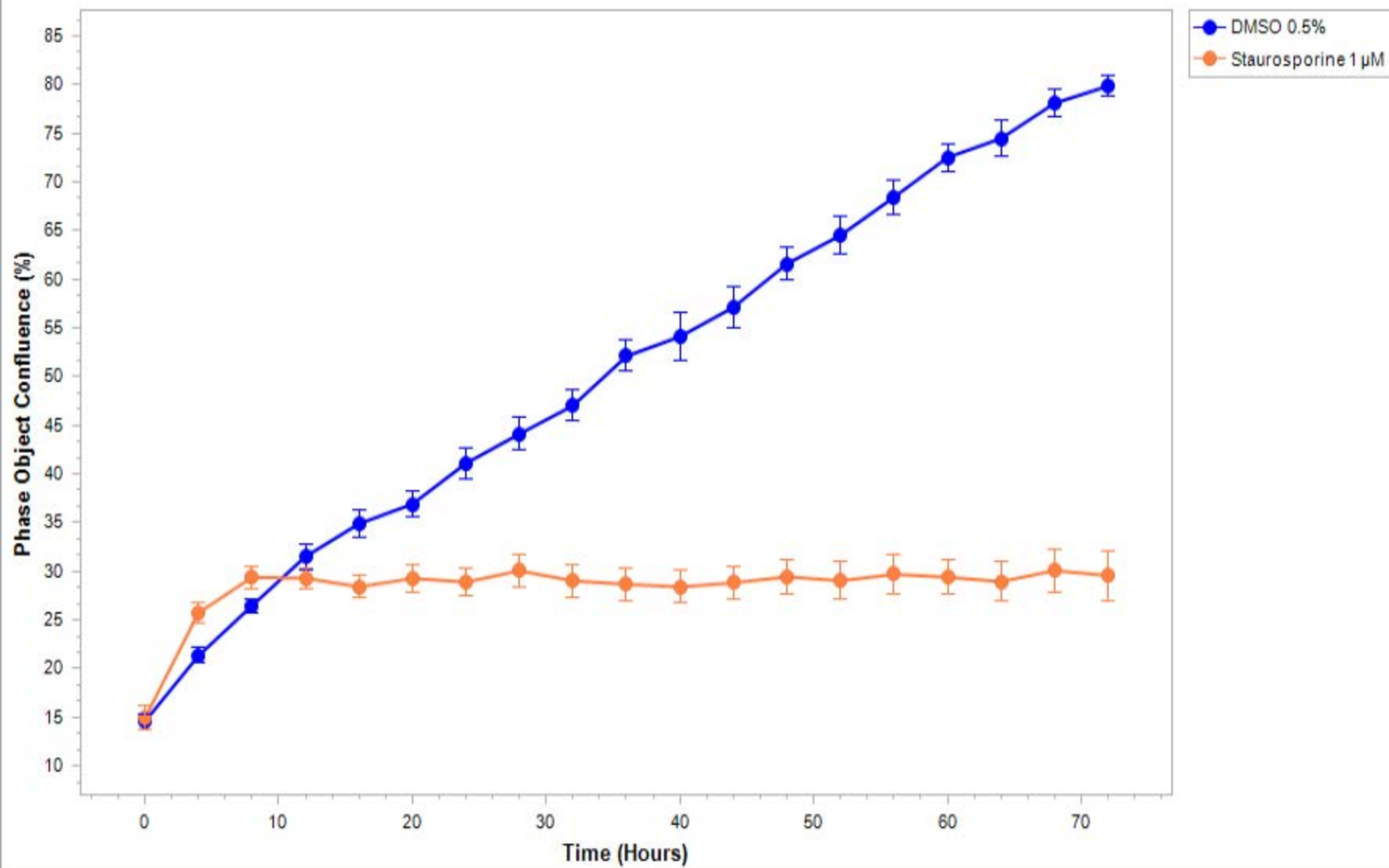

# NBL18-0619

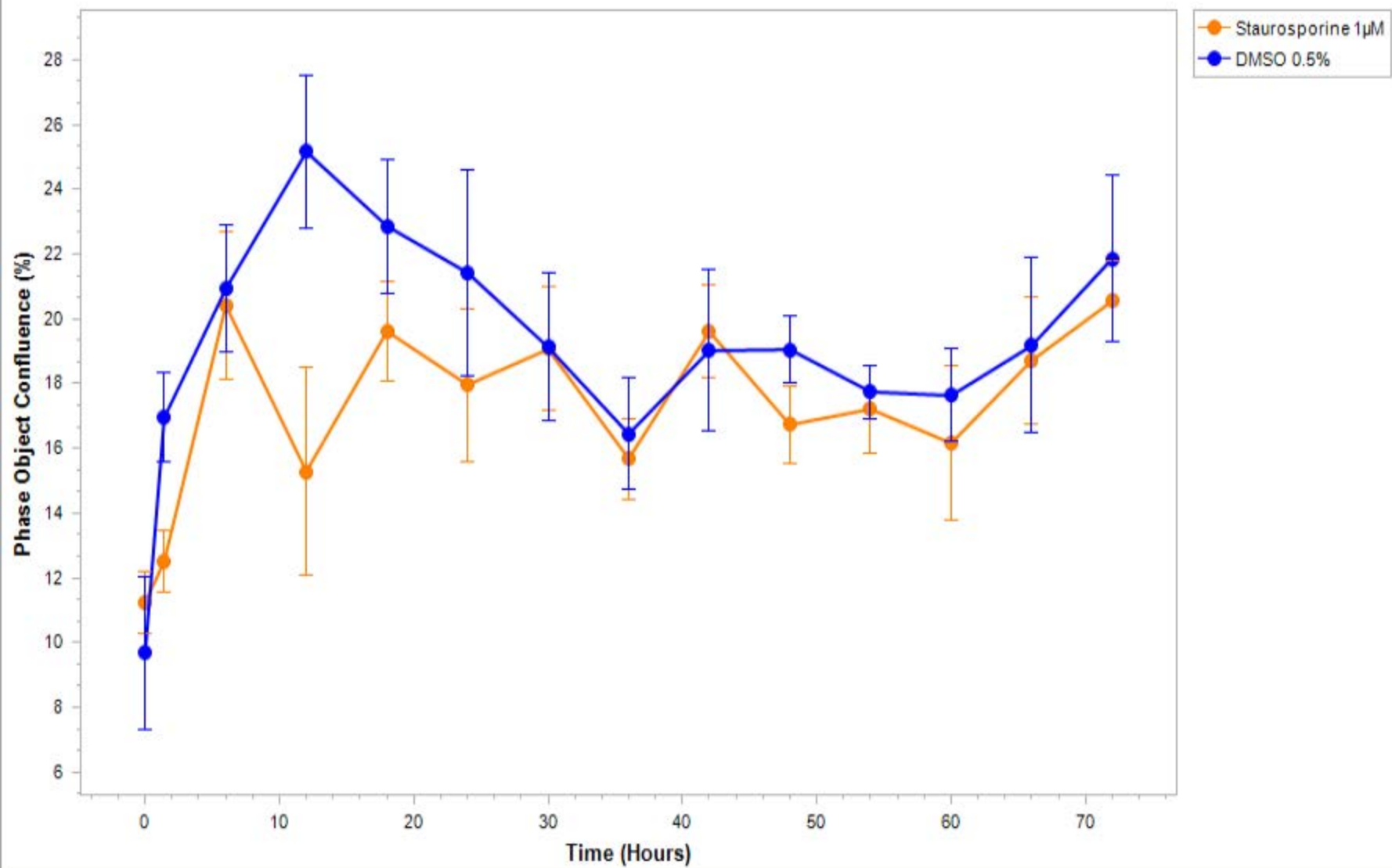

# NBL25-0619

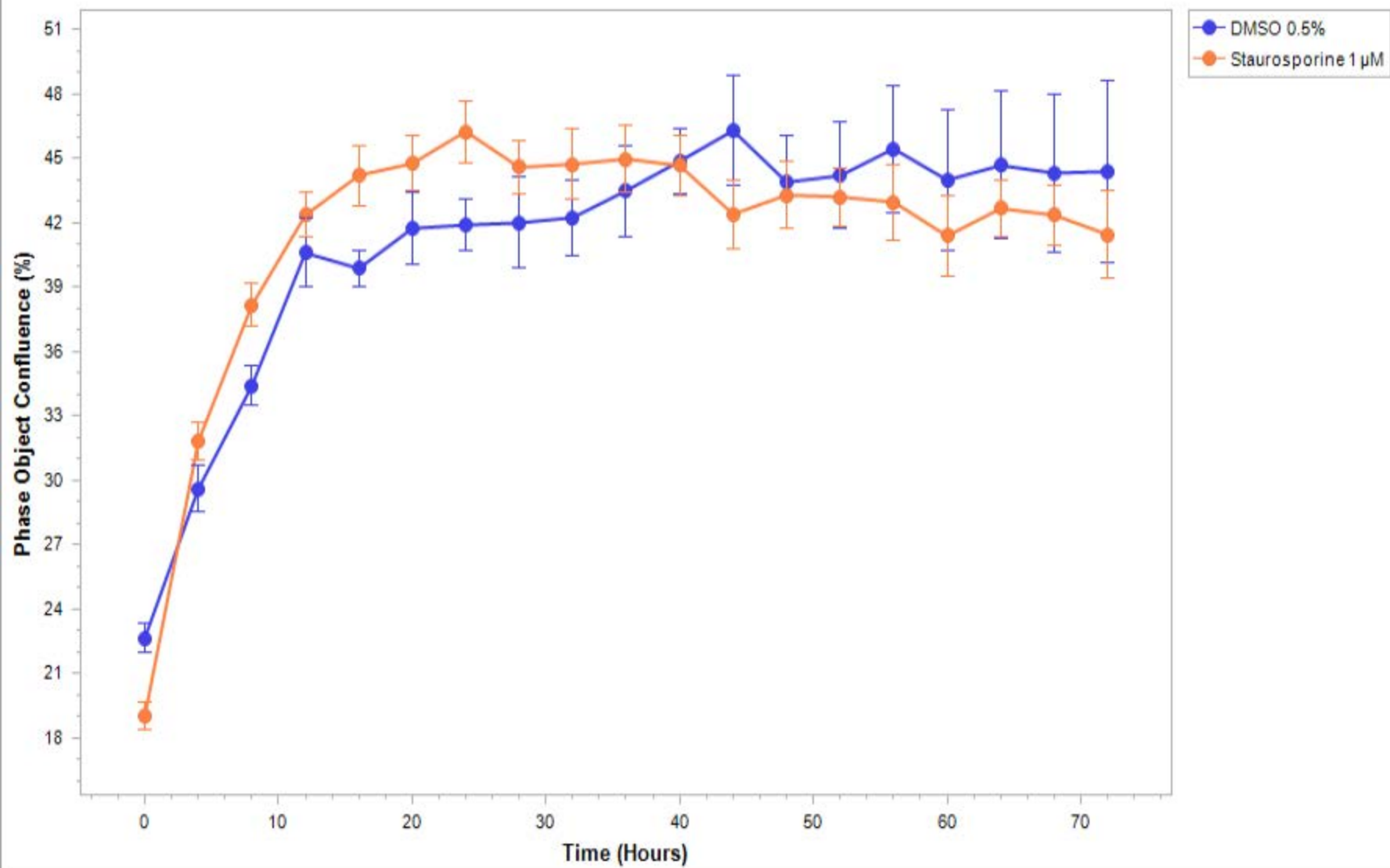

# NBL02-0719

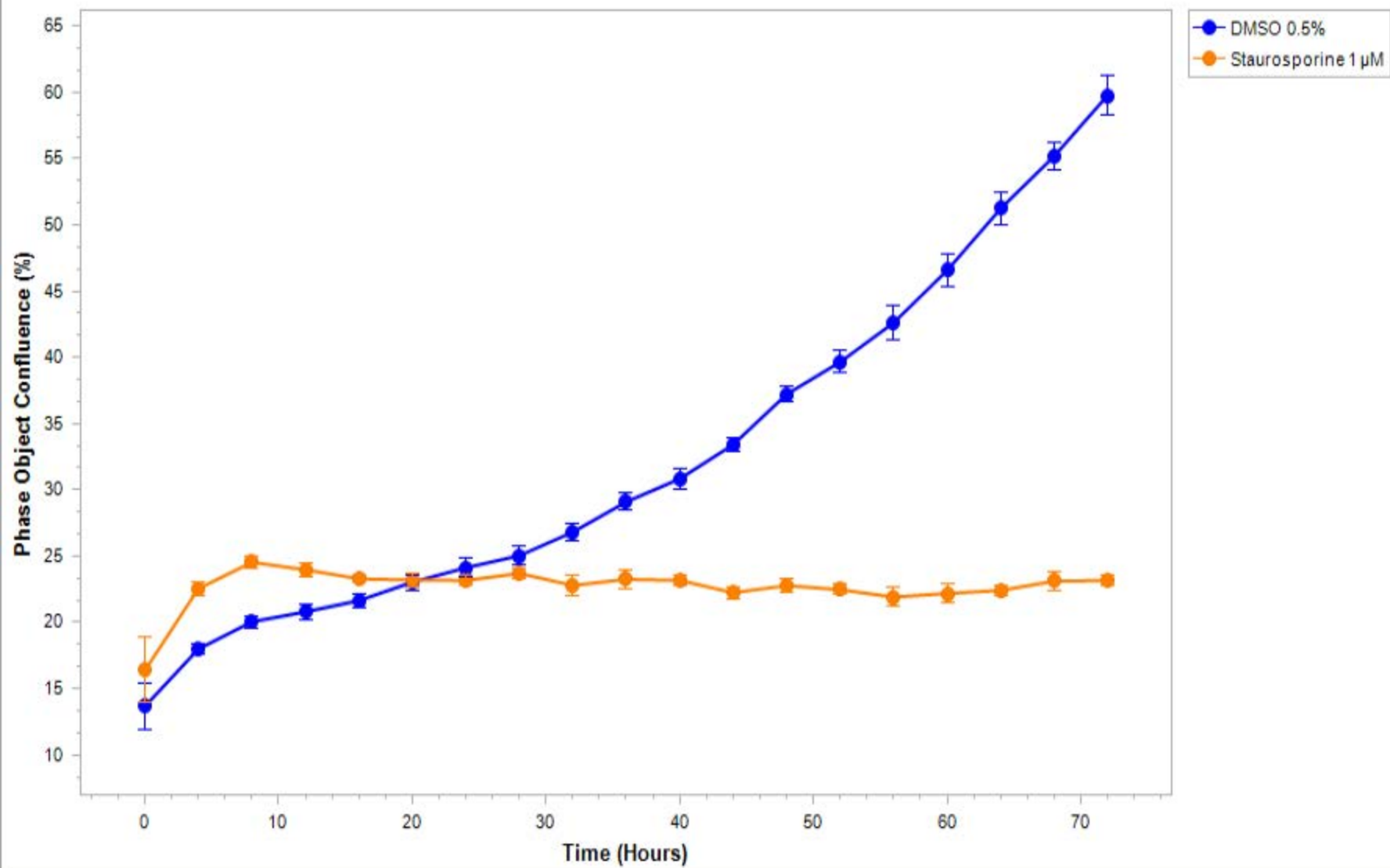

# NBL30-0719

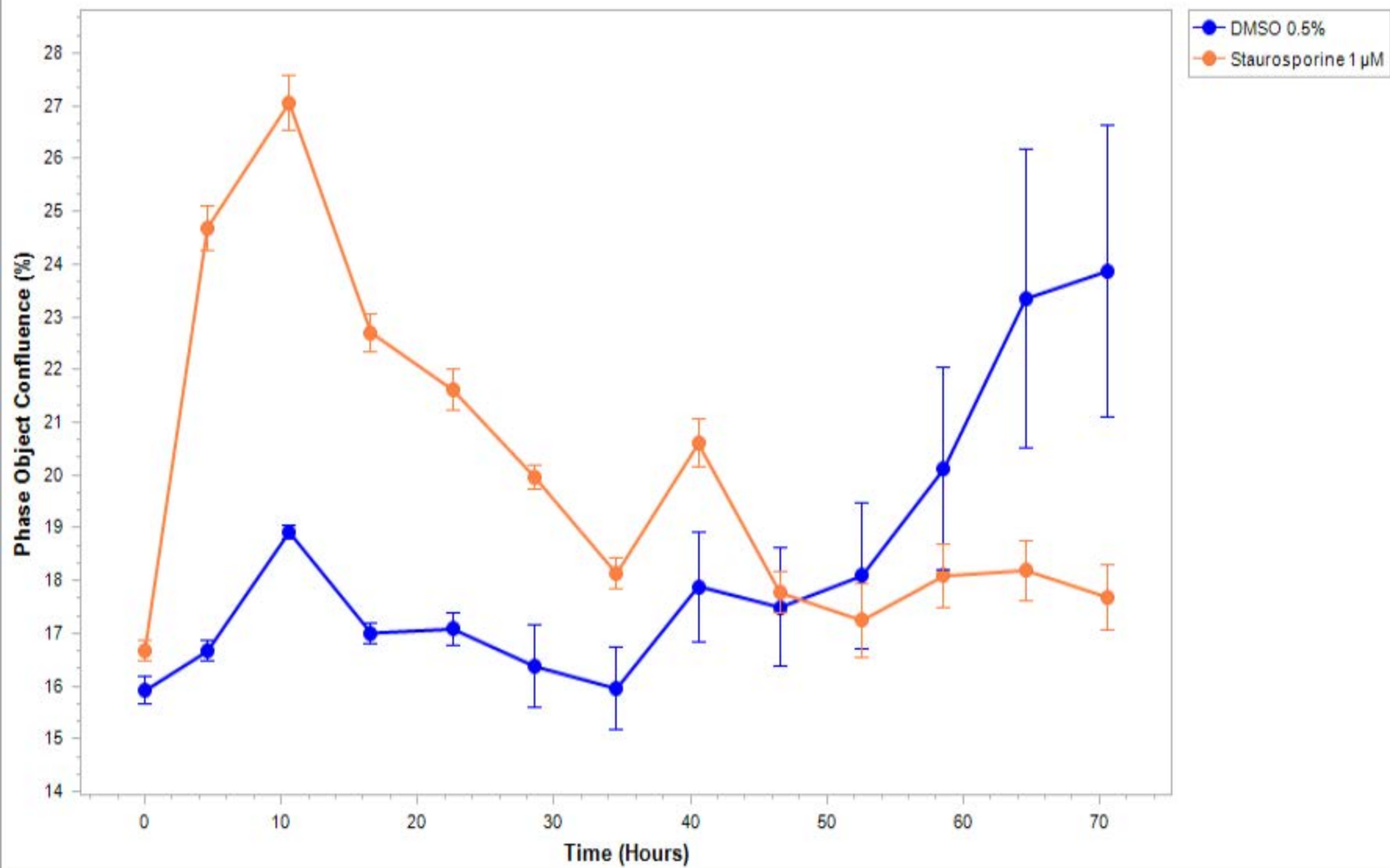

# NBL03-1019

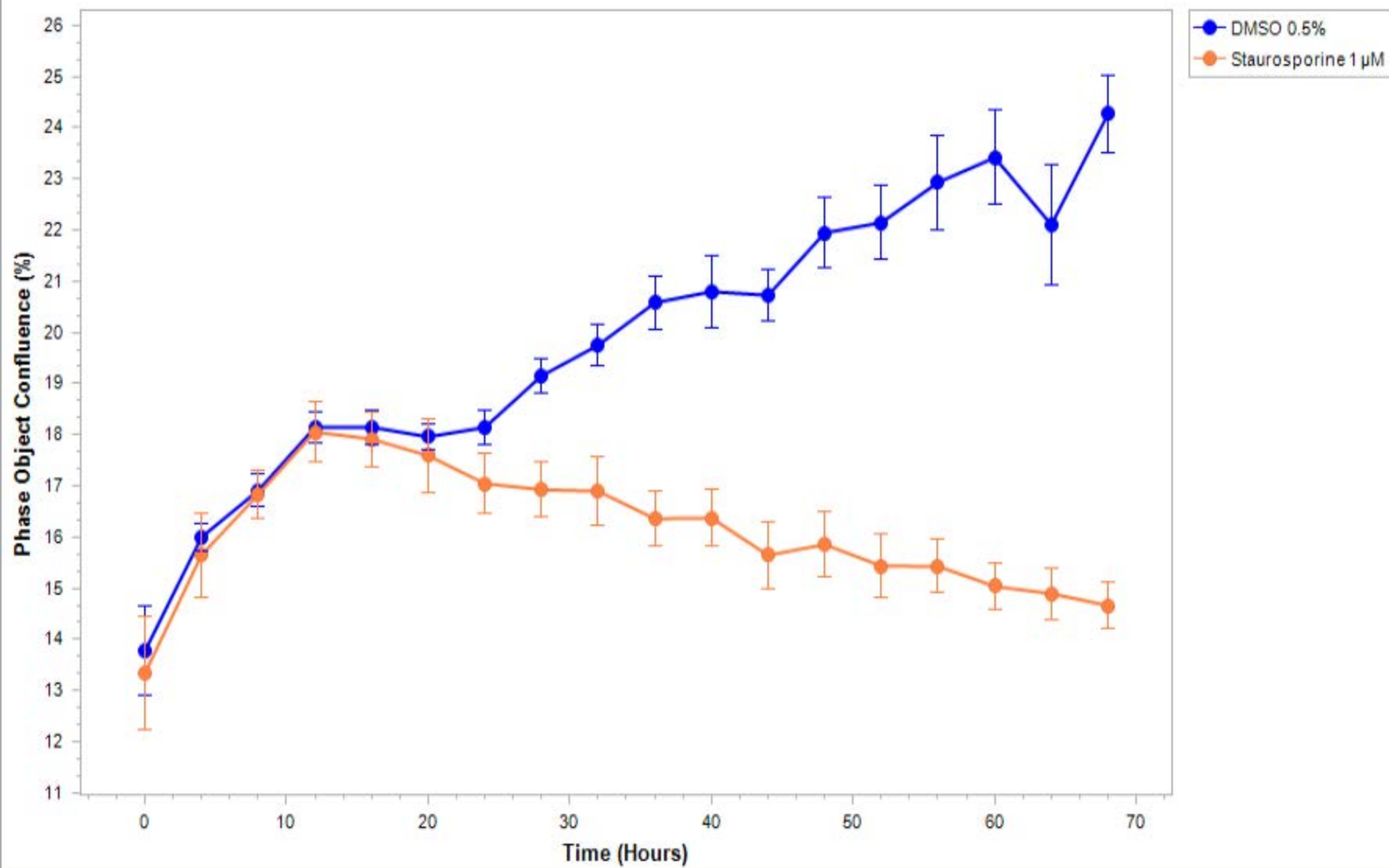

# NBL29-1019

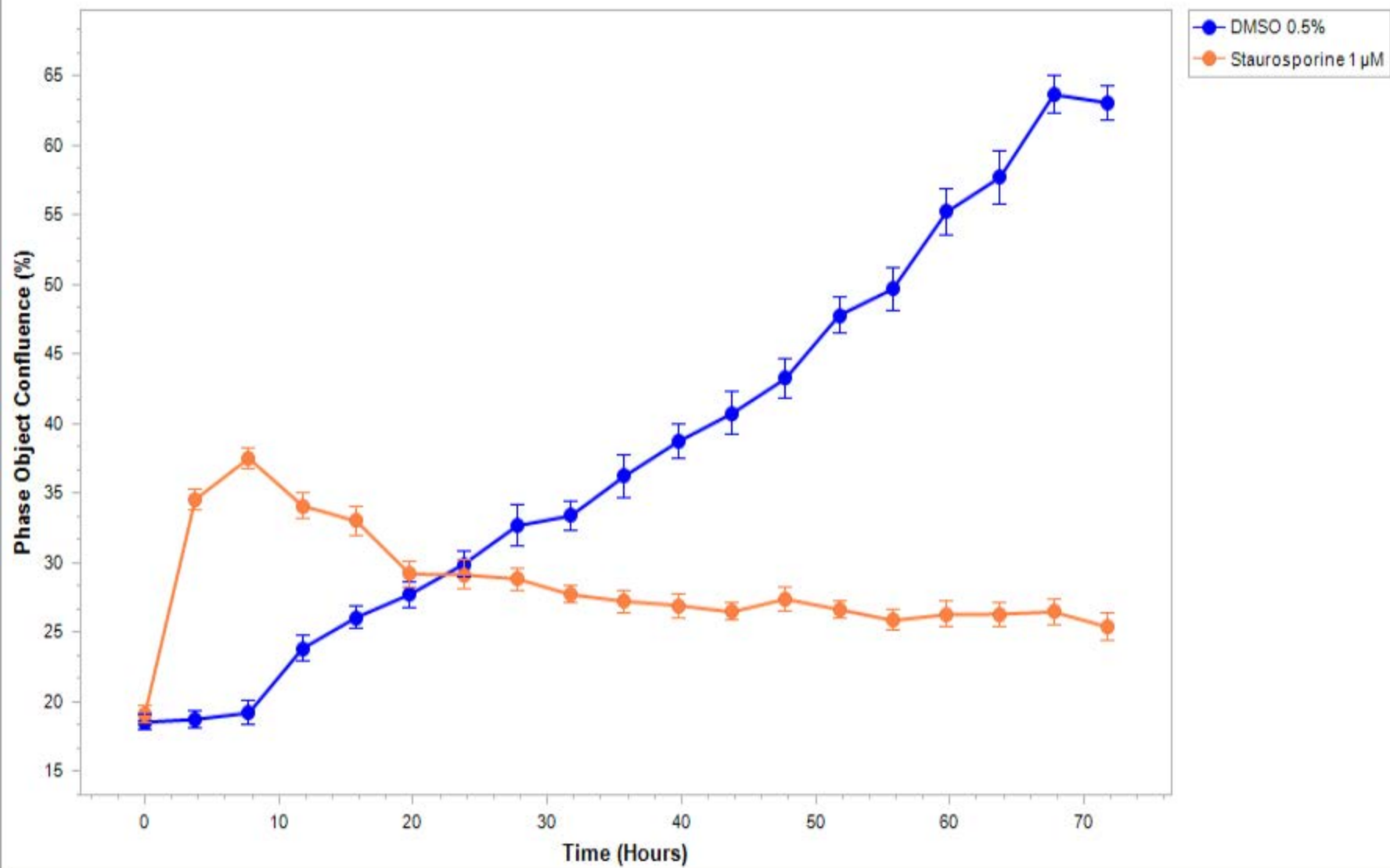

# NBL03-1219A

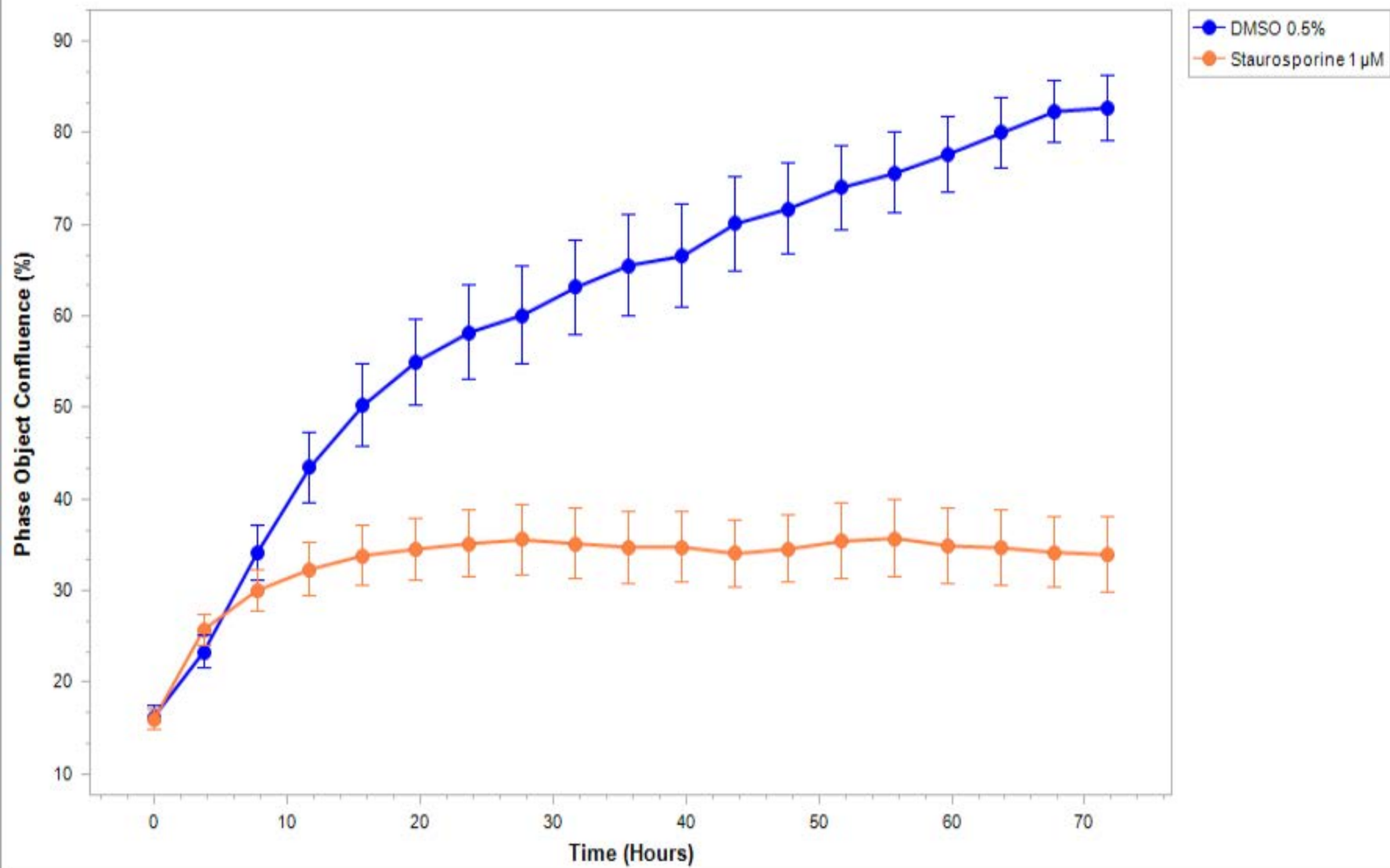

# NBL03-1219B

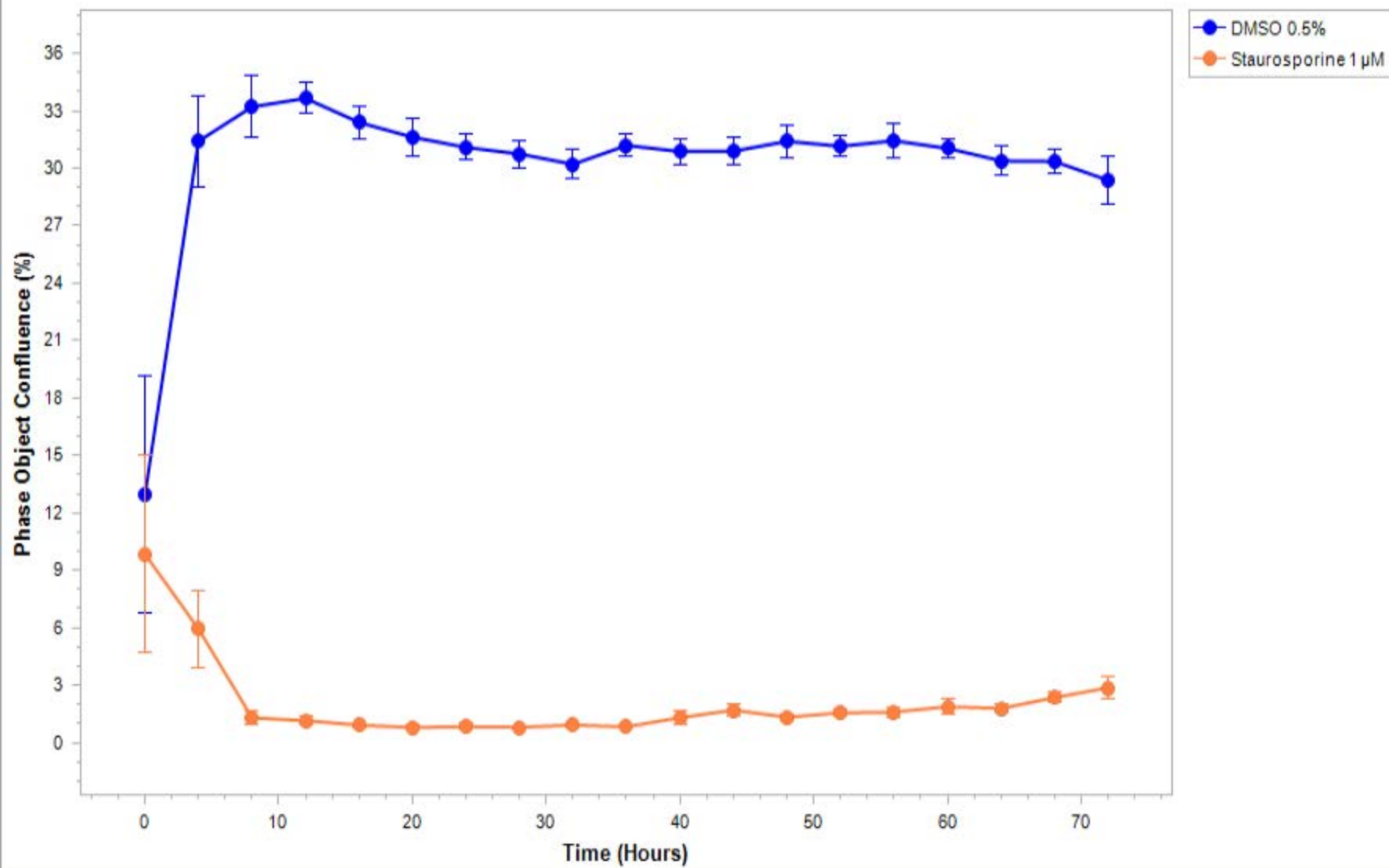

# NBL17-1219

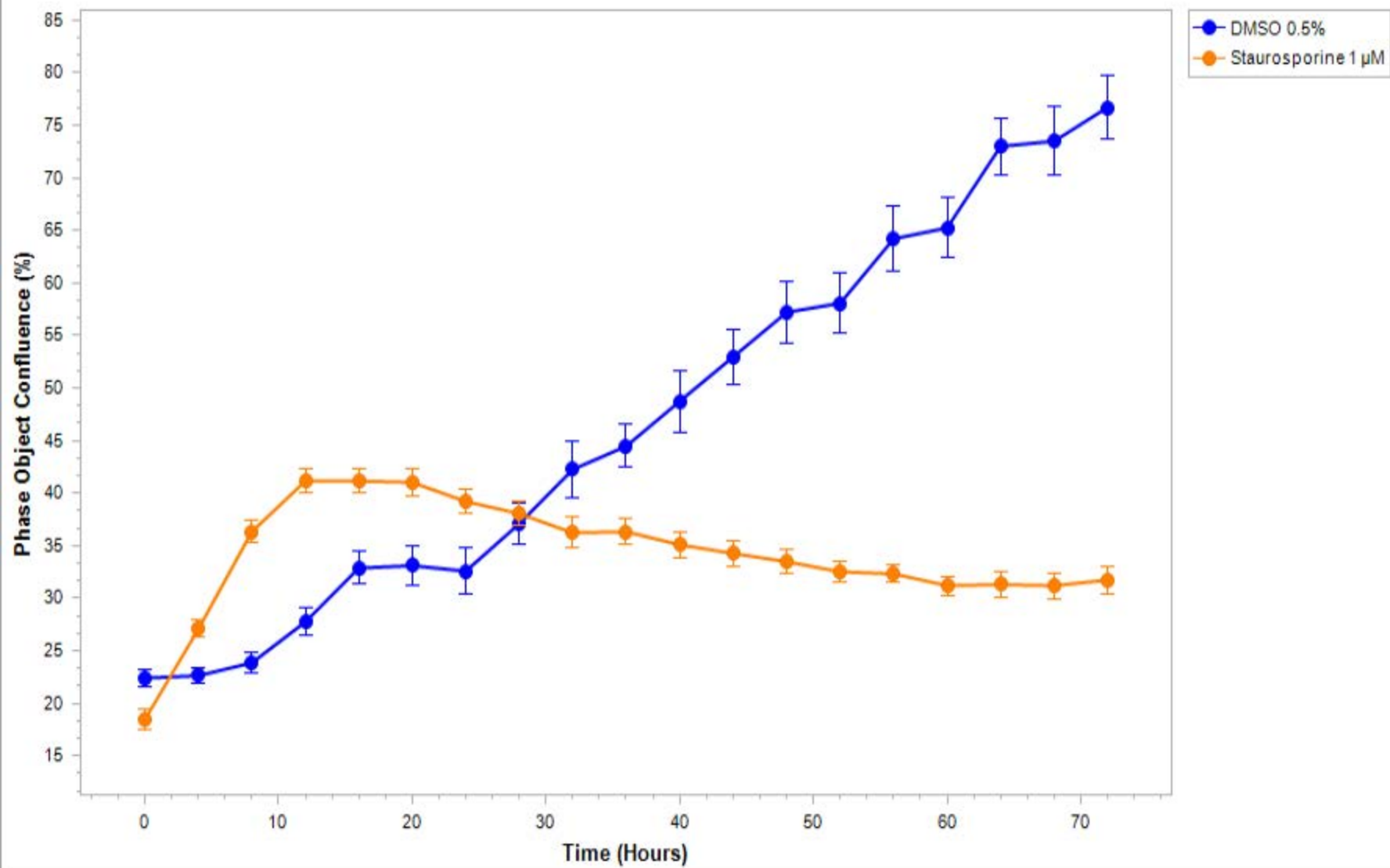

# NBL17-0120

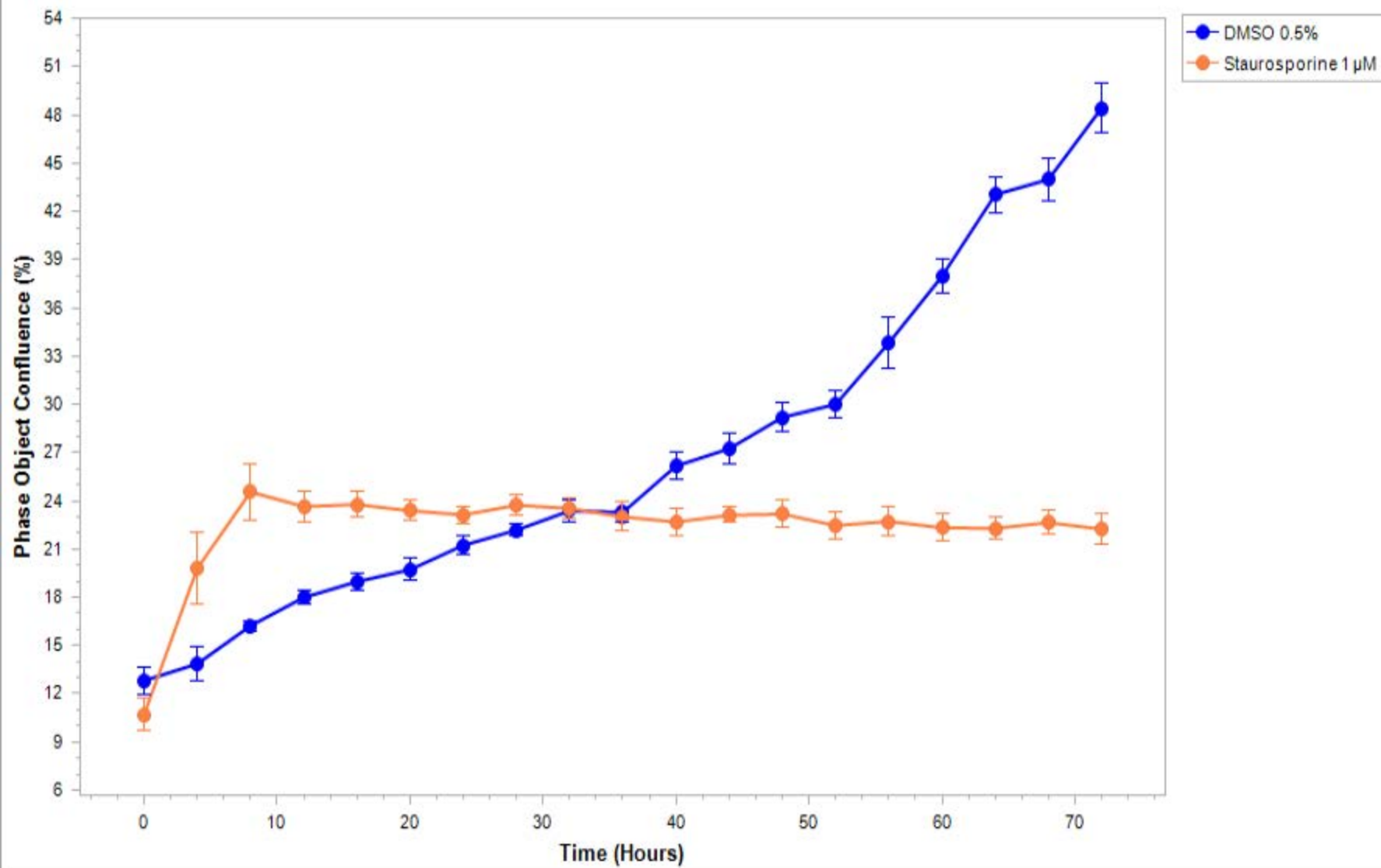

# NBL21-0120

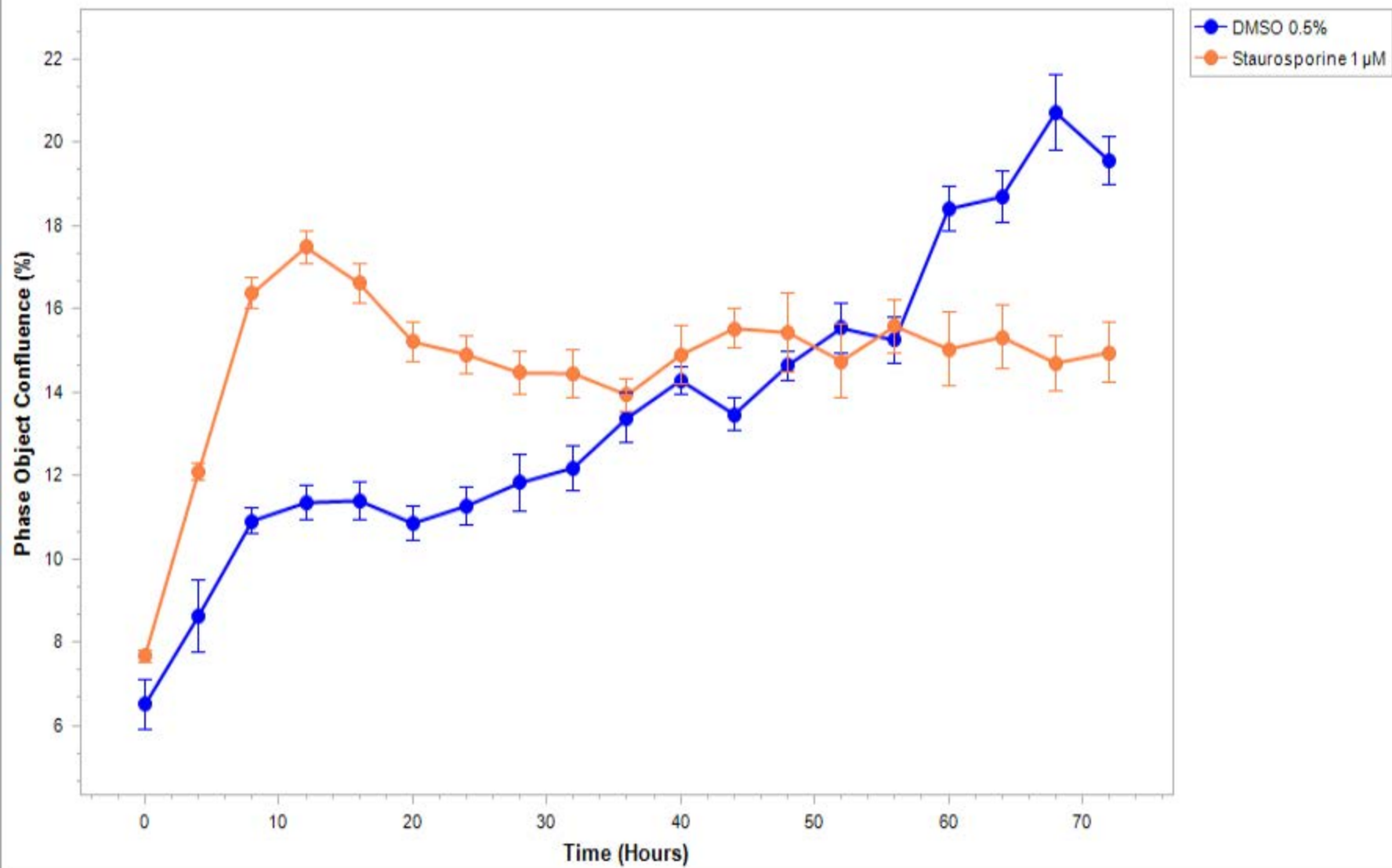

Supplement: Supplementary Data Sheet 2 — Reactome and GO annotations [file DataSheet_2.pdf]
