# Supplementary material for: Integrated Genomic Profiling and Drug Screening of Patient-Derived Cultures Identifies Individualized Copy Number-Dependent Susceptibilities Involving PI3K Pathway and 17q Genes in Neuroblastoma
Source: Front Oncol. 2021 Oct 14;11:709525. doi: 10.3389/fonc.2021.709525 (PMC8551924; doi:10.3389/fonc.2021.709525)

Applied Biosystems
GeneMapper Software 5

HM 20210127

Sample File	Sample Name	Panel	SQI	OS	SQ
A7_A1 NBL 06 1218_Sample_20210126_165322.fsa	A1 NBL 06 1218	PowerPlex_21_POP1_Panels_vSS2.0			

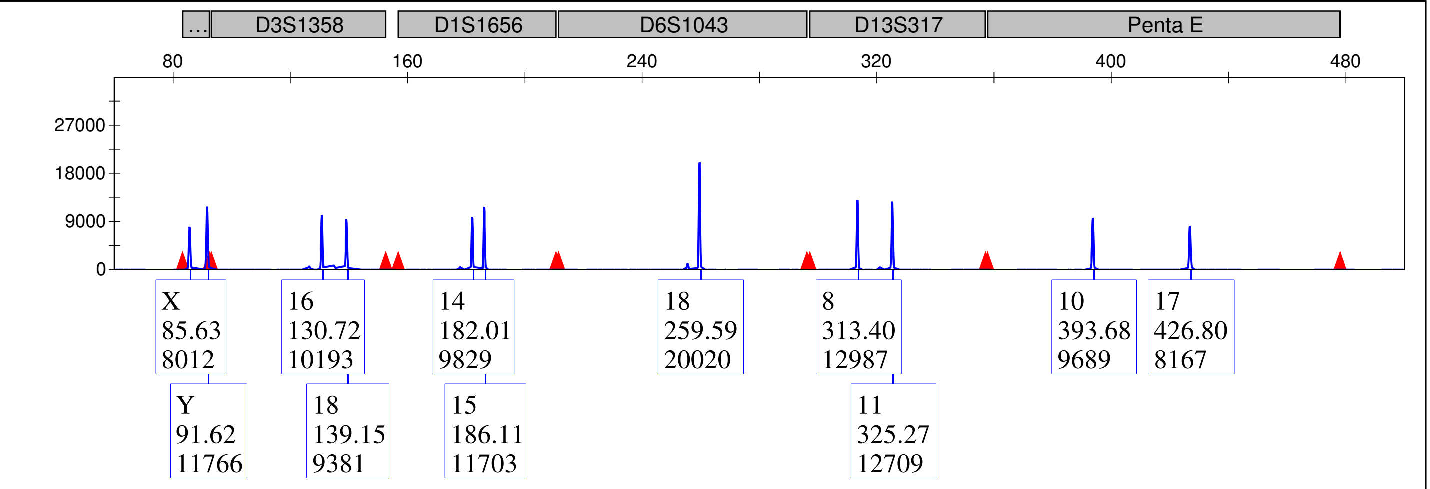

A7_A1 NBL 06 1218_Sample_20210126_165322.fsa	A1 NBL 06 1218	PowerPlex_21_POP1_Panels_vSS2.0			

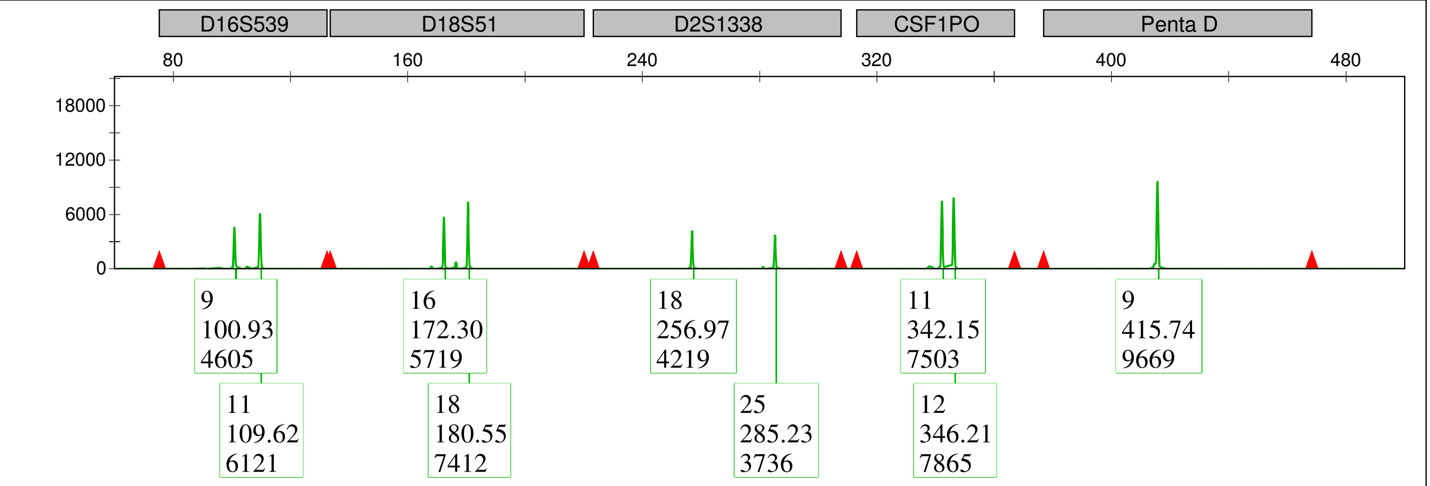

A7_A1 NBL 06 1218_Sample_20210126_165322.fsa	A1 NBL 06 1218	PowerPlex_21_POP1_Panels_vSS2.0			

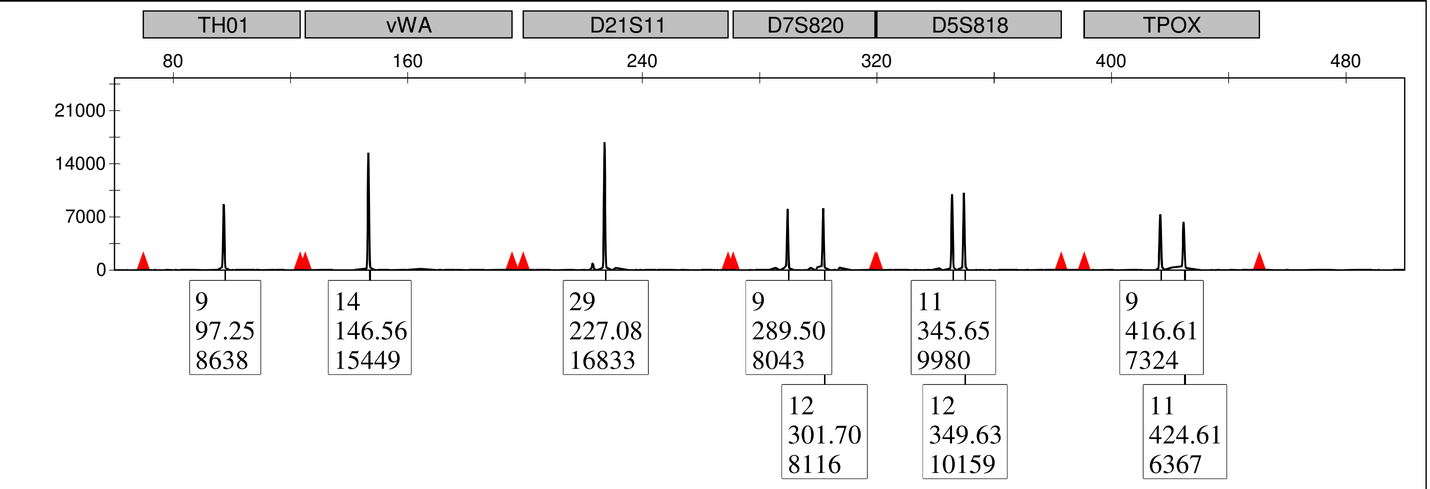

Sample File	Sample Name	Panel	SQI	OS	SQ
A7_A1 NBL 06 1218_Sample_20210126_165322.fsa	A1 NBL 06 1218	PowerPlex_21_POP1_Panels_vSS2.0			

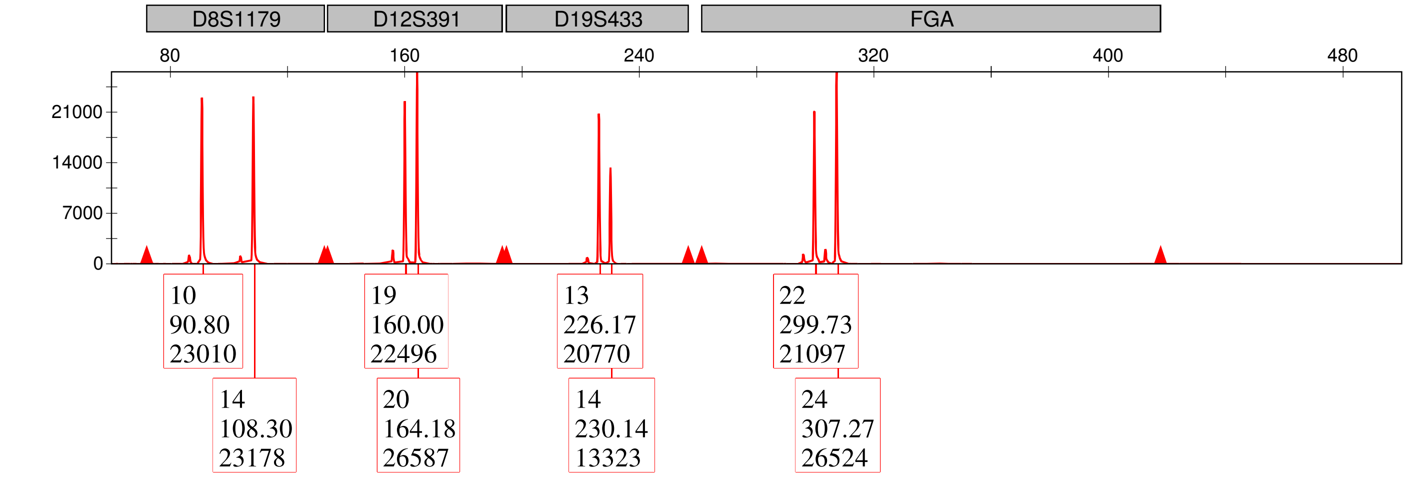

Sample File	Sample Name	Panel	SQI	OS	SQ
B7_B1 KD 18 2310 A10_Sample_20210126_165323.fsa	B1 KD 18 2310 A10	PowerPlex_21_POP1_Panels_vSS2.0			

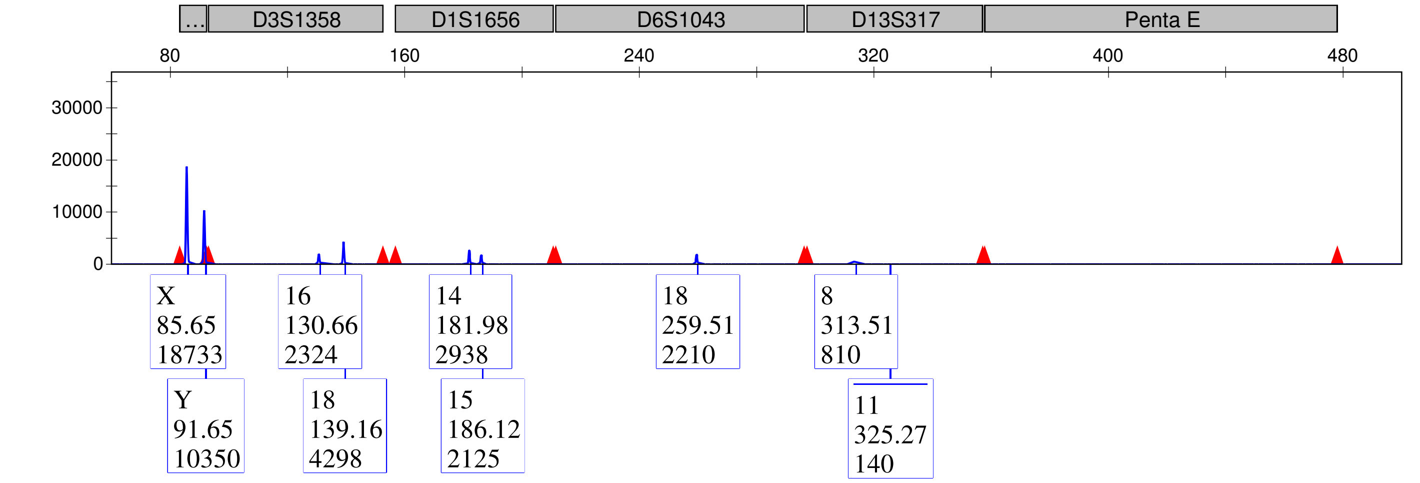

Sample File	Sample Name	Panel	SQI	OS	SQ
B7_B1 KD 18 2310 A10_Sample_20210126_165323.fsa	B1 KD 18 2310 A10	PowerPlex_21_POP1_Panels_vSS2.0			

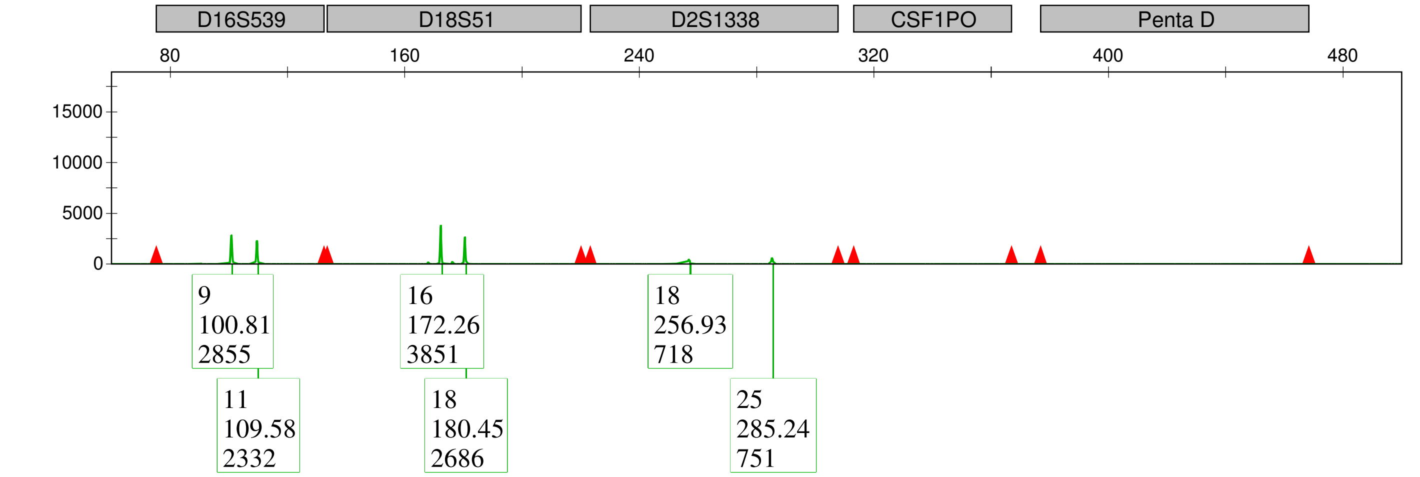

Sample File	Sample Name	Panel	SQI	OS	SQ
B7_B1 KD 18 2310 A10_Sample_20210126_165323.fsa	B1 KD 18 2310 A10	PowerPlex_21_POP1_Panels_vSS2.0			

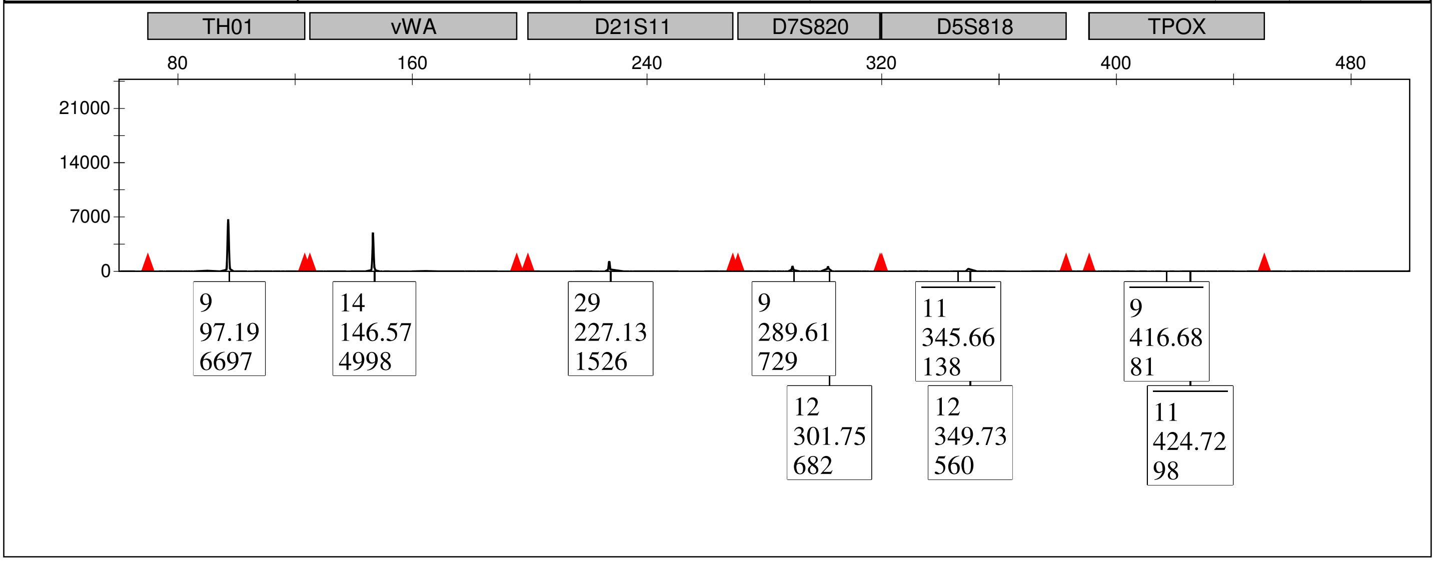

B7_B1 KD 18 2310 A10_Sample_20210126_165323.fsa	B1 KD 18 2310 A10	PowerPlex_21_POP1_Panels_vSS2.0			

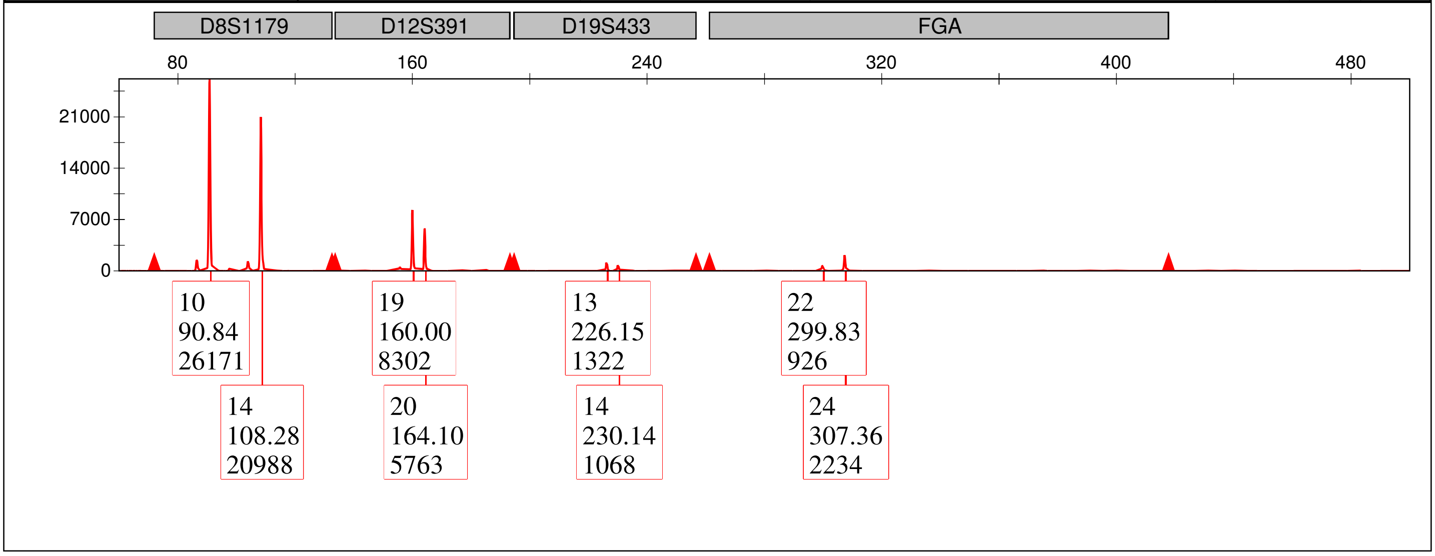

Sample File	Sample Name	Panel	SQI	OS	SQ
C7_A2 NBL 18 0619_Sample_20210126_165324.fsa	A2 NBL 18 0619	PowerPlex_21_POP1_Panels_vSS2.0			

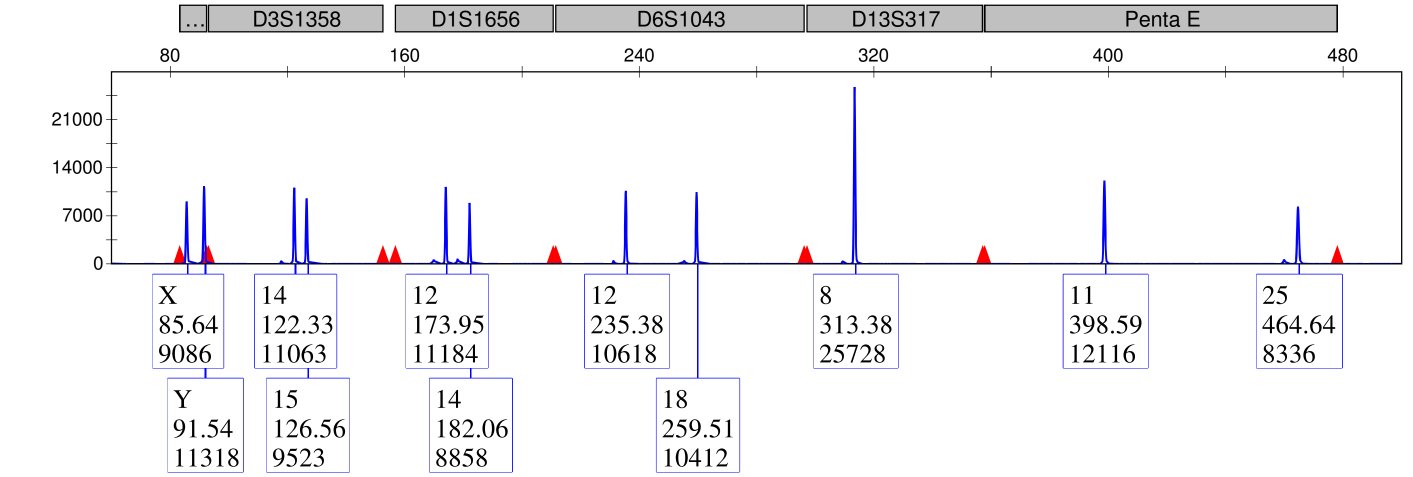

C7_A2 NBL 18 0619_Sample_20210126_165324.fsa	A2 NBL 18 0619	PowerPlex_21_POP1_Panels_vSS2.0			

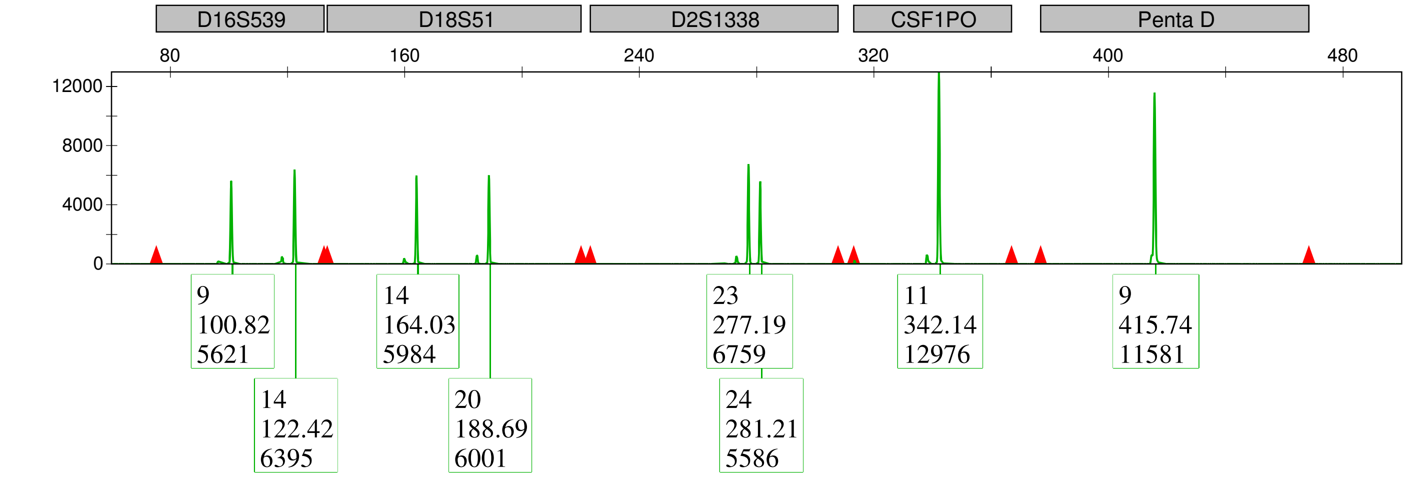

C7_A2 NBL 18 0619_Sample_20210126_165324.fsa	A2 NBL 18 0619	PowerPlex_21_POP1_Panels_vSS2.0			

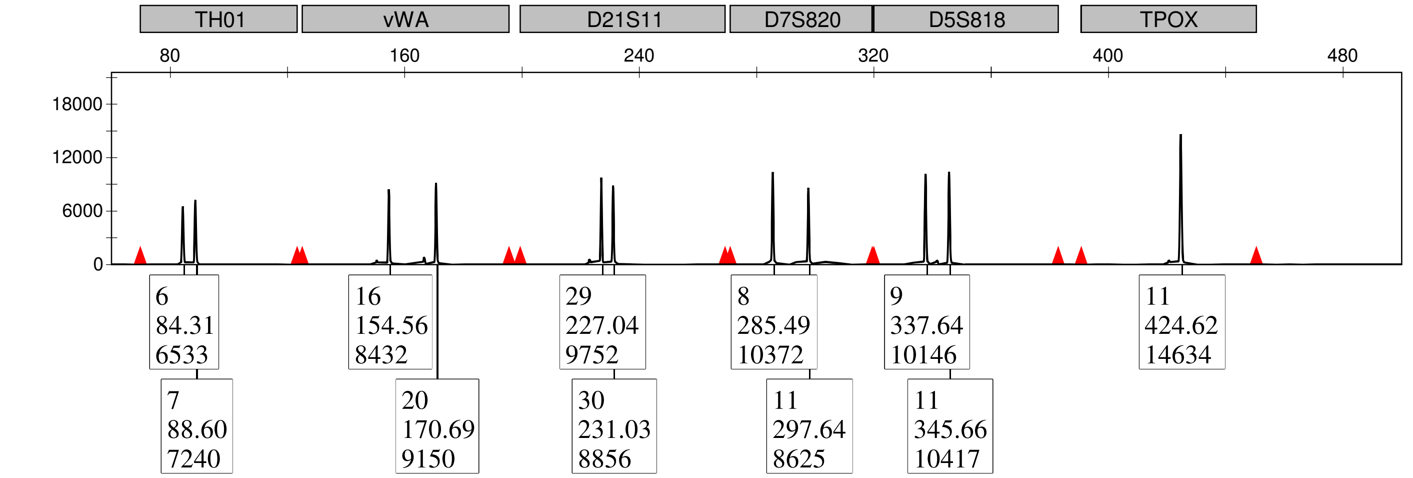

Sample File	Sample Name	Panel	SQI	OS	SQ
C7_A2 NBL 18 0619_Sample_20210126_165324.fsa	A2 NBL 18 0619	PowerPlex_21_POP1_Panels_vSS2.0			

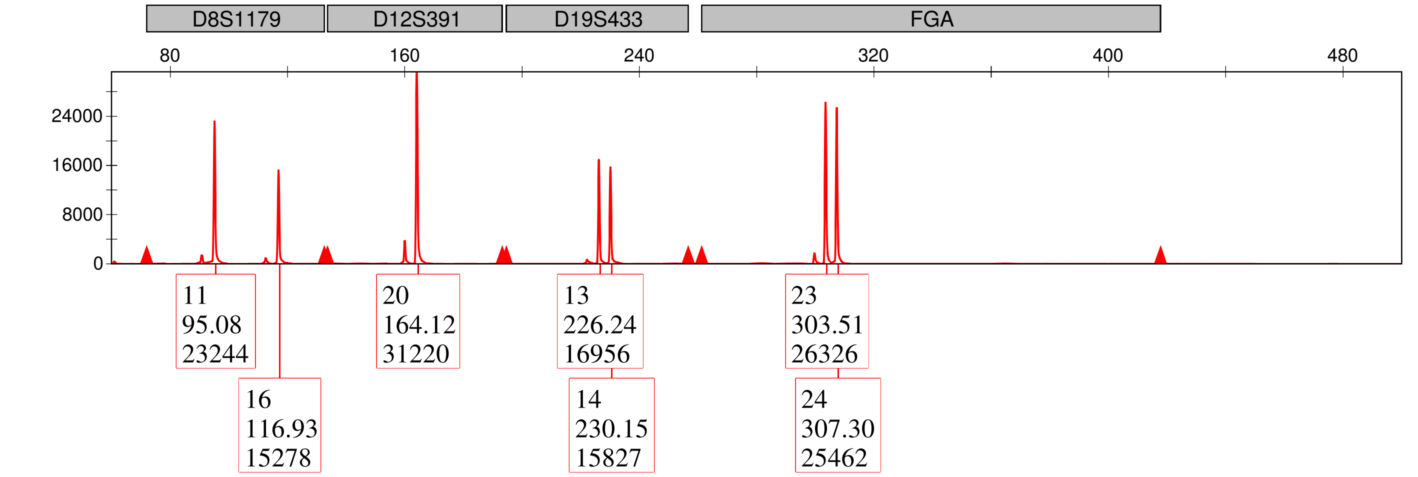

Sample File	Sample Name	Panel	SQI	OS	SQ
D7_B2 KD 19 1117_Sample_20210126_165325.fsa	B2 KD 19 1117	PowerPlex_21_POP1_Panels_vSS2.0			

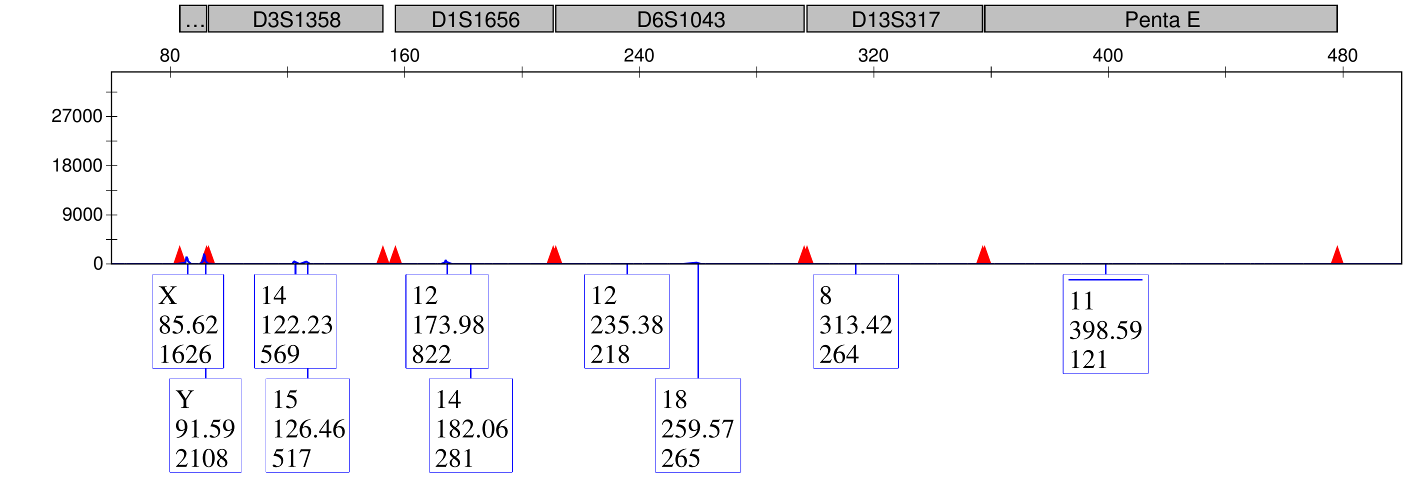

Sample File	Sample Name	Panel	SQI	OS	SQ
D7_B2 KD 19 1117_Sample_20210126_165325.fsa	B2 KD 19 1117	PowerPlex_21_POP1_Panels_vSS2.0			

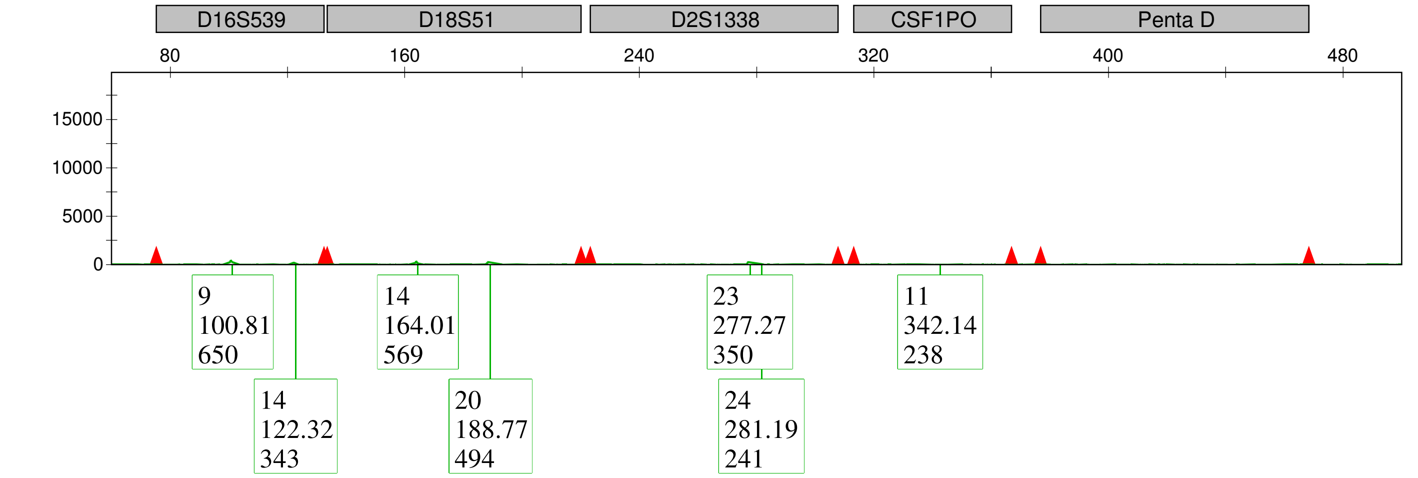

Sample File	Sample Name	Panel	SQI	OS	SQ
D7_B2 KD 19 1117_Sample_20210126_165325.fsa	B2 KD 19 1117	PowerPlex_21_POP1_Panels_vSS2.0			

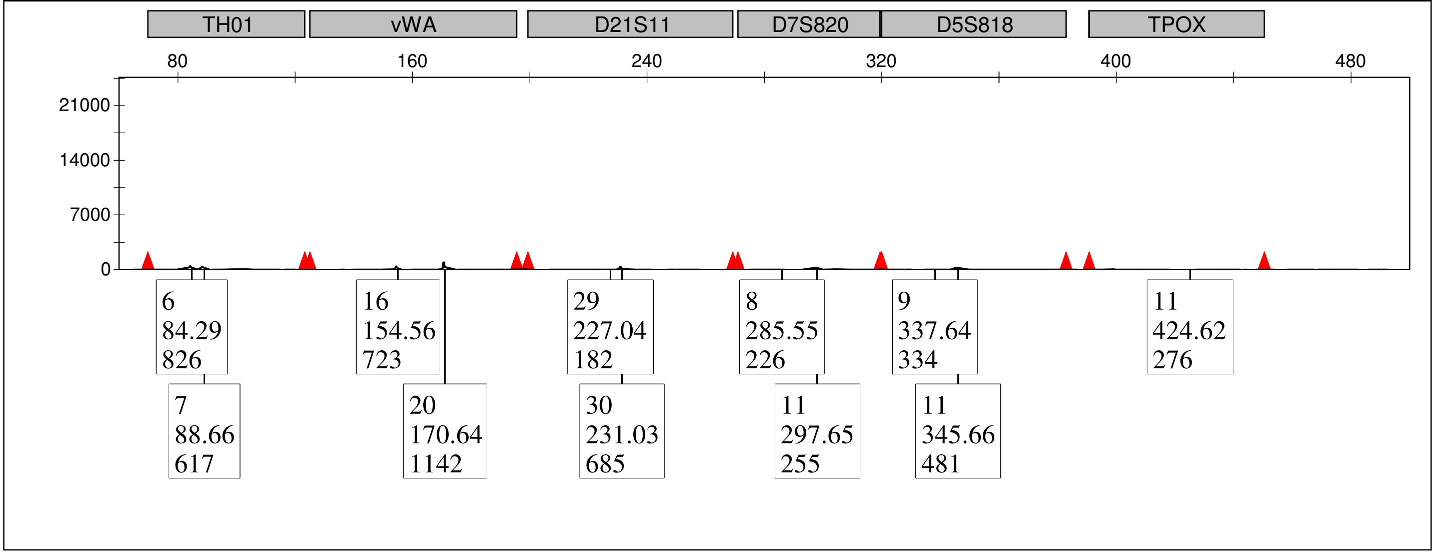

Sample File	Sample Name	Panel	SQI	OS	SQ
D7_B2 KD 19 1117_Sample_20210126_165325.fsa	B2 KD 19 1117	PowerPlex_21_POP1_Panels_vSS2.0			

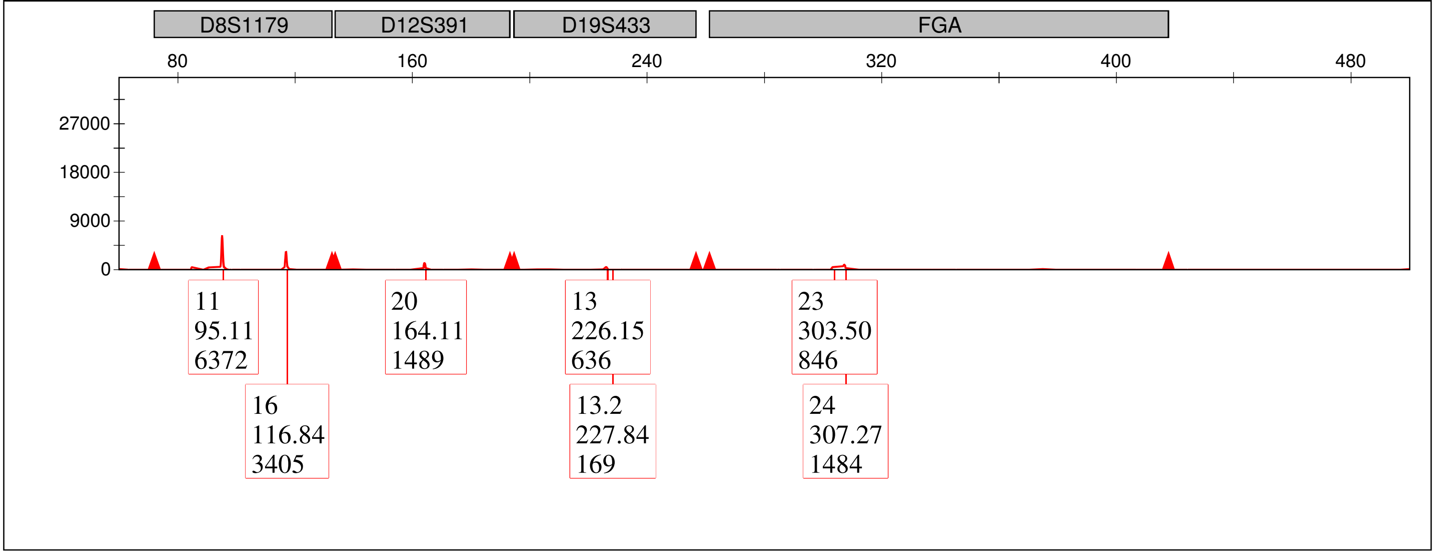

GeneMapper Software 5

HM 20210127

Sample File	Sample Name	Panel	SQI	OS	SQ
E7_A3 NBL 25 0619_Sample_20210126_173628.fsa	A3 NBL 25 0619	PowerPlex_21_POP1_Panels_vSS2.0			

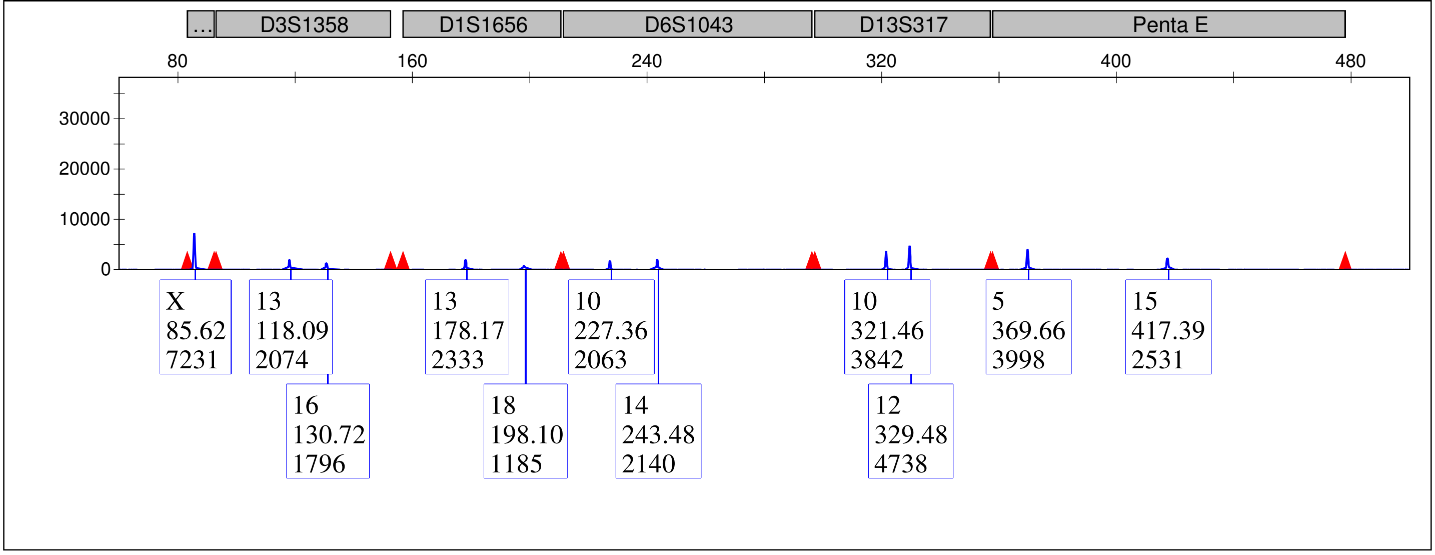

Sample File	Sample Name	Panel	SQI	OS	SQ
E7_A3 NBL 25 0619_Sample_20210126_173628.fsa	A3 NBL 25 0619	PowerPlex_21_POP1_Panels_vSS2.0			

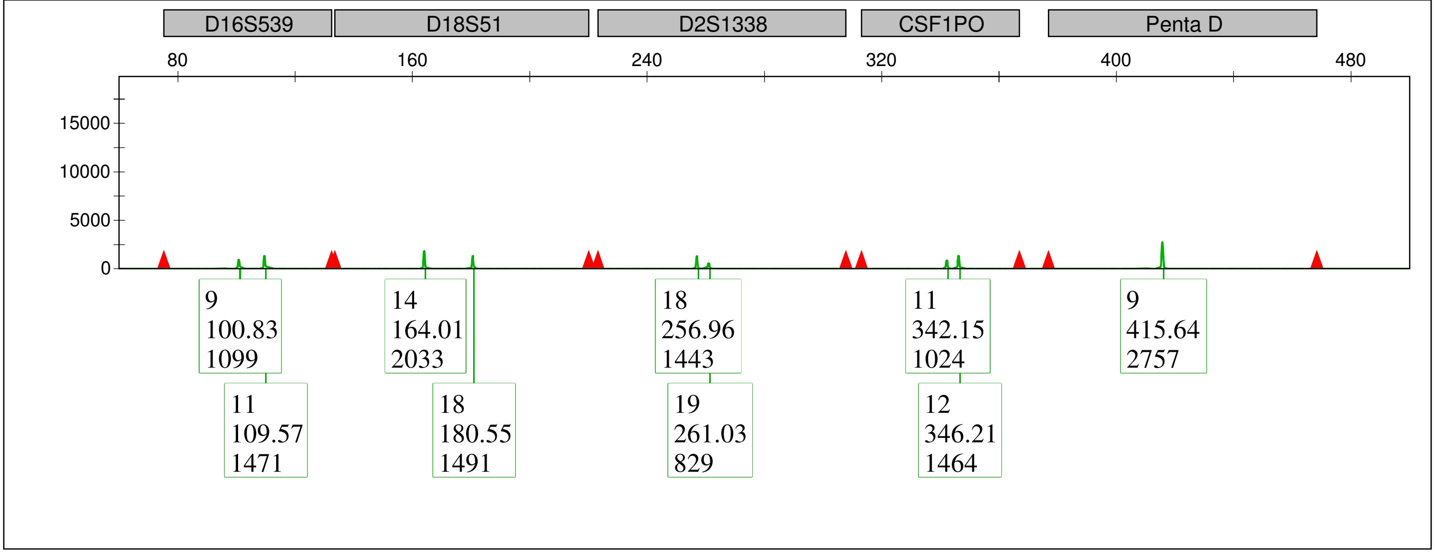

Sample File	Sample Name	Panel	SQI	OS	SQ
E7_A3 NBL 25 0619_Sample_20210126_173628.fsa	A3 NBL 25 0619	PowerPlex_21_POP1_Panels_vSS2.0			

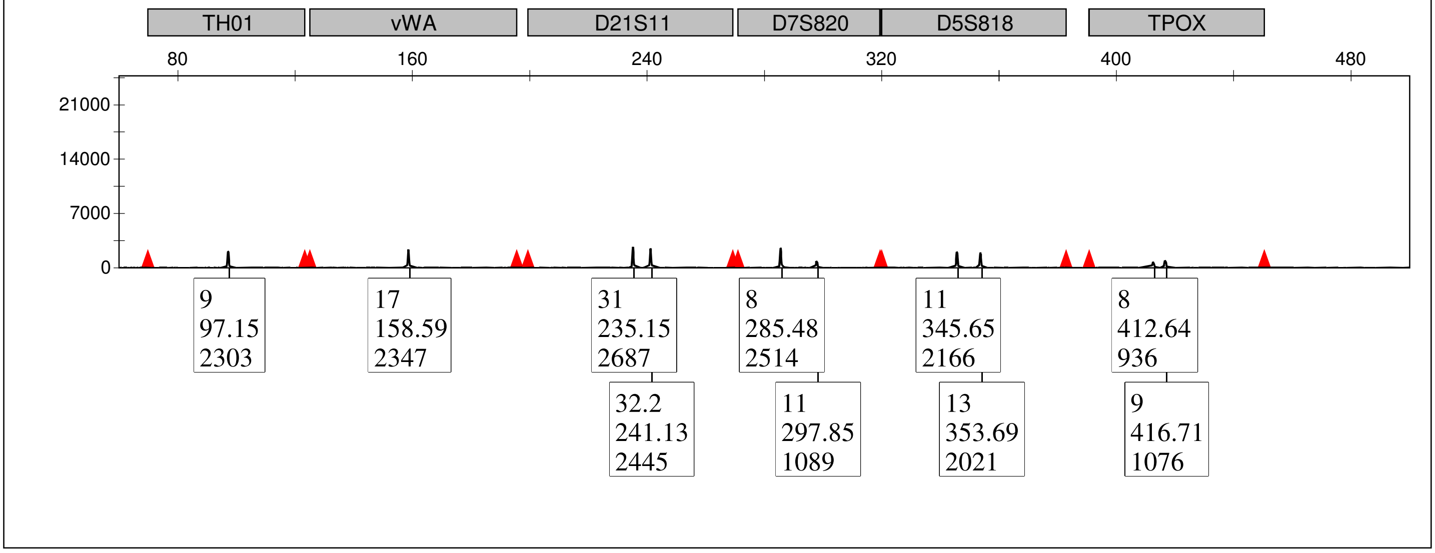

Sample File	Sample Name	Panel	SQI	OS	SQ
E7 A3 NBL 25 0619 Sample 20210126 173628.fsa	A3 NBL 25 0619	PowerPlex 21 POP1 Panels vSS2.0			

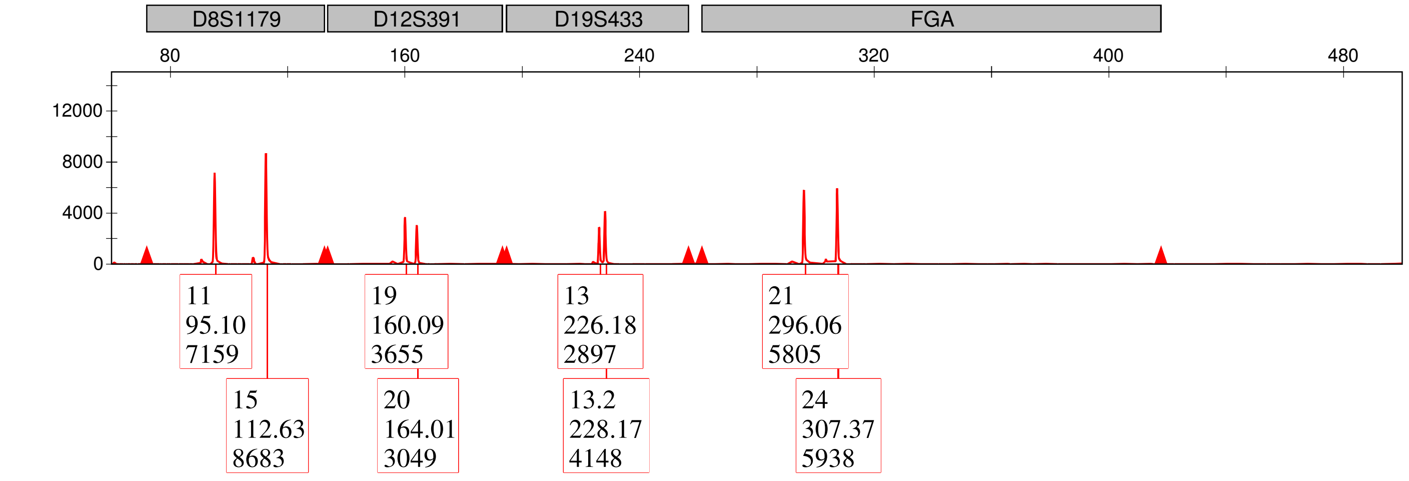

F7 B3 KD 19 1179 A Sample 20210126 173629.fsa	B3 KD 19 1179 A	PowerPlex 21 POP1 Panels vSS2.0			

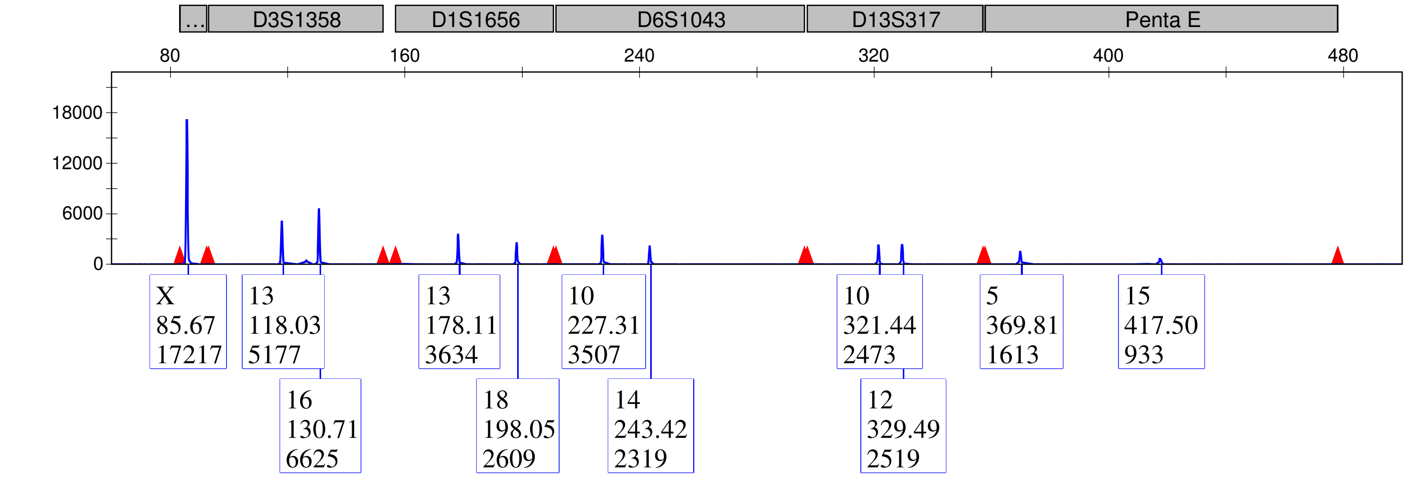

F7 B3 KD 19 1179 A Sample 20210126 173629.fsa	B3 KD 19 1179 A	PowerPlex 21 POP1 Panels vSS2.0			

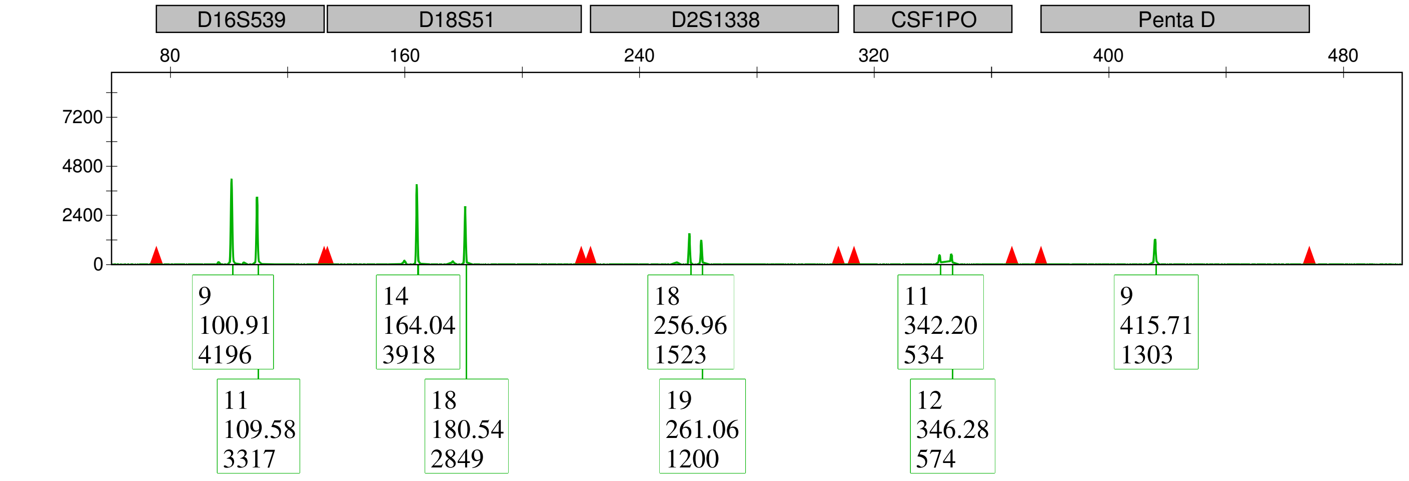

Sample File	Sample Name	Panel	SQI	OS	SQ
F7_B3 KD 19_1179 A_Sample_20210126_173629.fsa	B3 KD 19_1179 A	PowerPlex_21_POP1_Panels_vSS2.0			

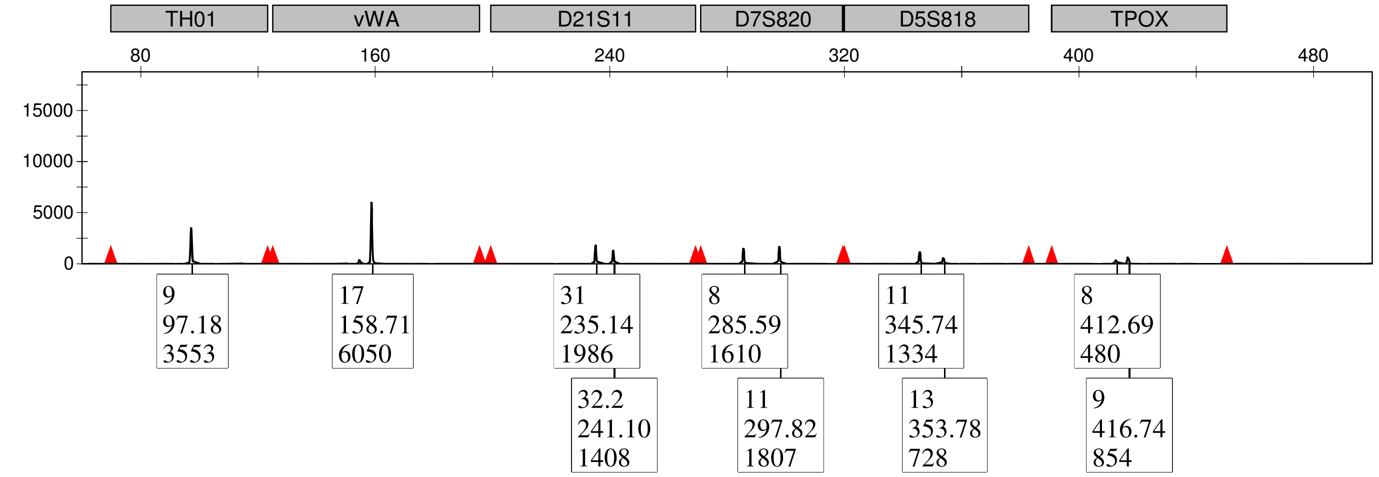

F7_B3 KD 19_1179 A_Sample_20210126_173629.fsa	B3 KD 19_1179 A	PowerPlex_21_POP1_Panels_vSS2.0			

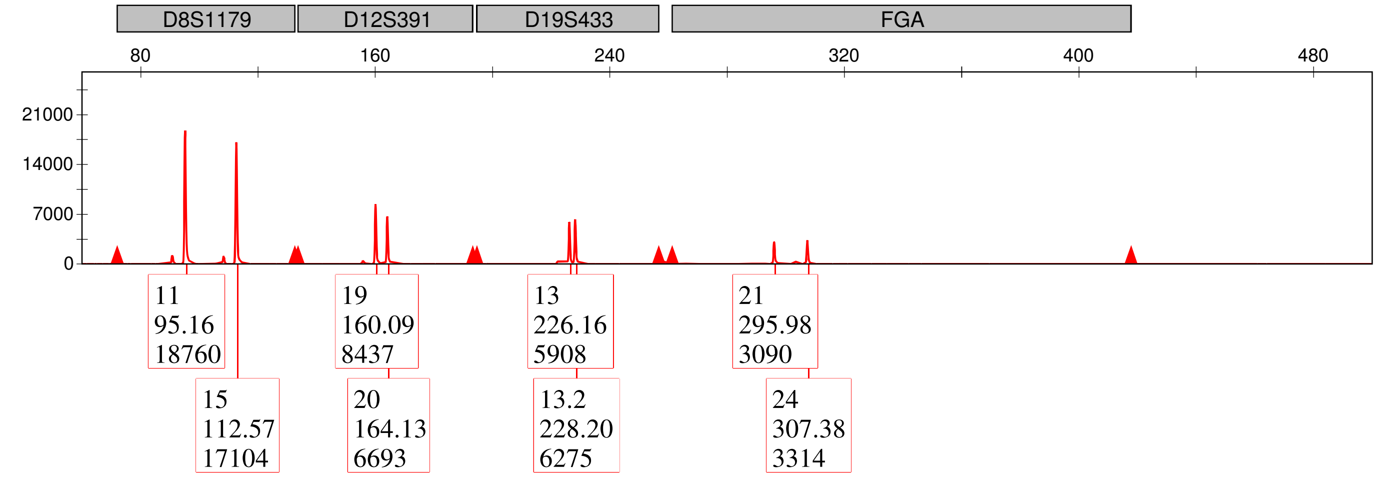

Sample File	Sample Name	Panel	SQI	OS	SQ
G7_A4 NBL 02 0719_Sample_20210126_173630.fsa	A4 NBL 02 0719	PowerPlex_21_POP1_Panels_vSS2.0			

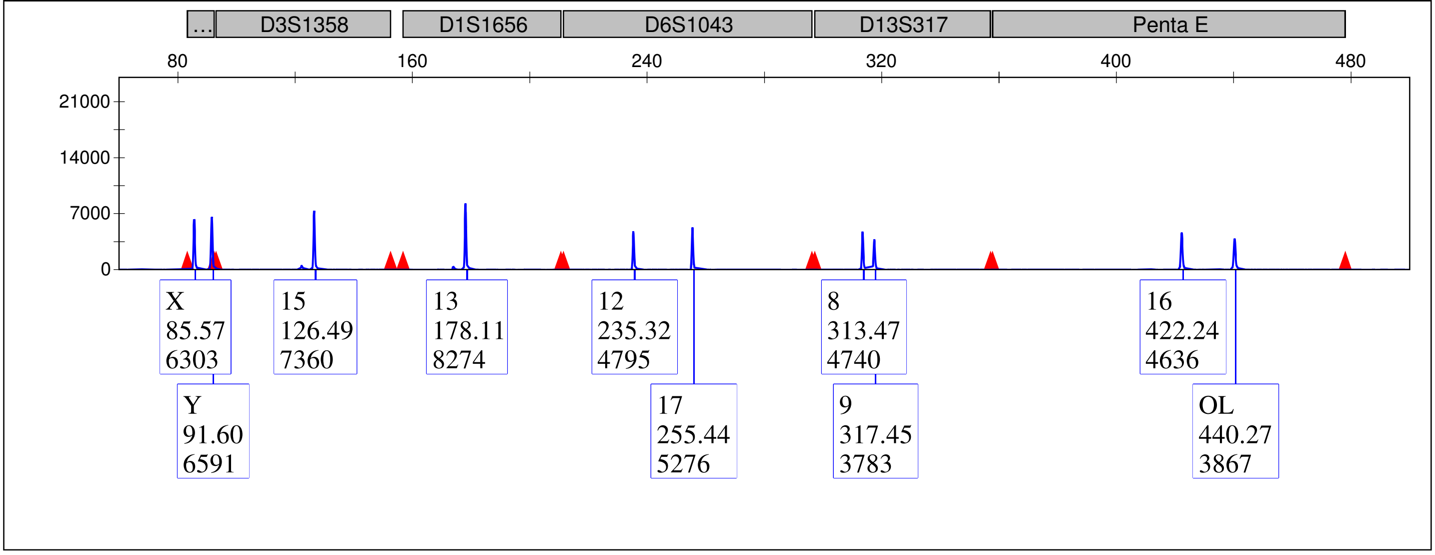

Sample File	Sample Name	Panel	SQI	OS	SQ
G7_A4 NBL 02 0719_Sample_20210126_173630.fsa	A4 NBL 02 0719	PowerPlex_21_POP1_Panels_vSS2.0			

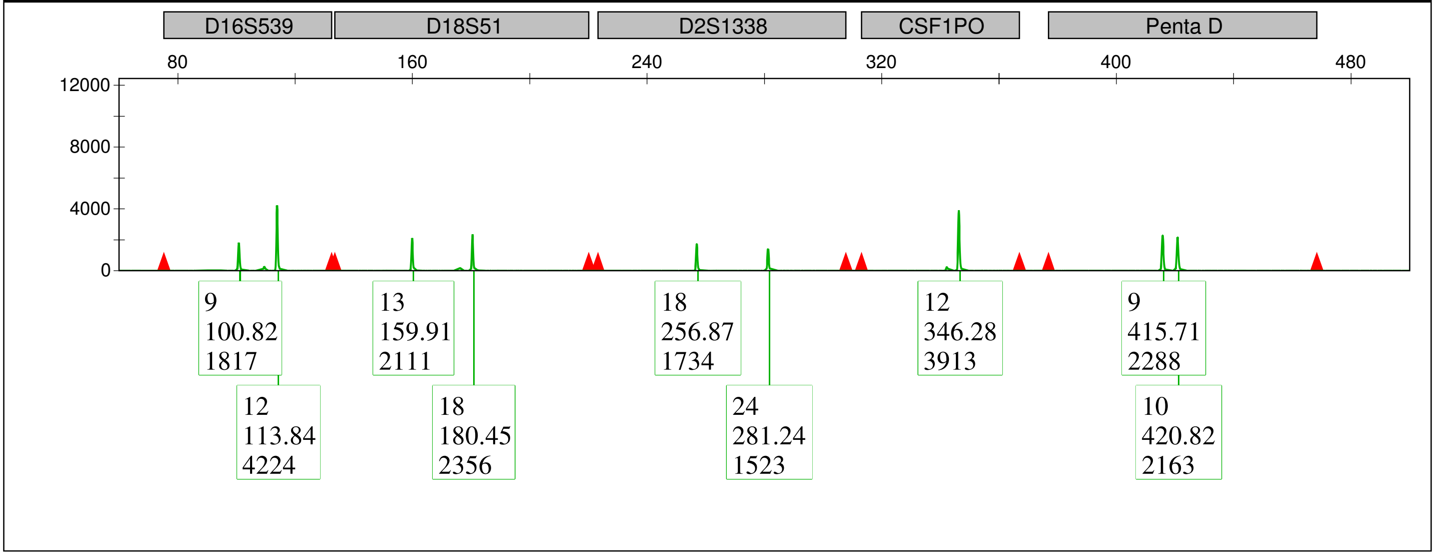

Sample File	Sample Name	Panel	SQI	OS	SQ
G7_A4 NBL 02 0719_Sample_20210126_173630.fsa	A4 NBL 02 0719	PowerPlex_21_POP1_Panels_vSS2.0			

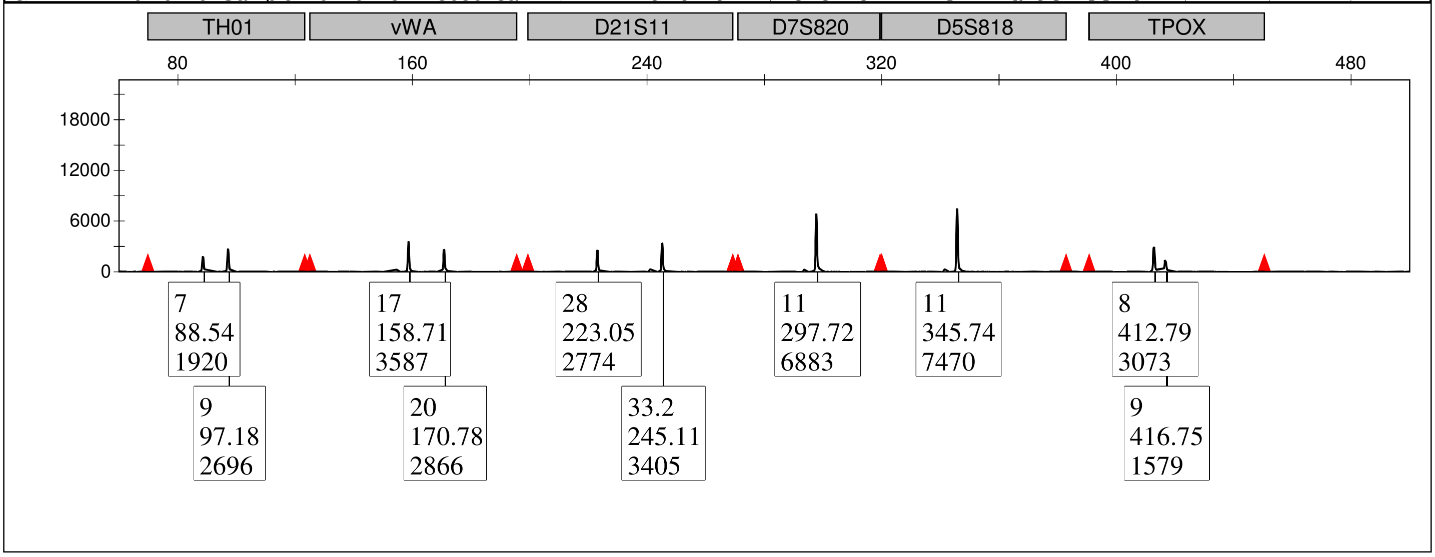

Applied Biosystems
GeneMapper Software 5

HM 20210127

Sample File	Sample Name	Panel	SQI	OS	SQ
G7_A4 NBL 02 0719_Sample_20210126_173630.fsa	A4 NBL 02 0719	PowerPlex_21_POP1_Panels_vSS2.0			

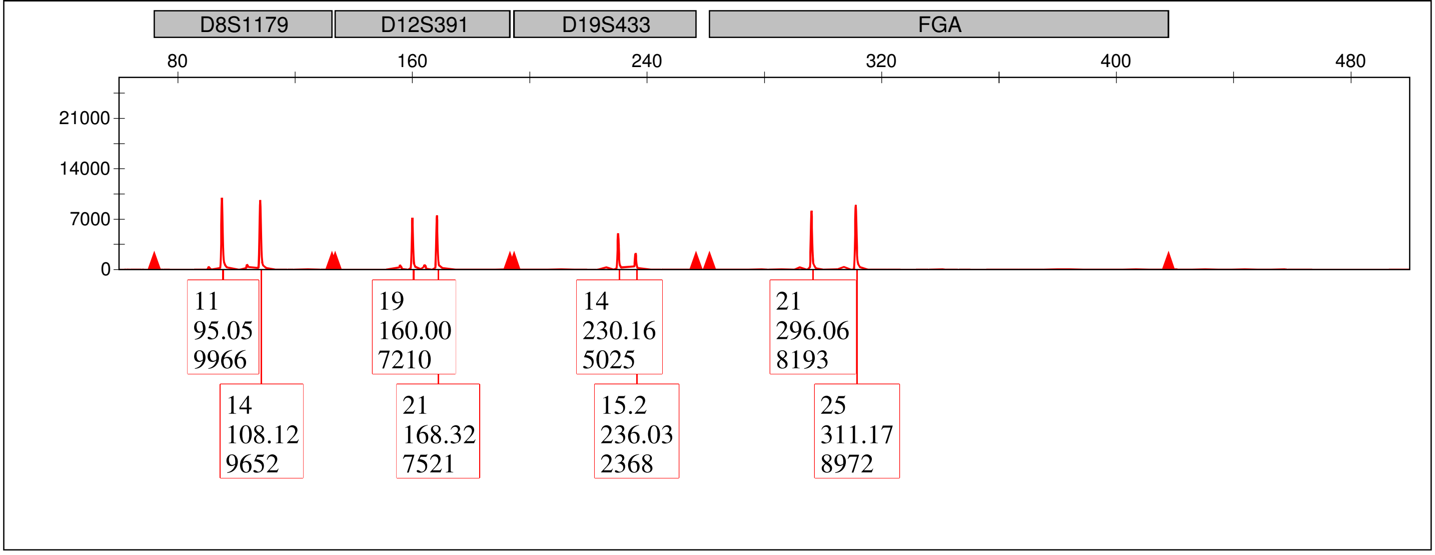

Sample File	Sample Name	Panel	SQI	OS	SQ
H7_B4 KD 19 1239 A9_Sample_20210126_173631.fsa	B4 KD 19 1239 A9	PowerPlex_21_POP1_Panels_vSS2.0			

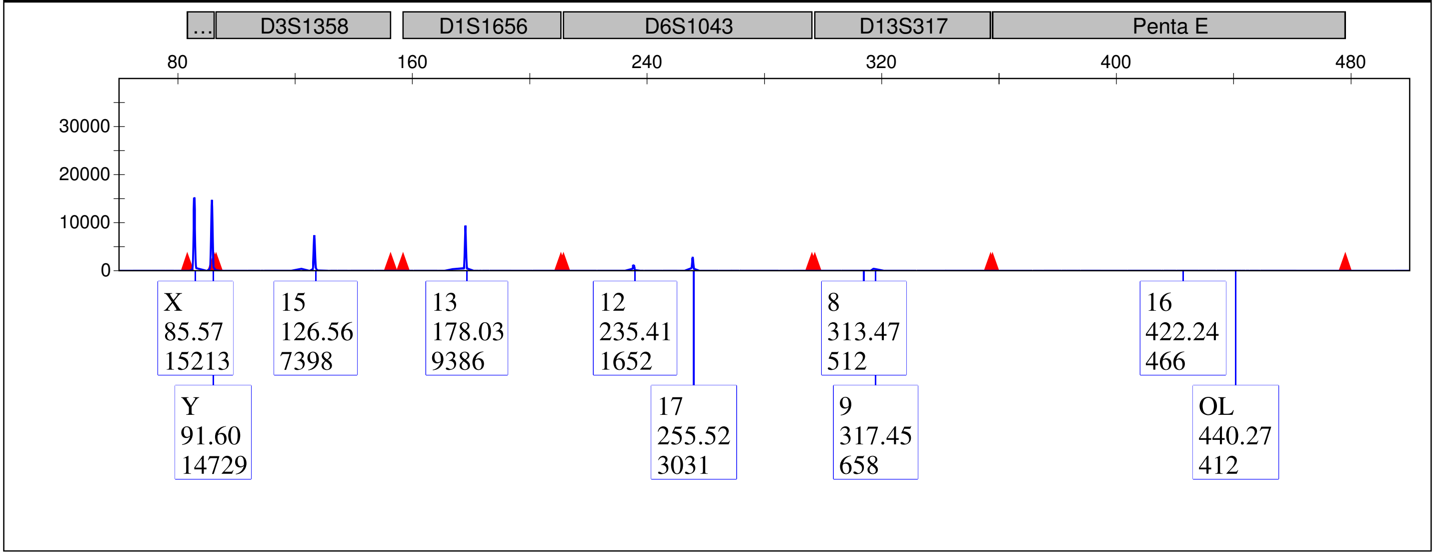

Sample File	Sample Name	Panel	SQI	OS	SQ
H7_B4 KD 19 1239 A9_Sample_20210126_173631.fsa	B4 KD 19 1239 A9	PowerPlex_21_POP1_Panels_vSS2.0			

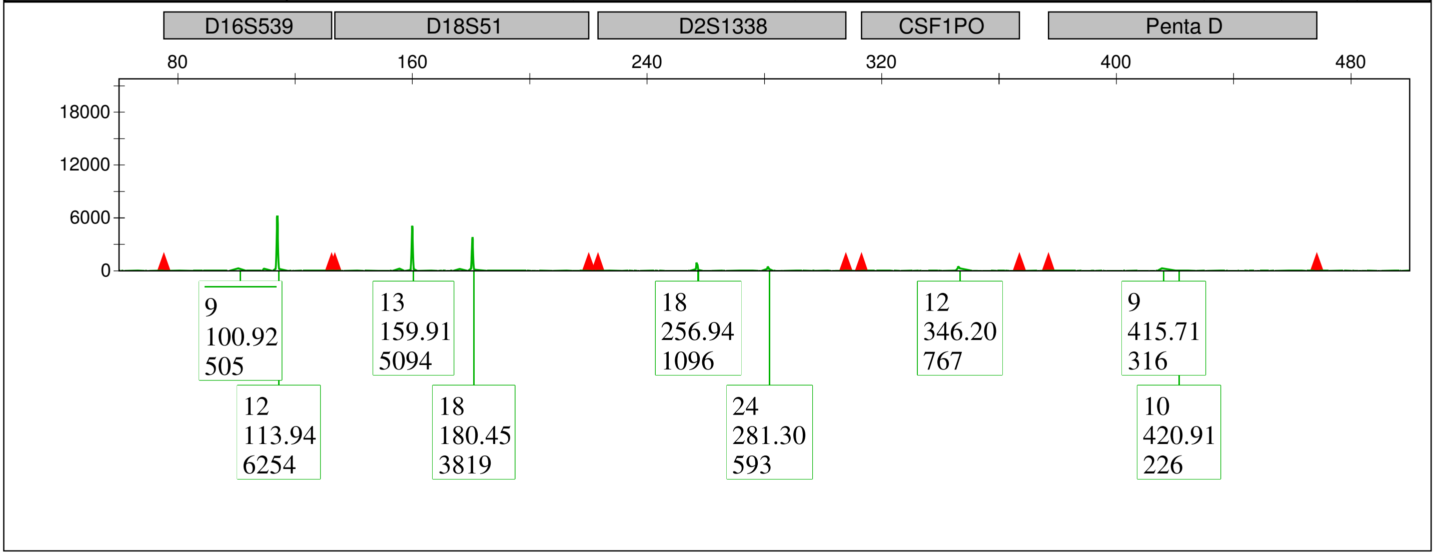

Sample File	Sample Name	Panel	SQI	OS	SQ
H7_B4 KD 19 1239 A9_Sample_20210126_173631.fsa	B4 KD 19 1239 A9	PowerPlex_21_POP1_Panels_vSS2.0			

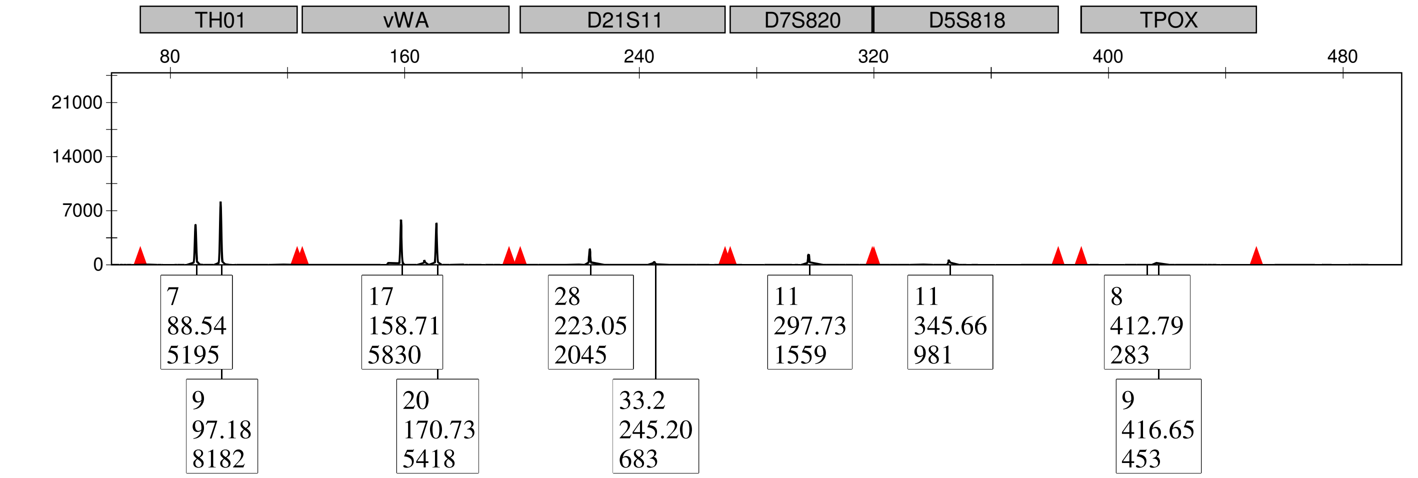

H7_B4 KD 19 1239 A9_Sample_20210126_173631.fsa	B4 KD 19 1239 A9	PowerPlex_21_POP1_Panels_vSS2.0			

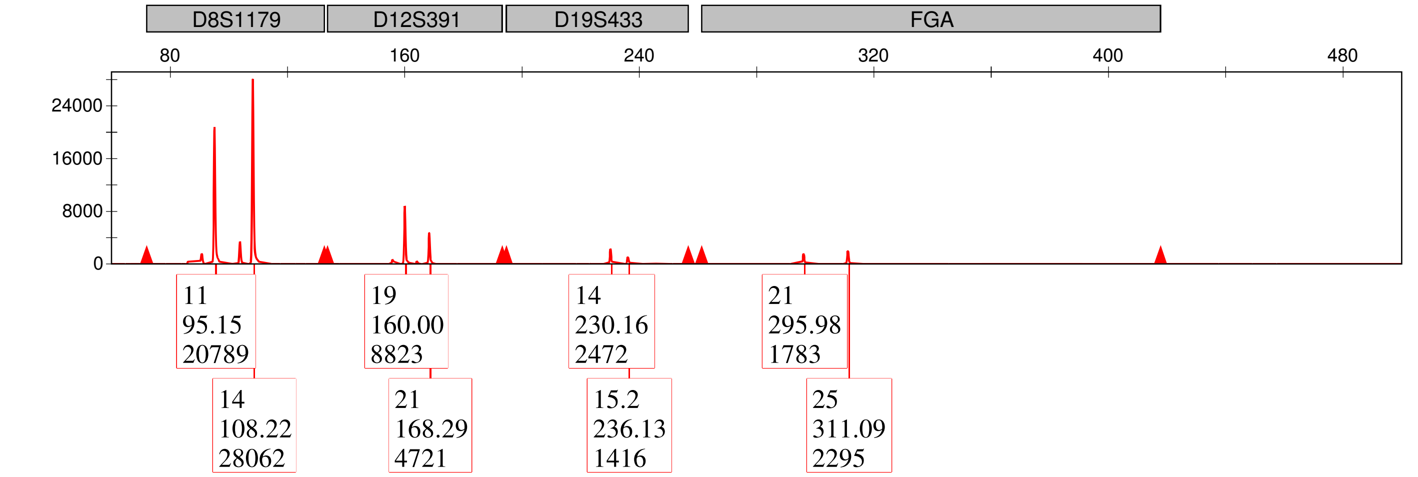

Applied Biosystems
GeneMapper Software 5

HM 20210127

Sample File	Sample Name	Panel	SQI	OS	SQ
A8_A5 NBL 30 0719_Sample_20210126_181924.fsa	A5 NBL 30 0719	PowerPlex_21_POP1_Panels_vSS2.0			

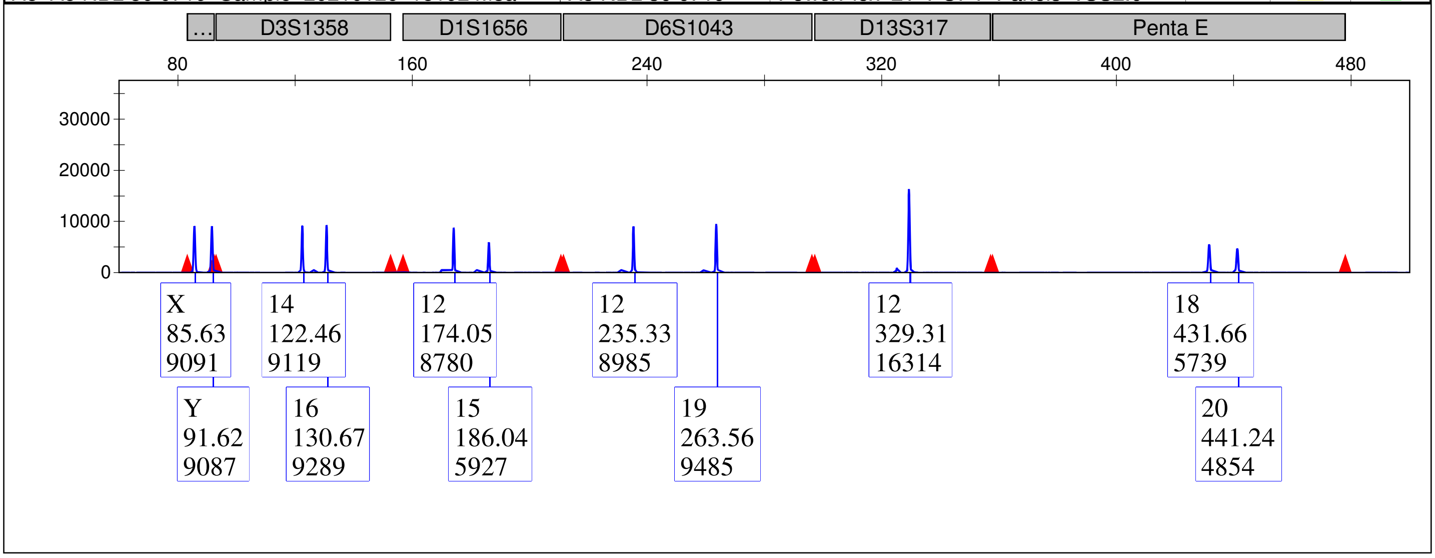

Sample File	Sample Name	Panel	SQI	OS	SQ
A8_A5 NBL 30 0719_Sample_20210126_181924.fsa	A5 NBL 30 0719	PowerPlex_21_POP1_Panels_vSS2.0			

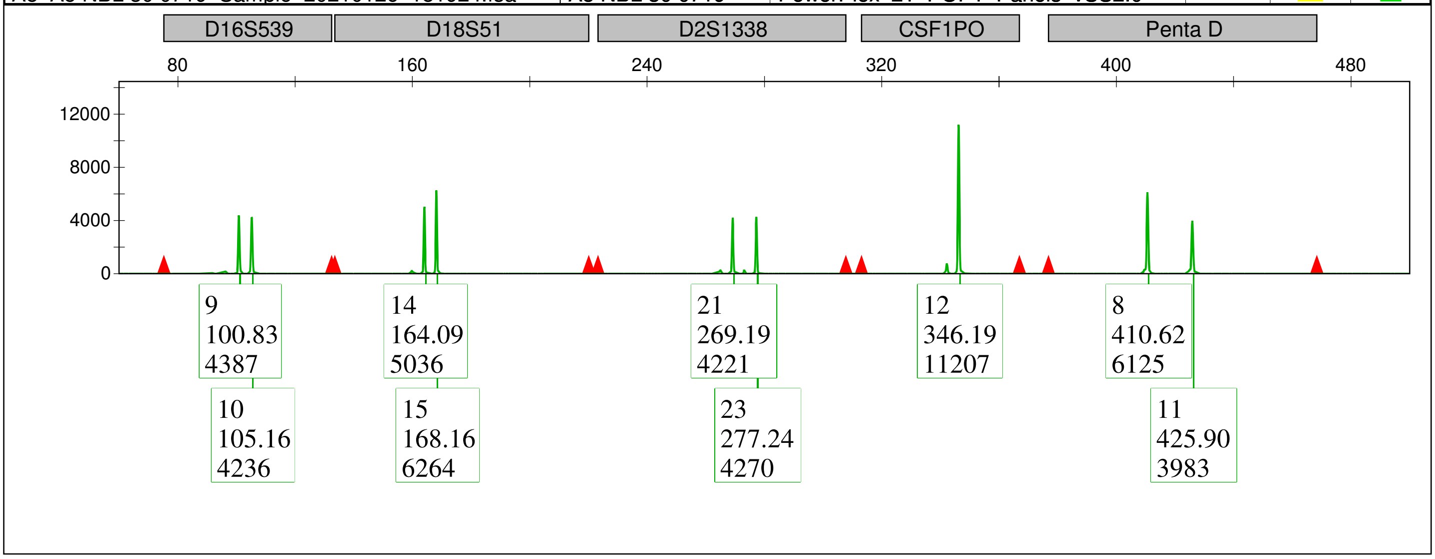

Sample File	Sample Name	Panel	SQI	OS	SQ
A8_A5 NBL 30 0719_Sample_20210126_181924.fsa	A5 NBL 30 0719	PowerPlex_21_POP1_Panels_vSS2.0			

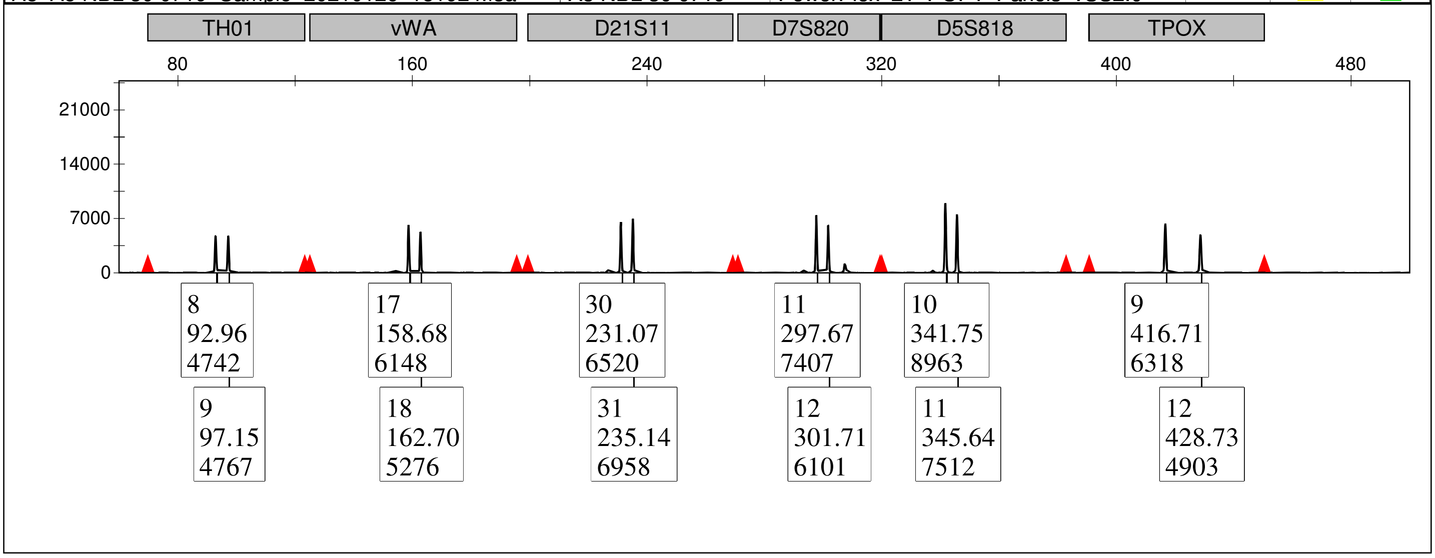

Sample File	Sample Name	Panel	SQI	OS	SQ
A8_A5 NBL 30 0719_Sample_20210126_181924.fsa	A5 NBL 30 0719	PowerPlex_21_POP1_Panels_vSS2.0			

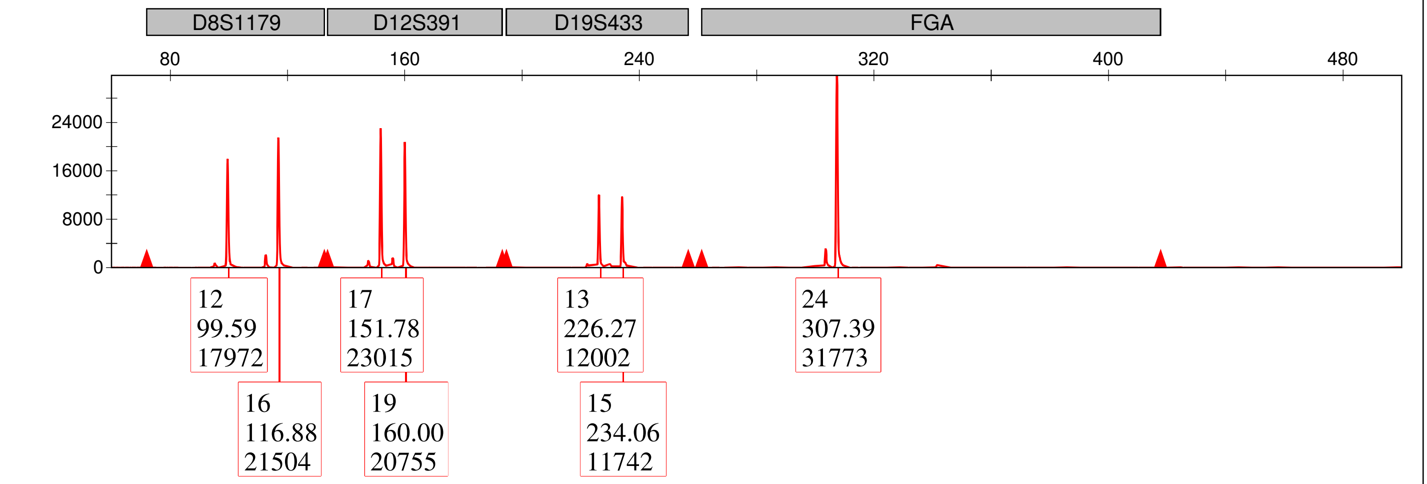

B8_B5 KD 19 1428_Sample_20210126_181925.fsa	B5 KD 19 1428	PowerPlex_21_POP1_Panels_vSS2.0			

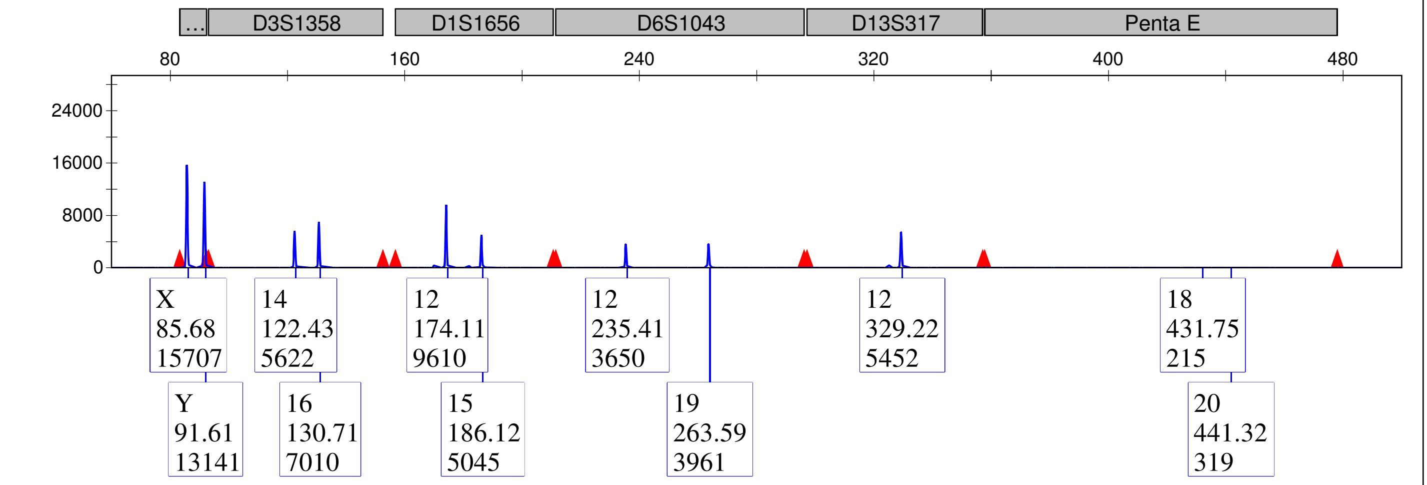

B8_B5 KD 19 1428_Sample_20210126_181925.fsa	B5 KD 19 1428	PowerPlex_21_POP1_Panels_vSS2.0			

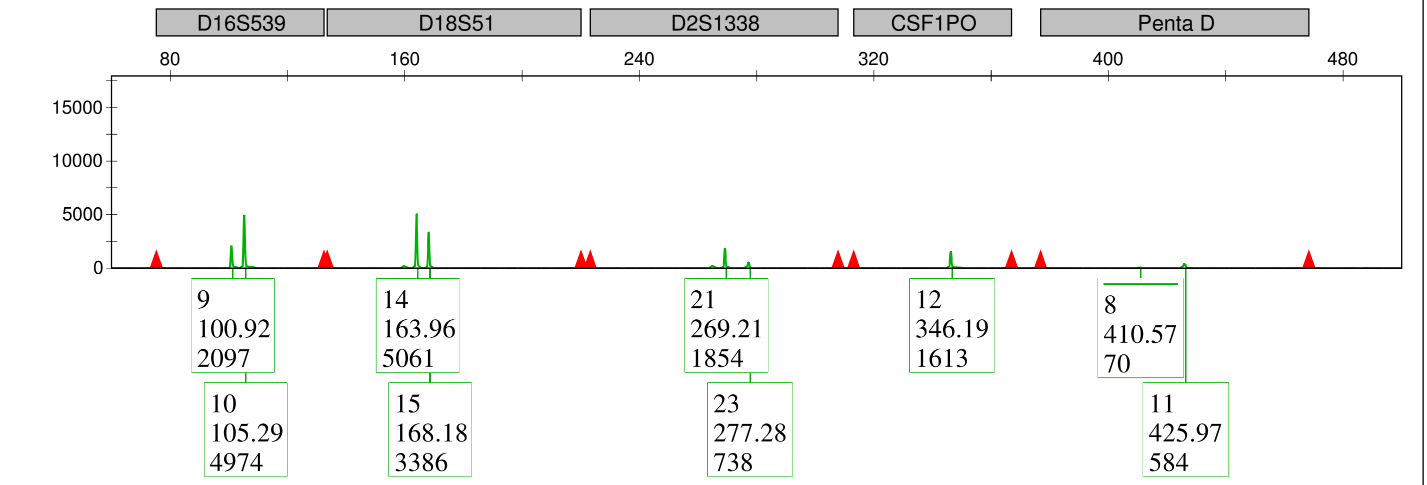

GeneMapper Software 5

HM 20210127

Sample File	Sample Name	Panel	SQI	OS	SQ
B8 B5 KD 19 1428 Sample 20210126 181925.fsa	B5 KD 19 1428	PowerPlex 21 POP1 Panels vSS2.0			

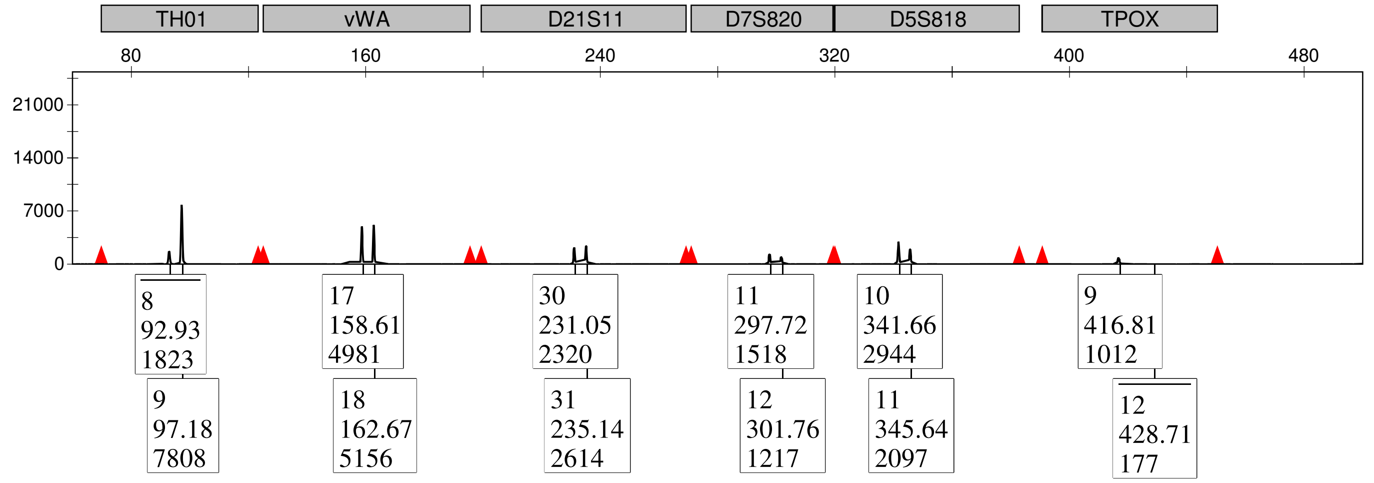

Sample File	Sample Name	Panel	SQI	OS	SQ
B8 B5 KD 19 1428 Sample 20210126 181925.fsa	B5 KD 19 1428	PowerPlex 21 POP1 Panels vSS2.0			

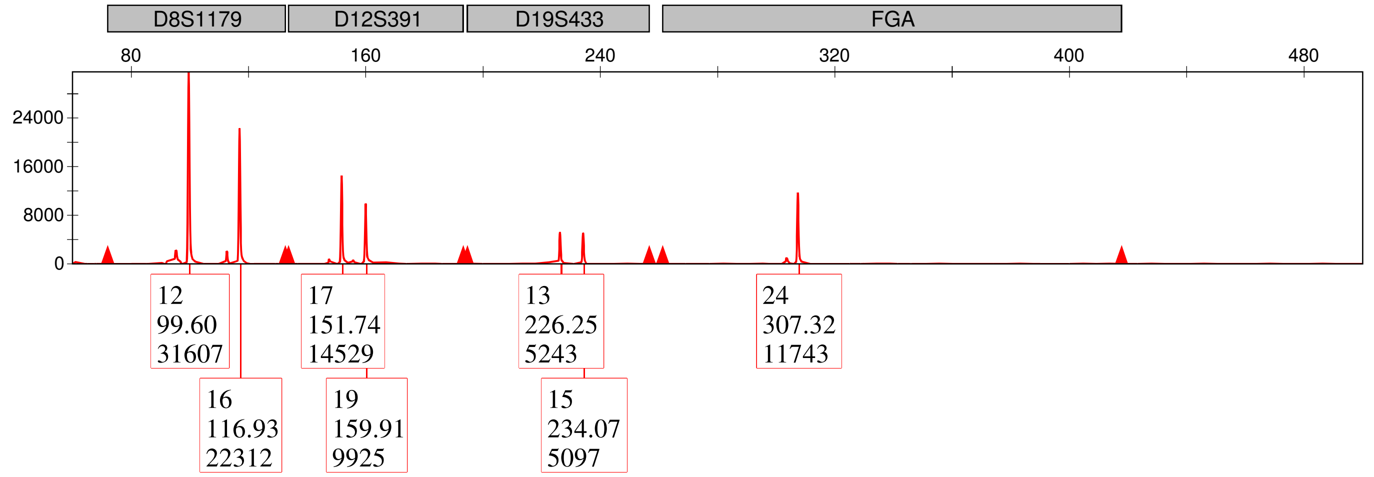

Applied Biosystems
GeneMapper Software 5

HM 20210127

Sample File	Sample Name	Panel	SQI	OS	SQ
C9_A6 NBL 03 1019_Sample_20210126_194534.fsa	A6 NBL 03 1019	PowerPlex_21_POP1_Panels_vSS2.0			

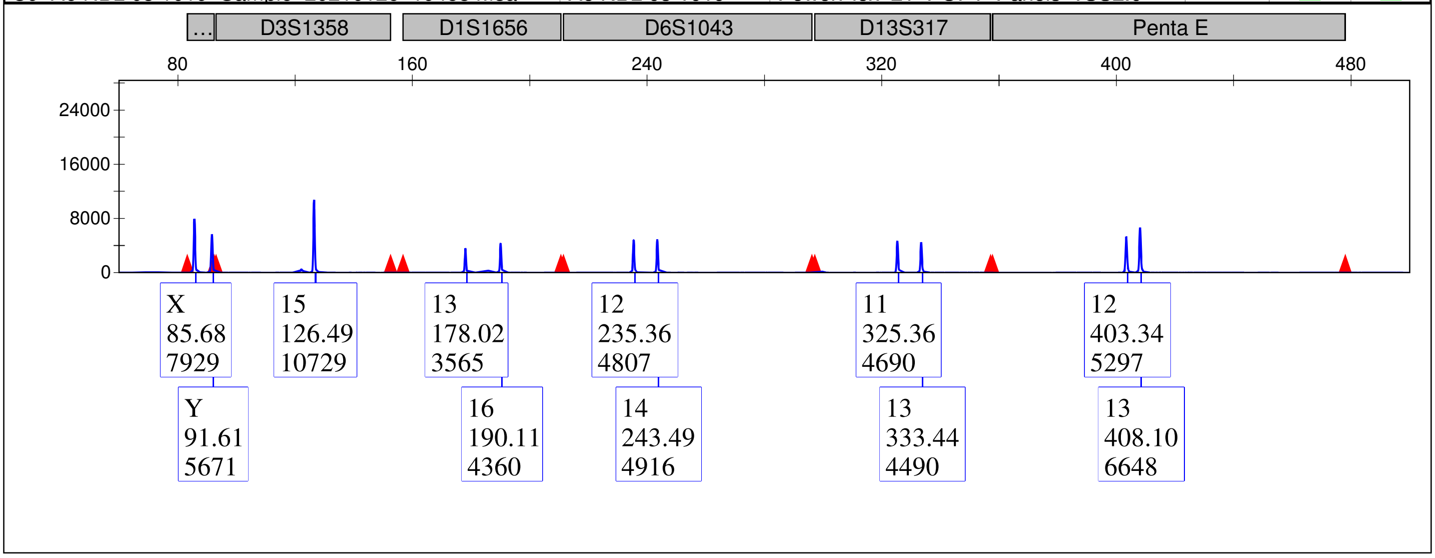

C9_A6 NBL 03 1019_Sample_20210126_194534.fsa	A6 NBL 03 1019	PowerPlex_21_POP1_Panels_vSS2.0			

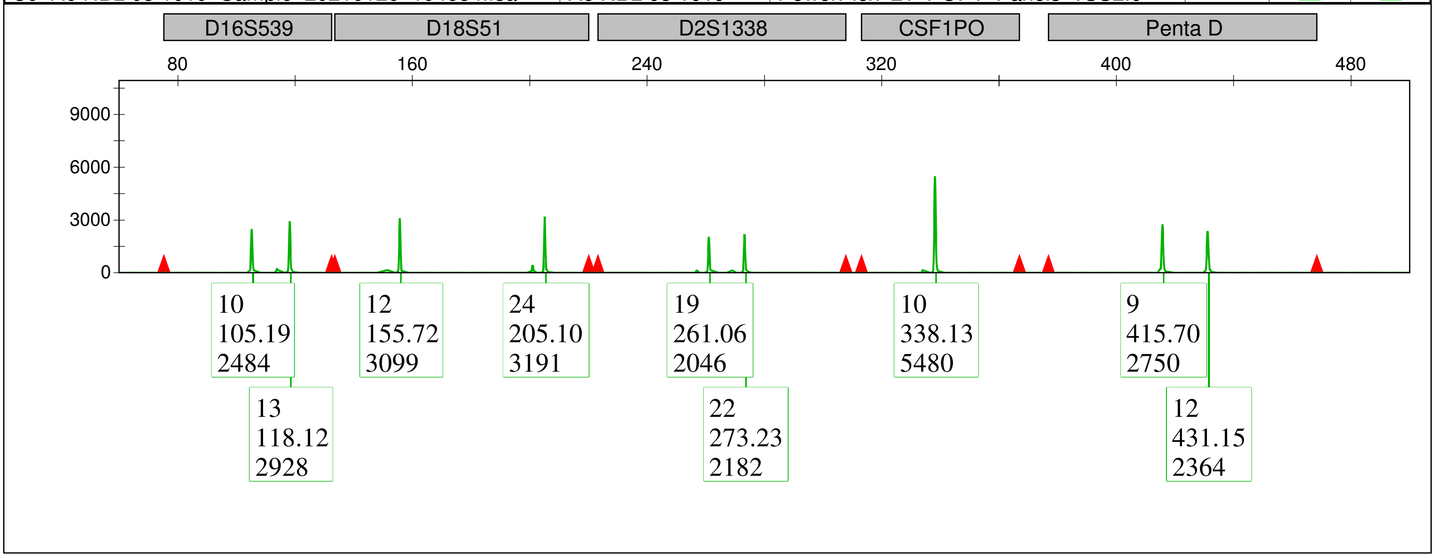

C9_A6 NBL 03 1019_Sample_20210126_194534.fsa	A6 NBL 03 1019	PowerPlex_21_POP1_Panels_vSS2.0			

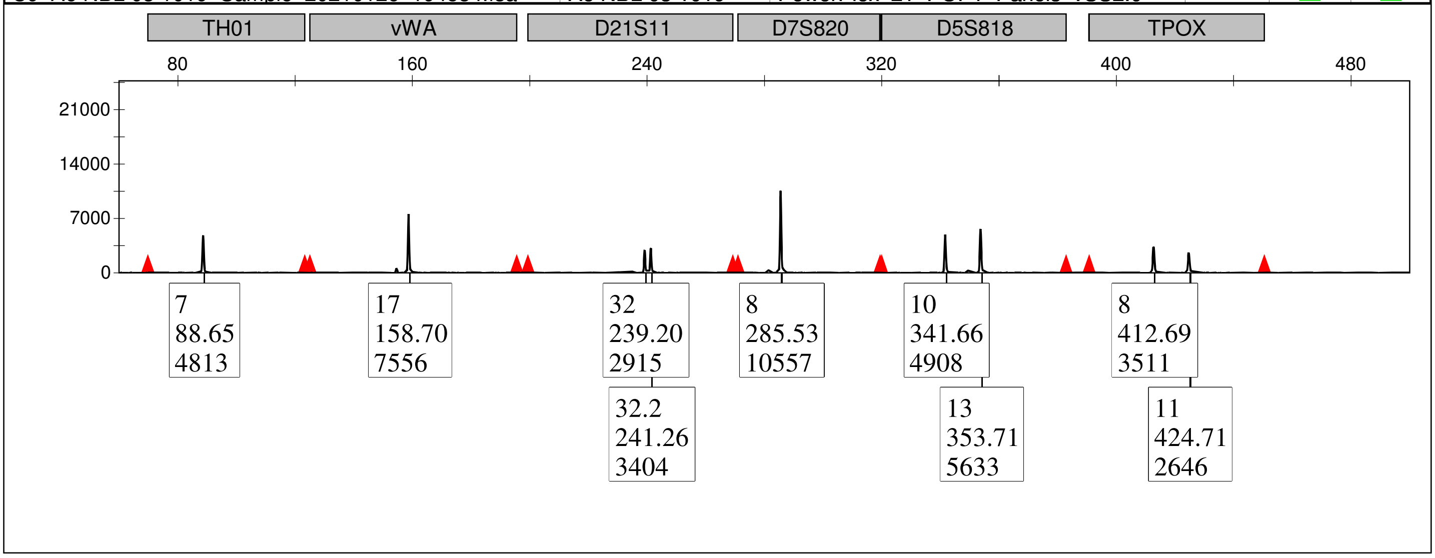

Applied Biosystems
GeneMapper Software 5

HM 20210127

Sample File	Sample Name	Panel	SQI	OS	SQ
C9_A6 NBL 03 1019_Sample_20210126_194534.fsa	A6 NBL 03 1019	PowerPlex_21_POP1_Panels_vSS2.0			

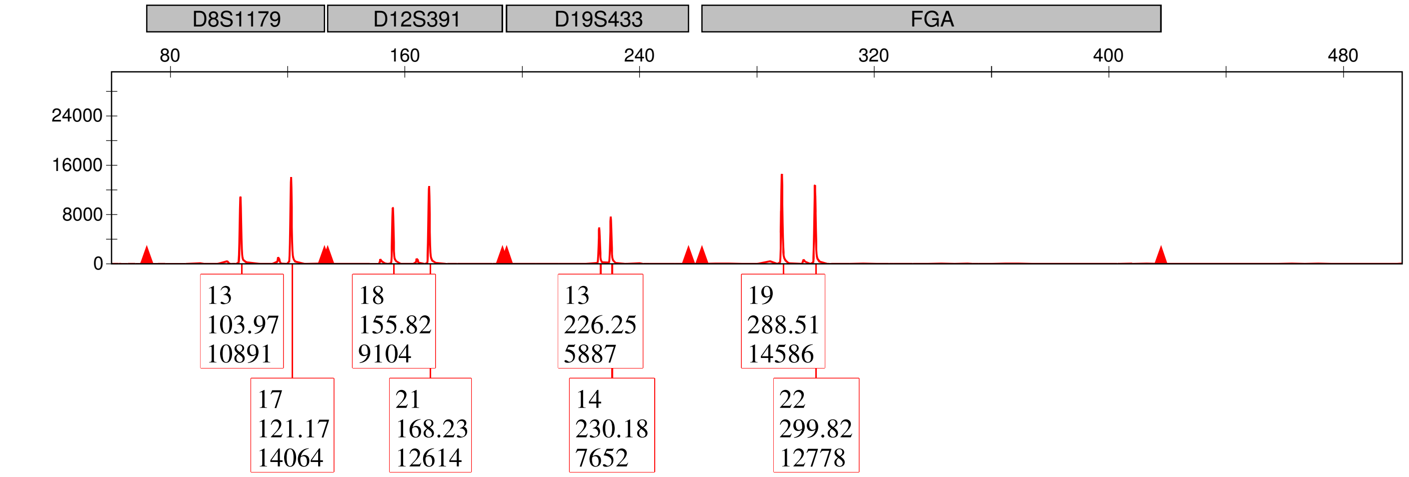

Sample File	Sample Name	Panel	SQI	OS	SQ
D8_B6 KD 19 1830A2_Sample_20210126_181927.fsa	B6 KD 19 1830A2	PowerPlex_21_POP1_Panels_vSS2.0			

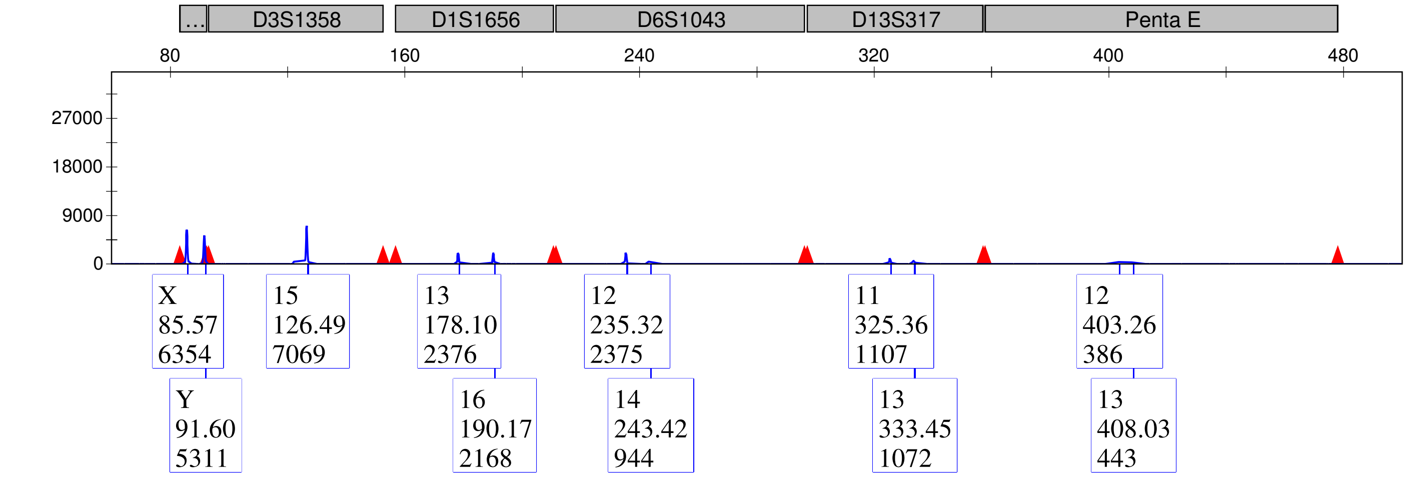

Sample File	Sample Name	Panel	SQI	OS	SQ
D8_B6 KD 19 1830A2_Sample_20210126_181927.fsa	B6 KD 19 1830A2	PowerPlex_21_POP1_Panels_vSS2.0			

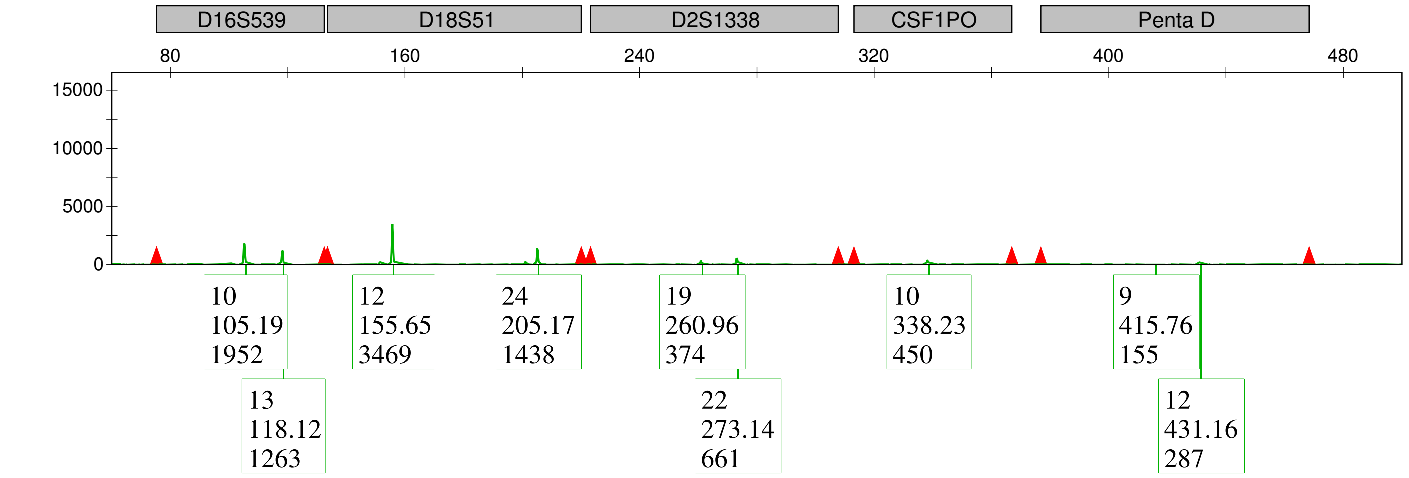

Applied Biosystems
GeneMapper Software 5

HM 20210127

Sample File	Sample Name	Panel	SQI	OS	SQ
D8_B6 KD 19 1830A2_Sample_20210126_181927.fsa	B6 KD 19 1830A2	PowerPlex_21_POP1_Panels_vSS2.0			

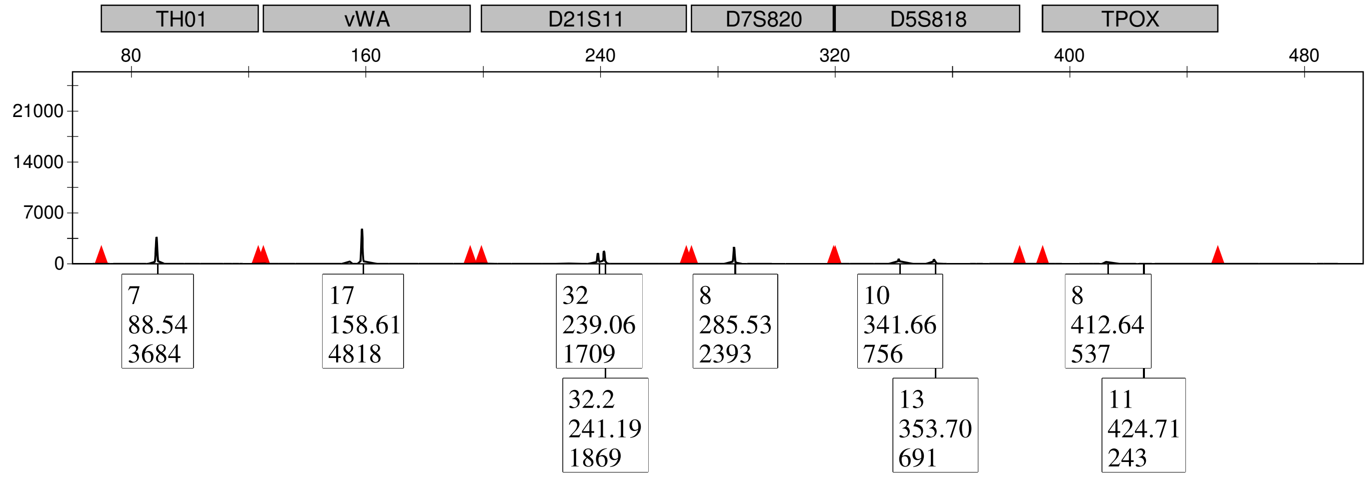

Sample File	Sample Name	Panel	SQI	OS	SQ
D8_B6 KD 19 1830A2_Sample_20210126_181927.fsa	B6 KD 19 1830A2	PowerPlex_21_POP1_Panels_vSS2.0			

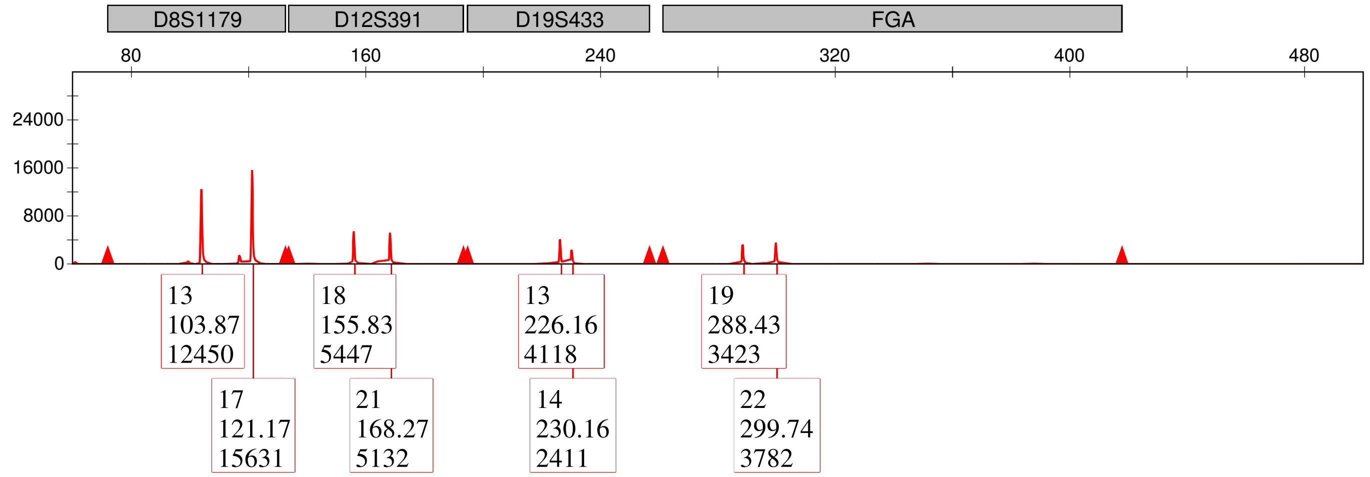

Sample File	Sample Name	Panel	SQI	OS	SQ
E8_A7 NBL 29 1019_Sample_20210126_190231.fsa	A7 NBL 29 1019	PowerPlex_21_POP1_Panels_vSS2.0			

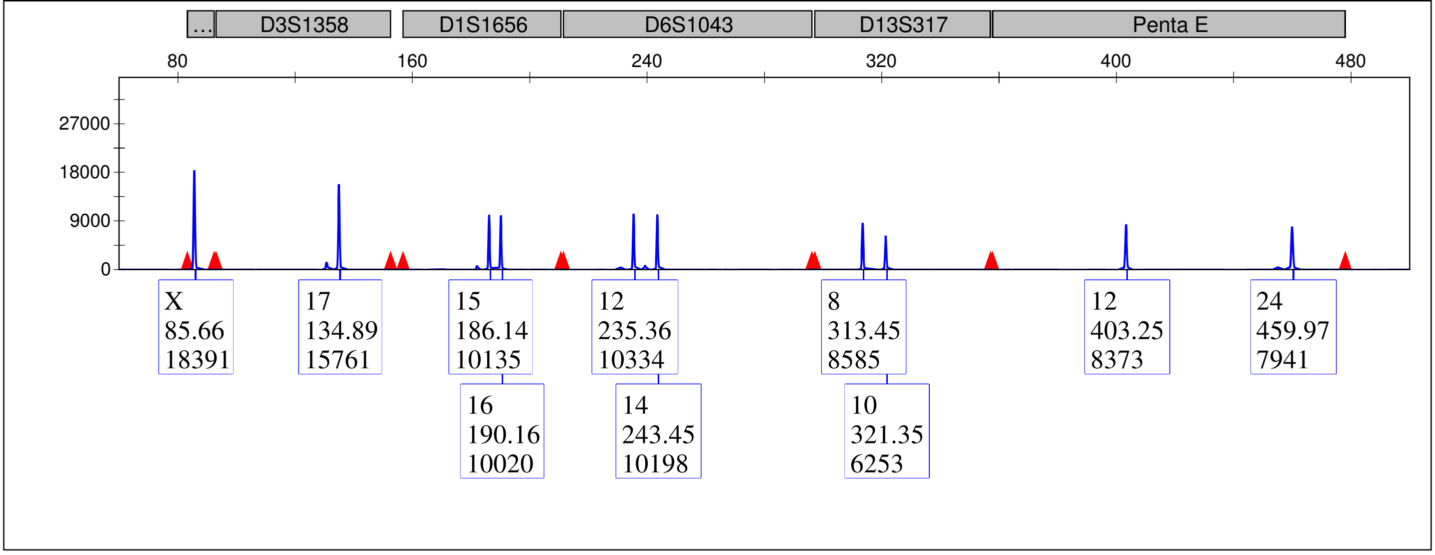

E8_A7 NBL 29 1019_Sample_20210126_190231.fsa	A7 NBL 29 1019	PowerPlex_21_POP1_Panels_vSS2.0			

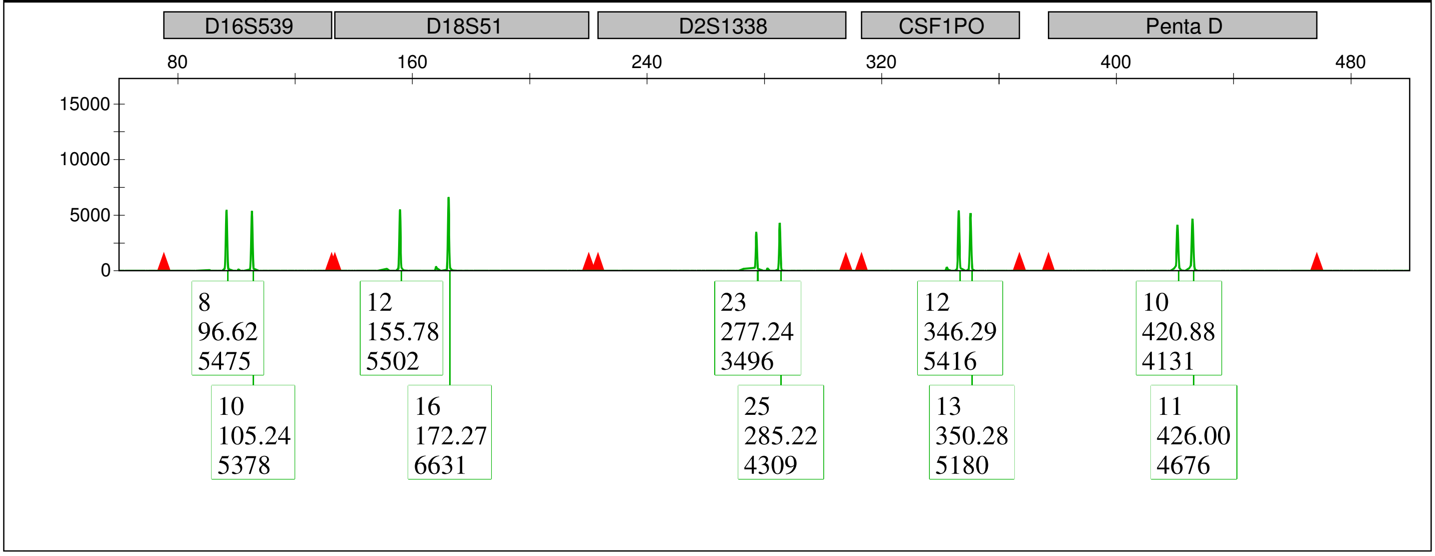

E8_A7 NBL 29 1019_Sample_20210126_190231.fsa	A7 NBL 29 1019	PowerPlex_21_POP1_Panels_vSS2.0			

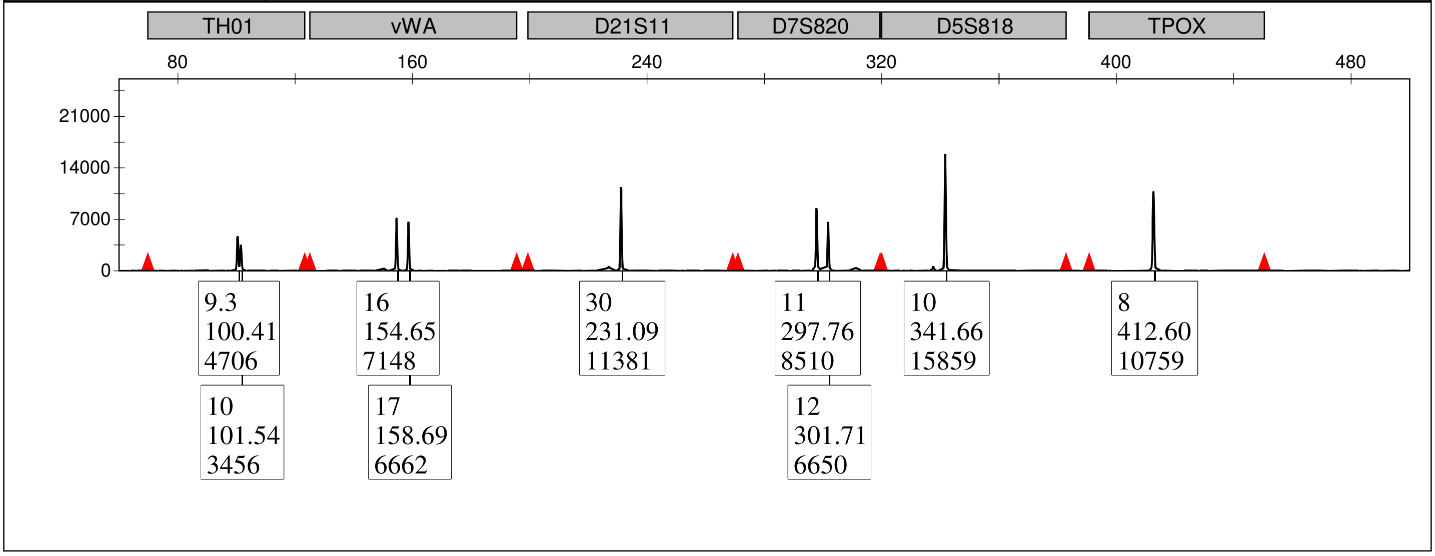

Sample File	Sample Name	Panel	SQI	OS	SQ
E8 A7 NBL 29 1019 Sample 20210126 190231.fsa	A7 NBL 29 1019	PowerPlex 21 POP1 Panels vSS2.0			

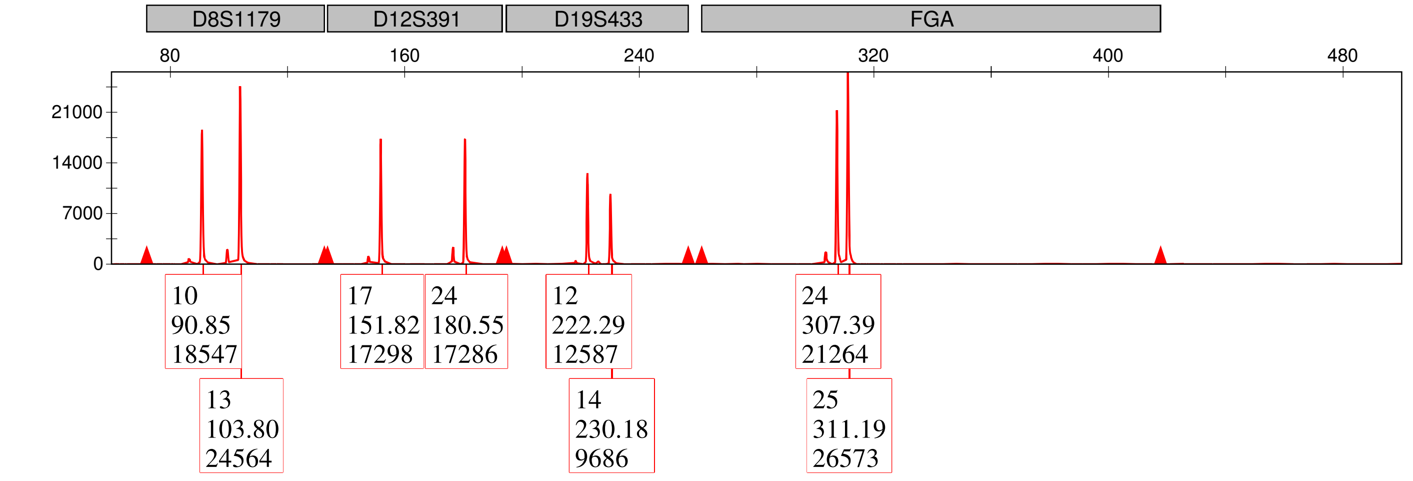

Sample File	Sample Name	Panel	SQI	OS	SQ
F8 B7 KD 19 1978A2 Sample 20210126 190232.fsa	B7 KD 19 1978A2	PowerPlex 21 POP1 Panels vSS2.0			

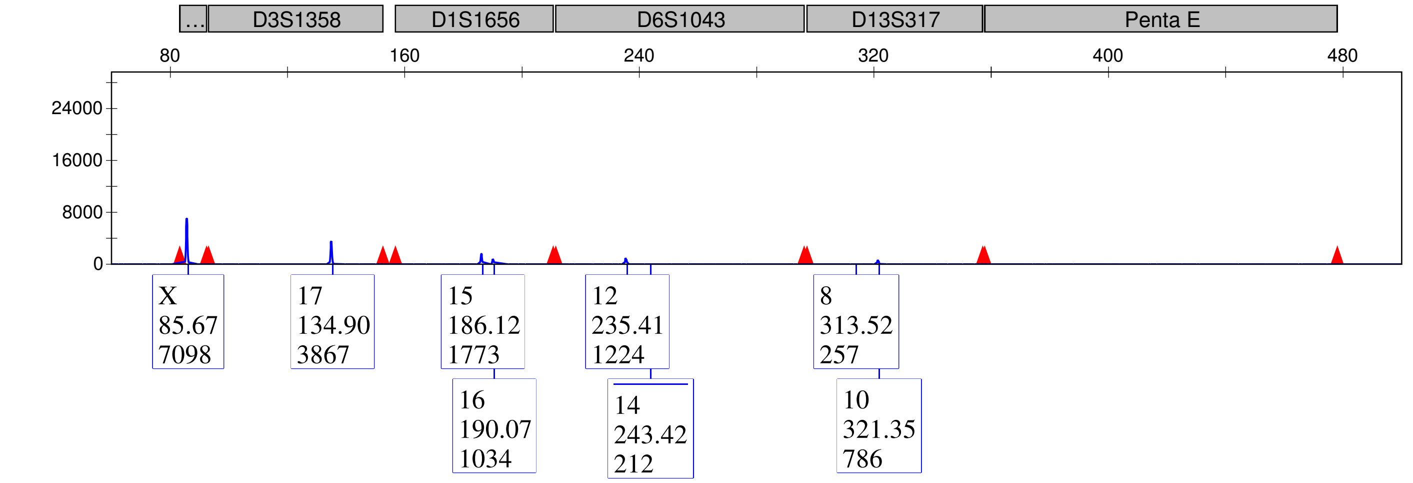

Sample File	Sample Name	Panel	SQI	OS	SQ
F8 B7 KD 19 1978A2 Sample 20210126 190232.fsa	B7 KD 19 1978A2	PowerPlex 21 POP1 Panels vSS2.0			

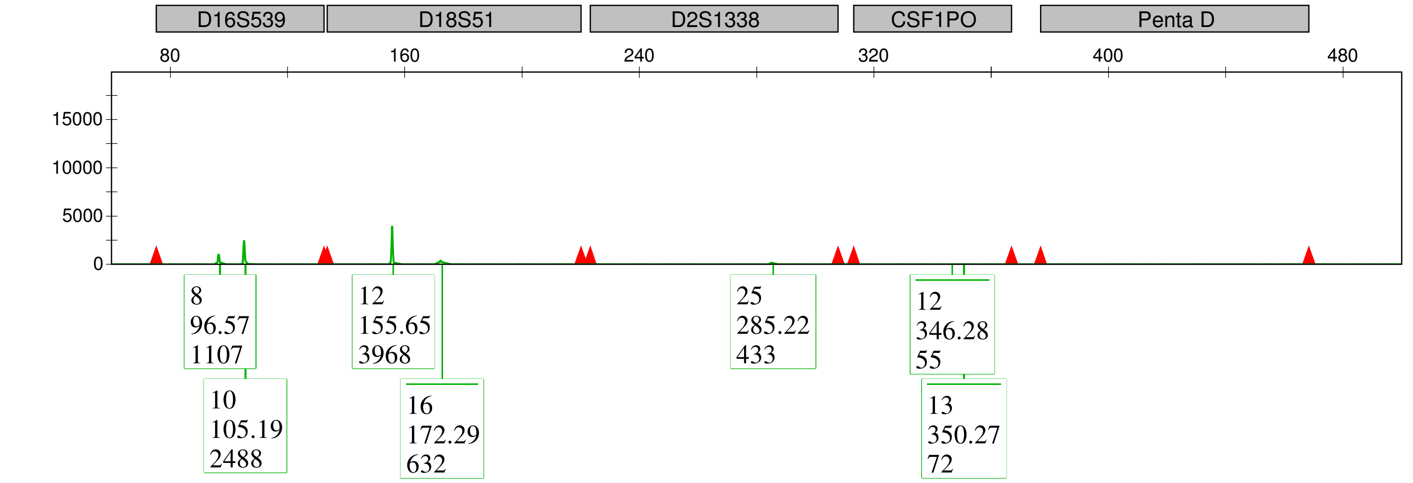

Sample File	Sample Name	Panel	SQI	OS	SQ
F8_B7 KD 19 1978A2_Sample_20210126_190232.fsa	B7 KD 19 1978A2	PowerPlex_21_POP1_Panels_vSS2.0			

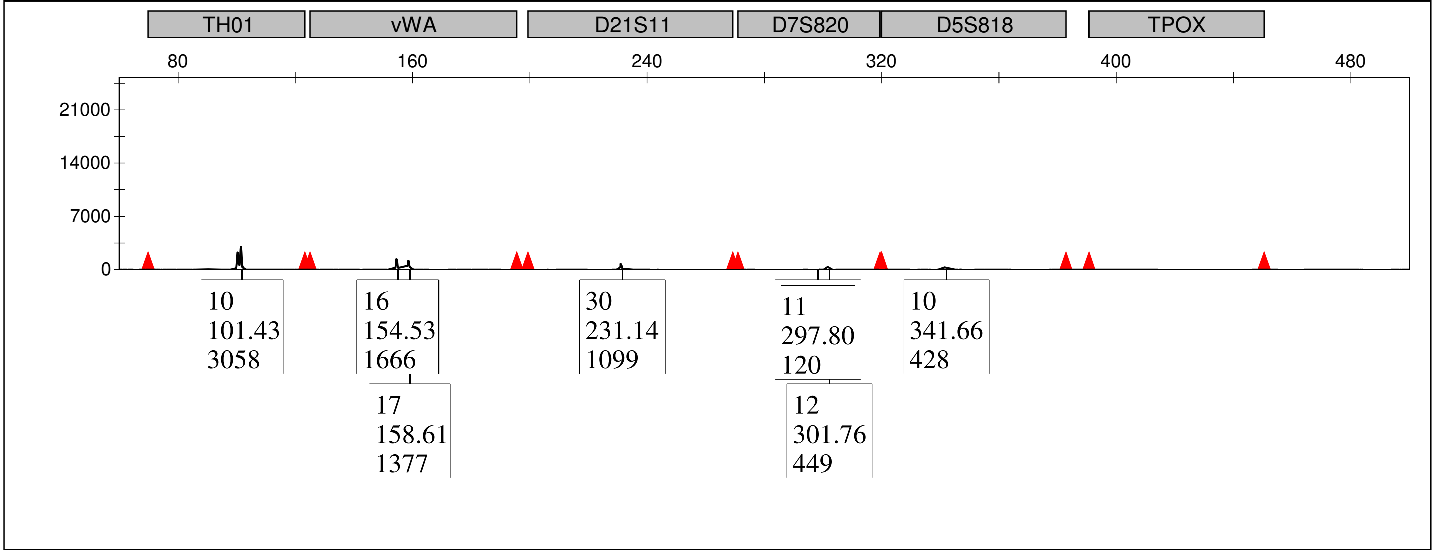

F8_B7 KD 19 1978A2_Sample_20210126_190232.fsa	B7 KD 19 1978A2	PowerPlex_21_POP1_Panels_vSS2.0			

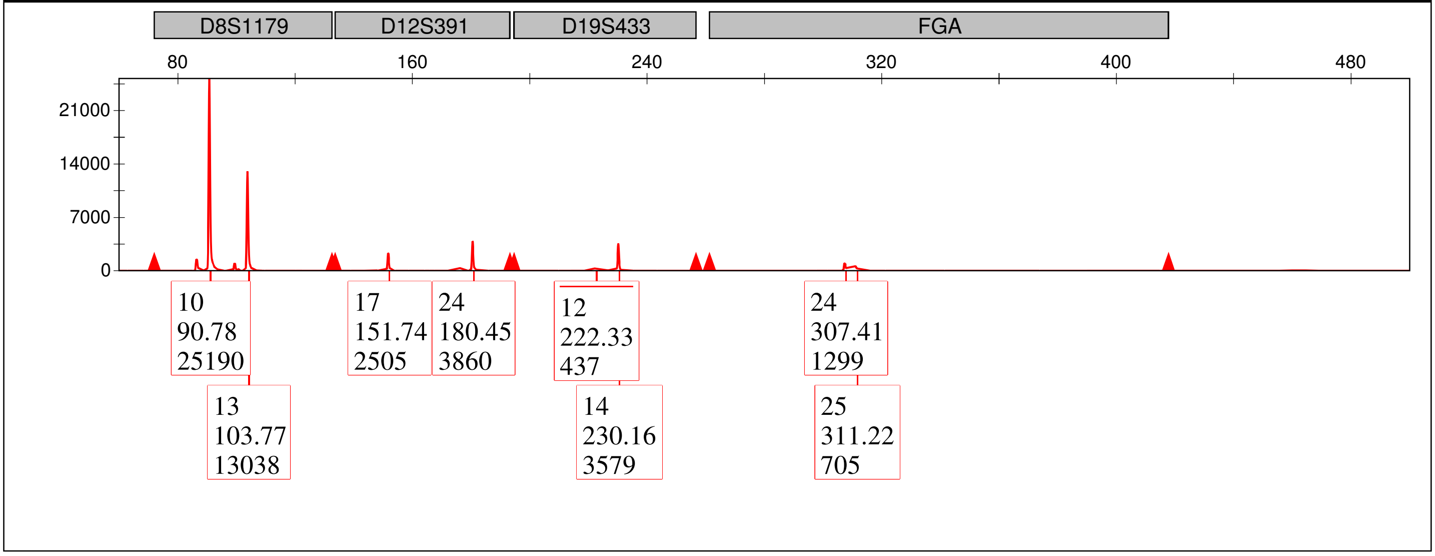

Applied Biosystems
GeneMapper Software 5

HM 20210127

Sample File	Sample Name	Panel	SQI	OS	SQ
G8_A8 NBL 03 1219A_Sample_20210126_190233.fsa	A8 NBL 03 1219A	PowerPlex_21_POP1_Panels_vSS2.0			

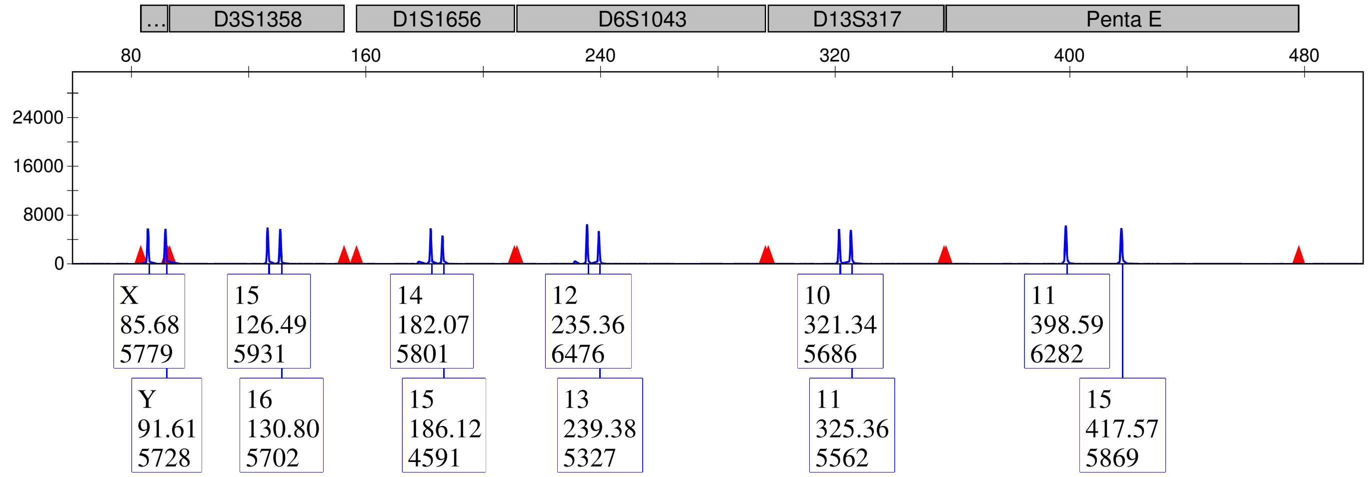

G8_A8 NBL 03 1219A_Sample_20210126_190233.fsa | A8 NBL 03 1219A | PowerPlex_21_POP1_Panels_vSS2.0

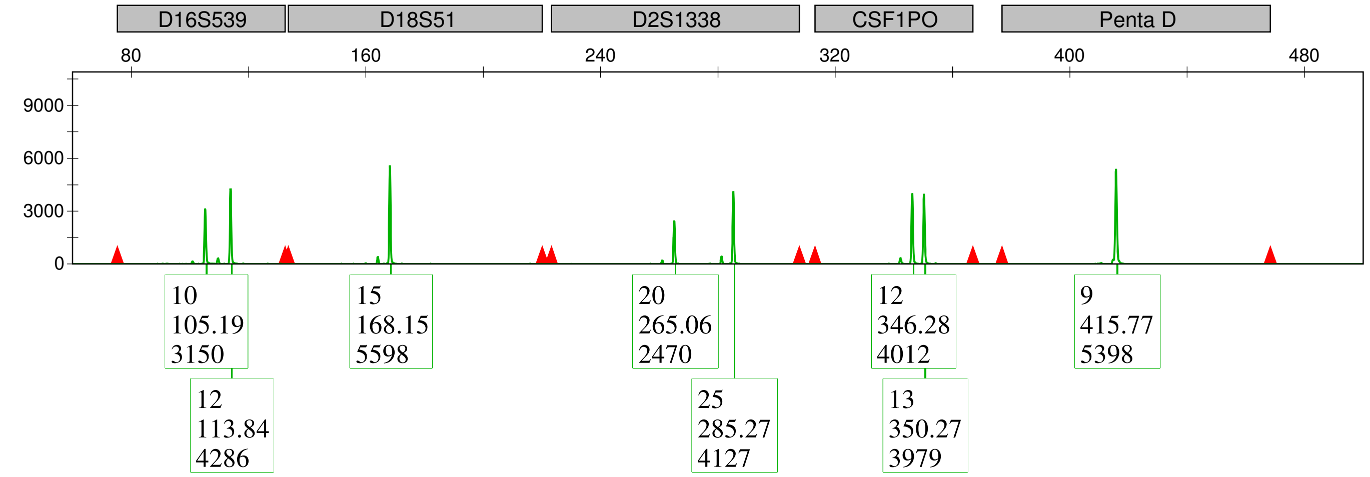

G8_A8 NBL 03 1219A_Sample_20210126_190233.fsa | A8 NBL 03 1219A | PowerPlex_21_POP1_Panels_vSS2.0

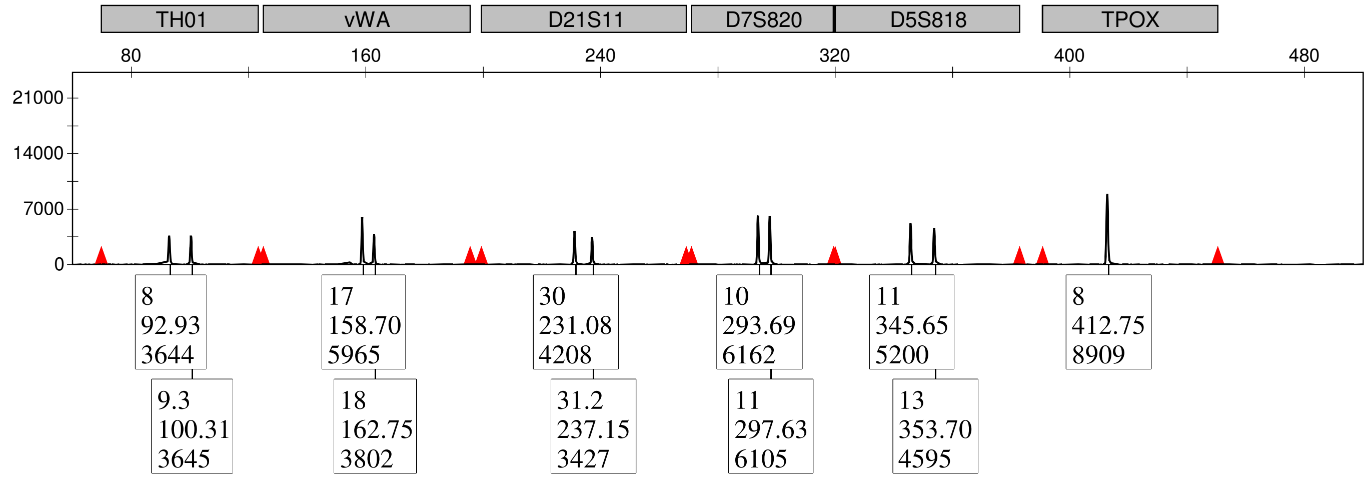

Applied Biosystems
GeneMapper Software 5

HM 20210127

Sample File	Sample Name	Panel	SQI	OS	SQ
G8 A8 NBL 03 1219A Sample 20210126 190233.fsa	A8 NBL 03 1219A	PowerPlex 21 POP1 Panels vSS2.0			

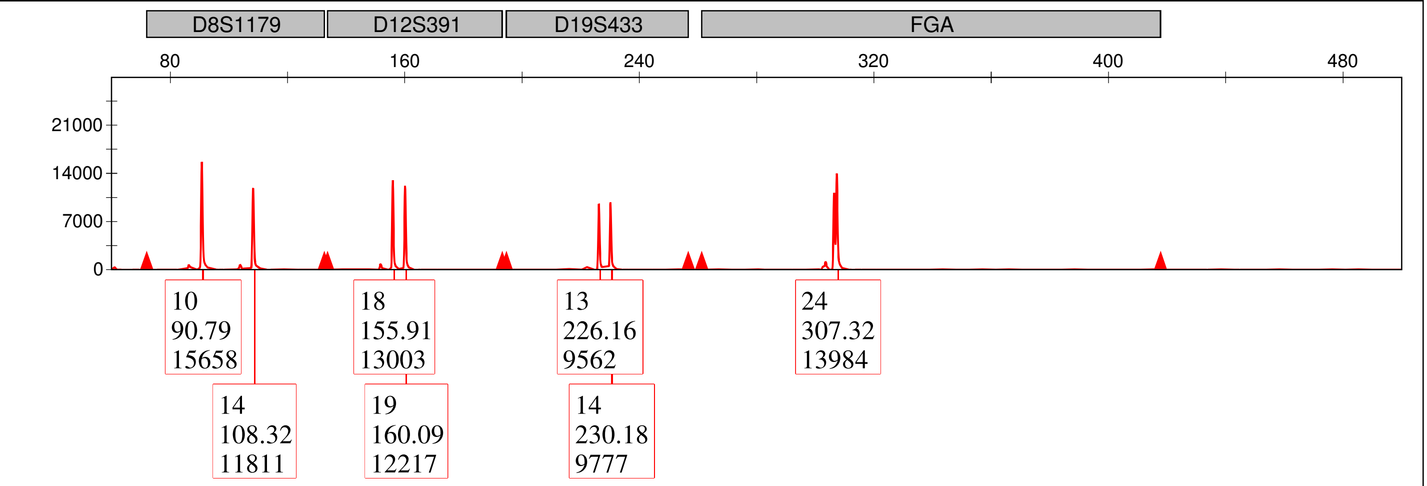

Sample File	Sample Name	Panel	SQI	OS	SQ
H8 B8 KD 19 2210A3 Sample 20210126 190234.fsa	B8 KD 19 2210A3	PowerPlex 21 POP1 Panels vSS2.0			

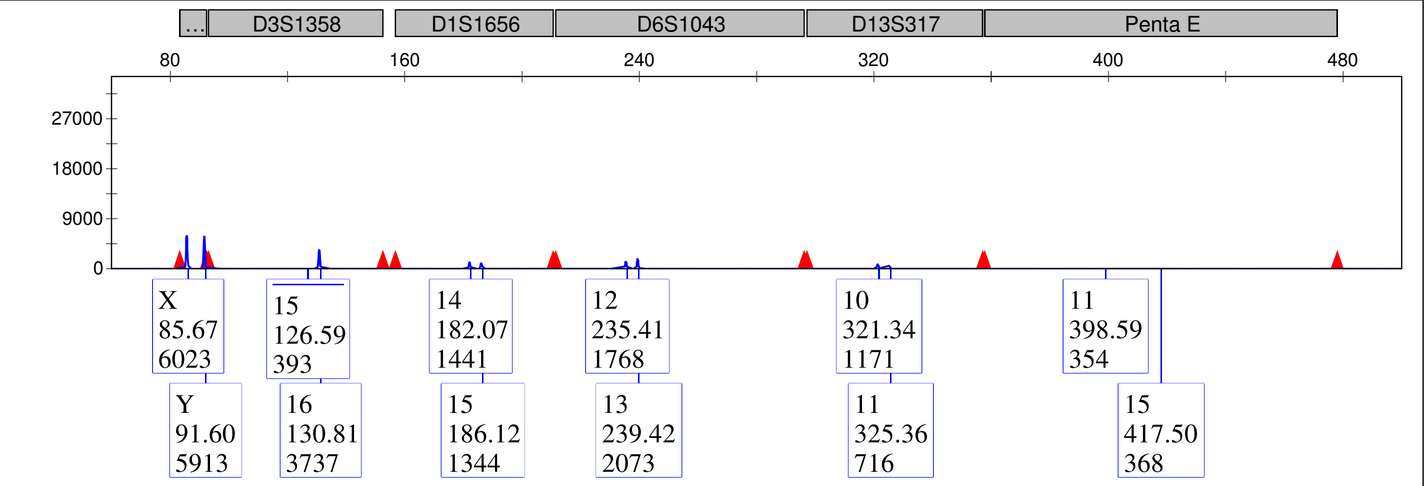

Sample File	Sample Name	Panel	SQI	OS	SQ
H8 B8 KD 19 2210A3 Sample 20210126 190234.fsa	B8 KD 19 2210A3	PowerPlex 21 POP1 Panels vSS2.0			

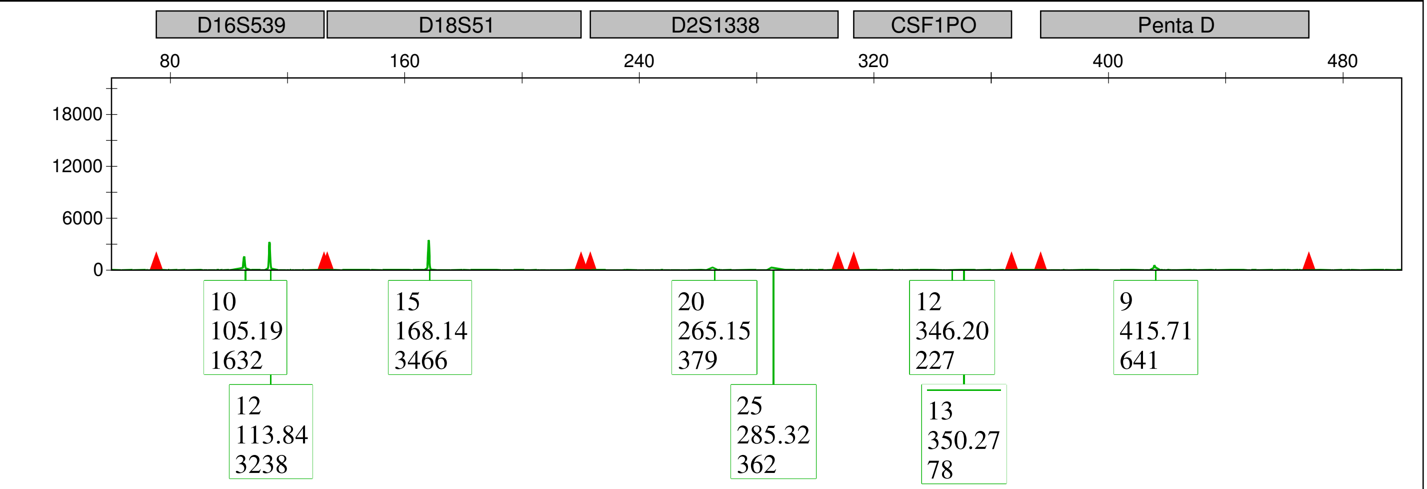

Applied Biosystems
GeneMapper Software 5

HM 20210127

Sample File	Sample Name	Panel	SQI	OS	SQ
H8_B8 KD 19 2210A3_Sample_20210126_190234.fsa	B8 KD 19 2210A3	PowerPlex_21_POP1_Panels_vSS2.0			

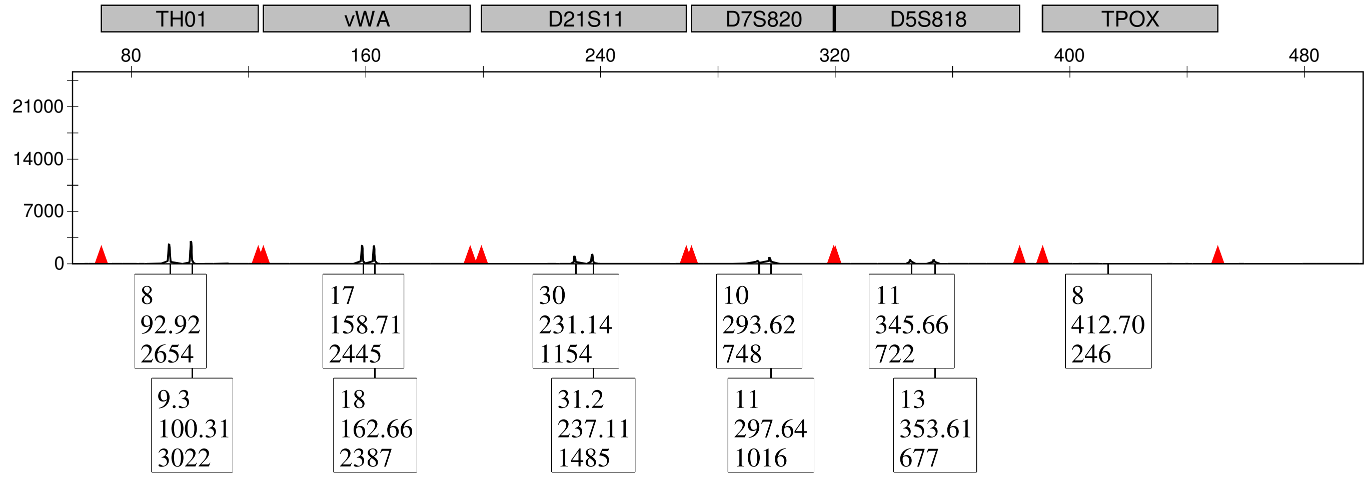

H8_B8 KD 19 2210A3_Sample_20210126_190234.fsa	B8 KD 19 2210A3	PowerPlex_21_POP1_Panels_vSS2.0			

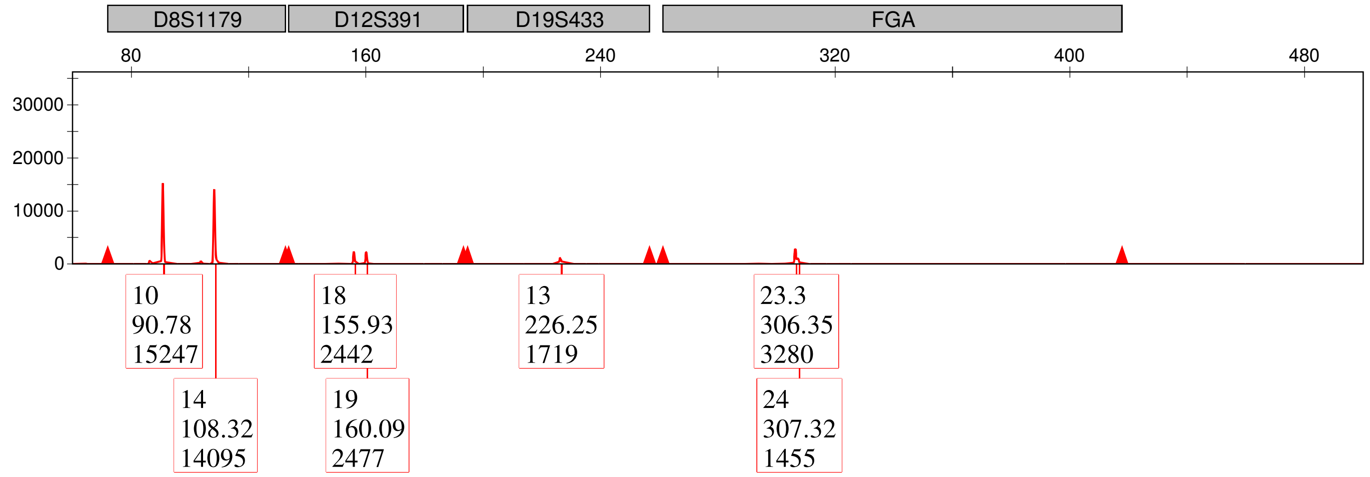

Sample File	Sample Name	Panel	SQI	OS	SQ
A9_A9 NBL 03 1219B_Sample_20210126_194532.fsa	A9 NBL 03 1219B	PowerPlex_21_POP1_Panels_vSS2.0			

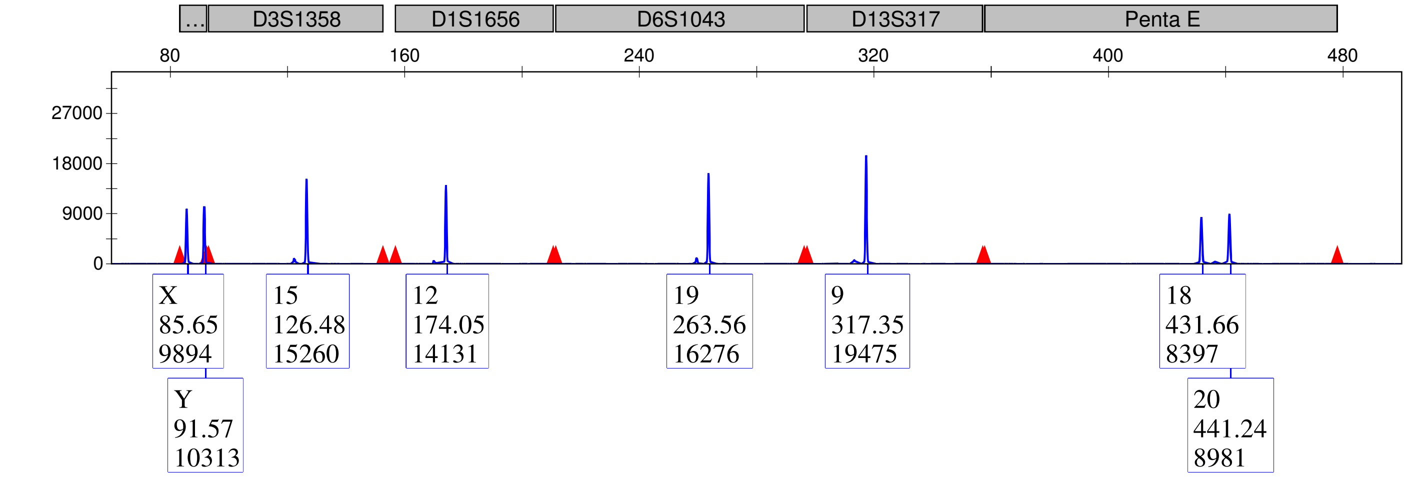

Sample File	Sample Name	Panel	SQI	OS	SQ
A9_A9 NBL 03 1219B_Sample_20210126_194532.fsa	A9 NBL 03 1219B	PowerPlex_21_POP1_Panels_vSS2.0			

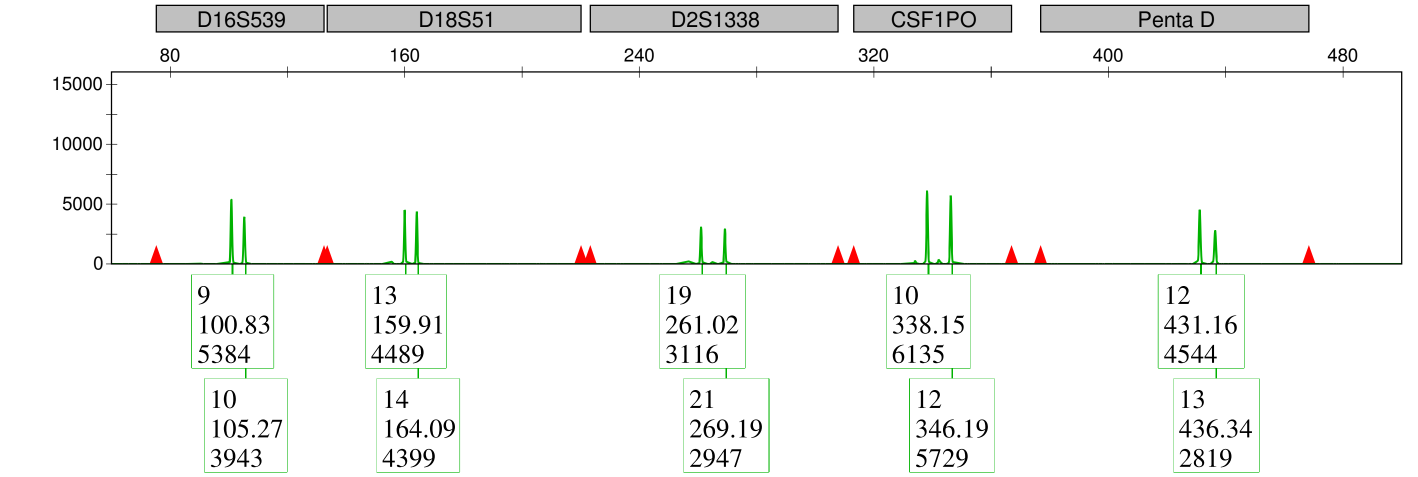

Sample File	Sample Name	Panel	SQI	OS	SQ
A9_A9 NBL 03 1219B_Sample_20210126_194532.fsa	A9 NBL 03 1219B	PowerPlex_21_POP1_Panels_vSS2.0			

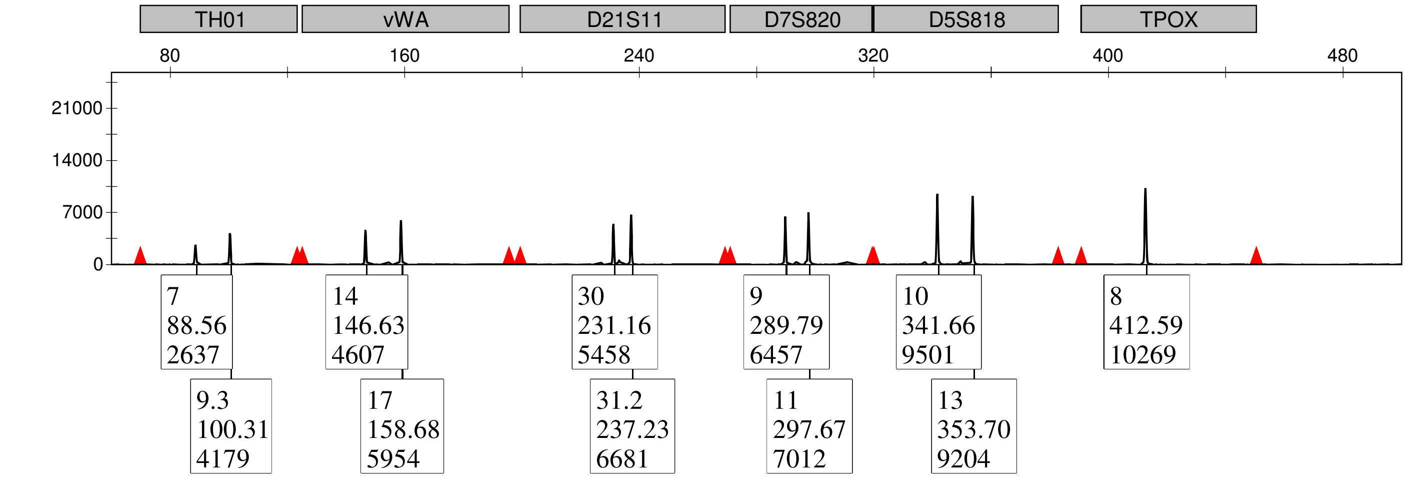

Sample File	Sample Name	Panel	SQI	OS	SQ
A9_A9 NBL 03 1219B_Sample_20210126_194532.fsa	A9 NBL 03 1219B	PowerPlex_21_POP1_Panels_vSS2.0			

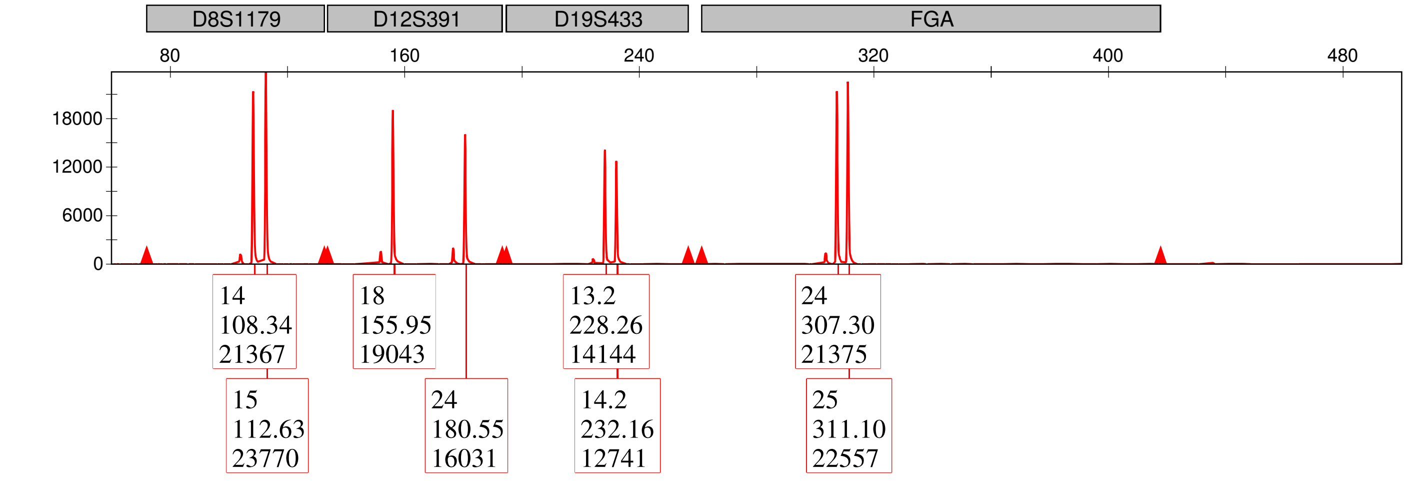

Sample File	Sample Name	Panel	SQI	OS	SQ
B9_B9 KD 19 2212 A4_Sample_20210126_194533.fsa	B9 KD 19 2212 A4	PowerPlex_21_POP1_Panels_vSS2.0			

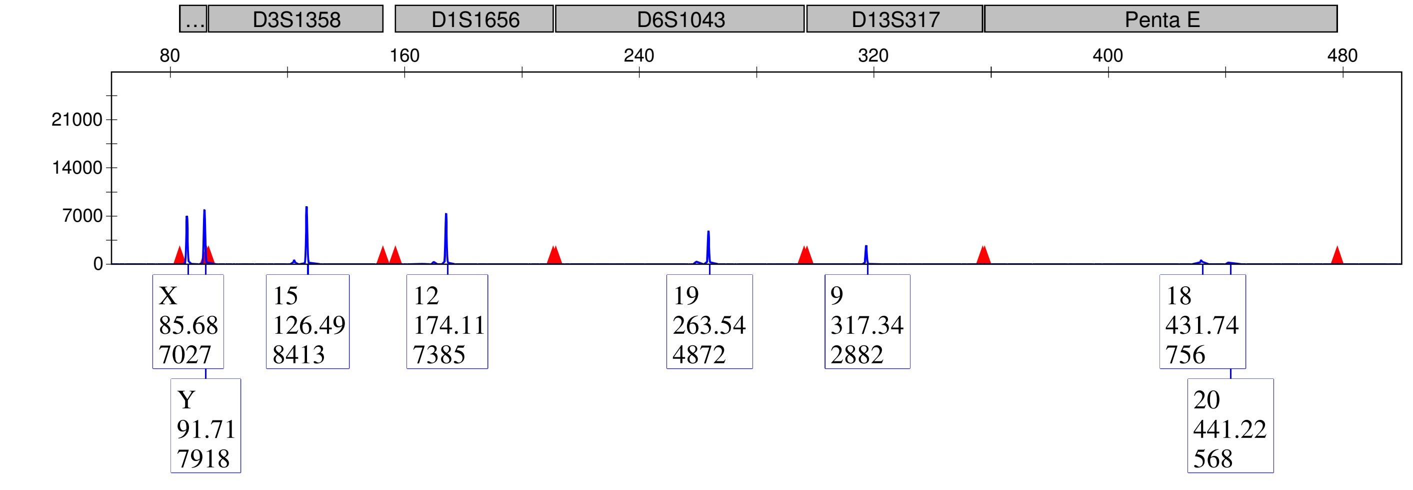

Sample File	Sample Name	Panel	SQI	OS	SQ
B9_B9 KD 19 2212 A4_Sample_20210126_194533.fsa	B9 KD 19 2212 A4	PowerPlex_21_POP1_Panels_vSS2.0			

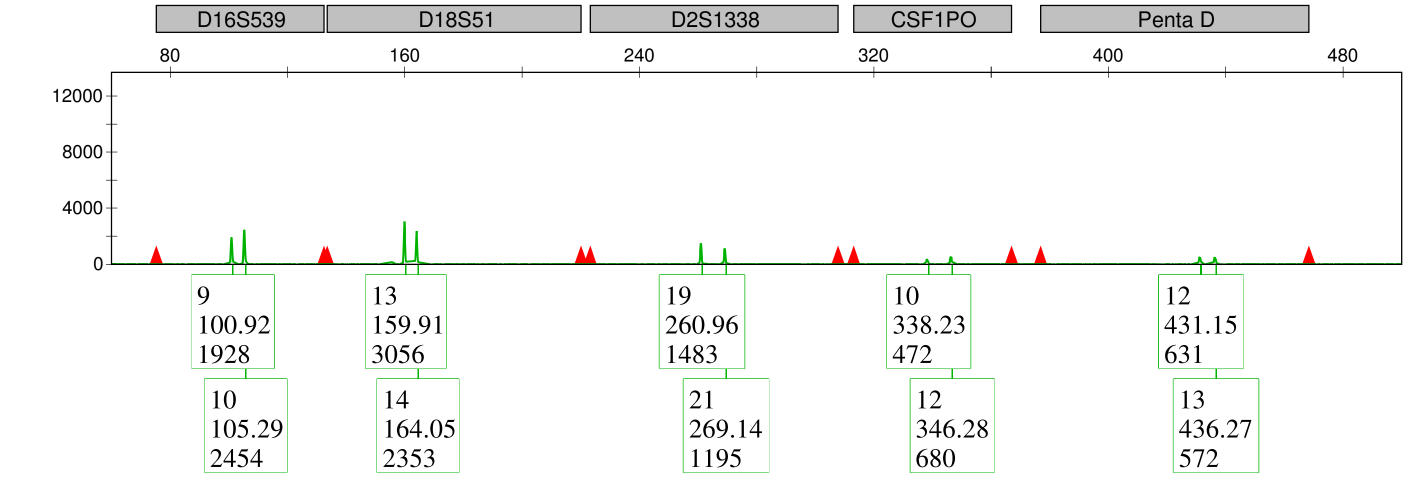

Sample File	Sample Name	Panel	SQI	OS	SQ
B9 B9 KD 19 2212 A4 Sample 20210126 194533.fsa	B9 KD 19 2212 A4	PowerPlex 21 POP1 Panels vSS2.0			

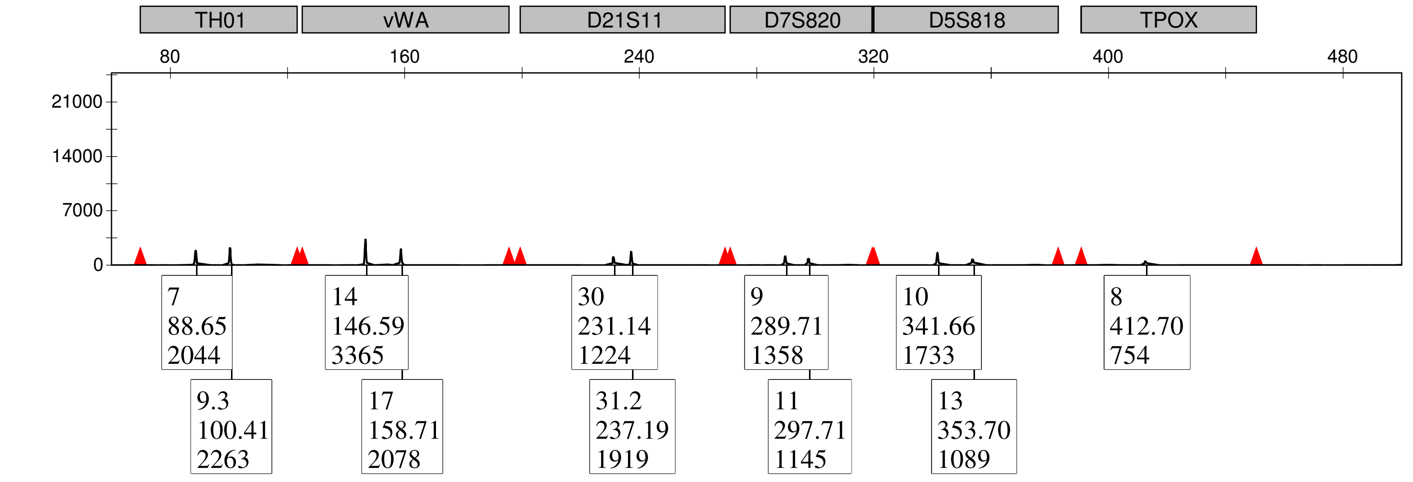

B9 B9 KD 19 2212 A4 Sample 20210126 194533.fsa	B9 KD 19 2212 A4	PowerPlex 21 POP1 Panels vSS2.0			

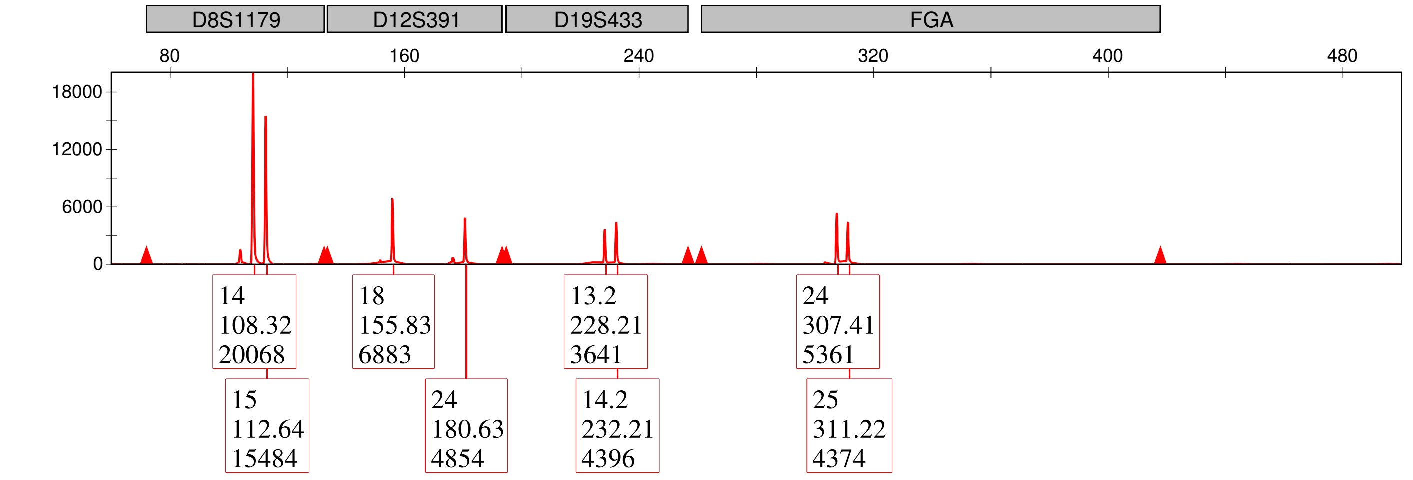

Applied Biosystems
GeneMapper Software 5

HM 20210127

Sample File	Sample Name	Panel	SQI	OS	SQ
C8_A10 NBL 17 1219_Sample_20210126_181926.fsa	A10 NBL 17 1219	PowerPlex_21_POP1_Panels_vSS2.0			

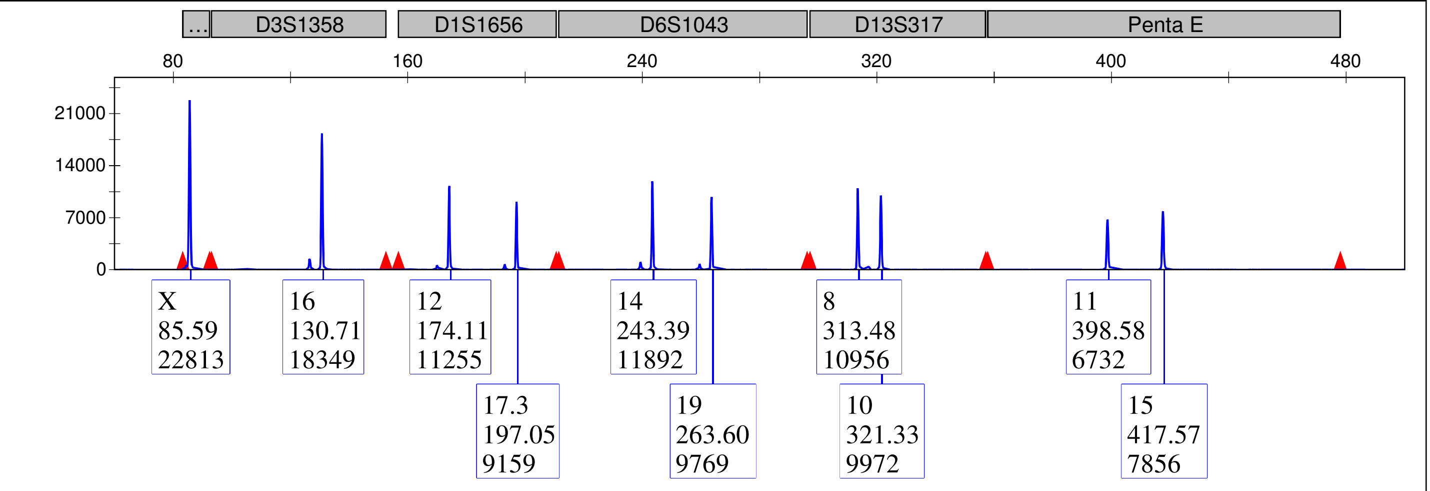

Sample File	Sample Name	Panel	SQI	OS	SQ
C8_A10 NBL 17 1219_Sample_20210126_181926.fsa	A10 NBL 17 1219	PowerPlex_21_POP1_Panels_vSS2.0			

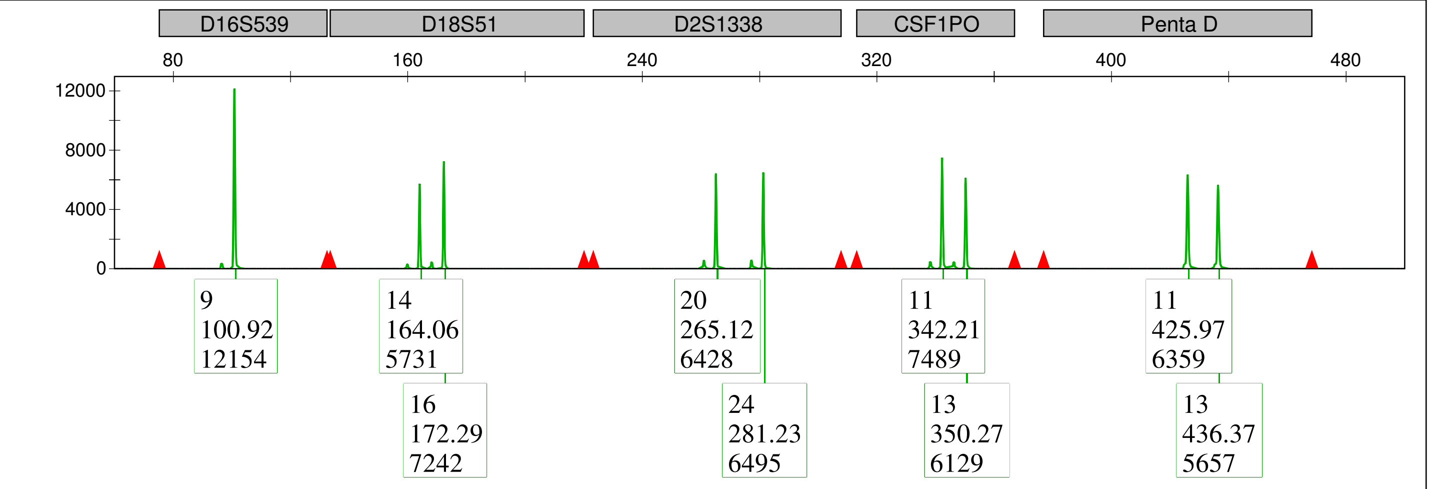

Sample File	Sample Name	Panel	SQI	OS	SQ
C8_A10 NBL 17 1219_Sample_20210126_181926.fsa	A10 NBL 17 1219	PowerPlex_21_POP1_Panels_vSS2.0			

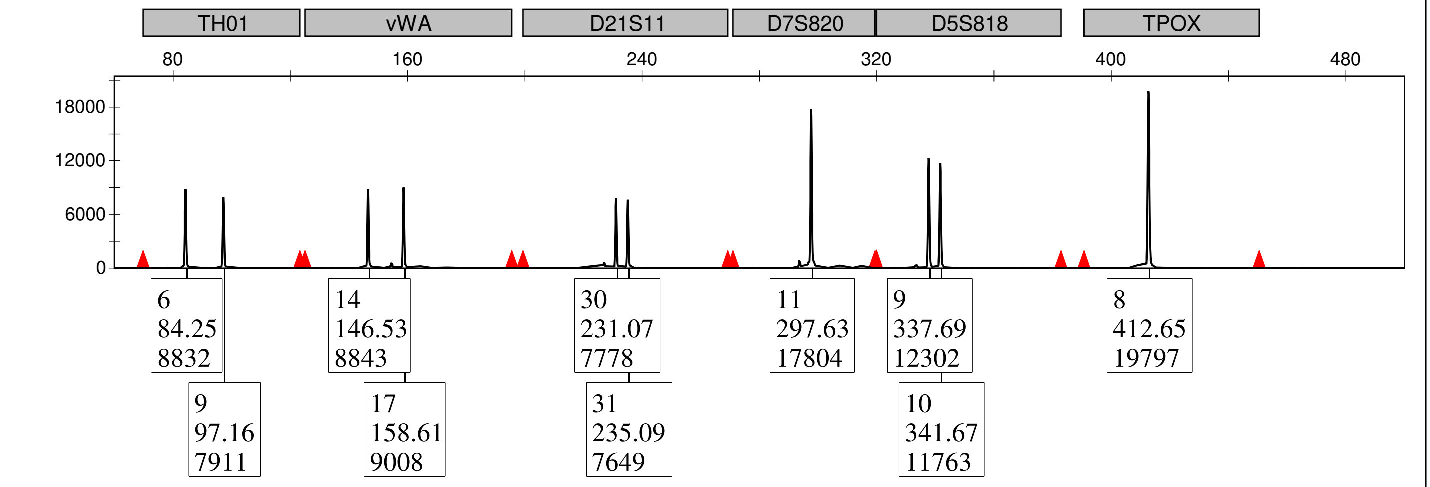

Sample File	Sample Name	Panel	SQI	OS	SQ
C8 A10 NBL 17 1219 Sample 20210126 181926.fsa	A10 NBL 17 1219	PowerPlex 21 POP1 Panels vSS2.0			

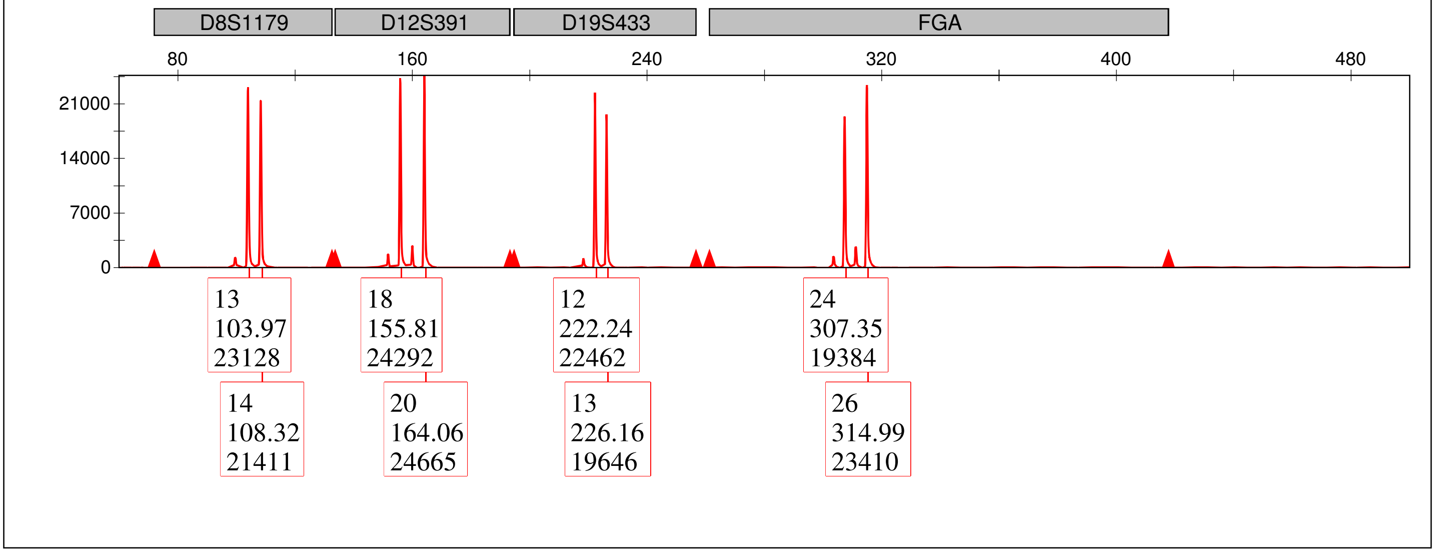

Sample File	Sample Name	Panel	SQI	OS	SQ
D9 B10 KD 19 2325C4 Sample 20210126 194535.fsa	B10 KD 19 2325C4	PowerPlex 21 POP1 Panels vSS2.0			

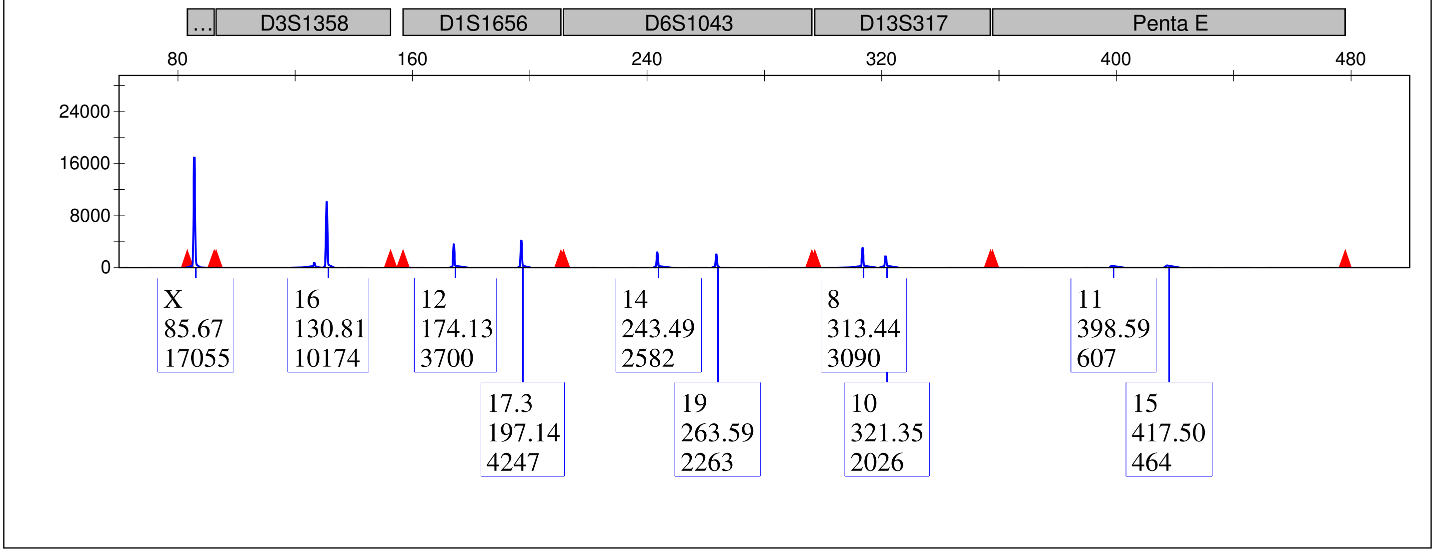

Sample File	Sample Name	Panel	SQI	OS	SQ
D9 B10 KD 19 2325C4 Sample 20210126 194535.fsa	B10 KD 19 2325C4	PowerPlex 21 POP1 Panels vSS2.0			

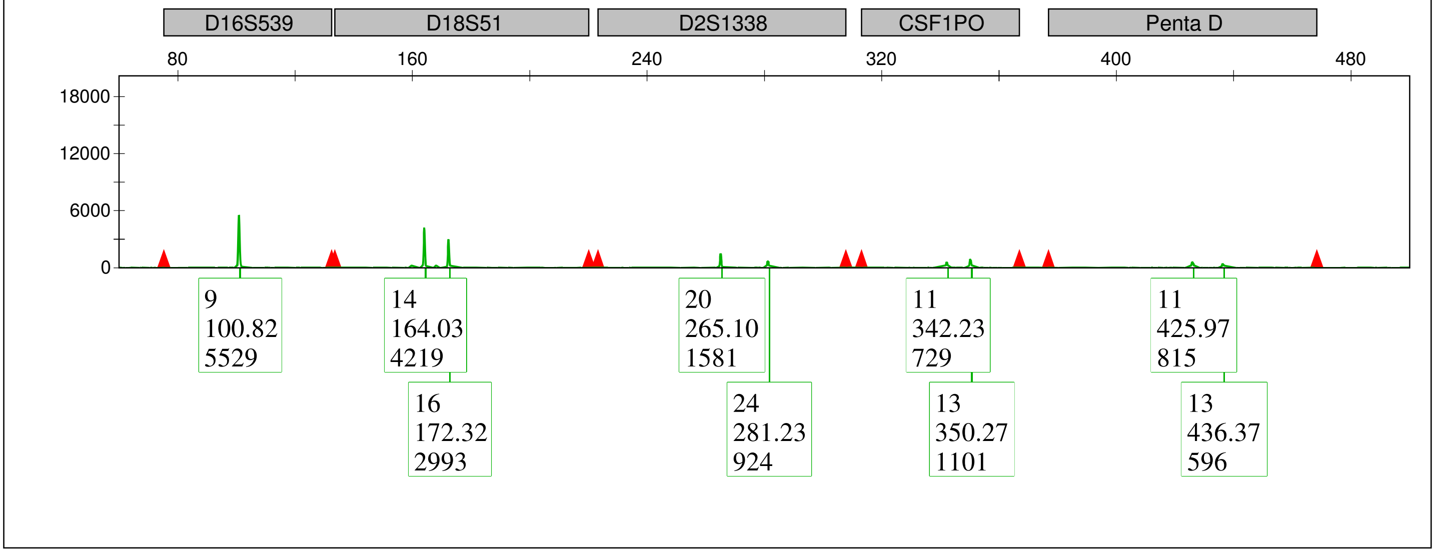

Applied Biosystems
GeneMapper Software 5

HM 20210127

Sample File	Sample Name	Panel	SQI	OS	SQ
D9_B10 KD 19 2325C4_Sample_20210126_194535.fsa	B10 KD 19 2325C4	PowerPlex_21_POP1_Panels_vSS2.0			

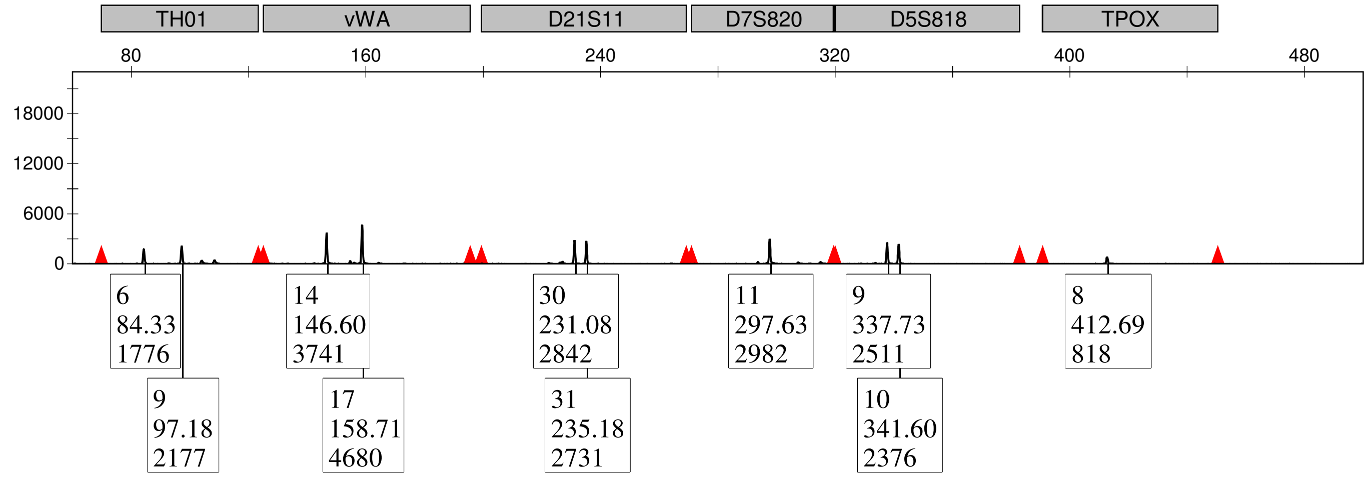

Sample File	Sample Name	Panel	SQI	OS	SQ
D9_B10 KD 19 2325C4_Sample_20210126_194535.fsa	B10 KD 19 2325C4	PowerPlex_21_POP1_Panels_vSS2.0			

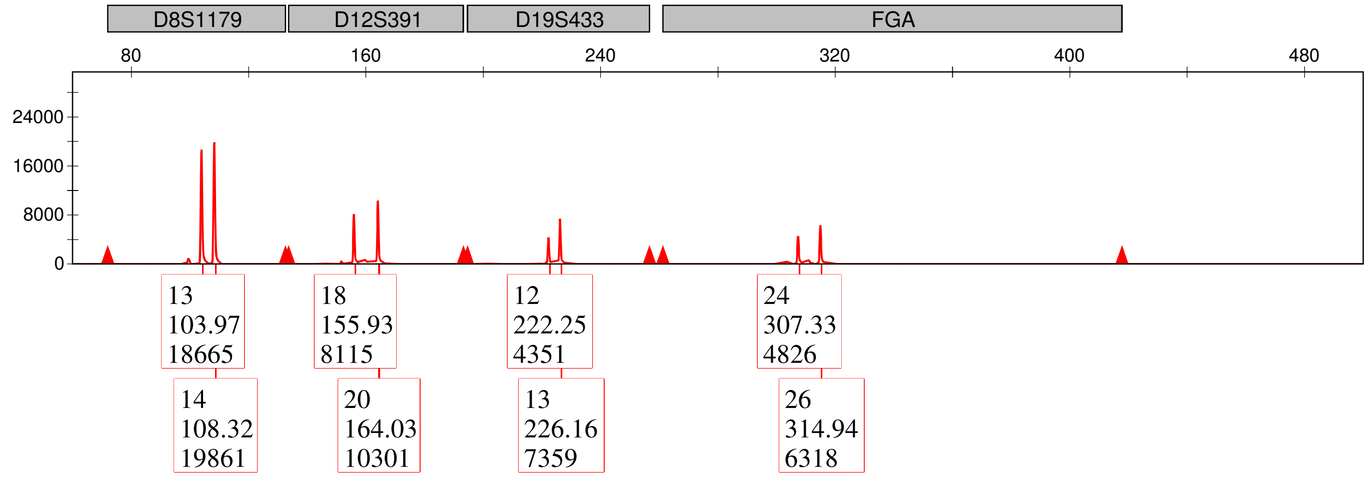

Sample File	Sample Name	Panel	SQI	OS	SQ
E9_A11 NBL 17 0120_Sample_20210126_202835.fsa	A11 NBL 17 0120	PowerPlex_21_POP1_Panels_vSS2.0			

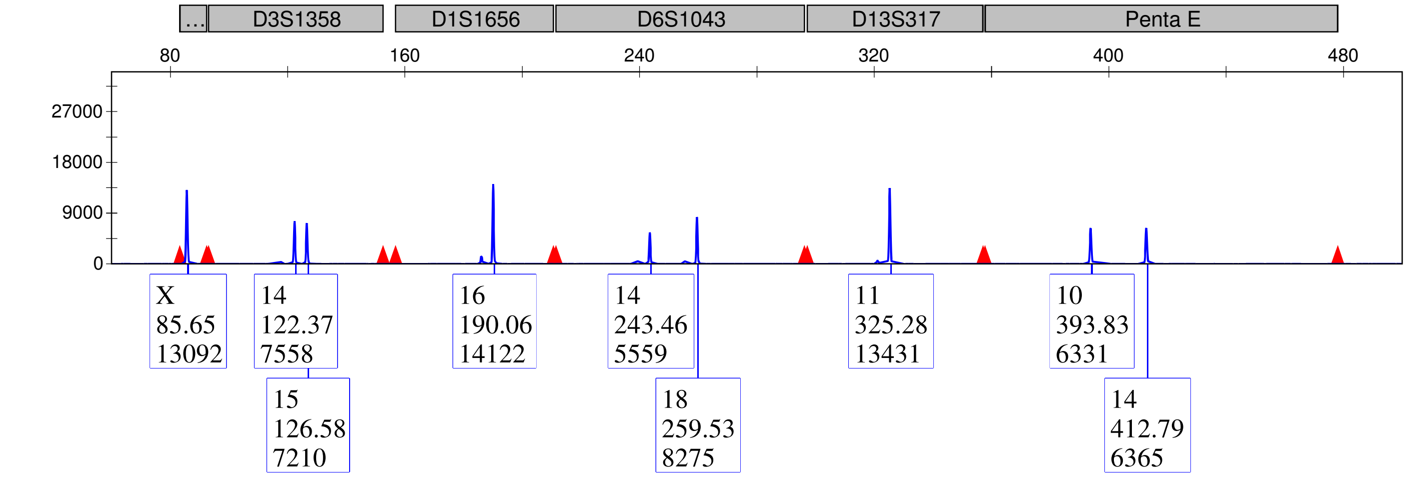

Sample File	Sample Name	Panel	SQI	OS	SQ
E9_A11 NBL 17 0120_Sample_20210126_202835.fsa	A11 NBL 17 0120	PowerPlex_21_POP1_Panels_vSS2.0			

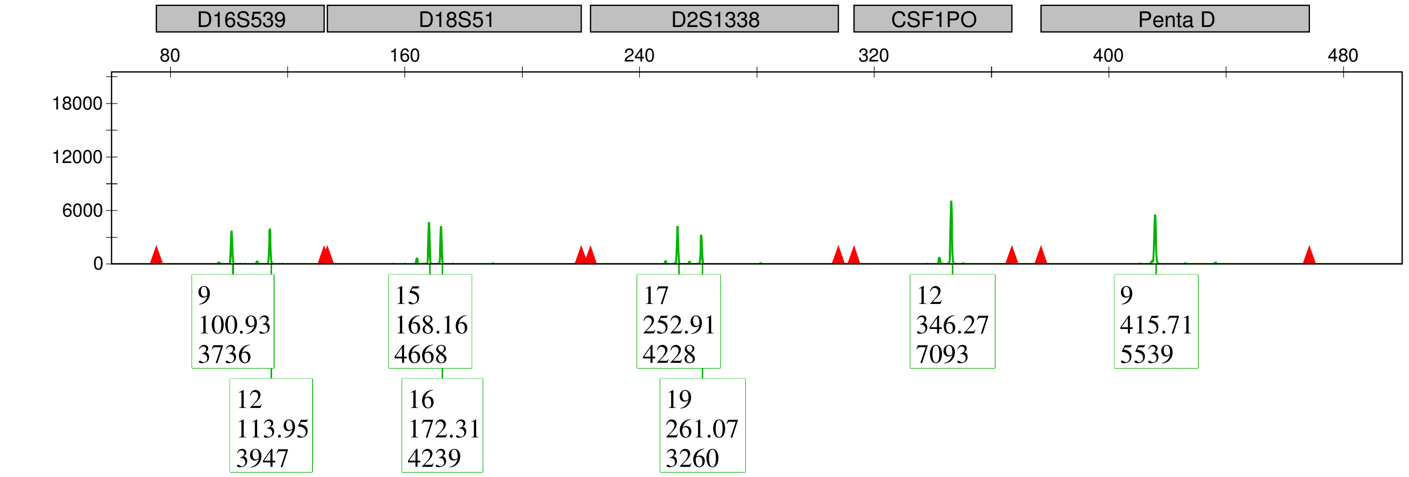

Sample File	Sample Name	Panel	SQI	OS	SQ
E9_A11 NBL 17 0120_Sample_20210126_202835.fsa	A11 NBL 17 0120	PowerPlex_21_POP1_Panels_vSS2.0			

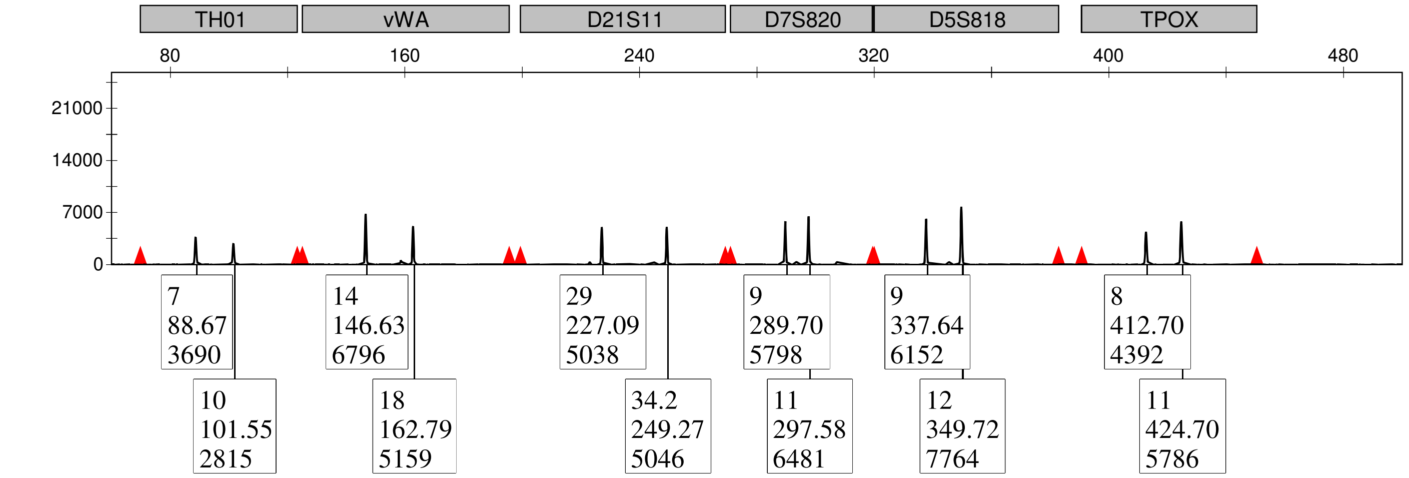

Sample File	Sample Name	Panel	SQI	OS	SQ
E9_A11 NBL 17 0120_Sample_20210126_202835.fsa	A11 NBL 17 0120	PowerPlex_21_POP1_Panels_vSS2.0			

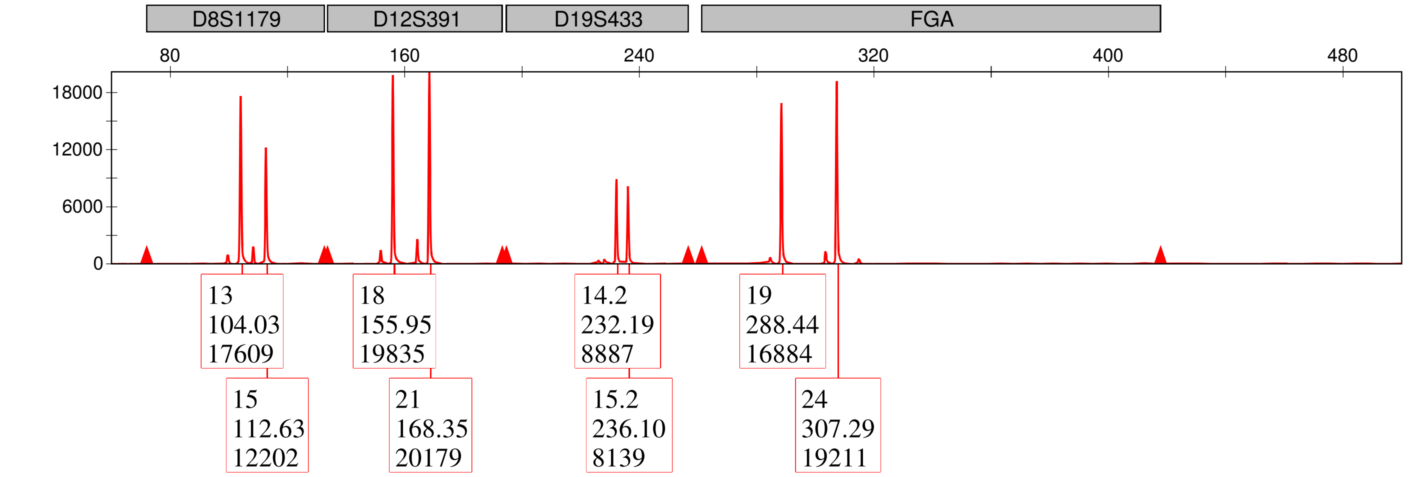

F9_B11 KD 20 100A1_Sample_20210126_202836.fsa	B11 KD 20 100A1	PowerPlex_21_POP1_Panels_vSS2.0			

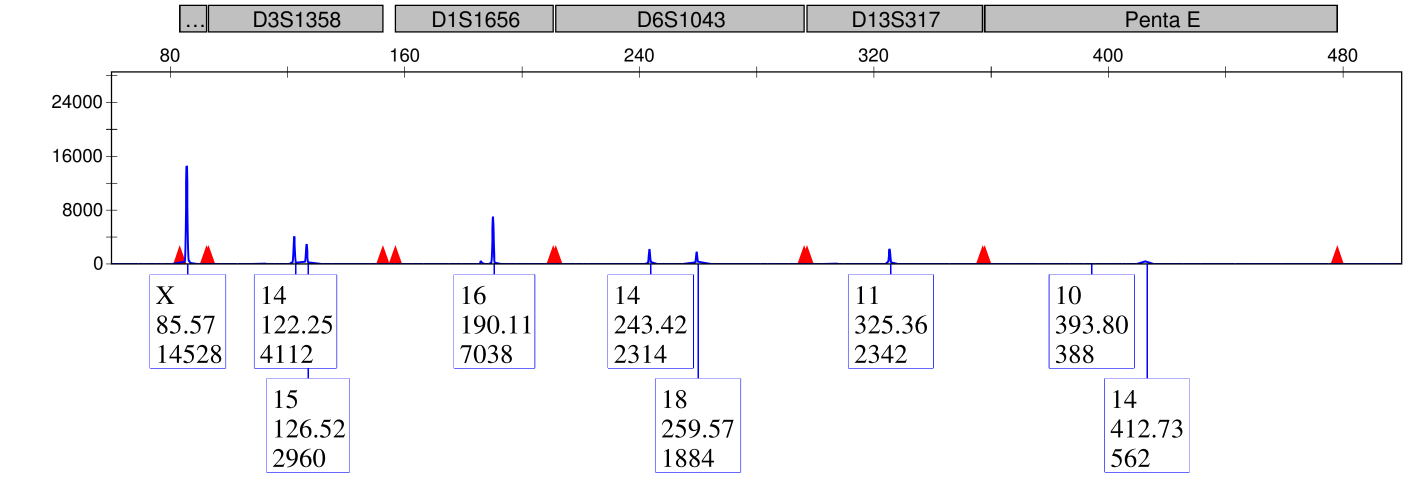

F9_B11 KD 20 100A1_Sample_20210126_202836.fsa	B11 KD 20 100A1	PowerPlex_21_POP1_Panels_vSS2.0			

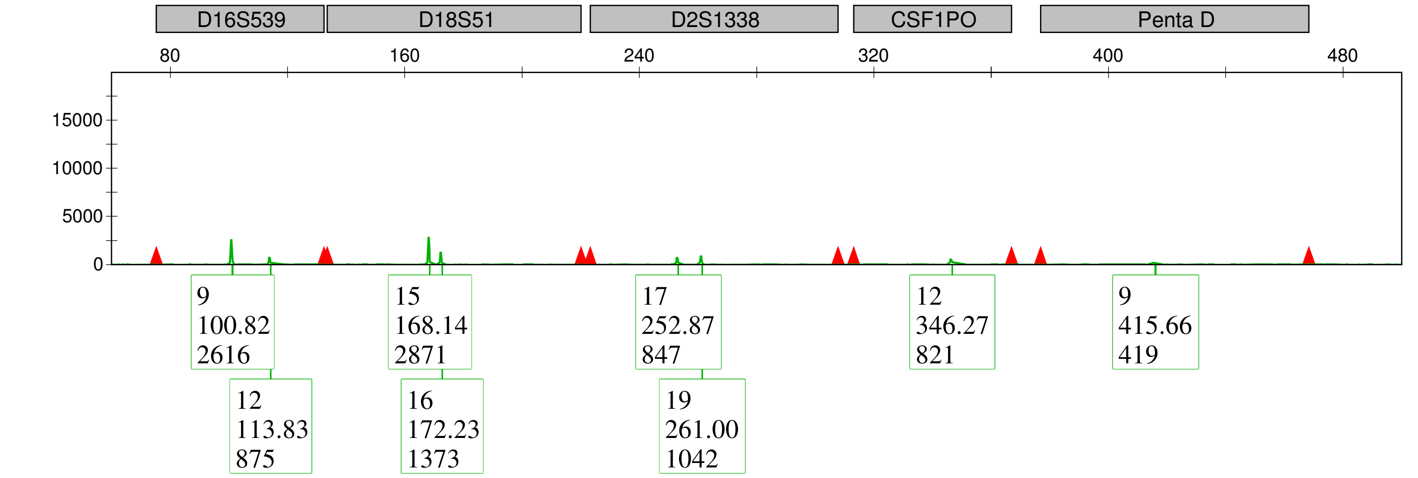

Sample File	Sample Name	Panel	SQI	OS	SQ
F9_B11 KD 20 100A1_Sample_20210126_202836.fsa	B11 KD 20 100A1	PowerPlex_21_POP1_Panels_vSS2.0			

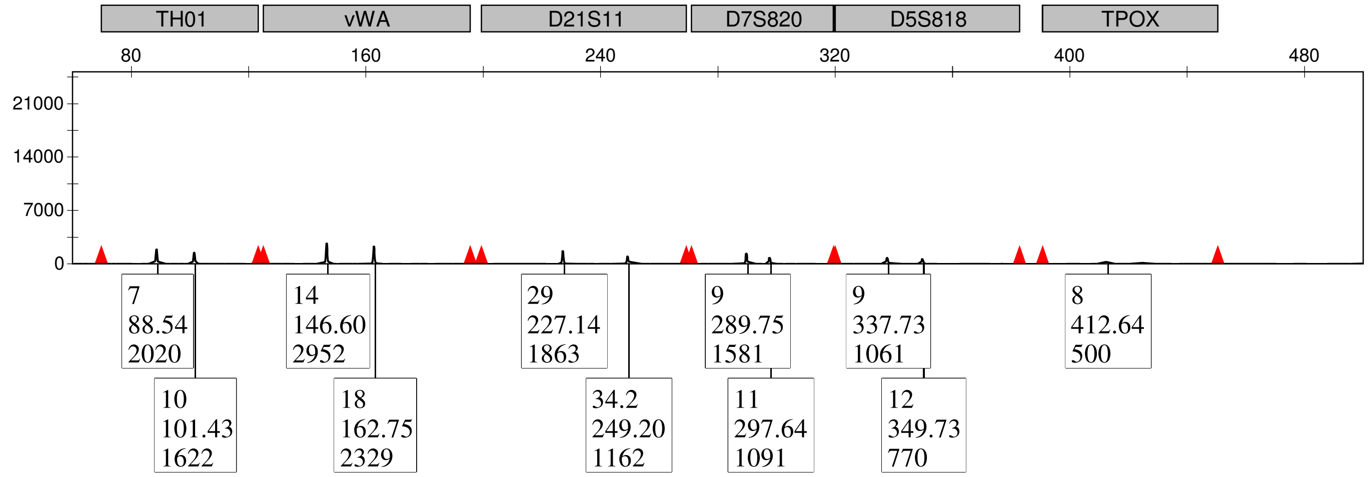

Sample File	Sample Name	Panel	SQI	OS	SQ
F9_B11 KD 20 100A1_Sample_20210126_202836.fsa	B11 KD 20 100A1	PowerPlex_21_POP1_Panels_vSS2.0			

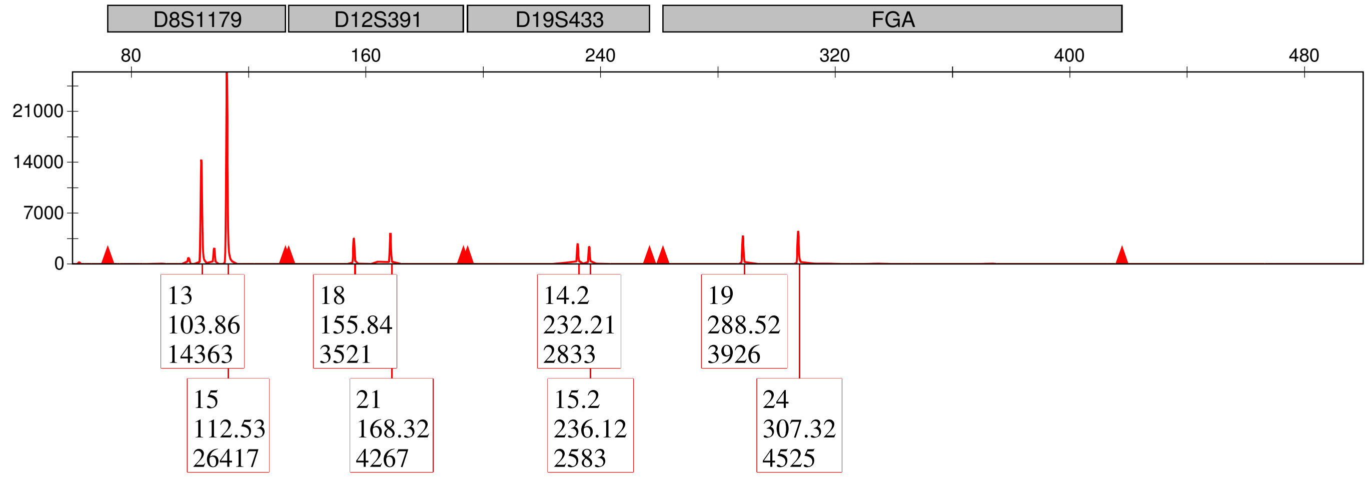

Applied Biosystems
GeneMapper Software 5

HM 20210127

Sample File	Sample Name	Panel	SQI	OS	SQ
G9_A12 NBL 21 0120_Sample_20210126_202837.fsa	A12 NBL 21 0120	PowerPlex_21_POP1_Panels_vSS2.0			

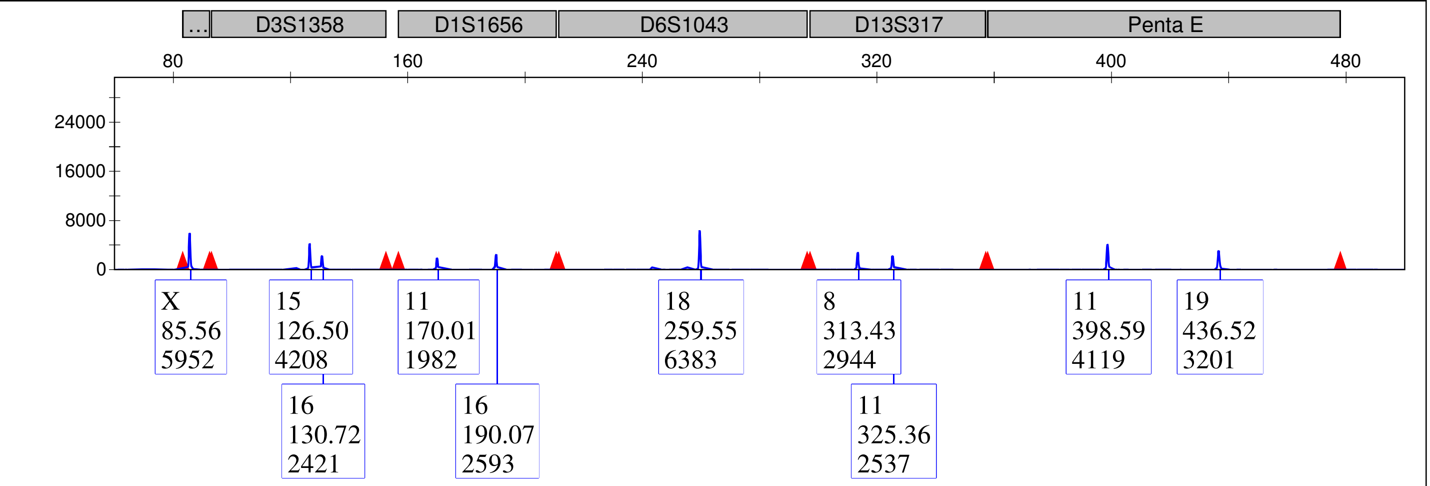

G9_A12 NBL 21 0120_Sample_20210126_202837.fsa | A12 NBL 21 0120 | PowerPlex_21_POP1_Panels_vSS2.0

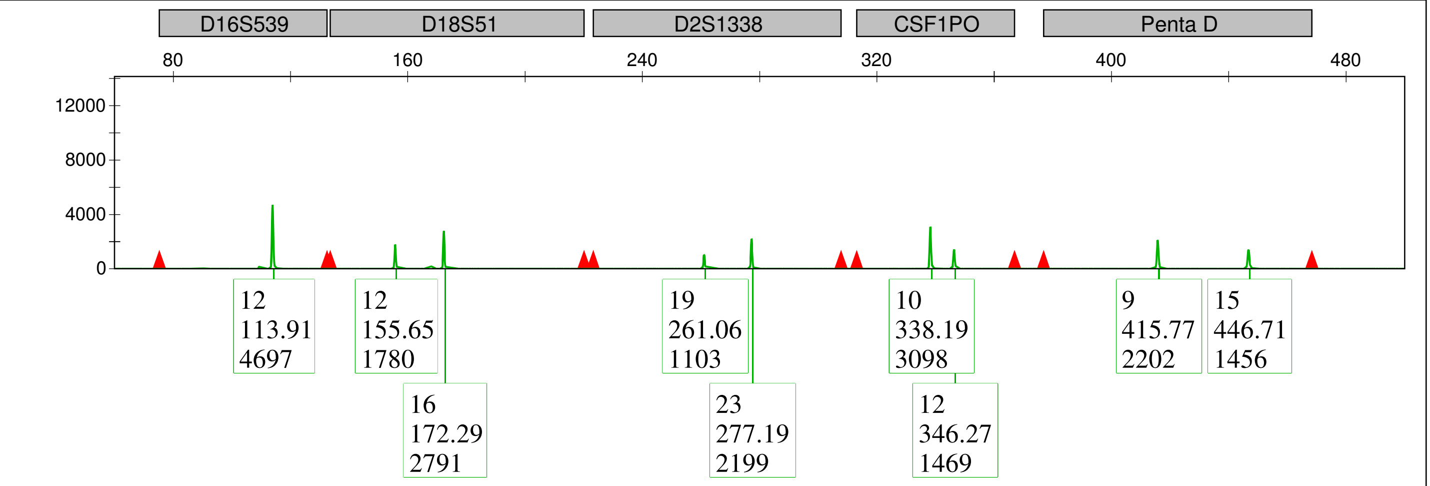

G9_A12 NBL 21 0120_Sample_20210126_202837.fsa | A12 NBL 21 0120 | PowerPlex_21_POP1_Panels_vSS2.0

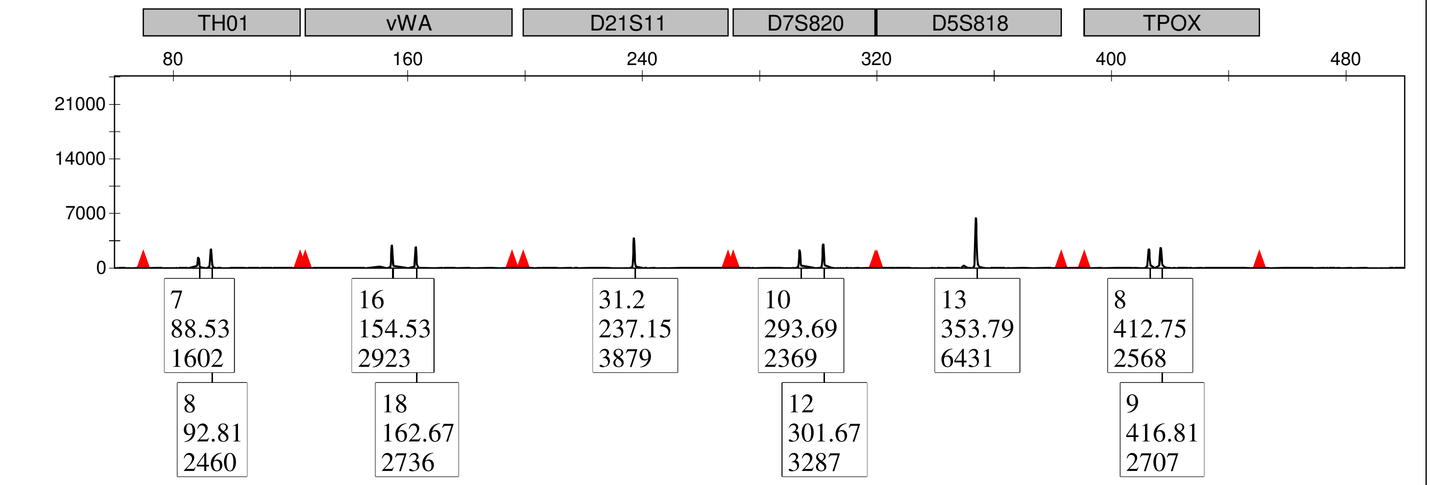

Sample File	Sample Name	Panel	SQI	OS	SQ
G9_A12 NBL 21 0120_Sample_20210126_202837.fsa	A12 NBL 21 0120	PowerPlex_21_POP1_Panels_vSS2.0			

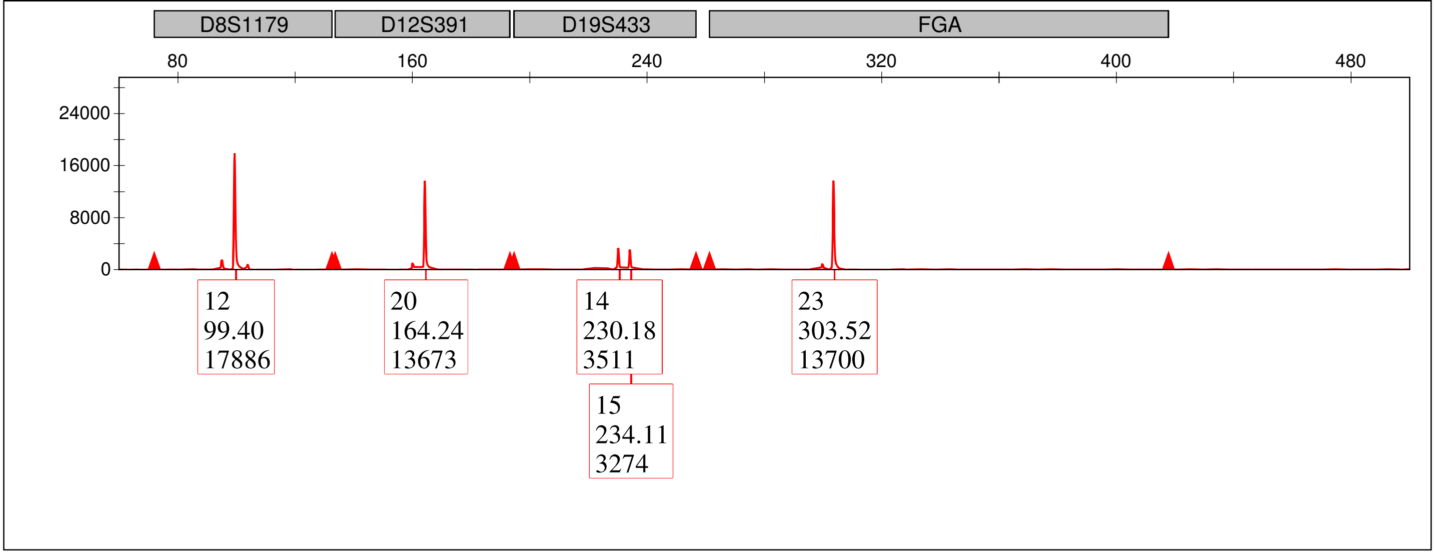

Sample File	Sample Name	Panel	SQI	OS	SQ
H9_B12 KD 20 120A2_Sample_20210126_202838.fsa	B12 KD 20 120A2	PowerPlex_21_POP1_Panels_vSS2.0			

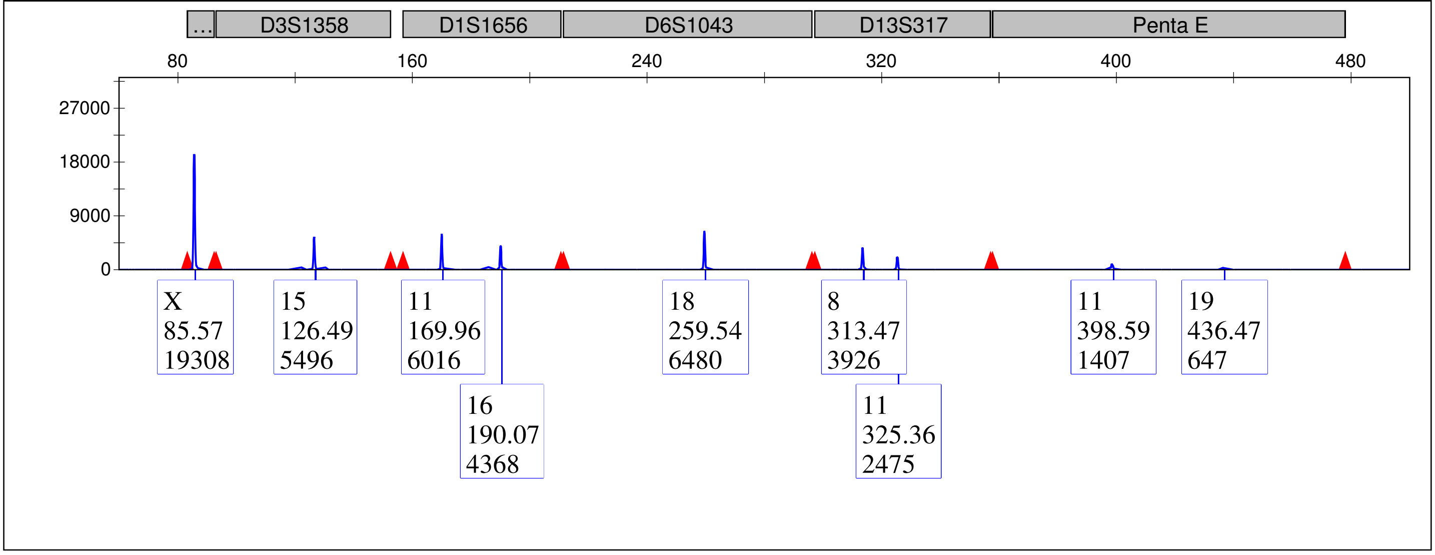

Sample File	Sample Name	Panel	SQI	OS	SQ
H9_B12 KD 20 120A2_Sample_20210126_202838.fsa	B12 KD 20 120A2	PowerPlex_21_POP1_Panels_vSS2.0			

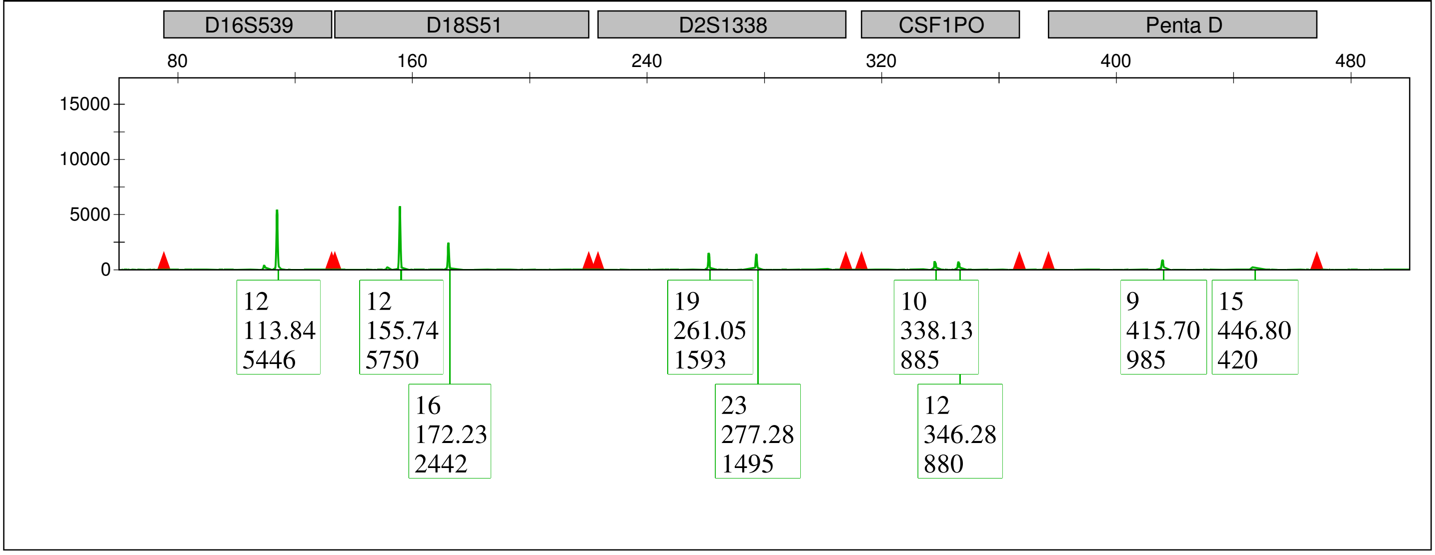

Applied Biosystems
GeneMapper Software 5

HM 20210127

Sample File	Sample Name	Panel	SQI	OS	SQ
H9 B12 KD 20 120A2 Sample 20210126 202838.fsa	B12 KD 20 120A2	PowerPlex 21 POP1 Panels vSS2.0			

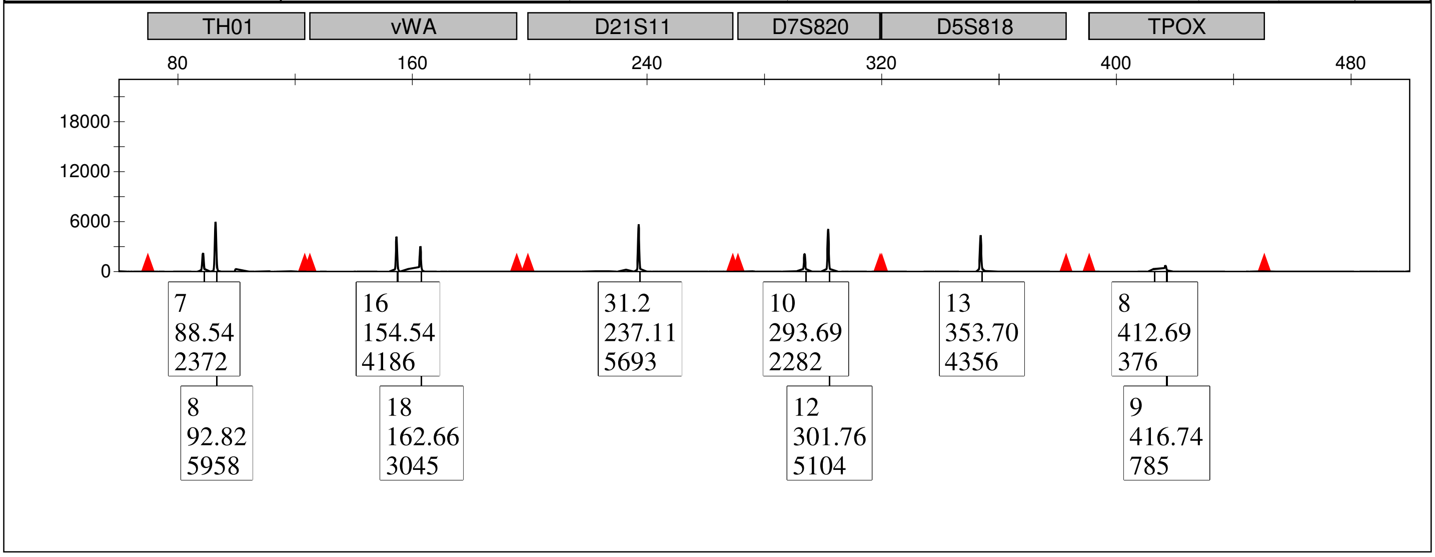

H9 B12 KD 20 120A2 Sample 20210126 202838.fsa	B12 KD 20 120A2	PowerPlex 21 POP1 Panels vSS2.0			

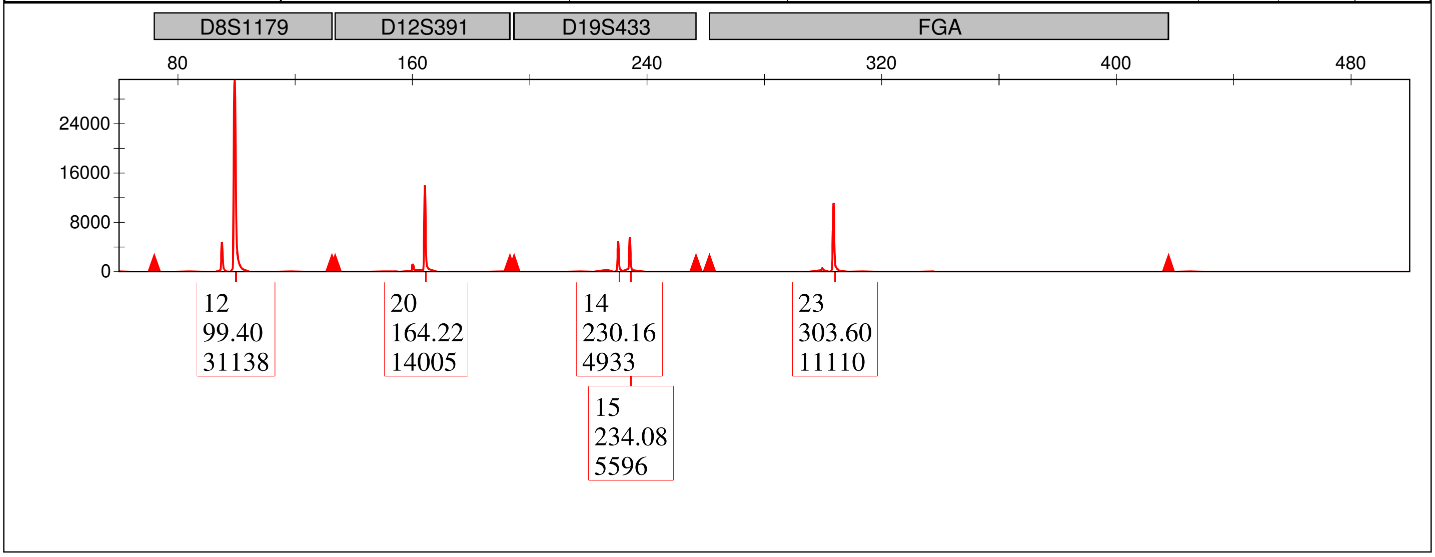

Supplement: Supplementary Data Sheet 1 — Genomic profiling, drug screening and Pearson correlation data. [file DataSheet_1.pdf]
