# Supplementary material for: Integrated Genomic Profiling and Drug Screening of Patient-Derived Cultures Identifies Individualized Copy Number-Dependent Susceptibilities Involving PI3K Pathway and 17q Genes in Neuroblastoma
Source: Front Oncol. 2021 Oct 14;11:709525. doi: 10.3389/fonc.2021.709525 (PMC8551924; doi:10.3389/fonc.2021.709525)
Supplement: Supplementary file 11 [file Table_1.docx]

**Supplementary Table S1. Patient characteristics**

| **S/N** | **ID** | **Sex** | **Age (years)** | **Treatment status** | **INPC** | **INSS stage** | **MYCN amp** | **1p del** | **11q del** |
| --- | --- | --- | --- | --- | --- | --- | --- | --- | --- |
| 1 | NBL 02-0616 | M | 2.24 | Post-Chemo | UH | 4 | No | 30% | 39% |
| 19 | NBL 06-1218 | M | 1.45 | Post-Chemo | FH | 4 | Massive | 14.80% | No |
| 21 | NBL 18-0619 | M | 2.79 | Post-Chemo | FH | 4 | Yes | No | No |
| 20 | NBL 25-0619 | F | 2.41 | Post-Chemo | FH | 4 | No | No | No |
| 22 | NBL 02-0719 | M | 5.89 | Pre-Chemo | UH | 2 | No | 85.70% | 78% |
| 24 | NBL 30-0719 | M | 4.17 | Post-Chemo | FH | 4 | No | Aneuploid | Aneuploid |
| 26 | NBL 03-1019 | M | 7.80 | Post-Chemo | UH | 4 | No | No | No |
| 27 | NBL 29-1019 | F | 6.41 | Post-Chemo | UH | 4 | No | No | No |
| 28 | NBL 03-1219A | M | 2.36 | Pre-Chemo | UH | 2 | No | No | 19.60% |
| 29 | NBL 03-1219B | M | 2.31 | Post-Chemo | FH | 4 | Massive | 68.80% | No |
| 30 | NBL 17-1219 | F | 7.3 | Relapse | FH | 2 | No | No | No |
| 31 | NBL 17-0120 | F | 3.19 | Relapse | UH | 2 | Massive | 58% | 64% |
| 32 | NBL 21-0120 | F | 2.50 | Pre-Chemo | UH | 4 | No | No | 31% |

INPC: International Neuroblastoma Pathology Classification, INSS: International Neuroblastoma Staging System; UH: unfavorable histology, FH: favorable histology
