# Supplementary material for: Integrated Genomic Profiling and Drug Screening of Patient-Derived Cultures Identifies Individualized Copy Number-Dependent Susceptibilities Involving PI3K Pathway and 17q Genes in Neuroblastoma
Source: Front Oncol. 2021 Oct 14;11:709525. doi: 10.3389/fonc.2021.709525 (PMC8551924; doi:10.3389/fonc.2021.709525)
Supplement: Supplementary file 12 [file Table_2.docx]

**Supplementary Table S2. Structural variants**

| ID | Single nucleotide variant | | | Copy number variation (Gene (C.N.)) |  |
| --- | --- | --- | --- | --- | --- |
|  | **Gene** | **Nucleotide** **Amino acid** | **A.F, read depth** **COSMIC IDs** |  |  |
| NBL 02-0616 | RET | c.2297C>T p.(Pro766Leu) | 0.48, 1819x 5762922, 5762923 | CD6 (3.5) | |
|  | TSC2 | c.856A>G p.(Met286Val) | 0.48, 885x 6538419, 6538420 |  |  |
|  | KMT2D | c.10045A>G p.(Met3349Val) | 0.41, 312x |  |  |
|  | ASXL2 | c.2750C>T p.(Ser917Leu) | 0.48, 4038x |  |  |
|  | CHD7 | c.1565G>T p.(Gly522Val) | 0.51, 4129x |  |  |
| NBL 06-1218 | TSC2 | c.856A>G p.(Met286Val) | 0.33, 349x 6538419, 6538420 | MYCN (77) | |
|  | ARID1B | c.4649G>A p.(Ser1550Asn) | 0.5, 5267x |  |  |
| NBL 25-0619 | TSC2 | c.673G>A p.(Val225Met) | 0.5, 379x 4059098, 4059099 | - | |
| NBL 18-0619 | TP53 | c.824G>T p.(Cys275Phe) | 0.24, 2427x 99932, 10701, 3723938, 1637959 | MYCN (92.5)  GLI2 (3) | |
|  | KMT2D | c.7046C>T p.(Pro2349Leu) | 0.52, 2103x |  |  |
| NBL 02-0719 | APC | c.5513T>C p.(Phe1838Ser) | 0.5, 6731x | CDK6 (4)  EGFR (4)  ERBB2 (3.5)  BRAF (3)  MET (3) | |
|  | ARID1A | c.5944G>A p.(Val717Leu) | 0.33, 6162x 1580528 |  |  |
|  | EGFR | c.2149G>C p.(Val717Leu) | 0.16, 3104x |  |  |
|  | TSC2 | c.856A>G p.(Met286Val) | 0.9, 1135x 6538419, 6538420 |  |  |
|  | NCOR2 | c.1523_1531dupAGCAGCAGC p.(Gln508_Gln510dup) | 0.26, 4310x |  |  |
|  | CRLF2 | c.671C>T p.(Pro224Leu) | 0.52, 6352x |  | |
| NBL 30-0719 | TP53 | c.528C>A p.(Cys176Ter) | 0.53, 898x 4813803, 4813802, 43734, 179825 | CCND1 (3)  FGFR4 (3)  MDM4 (3) | |
|  | TSC1 | c.250G>A p.(Ala84Thr) | 0.48, 3652x |  |  |
|  | TSC2 | c.3871G>A p.(Val1291Ile) | 0.50, 1113x |  |  |
| NBL 03-1019 |  |  |  | ALK (3)  MYCN (3) | |
|  |  |  |  |  |  |
| NBL29-1019 | ARID1A | c.3046G>A p.(Gly1016Ser) | 0.49, 2979x |  | |
|  | ARID1A | c.5615C>T p.(Ala1872Val) | 0.48, 1058x 1734087 |  |  |
|  | CHD7 | c.1565G>T p.(Gly522Val) | 0.37, 4492x |  |  |
|  | FAS | c.432C>A p.(Asp144Glu) | 0.65, 6207x |  |  |
| NBL 03-1219A | KDR | c.1444T>C p.(Cys482Arg) | 0.46, 2597x 5020657 | PIK3CA (3) | |
| NBL 03-1219B | TET2 | c.100C>T p.(Leu34Phe) | 0.53, 690x 5941242, 5941243 | MYCN (13.5)  JAK1 (1)  JAK2 (1) | |
|  | TET2 | c.4627_4644delAGACCCCAGCAGCAGCAG p.(Arg1543_Gln1548del) | 0.63, 2333x |  |  |
|  | FGFR1 | c.1638C>A p.(Asn546Lys) | 0.44, 575x 1284966, 1284967, 3670398, 19176 |  |  |
| NBL 17-1219 | TP53 | c.31G>C p.(Glu11Gln) | 0.49, 1515x 11606, 2745204, 4272170, 327260 | - | |
|  | RUNX1 | c.737C>T p.(Thr246Met) | 0.53, 1209x 1030459 |  | |
| NBL 17-0120 | ARID1B | c.4010C>T p.(Thr1337Met) | 0.47,1235x 1075349, 1596109 | MYCN (198.5)  JAK2 (1) | |
|  | GNA13 | c.392T>A p.(Met131Lys) | 0.07, 4989x |  |  |
| NBL21-0120 | TET2 | c.4931C>T p.(Pro1644Leu) | 0.51,3474x | - | |
|  | TSC1 | c.250G>A p.(Ala84Thr) | 0.48, 3652x |  |  |

a.a. amino acid, A.F, allelic frequency, C.N., copy number
