# Supplementary material for: Integrated Genomic Profiling and Drug Screening of Patient-Derived Cultures Identifies Individualized Copy Number-Dependent Susceptibilities Involving PI3K Pathway and 17q Genes in Neuroblastoma
Source: Front Oncol. 2021 Oct 14;11:709525. doi: 10.3389/fonc.2021.709525 (PMC8551924; doi:10.3389/fonc.2021.709525)
Supplement: Supplementary file 13 [file Table_3.docx]

**Supplementary Table S3. Mean copy number per gene segment.**

| **Gene** | **Chromosome** | **Position** | | **PDC** | | | | | | | | | | | | |
| --- | --- | --- | --- | --- | --- | --- | --- | --- | --- | --- | --- | --- | --- | --- | --- | --- |
|  |  | **Start** | **End** | **NBL 020616** | **NBL 061218** | **NBL 031019** | **NBL 031219A** | **NBL 031219B** | **NBL 171219** | **NBL 170120** | **NBL 020719** | **NBL 250619** | **NBL 180619** | **NBL 300719** | **NBL 291019** | **NBL 210120** |
| MTOR | 1 | 2 | 13 | 2.00 | 1.00 | 2.00 | 2.00 | 1.00 | 2.00 | 1.00 | 2.00 | 2.00 | 2.00 | 2.00 | 2.00 | 1.42 |
| ARID1A | 1 | 14 | 110 | 2.00 | 1.09 | 2.00 | 2.00 | 1.09 | 2.00 | 1.09 | 2.00 | 2.00 | 2.00 | 2.00 | 2.00 | 1.96 |
| CSF3R | 1 | 111 | 116 | 2.00 | 1.17 | 2.00 | 2.00 | 1.17 | 2.00 | 1.17 | 2.00 | 2.00 | 2.00 | 2.00 | 2.00 | 2.00 |
| MPL | 1 | 117 | 117 | 2.00 | 1.00 | 2.00 | 2.00 | 1.00 | 2.00 | 2.00 | 2.00 | 2.00 | 2.00 | 2.00 | 2.00 | 2.00 |
| JAK1 | 1 | 118 | 144 | 2.00 | 2.00 | 2.00 | 2.00 | 1.07 | 2.00 | 2.00 | 2.00 | 2.00 | 2.00 | 2.46 | 2.00 | 2.00 |
| CHP2 | 1 | 145 | 146 | 2.00 | 2.00 | 2.00 | 2.00 | 2.00 | 2.00 | 2.00 | 2.00 | 2.00 | 2.00 | 2.00 | 2.00 | 2.00 |
| FASLG | 1 | 147 | 152 | 2.00 | 2.00 | 2.00 | 2.00 | 2.00 | 2.00 | 2.00 | 2.00 | 2.00 | 2.00 | 2.00 | 2.00 | 2.00 |
| ABL2 | 1 | 153 | 172 | 2.45 | 2.00 | 2.00 | 2.00 | 2.00 | 2.00 | 2.00 | 2.03 | 2.00 | 2.00 | 2.05 | 2.00 | 2.00 |
| MDM4 | 1 | 173 | 184 | 2.50 | 2.00 | 2.00 | 2.00 | 2.00 | 2.00 | 2.00 | 2.50 | 2.00 | 2.00 | 3.00 | 2.00 | 2.00 |
| H3F3A | 1 | 185 | 185 | 2.50 | 2.00 | 2.00 | 2.00 | 2.00 | 2.00 | 2.00 | 2.50 | 2.00 | 2.00 | 3.00 | 2.00 | 2.00 |
| MYCN | 2 | 186 | 195 | 2.00 | 69.50 | 2.90 | 2.00 | 12.35 | 2.00 | 178.85 | 2.00 | 2.00 | 83.45 | 2.00 | 2.00 | 2.00 |
| DNMT3A | 2 | 196 | 220 | 2.00 | 2.00 | 3.00 | 2.00 | 2.00 | 2.00 | 1.50 | 2.02 | 2.00 | 2.50 | 2.00 | 2.00 | 2.00 |
| ASXL2 | 2 | 221 | 264 | 2.00 | 1.77 | 2.98 | 2.00 | 2.00 | 2.00 | 1.51 | 2.16 | 2.00 | 2.49 | 2.00 | 2.00 | 2.00 |
| ALK | 2 | 265 | 273 | 2.00 | 2.00 | 3.00 | 2.00 | 2.00 | 2.00 | 1.94 | 2.00 | 2.00 | 2.50 | 2.00 | 2.00 | 2.00 |
| ON | 2 | 274 | 274 | 2.00 | 2.00 | 3.00 | 2.00 | 2.00 | 2.00 | 2.00 | 2.00 | 2.00 | 2.50 | 2.00 | 2.00 | 2.00 |
| MSH6 | 2 | 275 | 277 | 2.00 | 2.00 | 2.00 | 2.00 | 2.00 | 2.00 | 2.00 | 2.00 | 2.00 | 2.67 | 2.00 | 2.00 | 2.00 |
| GLI2 | 2 | 278 | 288 | 2.00 | 2.00 | 2.00 | 2.00 | 2.00 | 2.00 | 2.00 | 2.00 | 2.00 | 3.00 | 2.00 | 2.00 | 2.00 |
| ACVR1 | 2 | 289 | 306 | 2.00 | 2.00 | 2.00 | 2.00 | 2.00 | 2.00 | 2.00 | 1.56 | 2.00 | 2.83 | 2.00 | 2.00 | 2.00 |
| SP | 2 | 307 | 307 | 2.00 | 2.00 | 2.00 | 2.00 | 2.00 | 2.00 | 2.00 | 1.50 | 2.00 | 2.00 | 2.00 | 2.00 | 2.00 |
| RAF1 | 2 | 308 | 312 | 1.50 | 1.50 | 2.00 | 2.00 | 2.00 | 2.00 | 2.00 | 1.50 | 2.00 | 2.00 | 2.00 | 2.00 | 1.40 |
| CTNNB1 | 3 | 313 | 324 | 1.50 | 1.50 | 2.00 | 2.00 | 2.00 | 2.00 | 2.00 | 1.50 | 2.00 | 2.00 | 2.00 | 2.00 | 1.00 |
| CCR5 | 3 | 325 | 325 | 1.50 | 1.50 | 2.00 | 2.00 | 2.00 | 2.00 | 2.00 | 1.50 | 2.00 | 2.00 | 2.00 | 2.00 | 1.00 |
| SETD2 | 3 | 326 | 332 | 1.57 | 1.57 | 2.00 | 2.00 | 2.00 | 2.00 | 2.00 | 1.57 | 2.00 | 1.93 | 1.93 | 2.00 | 1.14 |
| RHOA | 3 | 333 | 337 | 1.50 | 1.50 | 2.00 | 2.00 | 2.00 | 2.00 | 2.00 | 1.60 | 2.00 | 1.50 | 1.50 | 2.00 | 1.00 |
| GATA2 | 3 | 338 | 346 | 2.00 | 1.50 | 2.00 | 2.78 | 2.00 | 2.00 | 2.00 | 2.00 | 2.00 | 2.00 | 2.50 | 2.00 | 2.00 |
| PIK3CA | 3 | 347 | 357 | 2.00 | 1.55 | 2.00 | 2.91 | 2.00 | 2.00 | 2.00 | 2.00 | 2.00 | 2.00 | 2.45 | 2.00 | 2.00 |
| FGFR3 | 4 | 358 | 367 | 2.00 | 2.00 | 2.00 | 2.00 | 2.00 | 2.00 | 2.00 | 1.50 | 2.00 | 2.50 | 2.00 | 2.50 | 2.00 |
| WHSC1 | 4 | 368 | 427 | 2.00 | 2.00 | 2.00 | 2.00 | 2.00 | 2.00 | 2.00 | 1.51 | 2.00 | 2.49 | 2.00 | 2.49 | 2.00 |
| PDGFRA | 4 | 428 | 448 | 2.00 | 2.00 | 2.00 | 2.00 | 2.00 | 2.00 | 2.00 | 1.50 | 2.00 | 2.50 | 2.00 | 2.50 | 2.00 |
| KIT | 4 | 449 | 462 | 2.00 | 2.00 | 2.00 | 2.00 | 2.00 | 2.00 | 2.00 | 1.54 | 2.00 | 2.46 | 2.00 | 2.46 | 2.00 |
| KDR | 4 | 463 | 463 | 2.00 | 2.00 | 2.00 | 2.00 | 2.00 | 2.00 | 2.00 | 1.50 | 2.00 | 2.50 | 2.00 | 2.50 | 2.00 |
| TET2 | 4 | 464 | 538 | 1.73 | 2.00 | 2.00 | 2.00 | 2.00 | 2.00 | 2.00 | 1.53 | 2.00 | 2.47 | 2.00 | 2.47 | 2.00 |
| FBXW7 | 4 | 539 | 540 | 2.00 | 2.00 | 2.00 | 2.00 | 2.00 | 2.00 | 2.00 | 1.50 | 2.00 | 2.50 | 2.00 | 2.50 | 2.00 |
| IL7R | 4 | 541 | 543 | 2.00 | 2.00 | 2.00 | 2.00 | 2.00 | 2.00 | 2.00 | 2.00 | 2.00 | 2.00 | 3.00 | 2.00 | 2.00 |
| PIK3R1 | 5 | 544 | 557 | 2.00 | 2.00 | 2.00 | 2.00 | 2.00 | 2.00 | 2.00 | 2.00 | 2.00 | 2.00 | 2.93 | 2.00 | 2.00 |
| APC | 5 | 558 | 678 | 2.00 | 2.00 | 2.00 | 2.00 | 2.00 | 2.00 | 2.00 | 2.00 | 2.00 | 2.00 | 2.95 | 2.00 | 2.00 |
| CSF1R | 5 | 679 | 681 | 2.00 | 2.00 | 2.00 | 2.00 | 2.00 | 2.00 | 2.00 | 2.00 | 2.00 | 2.00 | 3.00 | 2.00 | 2.00 |
| PDGFRB | 5 | 682 | 683 | 2.00 | 2.00 | 2.00 | 2.00 | 2.00 | 2.00 | 2.00 | 2.00 | 2.00 | 2.00 | 3.00 | 2.00 | 2.00 |
| EBF1 | 5 | 684 | 715 | 2.00 | 2.00 | 2.00 | 2.00 | 2.00 | 2.00 | 2.00 | 2.00 | 2.00 | 2.00 | 2.97 | 2.00 | 2.00 |
| NPM1 | 5 | 716 | 718 | 2.00 | 2.00 | 2.00 | 2.00 | 2.00 | 2.00 | 2.00 | 2.00 | 2.00 | 2.00 | 3.00 | 2.00 | 2.00 |
| FGFR4 | 5 | 719 | 732 | 1.96 | 2.00 | 2.00 | 2.00 | 2.00 | 2.00 | 2.00 | 2.00 | 2.00 | 2.00 | 2.82 | 2.00 | 2.00 |
| TPMT | 6 | 733 | 735 | 1.50 | 2.00 | 2.00 | 2.00 | 2.00 | 2.00 | 2.00 | 2.00 | 2.00 | 2.00 | 1.50 | 2.00 | 2.00 |
| HIST1H3B | 6 | 736 | 736 | 1.50 | 2.00 | 2.00 | 2.00 | 2.00 | 2.00 | 2.00 | 2.00 | 2.00 | 2.00 | 1.50 | 2.00 | 2.00 |
| DAXX | 6 | 737 | 737 | 1.50 | 2.00 | 2.00 | 2.00 | 2.00 | 2.00 | 2.00 | 2.00 | 2.00 | 2.00 | 1.50 | 2.00 | 2.00 |
| CCND3 | 6 | 738 | 739 | 1.50 | 2.00 | 2.00 | 2.00 | 2.00 | 2.00 | 2.00 | 2.00 | 2.00 | 2.00 | 1.50 | 2.00 | 2.00 |
| OCP1 | 6 | 740 | 740 | 1.50 | 2.00 | 2.00 | 2.00 | 2.00 | 2.00 | 2.00 | 2.00 | 2.00 | 2.00 | 1.50 | 2.00 | 2.00 |
| ARID1B | 6 | 741 | 836 | 1.55 | 2.00 | 2.00 | 2.00 | 2.00 | 2.00 | 2.00 | 2.00 | 2.00 | 2.00 | 1.55 | 2.00 | 2.00 |
| HDAC9 | 6 | 837 | 838 | 2.00 | 2.00 | 2.00 | 2.50 | 2.00 | 2.00 | 2.00 | 2.00 | 2.00 | 2.00 | 2.00 | 2.00 | 3.00 |
| IKZF1 | 7 | 839 | 869 | 1.87 | 1.63 | 2.00 | 2.50 | 2.00 | 2.00 | 2.00 | 2.52 | 2.00 | 2.00 | 2.00 | 2.00 | 3.00 |
| EGFR | 7 | 870 | 873 | 2.00 | 2.00 | 2.00 | 2.50 | 2.00 | 2.00 | 2.00 | 4.00 | 2.00 | 2.00 | 2.00 | 2.00 | 3.00 |
| CCP | 7 | 874 | 877 | 2.00 | 2.00 | 2.00 | 2.50 | 2.00 | 2.00 | 2.00 | 4.00 | 2.00 | 2.00 | 2.00 | 2.00 | 3.00 |
| CDK6 | 7 | 878 | 895 | 3.00 | 2.00 | 2.00 | 2.47 | 2.00 | 2.00 | 2.00 | 3.89 | 2.00 | 2.00 | 2.00 | 2.00 | 2.94 |
| MET | 7 | 896 | 913 | 2.00 | 2.00 | 2.00 | 2.50 | 2.00 | 2.00 | 2.00 | 3.00 | 2.00 | 2.00 | 2.00 | 2.00 | 3.00 |
| SMO | 7 | 914 | 927 | 2.00 | 2.00 | 2.00 | 2.46 | 2.00 | 2.00 | 2.00 | 2.93 | 2.00 | 2.00 | 2.00 | 2.00 | 2.93 |
| EZH2 | 7 | 928 | 935 | 2.00 | 2.00 | 2.00 | 2.38 | 2.00 | 2.00 | 2.00 | 2.75 | 2.00 | 2.13 | 2.00 | 2.00 | 2.75 |
| FGFR1 | 8 | 936 | 952 | 2.00 | 2.00 | 2.00 | 2.00 | 2.00 | 2.00 | 2.00 | 2.00 | 2.00 | 2.50 | 2.00 | 2.00 | 2.00 |
| CHD7 | 8 | 953 | 1088 | 2.00 | 2.00 | 1.97 | 2.00 | 2.00 | 2.00 | 2.00 | 2.00 | 2.00 | 2.50 | 1.99 | 2.00 | 2.00 |
| MYC | 8 | 1089 | 1098 | 2.00 | 2.00 | 1.50 | 2.00 | 2.00 | 2.00 | 2.00 | 2.00 | 2.00 | 2.50 | 1.50 | 2.00 | 2.00 |
| JAK2 | 8 | 1099 | 1131 | 2.00 | 1.56 | 2.00 | 2.00 | 1.12 | 2.00 | 1.12 | 2.00 | 2.00 | 2.00 | 2.00 | 2.00 | 2.00 |
| KDM4C | 9 | 1132 | 1132 | 2.00 | 1.50 | 2.00 | 2.00 | 1.00 | 2.00 | 1.00 | 2.00 | 2.00 | 2.00 | 2.00 | 2.00 | 2.00 |
| CDKN2A | 9 | 1133 | 1145 | 2.00 | 1.50 | 2.00 | 2.00 | 1.00 | 2.00 | 0.50 | 2.00 | 2.00 | 2.00 | 2.00 | 2.00 | 2.00 |
| CDKN2B | 9 | 1146 | 1150 | 2.00 | 1.60 | 2.00 | 2.00 | 2.00 | 2.00 | 2.00 | 2.00 | 2.00 | 2.00 | 2.00 | 2.00 | 2.00 |
| PAX5 | 9 | 1151 | 1162 | 2.00 | 1.50 | 2.00 | 2.00 | 2.00 | 2.00 | 2.00 | 2.00 | 2.00 | 2.00 | 2.00 | 2.00 | 2.00 |
| PTCH1 | 9 | 1163 | 1301 | 2.00 | 1.53 | 2.00 | 2.00 | 2.00 | 2.00 | 2.45 | 2.00 | 2.00 | 2.00 | 2.00 | 2.00 | 2.00 |
| NOTCH1 | 9 | 1302 | 1345 | 2.00 | 1.55 | 2.00 | 2.00 | 2.00 | 2.00 | 2.91 | 2.00 | 2.00 | 2.00 | 2.00 | 2.00 | 2.00 |
| GATA3 | 9 | 1346 | 1369 | 2.00 | 1.50 | 2.00 | 2.00 | 2.00 | 2.00 | 2.00 | 2.00 | 2.00 | 2.00 | 1.63 | 3.00 | 2.00 |
| PTEN | 10 | 1370 | 1388 | 2.00 | 1.61 | 2.00 | 2.00 | 2.00 | 2.00 | 2.00 | 2.00 | 2.00 | 2.00 | 2.00 | 2.79 | 2.00 |
| FAS | 10 | 1389 | 1405 | 2.00 | 1.53 | 2.00 | 2.00 | 2.00 | 2.00 | 2.00 | 2.00 | 2.00 | 2.00 | 2.00 | 2.94 | 2.00 |
| SUFU | 10 | 1406 | 1428 | 2.00 | 1.54 | 2.00 | 2.00 | 2.00 | 2.00 | 2.00 | 2.00 | 2.00 | 2.00 | 2.00 | 2.91 | 2.00 |
| NT5C2 | 10 | 1429 | 1438 | 2.00 | 1.50 | 2.00 | 2.00 | 2.00 | 2.00 | 2.00 | 2.00 | 2.00 | 2.00 | 1.65 | 3.00 | 2.00 |
| FGFR2 | 10 | 1439 | 1445 | 2.00 | 1.64 | 2.00 | 2.14 | 2.00 | 2.00 | 2.00 | 2.00 | 2.00 | 2.00 | 1.64 | 2.71 | 2.43 |
| MYOD1 | 11 | 1446 | 1458 | 2.00 | 2.00 | 2.00 | 2.46 | 2.00 | 2.00 | 2.00 | 2.00 | 2.00 | 2.00 | 2.00 | 2.00 | 3.38 |
| WT1 | 11 | 1459 | 1483 | 2.00 | 2.00 | 2.00 | 2.48 | 2.00 | 2.00 | 2.00 | 2.00 | 2.00 | 2.00 | 2.96 | 2.00 | 3.44 |
| CCND1 | 11 | 1484 | 1494 | 2.00 | 2.00 | 2.00 | 2.50 | 2.00 | 2.00 | 2.00 | 2.00 | 2.00 | 2.00 | 3.00 | 2.00 | 3.50 |
| EED | 11 | 1495 | 1520 | 2.00 | 2.00 | 2.00 | 1.58 | 2.00 | 2.00 | 2.00 | 1.15 | 2.00 | 2.00 | 1.58 | 2.00 | 1.46 |
| CBL | 11 | 1521 | 1529 | 2.00 | 2.00 | 2.00 | 1.50 | 2.00 | 2.00 | 1.00 | 1.00 | 2.00 | 2.00 | 1.50 | 2.00 | 1.00 |
| KRAS | 11 | 1530 | 1541 | 2.00 | 2.00 | 2.50 | 2.00 | 1.50 | 2.00 | 1.50 | 2.50 | 2.00 | 2.00 | 2.00 | 2.50 | 2.00 |
| KMT2D | 12 | 1542 | 1754 | 2.00 | 2.00 | 2.01 | 2.00 | 1.52 | 2.00 | 1.52 | 2.48 | 2.00 | 2.00 | 2.00 | 2.48 | 2.00 |
| ERBB3 | 12 | 1755 | 1766 | 2.00 | 2.00 | 2.00 | 2.00 | 1.50 | 2.00 | 1.50 | 2.50 | 2.00 | 2.00 | 2.00 | 2.50 | 2.00 |
| GLI1 | 12 | 1767 | 1779 | 2.00 | 2.00 | 2.00 | 2.00 | 1.50 | 2.00 | 1.50 | 2.50 | 2.00 | 2.00 | 2.00 | 2.50 | 2.00 |
| CDK4 | 12 | 1780 | 1787 | 2.00 | 2.00 | 2.00 | 2.00 | 1.50 | 2.00 | 1.50 | 2.50 | 2.00 | 2.00 | 2.00 | 2.50 | 2.00 |
| MDM2 | 12 | 1788 | 1802 | 2.00 | 2.00 | 2.40 | 2.00 | 1.50 | 2.00 | 1.50 | 2.50 | 2.00 | 2.00 | 2.00 | 3.50 | 2.00 |
| SOCS2 | 12 | 1803 | 1811 | 2.00 | 2.00 | 2.50 | 2.00 | 1.50 | 2.00 | 1.50 | 2.50 | 2.00 | 2.00 | 2.00 | 3.50 | 2.00 |
| SH2B3 | 12 | 1812 | 1815 | 2.00 | 2.00 | 2.38 | 2.00 | 1.63 | 2.00 | 1.63 | 2.38 | 2.00 | 2.00 | 2.00 | 3.13 | 2.00 |
| PTPN11 | 12 | 1816 | 1818 | 2.00 | 2.00 | 2.50 | 2.00 | 1.50 | 2.00 | 1.50 | 2.50 | 2.00 | 2.00 | 2.00 | 3.50 | 2.00 |
| NCOR2 | 12 | 1819 | 1825 | 2.00 | 2.00 | 2.50 | 2.21 | 1.71 | 2.00 | 1.71 | 2.29 | 2.00 | 2.00 | 2.64 | 3.50 | 2.00 |
| FLT3 | 12 | 1826 | 1827 | 2.00 | 2.00 | 2.50 | 2.50 | 2.00 | 2.00 | 2.00 | 2.00 | 2.00 | 2.00 | 3.50 | 3.50 | 2.00 |
| RB1 | 13 | 1828 | 1881 | 2.00 | 2.00 | 2.43 | 2.43 | 2.00 | 2.00 | 2.00 | 2.00 | 2.00 | 2.00 | 3.28 | 3.28 | 2.00 |
| PSMB5 | 13 | 1882 | 1893 | 2.42 | 2.00 | 1.58 | 2.00 | 2.00 | 2.00 | 2.00 | 2.00 | 2.00 | 2.17 | 1.58 | 2.00 | 2.00 |
| DICER1 | 14 | 1894 | 1988 | 2.47 | 1.98 | 2.00 | 3.41 | 2.00 | 2.00 | 2.00 | 2.47 | 2.00 | 2.47 | 1.53 | 2.00 | 2.00 |
| IGF1R | 15 | 1989 | 2093 | 2.71 | 1.70 | 1.70 | 2.39 | 2.00 | 2.00 | 2.00 | 1.76 | 2.00 | 2.00 | 2.00 | 2.41 | 2.00 |
| CREBBP | 16 | 2094 | 2105 | 2.00 | 1.58 | 1.63 | 2.00 | 2.00 | 2.00 | 2.00 | 2.00 | 2.00 | 2.00 | 2.00 | 2.04 | 2.00 |
| USP7 | 16 | 2106 | 2115 | 2.00 | 1.50 | 2.00 | 2.00 | 2.00 | 2.00 | 2.00 | 2.00 | 2.00 | 2.00 | 2.00 | 2.00 | 2.00 |
| TP53 | 16 | 2116 | 2139 | 2.48 | 2.00 | 2.00 | 2.00 | 2.00 | 2.00 | 2.00 | 2.00 | 2.00 | 1.52 | 2.00 | 2.00 | 2.00 |
| NF1 | 17 | 2140 | 2289 | 2.46 | 2.00 | 2.00 | 2.44 | 2.00 | 2.00 | 2.00 | 2.00 | 2.00 | 2.46 | 2.00 | 3.39 | 2.00 |
| SUZ12 | 17 | 2290 | 2332 | 2.47 | 2.00 | 2.00 | 2.34 | 2.00 | 2.00 | 2.00 | 2.35 | 2.00 | 2.47 | 2.00 | 3.40 | 2.35 |
| STAT5B | 17 | 2333 | 2335 | 2.50 | 2.67 | 2.00 | 2.00 | 2.00 | 2.00 | 2.00 | 3.50 | 2.00 | 2.50 | 2.00 | 3.50 | 3.50 |
| STAT3 | 17 | 2336 | 2342 | 2.50 | 3.00 | 2.00 | 2.00 | 2.00 | 2.00 | 2.00 | 3.50 | 2.00 | 2.50 | 2.29 | 3.50 | 3.50 |
| PPM1D | 17 | 2343 | 2346 | 2.50 | 3.00 | 2.00 | 5.50 | 2.00 | 2.00 | 6.50 | 3.50 | 2.00 | 2.50 | 3.00 | 3.50 | 3.50 |
| GNA13 | 17 | 2347 | 2361 | 2.47 | 2.93 | 2.00 | 4.47 | 2.00 | 2.00 | 4.57 | 3.40 | 2.00 | 2.47 | 2.93 | 3.40 | 3.40 |
| SETBP1 | 17 | 2362 | 2362 | 2.00 | 2.00 | 2.00 | 2.00 | 2.00 | 2.00 | 2.00 | 2.00 | 2.00 | 2.00 | 2.00 | 2.00 | 2.00 |
| TCF3 | 18 | 2363 | 2404 | 2.00 | 1.51 | 2.00 | 1.51 | 1.51 | 2.00 | 1.51 | 2.83 | 2.00 | 2.00 | 2.49 | 2.00 | 2.00 |
| SMARCA4 | 19 | 2405 | 2486 | 2.00 | 1.51 | 2.00 | 1.98 | 1.51 | 2.00 | 1.51 | 1.51 | 2.00 | 2.00 | 2.49 | 2.00 | 2.00 |
| CALR | 19 | 2487 | 2491 | 2.00 | 1.50 | 2.00 | 2.00 | 1.50 | 2.00 | 1.50 | 1.50 | 2.00 | 2.00 | 2.00 | 2.00 | 2.00 |
| JAK3 | 19 | 2492 | 2521 | 2.00 | 1.52 | 2.00 | 2.00 | 1.52 | 2.00 | 1.52 | 1.52 | 2.00 | 2.00 | 2.00 | 2.00 | 2.00 |
| CRLF1 | 19 | 2522 | 2544 | 2.00 | 1.54 | 2.00 | 2.00 | 1.54 | 2.00 | 1.54 | 1.54 | 2.00 | 2.00 | 2.00 | 2.00 | 2.00 |
| CEBPA | 19 | 2545 | 2555 | 2.00 | 1.82 | 2.00 | 2.00 | 1.82 | 2.00 | 1.82 | 1.82 | 2.00 | 2.00 | 2.00 | 2.00 | 2.00 |
| CIC | 19 | 2556 | 2561 | 2.00 | 1.50 | 2.00 | 2.00 | 1.50 | 2.00 | 1.50 | 1.50 | 2.00 | 2.00 | 2.00 | 2.00 | 2.00 |
| ASXL1 | 19 | 2562 | 2603 | 2.00 | 2.00 | 2.00 | 2.50 | 2.00 | 2.00 | 2.00 | 2.00 | 2.00 | 2.00 | 2.50 | 2.00 | 2.00 |
| RUNX1 | 20 | 2604 | 2623 | 2.00 | 1.50 | 2.00 | 3.00 | 2.00 | 2.00 | 1.50 | 2.00 | 2.00 | 2.00 | 2.00 | 2.00 | 2.00 |
| SMARCB1 | 21 | 2624 | 2643 | 2.00 | 2.00 | 2.00 | 2.00 | 2.00 | 2.00 | 2.00 | 2.00 | 2.00 | 2.00 | 2.00 | 2.00 | 2.00 |
| NF2 | 22 | 2644 | 2673 | 2.00 | 2.00 | 2.00 | 2.00 | 2.00 | 2.00 | 2.00 | 2.00 | 2.00 | 2.00 | 2.00 | 2.00 | 2.00 |
| EP300 | 22 | 2674 | 2677 | 2.00 | 2.00 | 2.00 | 2.00 | 2.00 | 2.00 | 2.00 | 2.00 | 2.00 | 2.00 | 2.00 | 2.00 | 1.00 |
| CRLF2 | 22 | 2678 | 2680 | 1.00 | 1.00 | 1.00 | 1.00 | 1.00 | 2.00 | 2.00 | 1.50 | 2.00 | 1.00 | 1.00 | 1.50 | 2.00 |
| DDX3X | X | 2681 | 2719 | 1.03 | 1.03 | 1.03 | 1.03 | 1.03 | 2.00 | 2.00 | 1.51 | 2.00 | 1.03 | 1.03 | 1.51 | 2.00 |
| KDM6A | X | 2720 | 2795 | 1.04 | 1.04 | 1.04 | 1.04 | 1.04 | 2.00 | 2.00 | 1.52 | 2.00 | 1.04 | 1.04 | 1.52 | 2.00 |
| GATA1 | X | 2796 | 2812 | 1.00 | 1.00 | 1.00 | 1.00 | 1.00 | 2.00 | 2.00 | 1.00 | 2.00 | 1.00 | 1.00 | 1.50 | 2.00 |
| ZMYM3 | X | 2813 | 2817 | 1.20 | 1.20 | 1.20 | 1.20 | 1.20 | 2.00 | 2.00 | 1.20 | 2.00 | 1.20 | 1.20 | 1.60 | 2.00 |
| ATRX | X | 2818 | 2938 | 1.07 | 1.07 | 1.07 | 1.07 | 1.07 | 2.00 | 2.00 | 0.65 | 2.00 | 1.07 | 1.07 | 1.53 | 2.00 |
| PRPS1 | X | 2939 | 2951 | 1.08 | 1.08 | 1.08 | 1.08 | 1.08 | 2.00 | 2.00 | 0.62 | 2.00 | 1.08 | 1.08 | 1.54 | 2.00 |
| XIAP | X | 2952 | 2972 | 1.00 | 1.00 | 1.00 | 1.00 | 1.00 | 2.00 | 2.00 | 0.50 | 2.00 | 1.00 | 1.00 | 1.50 | 2.00 |
| SH2D1A | X | 2973 | 2978 | 1.00 | 1.00 | 1.00 | 1.00 | 1.00 | 2.00 | 2.00 | 0.50 | 2.00 | 1.00 | 1.00 | 1.50 | 2.00 |
| PHF6 | X | 2979 | 2999 | 1.05 | 1.05 | 1.05 | 1.05 | 1.05 | 2.00 | 2.00 | 0.57 | 2.00 | 1.05 | 1.05 | 1.52 | 2.00 |
