# Supplementary material for: Integrated Genomic Profiling and Drug Screening of Patient-Derived Cultures Identifies Individualized Copy Number-Dependent Susceptibilities Involving PI3K Pathway and 17q Genes in Neuroblastoma
Source: Front Oncol. 2021 Oct 14;11:709525. doi: 10.3389/fonc.2021.709525 (PMC8551924; doi:10.3389/fonc.2021.709525)
Supplement: Supplementary file 14 [file Table_4.docx]

**Supplementary Table S4. Significantly corelated gene-drug pairs verified in public datasets, ranked by product of cytotoxicity and z-score.**

| **Gene-drug pair** | **OCCRA panel** | **Drug class MOA** | **Drug development clinical trial phase** | **z-score** | **Copy no. (mean ± S.D.)** | **Cytotoxicity (%, mean ± S.D.)** | **Reference (gene-drug association)** |
| --- | --- | --- | --- | --- | --- | --- | --- |
| GNA11 Cabozantinib (XL184, BMS-907351) | Non-CNV | Protein Tyrosine Kinase | Preclinical | 1.0 | 1.8 ± 0.3 | 20.5 ± 11.2 |  |
| JAK1 CYT387 | CNV | JAK/STAT | 1 | 0.7 | 2 ± 0.3 | 25.5 ± 14 | [1] |
| JAK3 Sorafenib Tosylate | CNV | MAPK | Preclinical | 0.7 | 1.9 ± 0.2 | 18.7 ± 8 | [2] |
| ERBB2 AZD4547 | CNV | Angiogenesis | 1 | 0.7 | 2.4 ± 0.6 | 12 ± 9.8 | [3] |
| MAP2K2 Sunitinib Malate | Non-CNV | Protein Tyrosine Kinase | Preclinical | 0.8 | 1.8 ± 0.3 | 7.6 ± 12.5 |  |
| MAP2K2 Crizotinib (PF-02341066) | Non-CNV | Protein Tyrosine Kinase | Preclinical | 0.9 | 1.8 ± 0.3 | 6.4 ± 7.3 |  |
| JAK3 Imatinib Mesylate (STI571) | CNV | Protein Tyrosine Kinase | Preclinical | 0.8 | 1.9 ± 0.2 | 7.1 ± 6 | [4] |
| MAP2K2 Imatinib Mesylate (STI571) | Non-CNV | Protein Tyrosine Kinase | Preclinical | 0.7 | 1.8 ± 0.3 | 7.1 ± 6 | [5] |
| ALK Sunitinib Malate | CNV | Protein Tyrosine Kinase | Preclinical | 0.7 | 2.1 ± 0.3 | 7.6 ± 12.5 |  |
| ALK WHI-P154 | CNV | JAK/STAT | Preclinical | 0.8 | 2.1 ± 0.3 | 5.9 ± 6.2 | [6] |
| PIK3CA Bosutinib (SKI-606) | CNV | Angiogenesis | Preclinical | 0.9 | 2.1 ± 0.3 | 3.6 ± 12.3 | [7] |
| KDR MGCD-265 | Non-CNV | Protein Tyrosine Kinase | 1 | 0.8 | 2 ± 0.2 | 1.3 ± 6.1 |  |
| FGFR1 Vandetanib (ZD6474) | CNV | Protein Tyrosine Kinase | Preclinical | 0.8 | 2 ± 0.1 | 1 ± 5.5 |  |
| ACVR1 Vandetanib (ZD6474) | Non-CNV | Protein Tyrosine Kinase | Preclinical | 0.8 | 2 ± 0.3 | 1 ± 5.5 |  |
| ACVR1 LDN-214117 | Non-CNV | TGF-beta/Smad | Preclinical | -0.7 | 2 ± 0.3 | 3 ± 9.1 | [8] |
| JAK2 TGX-221 | CNV | PI3K/Akt/mTOR | Preclinical | -0.7 | 1.8 ± 0.3 | 8 ± 5.4 |  |
| FGFR3 Brivanib (BMS-540215) | CNV | Protein Tyrosine Kinase | 3 | 1.0 | 2 ± 0.2 | -5.5 ± 3.1 |  |
| KDR Brivanib (BMS-540215) | Non-CNV | Protein Tyrosine Kinase | 3 | 1.0 | 2 ± 0.2 | -5.5 ± 3.1 |  |
| JAK3 AT9283 | CNV | JAK/STAT | 1 | -0.7 | 1.9 ± 0.2 | 64.3 ± 16.8 | [9] |

MOA: mechanism of action

References:

1. Pardanani, A., T. Lasho, G. Smith, C. J. Burns, E. Fantino, and A. Tefferi. 2009. “CYT387, a Selective JAK1/JAK2 Inhibitor: In Vitro Assessment of Kinase Selectivity and Preclinical Studies Using Cell Lines and Primary Cells from Polycythemia Vera Patients.” *Leukemia* 23(8):1441–45. doi: 10.1038/leu.2009.50.
2. Man, Cheuk Him, Tsz Kan Fung, Christa Ho, Heron H. C. Han, Howard C. H. Chow, Alvin C. H. Ma, William W. L. Choi, Si Lok, Alice M. S. Cheung, Connie Eaves, Yok Lam Kwong, and Anskar Y. H. Leung. 2012. “Sorafenib Treatment of FLT3-ITD + Acute Myeloid Leukemia: Favorable Initial Outcome and Mechanisms of Subsequent Nonresponsiveness Associated with the Emergence of a D835 Mutation.” *Blood* 119(22):5133–43. doi: 10.1182/blood-2011-06-363960.
3. Zhao, Qingxia, Amanda B. Parris, Erin W. Howard, Ming Zhao, Zhikun Ma, Zhiying Guo, Ying Xing, and Xiaohe Yang. 2017. “FGFR Inhibitor, AZD4547, Impedes the Stemness of Mammary Epithelial Cells in the Premalignant Tissues of MMTV-ErbB2 Transgenic Mice.” *Scientific Reports* 7(1). doi: 10.1038/s41598-017-11751-7.
4. Yagi, Kenta, Akira Shimada, and Toshiaki Sendo. 2018. “Pharmacological Inhibition of JAK3 Enhances the Antitumor Activity of Imatinib in Human Chronic Myeloid Leukemia.” *European Journal of Pharmacology* 825:28–33. doi: 10.1016/j.ejphar.2018.02.022.
5. Chu, Su, Melissa Holtz, Mamta Gupta, and Ravi Bhatia. 2004. “BCR/ABL Kinase Inhibition by Imatinib Mesylate Enhances MAP Kinase Activity in Chronic Myelogenous Leukemia CD34+ Cells.” *Blood* 103(8):3167–74. doi: 10.1182/blood-2003-04-1271.
6. Marzec, Michal, Monika Kasprzycka, Andrzej Ptasznik, Pawel Wlodarski, Qian Zhang, Niels Odum, and Mariusz A. Wasik. 2005. “Inhibition of ALK Enzymatic Activity in T-Cell Lymphoma Cells Induces Apoptosis and Suppresses Proliferation and STAT3 Phosphorylation Independently of Jak3.” *Laboratory Investigation* 85(12):1544–54. doi: 10.1038/labinvest.3700348.
7. Segrelles, Carmen, David Contreras, Elena M. Navarro, Carmen Gutiérrez-Muñoz, Ramón García-Escudero, Jesús M. Paramio, and Corina Lorz. 2018. “Bosutinib Inhibits EGFR Activation in Head and Neck Cancer.” *International Journal of Molecular Sciences* 19(7). doi: 10.3390/ijms19071824.
8. Carvalho, Diana, Kathryn R. Taylor, Nagore Gene Olaciregui, Valeria Molinari, Matthew Clarke, Alan Mackay, Ruth Ruddle, Alan Henley, Melanie Valenti, Angela Hayes, Alexis De Haven Brandon, Suzanne A. Eccles, Florence Raynaud, Aicha Boudhar, Michelle Monje, Sergey Popov, Andrew S. Moore, Jaume Mora, Ofelia Cruz, Mara Vinci, Paul E. Brennan, Alex N. Bullock, Angel Montero Carcaboso, and Chris Jones. 2019. “ALK2 Inhibitors Display Beneficial Effects in Preclinical Models of ACVR1 Mutant Diffuse Intrinsic Pontine Glioma.” *Communications Biology* 2(1). doi: 10.1038/s42003-019-0420-8.
9. Kimura, Shinya. 2010. “AT-9283, a Small-Molecule Multi-Targeted Kinase Inhibitor for the Potential Treatment of Cancer.” *Current Opinion in Investigational Drugs* 11(12):1442–49.
