# Supplementary material for: Integrated Genomic Profiling and Drug Screening of Patient-Derived Cultures Identifies Individualized Copy Number-Dependent Susceptibilities Involving PI3K Pathway and 17q Genes in Neuroblastoma
Source: Front Oncol. 2021 Oct 14;11:709525. doi: 10.3389/fonc.2021.709525 (PMC8551924; doi:10.3389/fonc.2021.709525)
Supplement: Supplementary file 15 [file Table_5.docx]

**Supplementary Table S5. Fifty most significant positively-correlated gene-drug pairs not verified in public datasets, ranked by product of cytotoxicity and z-score.**

| **Gene-drug pair** | **OCCRA panel** | **Drug class MOA** | **Drug development clinical trial phase** | **z-score** | **Copy no. (mean ± S.D.)** | **Cytotoxicity (%, mean ± S.D.)** |
| --- | --- | --- | --- | --- | --- | --- |
| GNA13 PIK-75 | Non-CNV | PI3K/Akt/mTOR | Preclinical | 1.4 | 2.93 ± 0.9 | 78.87 ± 14 |
| GNA13 P276-00 | Non-CNV | Cell Cycle | Preclinical | 1.2 | 2.93 ± 0.9 | 79.72 ± 14 |
| PPM1D PIK-75 | Non-CNV | PI3K/Akt/mTOR | Preclinical | 1.2 | 3.19 ± 1.3 | 78.87 ± 14 |
| GNA13 Flavopiridol (Alvocidib) | Non-CNV | Cell Cycle | 1 | 1.1 | 2.93 ± 0.9 | 75.38 ± 15.8 |
| GNA13 Flavopiridol HCl | Non-CNV | Cell Cycle | 1 | 1.1 | 2.93 ± 0.9 | 73.43 ± 16 |
| PPM1D Flavopiridol HCl | Non-CNV | Cell Cycle | 1 | 1.1 | 3.19 ± 1.3 | 73.43 ± 16 |
| PPM1D P276-00 | Non-CNV | Cell Cycle | Preclinical | 1 | 3.19 ± 1.3 | 79.72 ± 14 |
| GNA13 SNS-032 (BMS-387032) | Non-CNV | Cell Cycle | 1 | 1.1 | 2.93 ± 0.9 | 70.79 ± 17.3 |
| PPM1D Flavopiridol (Alvocidib) | Non-CNV | Cell Cycle | 1 | 1 | 3.19 ± 1.3 | 75.38 ± 15.8 |
| MAP2K2 BGT226 (NVP-BGT226) | Non-CNV | PI3K/Akt/mTOR | 1 | 0.9 | 1.85 ± 0.3 | 83.56 ± 15.3 |
| GNA11 BGT226 (NVP-BGT226) | Non-CNV | PI3K/Akt/mTOR | 1 | 0.9 | 1.85 ± 0.3 | 83.56 ± 15.3 |
| PPM1D SNS-032 (BMS-387032) | Non-CNV | Cell Cycle | 1 | 1 | 3.19 ± 1.3 | 70.79 ± 17.3 |
| GNA13 Dinaciclib (SCH727965) | Non-CNV | Cell Cycle | 3 | 1 | 2.93 ± 0.9 | 70.71 ± 16.2 |
| PPM1D Dinaciclib (SCH727965) | Non-CNV | Cell Cycle | 3 | 1 | 3.19 ± 1.3 | 70.71 ± 16.2 |
| GNA13 AT7519 | Non-CNV | Cell Cycle | 1 | 1 | 2.93 ± 0.9 | 69.99 ± 21.6 |
| GNA13 Tivantinib (ARQ 197) | Non-CNV | Protein Tyrosine Kinase | 3 | 0.9 | 2.93 ± 0.9 | 76.71 ± 10.8 |
| GNA13 Staurosporine | Non-CNV | TGF-beta/Smad | 3 | 0.7 | 2.93 ± 0.9 | 95.67 ± 5.3 |
| MPL BGT226 (NVP-BGT226) | Non-CNV | PI3K/Akt/mTOR | 1 | 0.8 | 1.85 ± 0.4 | 83.56 ± 15.3 |
| JAK1 AT7519 | CNV | Cell Cycle | 1 | 0.9 | 1.96 ± 0.3 | 69.99 ± 21.6 |
| PPM1D Tivantinib (ARQ 197) | Non-CNV | Protein Tyrosine Kinase | 3 | 0.8 | 3.19 ± 1.3 | 76.71 ± 10.8 |
| TCF3 AZD3463 | Non-CNV | Protein Tyrosine Kinase | Preclinical | 0.9 | 1.96 ± 0.4 | 65.18 ± 16.7 |
| JAK1 BGT226 (NVP-BGT226) | CNV | PI3K/Akt/mTOR | 1 | 0.7 | 1.96 ± 0.3 | 83.56 ± 15.3 |
| JAK1 AZD2014 | CNV | PI3K/Akt/mTOR | 2 | 0.9 | 1.96 ± 0.3 | 63.68 ± 8.8 |
| JAK1 Torin 2 | CNV | PI3K/Akt/mTOR | Preclinical | 0.8 | 1.96 ± 0.3 | 70.41 ± 7.5 |
| JAK1 PF-477736 | CNV | Cell Cycle | 1 | 1 | 1.96 ± 0.3 | 56.18 ± 18.4 |
| PPM1D AT7519 | Non-CNV | Cell Cycle | 1 | 0.8 | 3.19 ± 1.3 | 69.99 ± 21.6 |
| MET PIK-75 | CNV | PI3K/Akt/mTOR | Preclinical | 0.7 | 2.19 ± 0.4 | 78.87 ± 14 |
| BRAF PIK-75 | CNV | PI3K/Akt/mTOR | Preclinical | 0.7 | 2.17 ± 0.3 | 78.87 ± 14 |
| EZH2 PIK-75 | Non-CNV | PI3K/Akt/mTOR | Preclinical | 0.7 | 2.19 ± 0.4 | 78.87 ± 14 |
| SMO PIK-75 | Non-CNV | PI3K/Akt/mTOR | Preclinical | 0.7 | 2.19 ± 0.4 | 78.87 ± 14 |
| JAK1 AZD8055 | CNV | PI3K/Akt/mTOR | 1 | 0.8 | 1.96 ± 0.3 | 67.68 ± 8.6 |
| JAK1 VS-5584 (SB2343) | CNV | PI3K/Akt/mTOR | 1 | 0.8 | 1.96 ± 0.3 | 65.24 ± 7.6 |
| JAK1 GDC-0980 (RG7422) | CNV | PI3K/Akt/mTOR | 2 | 0.8 | 1.96 ± 0.3 | 64.5 ± 7.8 |
| JAK1 Rigosertib (ON-01910) | CNV | Cell Cycle | 3 | 0.9 | 1.96 ± 0.3 | 57.16 ± 10.5 |
| MPL AZD2014 | Non-CNV | PI3K/Akt/mTOR | 2 | 0.8 | 1.85 ± 0.4 | 63.68 ± 8.8 |
| JAK1 JNK Inhibitor IX | CNV | MAPK | Preclinical | 0.9 | 1.96 ± 0.3 | 55.62 ± 11 |
| JAK1 TAK-901 | CNV | Cell Cycle | 1 | 0.9 | 1.96 ± 0.3 | 51.56 ± 17.6 |
| TCF3 PP242 | Non-CNV | PI3K/Akt/mTOR | Preclinical | 0.8 | 1.96 ± 0.4 | 57.56 ± 9.9 |
| MPL PP242 | Non-CNV | PI3K/Akt/mTOR | Preclinical | 0.8 | 1.85 ± 0.4 | 57.56 ± 9.9 |
| H3F3A WYE-125132 (WYE-132) | CNV | PI3K/Akt/mTOR | Preclinical | 0.7 | 2.15 ± 0.3 | 65.64 ± 7.4 |
| MDM4 WYE-125132 (WYE-132) | CNV | PI3K/Akt/mTOR | Preclinical | 0.7 | 2.15 ± 0.3 | 65.64 ± 7.4 |
| MPL VS-5584 (SB2343) | Non-CNV | PI3K/Akt/mTOR | 1 | 0.7 | 1.85 ± 0.4 | 65.24 ± 7.6 |
| JAK1 AZD3463 | CNV | Protein Tyrosine Kinase | Preclinical | 0.7 | 1.96 ± 0.3 | 65.18 ± 16.7 |
| KDM6A OSI-027 | Non-CNV | PI3K/Akt/mTOR | Preclinical | 0.9 | 1.41 ± 0.4 | 50.54 ± 7.8 |
| CRLF2 OSI-027 | Non-CNV | PI3K/Akt/mTOR | Preclinical | 0.9 | 1.38 ± 0.4 | 50.54 ± 7.8 |
| DDX3X OSI-027 | Non-CNV | PI3K/Akt/mTOR | Preclinical | 0.9 | 1.4 ± 0.4 | 50.54 ± 7.8 |
| MPL GDC-0980 (RG7422) | Non-CNV | PI3K/Akt/mTOR | 2 | 0.7 | 1.85 ± 0.4 | 64.5 ± 7.8 |
| TCF3 GDC-0980 (RG7422) | Non-CNV | PI3K/Akt/mTOR | 2 | 0.7 | 1.96 ± 0.4 | 64.5 ± 7.8 |
| JAK1 PF-3758309 | CNV | Cytoskeletal Signaling | Preclinical | 0.8 | 1.96 ± 0.3 | 55.25 ± 18 |
| TCF3 TG101209 | Non-CNV | JAK/STAT | Preclinical | 0.9 | 1.96 ± 0.4 | 48.46 ± 16.2 |
| GNA13 PIK-75 | Non-CNV | PI3K/Akt/mTOR | Preclinical | 1.4 | 2.93 ± 0.9 | 78.87 ± 14 |

MOA: mechanism of action
