# Supplementary material for: Integrated Genomic Profiling and Drug Screening of Patient-Derived Cultures Identifies Individualized Copy Number-Dependent Susceptibilities Involving PI3K Pathway and 17q Genes in Neuroblastoma
Source: Front Oncol. 2021 Oct 14;11:709525. doi: 10.3389/fonc.2021.709525 (PMC8551924; doi:10.3389/fonc.2021.709525)
Supplement: Supplementary file 16 [file Table_6.docx]

**Supplementary Table S6. Fifty most significant negatively-correlated gene-drug pairs not verified in public datasets, ranked by product of cytotoxicity and z-score.**

| **Gene-drug pair** | **OCCRA panel** | **Drug class MOA** | **Drug development clinical trial phase** | **z-score** | **Copy no. (mean ± S.D.)** | **Cytotoxicity (%, mean ± S.D.)** |
| --- | --- | --- | --- | --- | --- | --- |
| CBL PIK-75 | Non-CNV | PI3K/Akt/mTOR | Preclinical | -1.4 | 1.69 ± 0.4 | 78.87 ± 14 |
| CBL Flavopiridol (Alvocidib) | Non-CNV | Cell Cycle | 1 | -1.3 | 1.69 ± 0.4 | 75.38 ± 15.8 |
| CBL Flavopiridol HCl | Non-CNV | Cell Cycle | 1 | -1.2 | 1.69 ± 0.4 | 73.43 ± 16 |
| CBL SNS-032 (BMS-387032) | Non-CNV | Cell Cycle | 1 | -1.2 | 1.69 ± 0.4 | 70.79 ± 17.3 |
| CBL P276-00 | Non-CNV | Cell Cycle | Preclinical | -1 | 1.69 ± 0.4 | 79.72 ± 14 |
| CBL Dinaciclib (SCH727965) | Non-CNV | Cell Cycle | 3 | -1.1 | 1.69 ± 0.4 | 70.71 ± 16.2 |
| CBL Tivantinib (ARQ 197) | Non-CNV | Protein Tyrosine Kinase | 3 | -1 | 1.69 ± 0.4 | 76.71 ± 10.8 |
| ABL2 Staurosporine | CNV | TGF-beta/Smad | 3 | -0.8 | 2.04 ± 0.1 | 95.67 ± 5.3 |
| EED PIK-75 | Non-CNV | PI3K/Akt/mTOR | Preclinical | -0.9 | 1.83 ± 0.3 | 78.87 ± 14 |
| CBL INK 128 (MLN0128) | Non-CNV | PI3K/Akt/mTOR | 1 | -1 | 1.69 ± 0.4 | 70.52 ± 7.3 |
| DNMT3A P276-00 | Non-CNV | Cell Cycle | Preclinical | -0.8 | 2.08 ± 0.3 | 79.72 ± 14 |
| DNMT3A Flavopiridol (Alvocidib) | Non-CNV | Cell Cycle | 1 | -0.8 | 2.08 ± 0.3 | 75.38 ± 15.8 |
| EED Flavopiridol (Alvocidib) | Non-CNV | Cell Cycle | 1 | -0.8 | 1.83 ± 0.3 | 75.38 ± 15.8 |
| CBL WYE-125132 (WYE-132) | Non-CNV | PI3K/Akt/mTOR | Preclinical | -0.9 | 1.69 ± 0.4 | 65.64 ± 7.4 |
| EED Flavopiridol HCl | Non-CNV | Cell Cycle | 1 | -0.8 | 1.83 ± 0.3 | 73.43 ± 16 |
| EED SNS-032 (BMS-387032) | Non-CNV | Cell Cycle | 1 | -0.8 | 1.83 ± 0.3 | 70.79 ± 17.3 |
| DNMT3A Dinaciclib (SCH727965) | Non-CNV | Cell Cycle | 3 | -0.8 | 2.08 ± 0.3 | 70.71 ± 16.2 |
| CBL Torin 2 | Non-CNV | PI3K/Akt/mTOR | Preclinical | -0.8 | 1.69 ± 0.4 | 70.41 ± 7.5 |
| CBL AT7519 | Non-CNV | Cell Cycle | 1 | -0.8 | 1.69 ± 0.4 | 69.99 ± 21.6 |
| ASXL2 P276-00 | Non-CNV | Cell Cycle | Preclinical | -0.7 | 2.07 ± 0.3 | 79.72 ± 14 |
| CBL GSK2126458 (GSK458) | Non-CNV | PI3K/Akt/mTOR | 1 | -0.8 | 1.69 ± 0.4 | 67.78 ± 7.7 |
| DNMT3A Tivantinib (ARQ 197) | Non-CNV | Protein Tyrosine Kinase | 3 | -0.7 | 2.08 ± 0.3 | 76.71 ± 10.8 |
| ASXL2 Flavopiridol (Alvocidib) | Non-CNV | Cell Cycle | 1 | -0.7 | 2.07 ± 0.3 | 75.38 ± 15.8 |
| CBL CHIR-124 | Non-CNV | Cell Cycle | Preclinical | -1 | 1.69 ± 0.4 | 50.74 ± 12.2 |
| CBL PF-3758309 | Non-CNV | Cytoskeletal Signaling | Preclinical | -0.9 | 1.69 ± 0.4 | 55.25 ± 18 |
| ASXL2 Dinaciclib (SCH727965) | Non-CNV | Cell Cycle | 3 | -0.7 | 2.07 ± 0.3 | 70.71 ± 16.2 |
| CBL PF-04691502 | Non-CNV | PI3K/Akt/mTOR | 2 | -0.7 | 1.69 ± 0.4 | 69 ± 6.9 |
| NF1 GSK2126458 (GSK458) | Non-CNV | PI3K/Akt/mTOR | 1 | -0.7 | 2.21 ± 0.4 | 67.78 ± 7.7 |
| SUZ12 GSK2126458 (GSK458) | Non-CNV | PI3K/Akt/mTOR | 1 | -0.7 | 2.21 ± 0.4 | 67.78 ± 7.7 |
| DNMT3A AT9283 | Non-CNV | JAK/STAT | 1 | -0.7 | 2.08 ± 0.3 | 64.29 ± 16.8 |
| CBL AT9283 | Non-CNV | JAK/STAT | 1 | -0.7 | 1.69 ± 0.4 | 64.29 ± 16.8 |
| CALR AT9283 | Non-CNV | JAK/STAT | 1 | -0.7 | 1.85 ± 0.2 | 64.29 ± 16.8 |
| CEBPA AT9283 | Non-CNV | JAK/STAT | 1 | -0.7 | 1.94 ± 0.1 | 64.29 ± 16.8 |
| CIC AT9283 | Non-CNV | JAK/STAT | 1 | -0.7 | 1.85 ± 0.2 | 64.29 ± 16.8 |
| CRLF1 AT9283 | Non-CNV | JAK/STAT | 1 | -0.7 | 1.86 ± 0.2 | 64.29 ± 16.8 |
| IL7R IMD 0354 | Non-CNV | NF-KB | Preclinical | -0.9 | 2.08 ± 0.3 | 45.55 ± 8.2 |
| CSF1R IMD 0354 | Non-CNV | NF-KB | Preclinical | -0.9 | 2.08 ± 0.3 | 45.55 ± 8.2 |
| APC IMD 0354 | Non-CNV | NF-KB | Preclinical | -0.9 | 2.07 ± 0.3 | 45.55 ± 8.2 |
| EBF1 IMD 0354 | Non-CNV | NF-KB | Preclinical | -0.9 | 2.07 ± 0.3 | 45.55 ± 8.2 |
| NPM1 IMD 0354 | Non-CNV | NF-KB | Preclinical | -0.9 | 2.08 ± 0.3 | 45.55 ± 8.2 |
| FGFR4 IMD 0354 | CNV | NF-KB | Preclinical | -0.9 | 2.07 ± 0.2 | 45.55 ± 8.2 |
| PDGFRB IMD 0354 | Non-CNV | NF-KB | Preclinical | -0.9 | 2.08 ± 0.3 | 45.55 ± 8.2 |
| PIK3R1 IMD 0354 | Non-CNV | NF-KB | Preclinical | -0.9 | 2.07 ± 0.2 | 45.55 ± 8.2 |
| DNMT3A KX2-391 | Non-CNV | Angiogenesis | 1 | -0.8 | 2.08 ± 0.3 | 51.21 ± 9 |
| ASXL1 Milciclib (PHA-848125) | Non-CNV | Cell Cycle | 2 | -0.7 | 2.08 ± 0.2 | 55.2 ± 8.8 |
| RUNX1 R406 (free base) | Non-CNV | Angiogenesis | 2 | -1.1 | 2 ± 0.3 | 34.59 ± 10.4 |
| GNA11 IMD 0354 | Non-CNV | NF-KB | Preclinical | -0.8 | 1.85 ± 0.3 | 45.55 ± 8.2 |
| MAP2K2 IMD 0354 | Non-CNV | NF-KB | Preclinical | -0.8 | 1.85 ± 0.3 | 45.55 ± 8.2 |
| ASXL2 KX2-391 | Non-CNV | Angiogenesis | 1 | -0.7 | 2.07 ± 0.3 | 51.21 ± 9 |
| RUNX1 Pelitinib (EKB-569) | Non-CNV | Protein Tyrosine Kinase | 1 | -0.7 | 2 ± 0.3 | 50.98 ± 13.2 |

MOA: mechanism of action
